# Stereocontrolled Total Synthesis of Bastimolide B Using Iterative Homologation of Boronic Esters

**DOI:** 10.1021/jacs.2c03192

**Published:** 2022-05-02

**Authors:** Daniele Fiorito, Selbi Keskin, Joseph M. Bateman, Malcolm George, Adam Noble, Varinder K. Aggarwal

**Affiliations:** School of Chemistry, University of Bristol, Cantock’s Close, Bristol BS8 1TS, U.K.

## Abstract

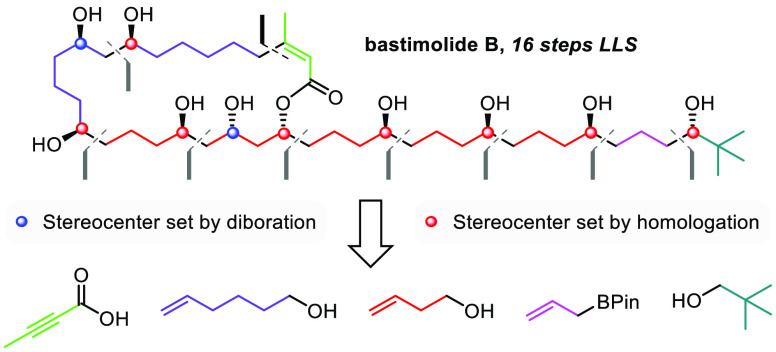

Bastimolide B is a polyhydroxy macrolide isolated from marine cyanobacteria displaying antimalarial activity. It features a dense array of hydroxylated stereogenic centers with 1,5-relationships along a hydrocarbon chain. These 1,5-polyols represent a particularly challenging motif for synthesis, as the remote position of the stereocenters hampers stereocontrol. Herein, we present a strategy for 1,5-polyol stereocontrolled synthesis based on iterative boronic ester homologation with enantiopure magnesium carbenoids. By merging boronic ester homologation and transition-metal-catalyzed alkene hydroboration and diboration, the acyclic backbone of bastimolide B was rapidly assembled from readily available building blocks with full control over the remote stereocenters, enabling the total synthesis to be completed in 16 steps (LLS).

Polyketides are arguably the most important class of natural products, having been extensively mined, studied, and exploited as therapeutic agents for the promotion of human health.^1^ Their complex structures coupled with their significant biological activity have fueled intense interest in their synthesis.^2^ Indeed, the stereoselective synthesis of polypropionates/polyacetates represents one of the crowning achievements of synthesis in the 20th century.^3−10^ Despite the advances in polyketide synthesis, several challenges still remain, such as the stereoselective construction of 1,5-stereogenic centers.^11^ Of particular interest is the construction of 1,5-polyols, as they occur in many polyketides.^12^ The current state-of-the-art technology to access 1,5-diols is through a three-step sequence comprising stereoselective aldehyde allylation, cross-metathesis with acrolein, and alcohol protection (Figure 1A).^13^ This method, like numerous others,^4,14−18^ is attractive because it can be iterated to access 1,5-polyols.

**Figure 1:**
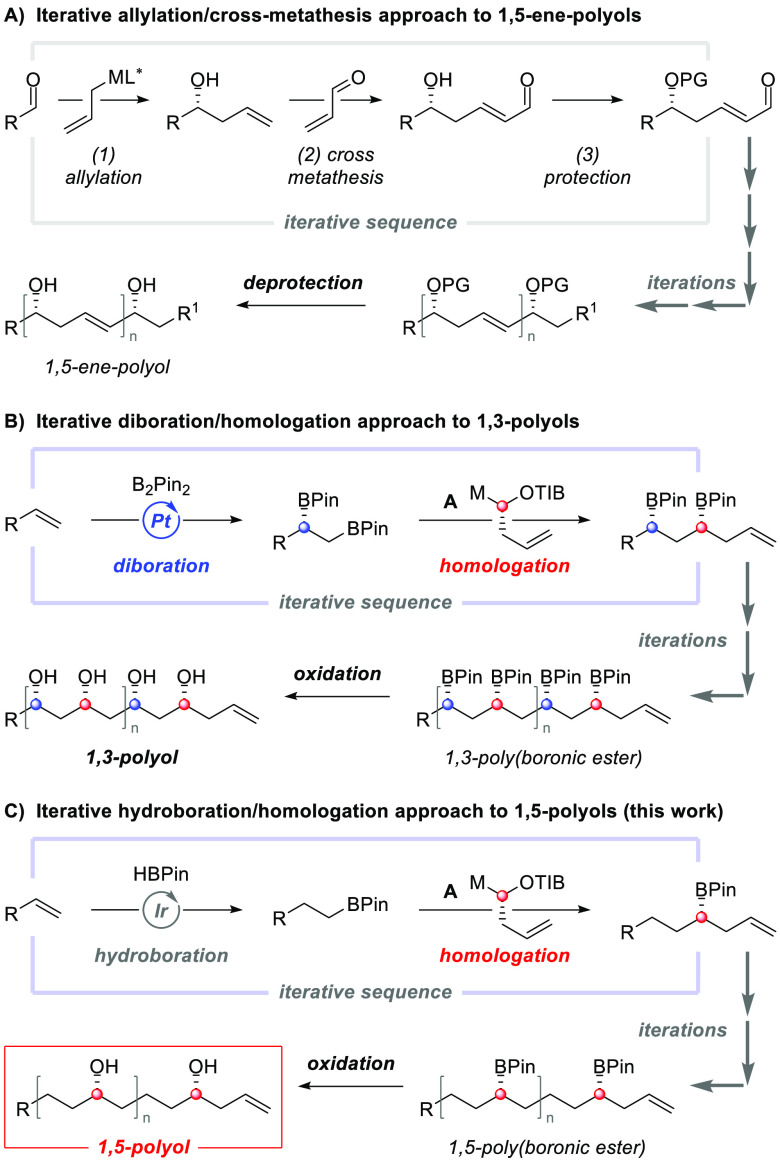
Iterative strategies toward the stereocontrolled synthesis of polyols.

Iterative methods are ideal in the synthesis of molecules bearing common repeat motifs as they simplify not only synthesis but also analysis (retrosynthesis) because they allow disconnections around the common repeating building blocks.^19^ Indeed, by use of a comprehensive knowledge base of individual reactions, iterative methods can also now be recognized by computer algorithms in the construction of complex molecules.^18^ We recently reported the iterative synthesis of 1,3-polyols through a sequence of asymmetric alkene diboration and selective homologation of the resulting primary boronic ester with a metalated butenyl TIB ester **A** (Figure 1B, M = Li, MgCl, TIB = 2,4,6-triisopropylbenzoyl).^20^ We reasoned that performing anti-Markovnikov hydroboration, instead of diboration, followed by homologation with the same metalated butenyl TIB ester **A** could provide an iterative strategy for the construction of 1,5-polyols in just two steps per iteration rather than three (Figure 1C).

In this paper we describe the success of this approach and its application to the first total synthesis of bastimolide B (**1**, Scheme 1), one of the most complex polyketides, featuring 10 hydroxylated stereocenters, six of which feature 1,5-relationships.

**Scheme 1:**
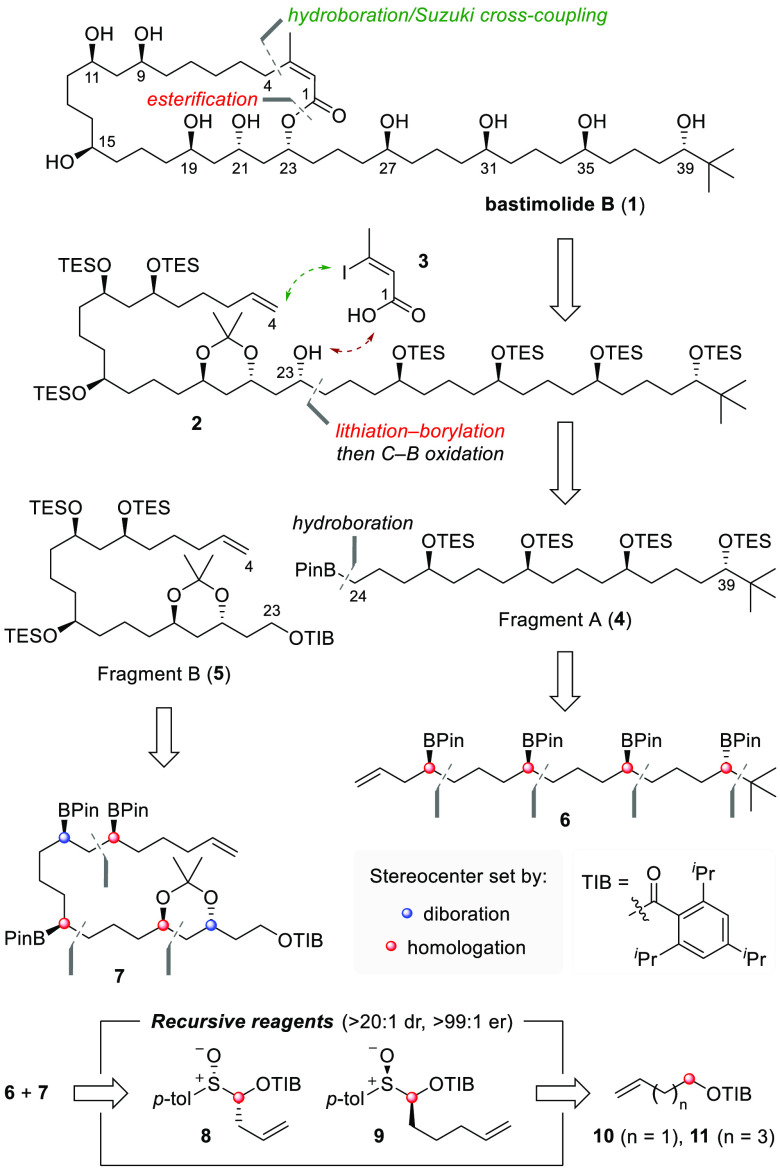
Retrosynthetic Approach to Bastimolide B

Bastimolide B (**1**) is a 24-membered macrolide whose structure and stereochemistry were assigned by analogy to its 40-membered ring analogue bastimolide A (C1–C39 lactone formation), which was characterized by X-ray analysis.^21,22^ The interest in bastimolides A and B stems from their potent antimalarial activity against multidrug-resistant strains of *P. falciparium*.^23^ Although synthetic studies toward these natural products have been reported, there have been no total syntheses to date.^24^ Interestingly, a recent report described the use of a computer algorithm to propose a plausible route to bastimolide A in 43 steps [longest linear sequence (LLS)] by using the most efficient iterative homologation reactions currently available.^18^ Herein, we describe a novel stereocontrolled approach to 1,5-polyols based on iterative boronic ester homologations and its application to the total synthesis of **1** in just 16 steps LLS.

In our retrosynthetic analysis of **1**, we envisioned constructing the macrocycle from terminal alkene-containing polyol **2** using (*Z*)-iodocrotonic acid (**3**) as a synthetic linchpin, which could undergo esterification followed by a stereoretentive C(sp^3^)–C(sp^2^) Suzuki cross-coupling macrocyclization (Scheme 1).

Polyol **2** could be obtained in a convergent manner by a late-stage lithiation–borylation reaction between two equally complex fragments A (**4**, C24–C39) and B (**5**, C4–C23). These fragments would be derived from diastereo- and enantiopure poly(boronic esters) **6** and **7**, respectively. Crucially, **6** and **7** could be rapidly constructed by using the new hydroboration/homologation approach for the iterative assembly of 1,5-polyols with sulfoxide **8** as the precursor of metalated TIB ester **A** (Scheme 1).^25^ In addition, we reasoned that simple modification of this iterative approach by employing elongated TIB ester **9** in the homologation followed by diboration would enable construction of the alternating 1,5- and 1,3-related stereogenic centers in **7**. Overall, our retrosynthetic approach combines efficient iterative reactions in the synthesis of key building blocks with late-stage fragment coupling, culminating in a convergent synthesis with low step count. One distinctive aspect of our synthesis is that both the iterative reactions and the key fragment coupling employ our boronic ester homologations, which occur with exquisite reagent control.

We began our synthesis by targeting fragment A, which required initial synthesis of the sterically hindered neopentyl boronic ester **15** (Scheme 2A). In principle, there are two possible homologation reactions to construct **15**: (1) reaction of metalated butenyl TIB ester **A** with ^*t*^BuBPin or (2) reaction of a metalated neopentyl TIB ester with allylBpin. Unfortunately, the first was unsuccessful due to the failure of **A** (M = Li) to form a boronate complex with ^*t*^BuBPin so we explored the second method.^26^ Carbenoid formation by deprotonation of neopentyl TIB ester **12** with ^*s*^BuLi in the presence of (−)-sparteine (*t*_1/2_ = ∼90 min, −78 °C) followed by addition of allylBPin **14** gave **15** in only 17% ^1^H NMR yield but 98:2 er. We reasoned that the high steric demand of the neopentyl carbenoid resulted in low nucleophilicity toward the boronic ester. To reduce steric hindrance, we elected to use diamine-free conditions in the homologation, which could be achieved by generating the carbenoid via tin–lithium exchange of stannane **13**.^27^ Thus, treatment of **13** with ^*n*^BuLi followed by trapping with allylBPin **14** and heating delivered homoallylic boronic ester **15** in high yield (94% NMR yield) and enantioselectivity (98:2 er). With **15** in hand, we explored our proposed iterative hydroboration/boronic ester homologation sequence for the construction of 1,5-related stereogenic centers. Without purification, crude **15** was directly engaged in an iridium-catalyzed hydroboration with HBPin to give exclusively the anti-Markovnikov 1,4-bis(boronic ester) **16** in 72% yield over two steps on a multigram scale.^28^ Homologation of **16** with the magnesiated carbenoid generated *in situ* from bench-stable sulfoxide **8** revealed 1,5-bis(boronic ester) **17** as a single diastereoisomer. The magnesium–sulfoxide exchange and subsequent borylation and 1,2-migration proceeded under mild reaction conditions by using ^*i*^PrMgCl·LiCl, and no competing addition to the secondary boronic ester was observed. Pleasingly, two further iterations of this hydroboration/homologation sequence provided 1,5-tetra(boronic ester) **6** in 46% yield over four steps on a gram scale. Notably, homologation of boronic ester **20**, bearing three secondary boronic esters and one primary boronic ester, resulted in exclusive chemoselective reaction of the primary boronic ester in 89% yield. Oxidation of the tetra(boronic ester) yielded stereochemically pure tetraol **21** with the desired stereochemistry at C27, C31, C35, and C39. Protection of **21** as triethylsilyl ether **22** followed by catalytic hydroboration of the terminal olefin provided primary boronic ester **4** (fragment A) in 11 steps from **12** (12 steps from commercially available neopentanol).

**Scheme 2:**
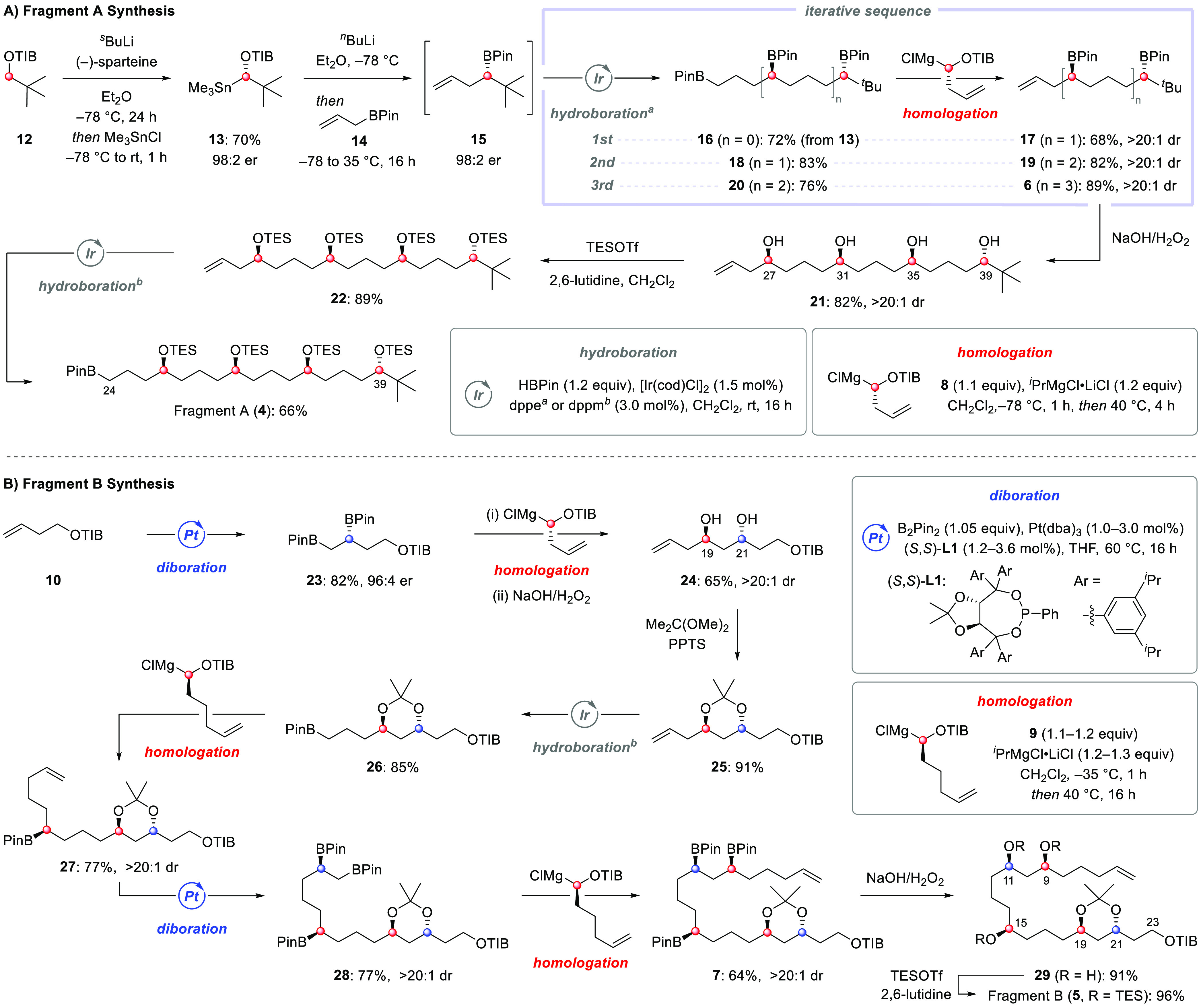
Synthesis of Fragments A (**4**) and B (**5**)

The synthesis of fragment B started from homoallylic TIB ester **10** (Scheme 2B), which is also the precursor of recursive reagent sulfoxide **8**; this maximized the convergency of our approach by reducing the number of distinct building blocks for the construction of bastimolide B. Enantioselective Pt-catalyzed diboration with B_2_Pin_2_ gave enantioenriched 1,2-bis(boronic ester) **23** with 96:4 er on a multigram scale.^29^ Homologation of **23** with sulfoxide **8**, followed by oxidation of the 1,3-bis(boronic ester), yielded diol **24** as a single diastereoisomer after chromatographic purification (>20:1 dr). The *anti* configuration of the diol was confirmed by ^13^C NMR analysis of acetonide **25**.^30^ Oxidation and protection of the C19–C21 diol at this stage not only ensured diastereopurity of this intermediate but also provided a suitable protecting group to aid in the late-stage lithiation–borylation at C23 for the coupling of fragments A and B (*vide infra*). Iridium-catalyzed hydroboration of the terminal olefin with pinacolborane gave primary boronic ester **26** in 85% yield. Homologation of **26** with sulfoxide **9** proceeded smoothly to afford secondary boronic ester **27** as a single diastereoisomer in 77% yield. Once the remote stereocenter at C15 was set via reagent-controlled homologation, catalyst-controlled diastereoselective diboration of the terminal olefin was conducted with (*S*,*S*)-**L1** to afford the 1,2-bis(boronic ester) **28**. One further homologation with the recursive reagent **9** allowed the installation of the desired 1,5,7-tris(boronic ester) with the correct stereochemistry at C9, C11, and C15. Simultaneous oxidation of the three boronic esters, followed by *O*-TES protection, led to fragment B (**5**). Overall, our iterative approach utilizing a combination of diboration and hydroboration coupled with boron homologations with recursive reagents enabled fragment B to be constructed in 10 steps from **10** with excellent stereocontrol.

With fragments **4** and **5** in hand, we turned our attention to their union through a stereocontrolled lithiation–borylation reaction.^20,31^ Before embarking on this key C–C bond forming reaction, we wanted to explore what level of substrate control might be imparted by the neighboring six-ring acetonide moiety in TIB ester **5**. Previously, Hoppe reported that the five-ring acetonide in carbamate **30** exerted strong control over stereoselectivity in the deprotonation with ^*s*^BuLi in the absence of diamine ligands (Scheme 3A).^32^ However, our attempts at a diamine-free lithiation–borylation using model acetonide-containing TIB ester **25** and boronic ester **32** were unsuccessful due to the failure of **25** to be deprotonated under these conditions (Scheme 3B). We attribute this contrasting reactivity to the weaker complexation of ^*s*^BuLi to the six-membered acetonide in **25** compared to the five-membered acetonide in **30**. Hoppe found that substrate control in the deprotonation of **30** could be largely overridden with diamine ligands, including TMEDA and sparteine. Fortunately, we found that addition of TMEDA enabled the coupling of **25** and **32** to proceed in excellent yield. Using TMEDA the diastereoselectivity was low, indicating no substrate control, but using (+)- or (−)-sparteine enabled either diastereoisomer of **33** to be obtained with high selectivity. Further studies by ReactIR revealed that deprotonation of **25** was exceptionally fast (*t*_1/2_ deprotonation ∼2 min at −78 °C) and that borylation was essentially instantaneous with primary boronic ester **32**.^33^ These conditions for the lithiation–borylation of model TIB ester **25** were successfully implemented in the coupling of the advanced fragments A and B (Scheme 4), and subsequent oxidation gave alcohol **2** in 68% yield with excellent stereocontrol at C23 (>20:1 dr). Although a significant excess of TIB ester **5** was required for optimum yield, unreacted **5** could easily be recovered by chromatographic purification. The lithiation–borylation between **5** and **4** not only provided the open-chain backbone of bastimolide B with all the correct functionality and stereochemistry in place, but boronic ester oxidation also revealed the alcohol required for closure of the 24-membered macrolactone without having to manipulate protecting groups. The final stages of the synthesis involved esterification of the alcohol at C23 under modified Yamaguchi conditions with acid (*Z*)-**3**.^34^ In the following one-pot protocol, the terminal olefin underwent hydroboration with 9-BBN followed by stereoretentive Suzuki cross-coupling with the (*Z*)-iodoalkene, giving the 24-membered macrolactone **35** in 42% yield.^35,36^ This macrocyclization approach demonstrated the power of intramolecular Suzuki cross-coupling both for macrolactone formation and for the installation of a *Z*-configured alkene in a particularly challenging setting.^37^ Finally, deprotection of the *O*-TES and acetonide groups in macrolide **35** occurred smoothly in methanol with DOWEX acidic resin, affording a pure sample of bastimolide B (**1**, 94% yield). The synthetic material matched the natural sample in all aspects (^1^H NMR, ^13^C NMR, HRMS, IR, optical rotation, and ECD), confirming the proposed structure, and its absolute stereochemistry. Using very high ^13^C resolution (<0.01 ppm) data from pure-shift HSQC and HSQC-TOCSY NMR methods, we were also able to establish the two- and three-bond connectivity of the molecule and fully assign every ^1^H and ^13^C NMR signal. It is worth noting that although the ^1^H and ^13^C NMR of our synthetic sample matched the data for the natural product, many of the assignments of the NMR signals did not (see the Supporting Information for details). Fortunately, these errors in the ^1^H/^13^C assignments did not detract from the 2D and 3D structure determination in the original report because their structure proposal was informed by comparison to X-ray analysis of the related natural product bastimolide A rather than relying exclusively on NMR.^21,22^

**Scheme 3:**
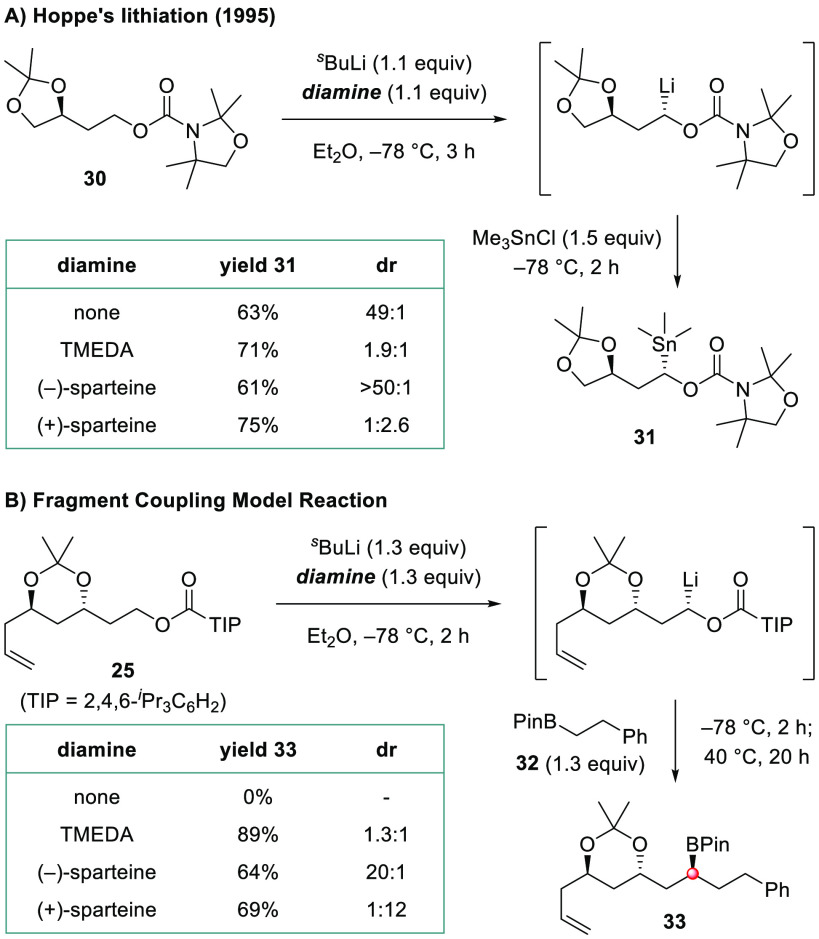
Study of Effects of Substrate Control on Asymmetric Lithiation

**Scheme 4:**
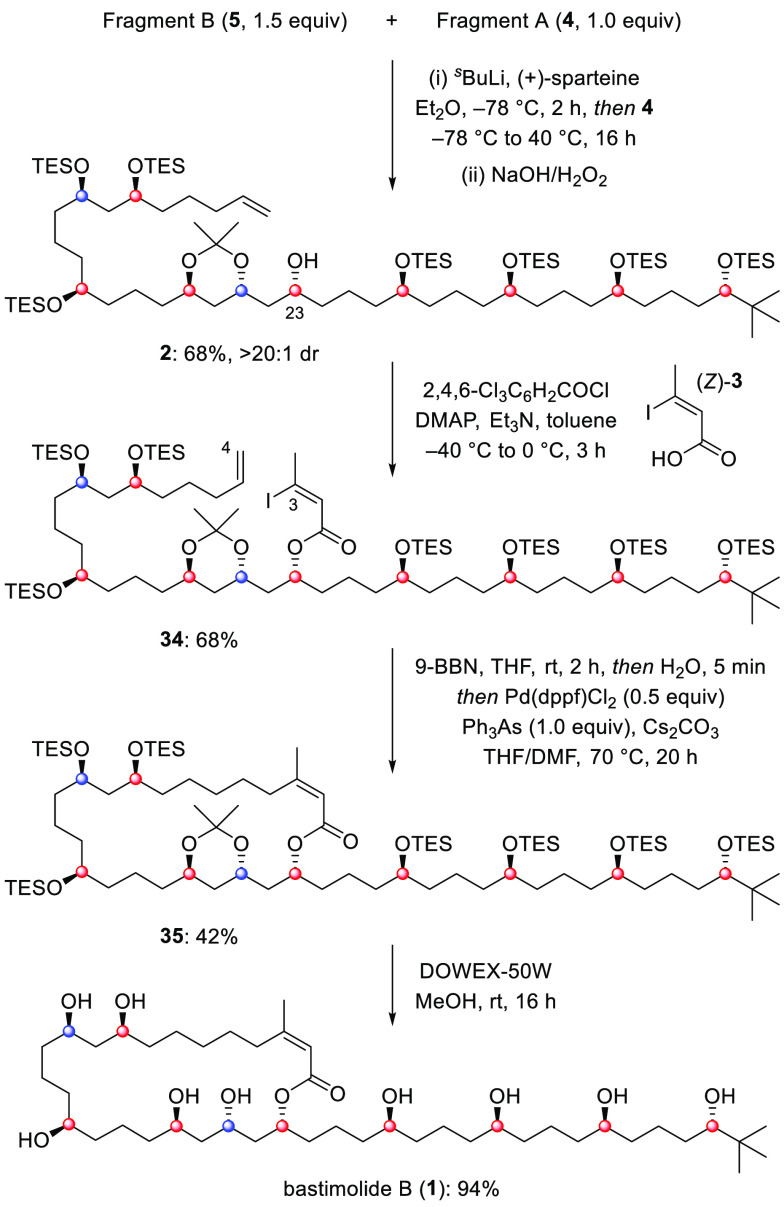
Fragment Coupling and Completion of the Synthesis of Bastimolide B

In conclusion, we have shown that homologation of boronic esters using a magnesiated butenyl TIB ester followed by hydroboration of the terminal alkene provides an iterative method for the generation of 1,5-polyols with full stereocontrol. This method has been applied to a 16-step (LLS) synthesis of bastimolide B (26 steps in total) with full stereocontrol. Reagent-controlled boronic ester homologations were used to create all the key C–C bonds, including a late-stage fragment coupling, as well as installing eight of the ten stereogenic centers, with the remaining two installed by asymmetric diboration. Four of the six 1,5-related stereocenters were generated by using the magnesiated-butenyl building block in homologations of poly(boronic esters) which showed essentially complete selectivity in favor of reaction at the primary boronic ester, even in the presence of multiple secondary boronic esters. This versatile methodology will undoubtedly find further applications in synthesis.

## Supporting Information Available

Experimental procedures and characterization data for new compounds (PDF)
