# Supplementary material for: Stereocontrolled Total Synthesis of Bastimolide B Using Iterative Homologation of Boronic Esters
Source: J Am Chem Soc. 2022 May 2;144(18):7995–8001. doi: 10.1021/jacs.2c03192 (PMC9100475; doi:10.1021/jacs.2c03192)
Supplement: Supplementary file 1 — ja2c03192_si_001.pdf [file ja2c03192_si_001.pdf]

Stereocontrolled Total Synthesis of Bastimolide B using Iterative Homologation of Boronic Esters

Daniele Fiorito, Selbi Keskin, Joseph M. Bateman, Malcolm George, Adam Noble and Varinder K. Aggarwal*

School of Chemistry, University of Bristol, Cantock's Close, Bristol BS8 1TS, U.K.

v.aggarwal@bristol.ac.uk

Table of Contents

1. General Methods and Reagents **5**

2. Preparation of Known Compounds **8**

2.1 (*S*)-1-((*S*)-*p*-tolylsulfinyl)but-3-en-1-yl 2,4,6-triisopropylbenzoate (**8**) 8

2.2 Compounds (*S*,*S*)-**L1**, **3**, **10**, **12**, **32** 11

3. Preparation and Characterization Data of New Compounds **12**

(*S*)-2,2-Dimethyl-1-(trimethylstannyl)propyl 2,4,6-triisopropylbenzoate (**13**) 12

(*S*)-2-(2,2-Dimethylhex-5-en-3-yl)-4,4,5,5-tetramethyl-1,3,2-dioxaborolane (**15**) 14

(*S*)-2,2'-(5,5-dimethylhexane-1,4-diyl)bis(4,4,5,5-tetramethyl-1,3,2-dioxaborolane) (**16**) 16

2,2'-((3*S*,7*S*)-2,2-Dimethyldec-9-ene-3,7-diyl)bis(4,4,5,5-tetramethyl-1,3,2-dioxaborolane) (**17**) 17

2,2',2''-((4*R*,8*S*)-9,9-Dimethyldecane-1,4,8-triyl)tris(4,4,5,5-tetramethyl-1,3,2-dioxaborolane) (**18**) 18

2,2',2''-((3*S*,7*R*,11*S*)-2,2-Dimethyltetradec-13-ene-3,7,11-triyl)tris(4,4,5,5-tetramethyl-1,3,2-dioxaborolane) (**19**) 19

2,2',2'',2'''-((4*R*,8*R*,12*S*)-13,13-dimethyltetradecane-1,4,8,12-tetrayl)tetrakis(4,4,5,5-tetramethyl-1,3,2-dioxaborolane) (**20**) 20

2,2',2'',2'''-((3*S*,7*R*,11*S*,15*S*)-2,2-dimethyloctadec-17-ene-3,7,11,15-tetrayl)tetrakis(4,4,5,5-tetramethyl-1,3,2-dioxaborolane) (**6**) 21

(3*S*,7*R*,11*S*,15*S*)-2,2-Dimethyloctadec-17-ene-3,7,11,15-tetraol (**21**) 23

(5*S*,9*S*,13*R*,17*S*)-5-Allyl-17-(*tert*-butyl)-3,3,19,19-tetraethyl-9,13-bis((triethylsilyl)oxy)-4,18-dioxa-3,19-disilahenicosane (**22**) 25

(5*S*,9*R*,13*R*,17*R*)-5-(*tert*-Butyl)-3,3,19,19-tetraethyl-17-(3-(4,4,5,5-tetramethyl-1,3,2-dioxaborolan-2-yl)propyl)-9,13-bis((triethylsilyl)oxy)-4,18-dioxa-3,19-disilahenicosane (**4**, **Fragment A**) 26

(*S*)-3,4-bis(4,4,5,5-Tetramethyl-1,3,2-dioxaborolan-2-yl)butyl 2,4,6-triisopropylbenzoate (**23**) 28

(*R*)-3,4-Dihydroxybutyl 2,4,6-triisopropylbenzoate (**23-ox**) 30

(3*R*,5*R*)-3,5-Dihydroxyoct-7-en-1-yl 2,4,6-triisopropylbenzoate (**24**) 32

2-((4*R*,6*R*)-6-Allyl-2,2-dimethyl-1,3-dioxan-4-yl)ethyl 2,4,6-triisopropylbenzoate (**25**) 34

2-((4*R*,6*R*)-2,2-Dimethyl-6-(3-(4,4,5,5-tetramethyl-1,3,2-dioxaborolan-2-yl)propyl)-1,3-dioxan-4-yl)ethyl 2,4,6-triisopropylbenzoate (**26**) 35

hex-5-en-1-yl 2,4,6-triisopropylbenzoate (**11**) 36
(*R*)-1-((*R*)-*p*-tolylsulfinyl)hex-5-en-1-yl 2,4,6-triisopropylbenzoate (**9**) 37
2-((4*R*,6*R*)-2,2-dimethyl-6-((*S*)-4-(4,4,5,5-tetramethyl-1,3,2-dioxaborolan-2-yl)non-8-en-1-yl)-1,3-dioxan-4-yl)ethyl 2,4,6-triisopropylbenzoate (**27**) 41
2-((4*R*,6*R*)-2,2-Dimethyl-6-((4*S*,8*S*)-4,8,9-tris(4,4,5,5-tetramethyl-1,3,2-dioxaborolan-2-yl)nonyl)-1,3-dioxan-4-yl)ethyl 2,4,6-triisopropylbenzoate (**28**) 43
2-((4*R*,6*R*)-2,2-Dimethyl-6-((4*S*,8*R*,10*S*)-4,8,10-tris(4,4,5,5-tetramethyl-1,3,2-dioxaborolan-2-yl)pentadec-14-en-1-yl)-1,3-dioxan-4-yl)ethyl 2,4,6-triisopropylbenzoate (**7**) 45
2-((4*R*,6*R*)-2,2-Dimethyl-6-((4*S*,8*R*,10*S*)-4,8,10-trihydroxypentadec-14-en-1-yl)-1,3-dioxan-4-yl)ethyl 2,4,6-triisopropylbenzoate (**29**) 47
2-((4*R*,6*R*)-2,2-Dimethyl-6-((4*R*,8*R*,10*S*)-4,8,10-tris((triethylsilyl)oxy)pentadec-14-en-1-yl)-1,3-dioxan-4-yl)ethyl 2,4,6-triisopropylbenzoate (**5**, **Fragment B**) 49
(2*R*,6*R*,10*R*,14*R*,18*S*)-1-((4*R*,6*R*)-2,2-Dimethyl-6-((4*R*,8*R*,10*S*)-4,8,10-tris((triethylsilyl)oxy)pentadec-14-en-1-yl)-1,3-dioxan-4-yl)-19,19-dimethyl-6,10,14,18-tetrakis((triethylsilyl)oxy)icosan-2-ol (**2**) 51
(2*R*,6*R*,10*R*,14*R*,18*S*)-1-((4*S*,6*R*)-2,2-Dimethyl-6-((4*R*,8*R*,10*S*)-4,8,10-tris((triethylsilyl)oxy)pentadec-14-en-1-yl)-1,3-dioxan-4-yl)-19,19-dimethyl-6,10,14,18-tetrakis((triethylsilyl)oxy)icosan-2-yl (*Z*)-3-iodobut-2-enoate (**34**) 53
Protected bastimolide B (**35**) 55
4. Preparation and Characterization Data of Bastimolide B (1) 57
4.1 Bastimolide B Synthesis 57
4.2 Bastimolide B Characterization 58
4.3. Bastimolide B Full Chemical Shift Assignment (Py-*d*5) 62
5. Optimization of Fragment Coupling on Model Compounds 65
2-((*R*)-1-((4*R*,6*R*)-6-Allyl-2,2-dimethyl-1,3-dioxan-4-yl)-4-phenylbutan-2-yl)-4,4,5,5-tetramethyl-1,3,2-dioxaborolane (**33**) 68
6. NMR Spectra of New Compounds 69
^{1}H NMR (400 MHz, $CDCl_3$) of compound **13** 70
^{13}C NMR (101 MHz, $CDCl_3$) of compound **13** 70
^{1}H NMR (400 MHz, $CDCl_3$) of compound **15** 71
^{1}H NMR (400 MHz, $CDCl_3$) of compound **16** 72
^{13}C NMR (101 MHz, $CDCl_3$) of compound **16** 72
^{13}C NMR (101 MHz, $CDCl_3$) of compound **17** 73
^{1}H NMR (400 MHz, $CDCl_3$) of compound **18** 75
^{13}C NMR (101 MHz, $CDCl_3$) of compound **18** 75
^{1}H NMR (400 MHz, $CDCl_3$) of compound **19** 76
^{13}C NMR (101 MHz, $CDCl_3$) of compound **19** 76
^{1}H NMR (400 MHz, $CDCl_3$) of compound **20** 77

^{13}C NMR (101 MHz, $CDCl_3$) of compound **20**........77
^{1}H NMR (400 MHz, $CDCl_3$) of compound **6**........78
^{13}C NMR (101 MHz, $CDCl_3$) of compound **6**........78
^{1}H NMR (500 MHz, CD_3OD) of compound **21**........79
^{13}C NMR (126 MHz, CD_3OD) of compound **21**........79
^{1}H NMR (500 MHz, Py-*d*5) of compound **21**........81
^{13}C NMR (126 MHz, Py-d5) and HSQC (500 MHz (^{1}H), 126 MHz (^{13}C), py-*d*5) of compound **21** 83
^{1}H NMR (500 MHz, $CDCl_3$) of compound **22**........85
^{13}C NMR (126 MHz, $CDCl_3$) of compound **22**........85
^{1}H NMR (500 MHz, $CDCl_3$) of compound **4**........86
^{13}C NMR (126 MHz, $CDCl_3$) of compound **4**........87
^{1}H NMR (400 MHz, $CDCl_3$) of compound **23**........88
^{13}C NMR (101 MHz, $CDCl_3$) of compound **23**........88
^{1}H NMR (400 MHz, $CDCl_3$) of compound **23-ox**........89
^{13}C NMR (101 MHz, $CDCl_3$) of compound **23-ox**.........89
^{1}H NMR (400 MHz, $CDCl_3$) of compound **24**........90
^{13}C NMR (101 MHz, $CDCl_3$) of compound **24**........90
^{1}H NMR (400 MHz, $CDCl_3$) of compound **25**........91
^{13}C NMR (101 MHz, $CDCl_3$) of compound **25**........91
^{1}H NMR (400 MHz, CD_2Cl_2) of compound **26**........92
^{13}C NMR (101 MHz, CD_2Cl_2) of compound **26**........92
^{1}H NMR (400 MHz, $CDCl_3$) of compound **11**........93
^{13}C NMR (101 MHz, $CDCl_3$) of compound **11**........93
^{1}H NMR (400 MHz, $CDCl_3$) of compound **9**........94
^{13}C NMR (101 MHz, $CDCl_3$) of compound **9**........94
^{1}H NMR (400 MHz, CD_2Cl_2) of compound **27**........95
^{13}C NMR (126 MHz, CD_2Cl_2) of compound **27**........95
^{1}H NMR (400 MHz, CD_2Cl_2) of compound **28**........96
^{13}C NMR (126 MHz, CD_2Cl_2) of compound **28**........96
^{1}H NMR (500 MHz, $CDCl_3$) of compound **7**........97
^{13}C NMR (126 MHz, $CDCl_3$) of compound **7**........97
^{1}H NMR (500 MHz, $CDCl_3$) of compound **29**........98
^{13}C NMR (126 MHz, $CDCl_3$) of compound **29**........98
^{1}H NMR (500 MHz, $CDCl_3$) of compound **5**........100
^{13}C NMR (126 MHz, $CDCl_3$) of compound **5**........101
^{1}H NMR (500 MHz, $CDCl_3$) of compound **2**........103

^{13}C NMR (126 MHz, $CDCl_3$) of compound **2**. 104
^{1}H NMR (400 MHz, CD_2Cl_2) of compound **33** 106
^{13}C NMR (101 MHz, CD_2Cl_2) of compound **33** 106
^{1}H NMR (500 MHz, $CDCl_3$) of compound **34**. 107
^{13}C NMR (126 MHz, $CDCl_3$) of compound **34**. 108
^{1}H NMR (500 MHz, $CDCl_3$) of compound **35**. 110
^{13}C NMR (126 MHz, $CDCl_3$) of compound **35** 111
7. NMR spectra of Bastimolide B (1) 113
^{1}H NMR (500 MHz, CD_3COD) of synthetic bastimolide B (**1**) 113
^{1}H NMR (500 MHz, py-*d*5) of synthetic bastimolide B (**1**) 115
^{13}C NMR (126 MHz, CD_3OD) of synthetic bastimolide B (**1**). 117
^{13}C NMR (126 MHz, py-*d*5) of synthetic bastimolide B (**1**) 119
^{1}H NMR (py-*d*5) comparison of natural and synthetic bastimolide B (**1**) 121
^{13}C NMR (py-*d*5) comparison of natural and synthetic bastimolide B (**1**) 122
NMR Spectra for the Structural Assignment of Bastimolide B (**1**) 125
8. References 129

1. General Methods, Material and Reagents

Glassware and Reaction Setup: unless otherwise stated, all reactions were conducted under an inert atmosphere of nitrogen in flame dried glassware using standard Schlenk techniques. Air- and moisture-sensitive liquids and solutions were transferred via syringe into the reaction vessels through a rubber septum. Temperatures described below −10 °C were achieved using Thermo Scientific EK-90 or Huber TC100E cryostats or appropriate solvent/dry ice baths.

Solvents: Non-anhydrous solvents were purchased (unless specified) at the highest commercial quality and used as received. CH_2Cl_2, Et_2O and THF were dried on an Anhydrous Engineering alumina column drying system.

Reagents: Pt(dba)$_3$ [CAS 11072-92-7] was purchased from Strem Chemicals, Inc. and used as received. [Ir(1,5-cod)Cl]$_2$ [CAS12112-67-3] was purchased from Sigma Aldrich and used as received. (+)-Andersen's sulfinate [CAS 91796-57-5] and (-)-Andersen's sulfinate [CAS 1517-82-4] were purchased from Sigma Aldrich and used as received. ^{n}Butyl lithium [CAS 109-72-8] was purchased from Acros as a 1.6 M solution in n-hexane. ^{s}Butyl lithium [CAS 598-30-1] was purchased from Acros Organics as a 1.3 M solution in cyclohexane:^{n}hexane 98:2. The molarity of organolithium solutions was regularly determined by titration with *N*-benzylbenzamide.[1] ^{i}PrMgCl·LiCl was purchased from Sigma Aldrich as a 1.2 M solution in THF and the molarity was verified by titration with iodine.[2] TMEDA and Et_3N were distilled over CaH_2 before use. Pinacolborane (HBPin), triethylsilyl trifluoromethanesulfonate (TESOTf) and 2,4,6-trichlorobenzoyl chloride were distilled before use. (–)-Sparteine was isolated from the commercially available sulfate pentahydrate salt following a procedure by Beak.[3] (+)-Sparteine was purchased as the free base and distilled over CaH_2. The sparteine free base readily absorbs atmospheric carbon dioxide (CO_2) and so should be stored under argon/nitrogen at −20 °C in a sealed Schlenk tube. Sparteine can be recovered reliably during work-up with aqueous HCl as reported in the literature.[4] All other reagents were purchased from various commercial sources at highest commercial quality and used.

Chromatography: flash column chromatography was carried out using Sigma-Aldrich silica gel (60 Å, 230-400 mesh, 40-63 μm) or a Biotage Isolera One automated flash purification system, as indicated. Reactions were followed by thin-layer chromatography (TLC) where practical, using aluminium-backed Merck Kieselgel 60 F254 fluorescent treated silica gel plates, which were visualised under UV light or by staining with aqueous basic KMnO4, acidic *p*-anisaldehyde solution in ethanol, or phosphomolybdic acid solution in ethanol.

NMR spectra were recorded on Jeol ECS (400 MHz), Jeol ECZ (400 MHz), Bruker Avance (400 MHz) or Bruker Cryo (500 MHz) instruments. Chemical shifts (δ) are quoted in parts per million (ppm) and referenced to the appropriate NMR solvent peak(s) and are assigned in accordance with numbered

diagrams; with resonances described as s (singlets), d (doublets), t (triplets), q (quartets), p (pentets), combinations thereof (i.e. td indicates a triplet of doublets) or m (multiplets) and br s (broad singlet). Numbering of C-atoms for NMR assignment follows the numbering given to Bastimolide B in the original isolation paper.[5]

NMR yields: following work up, 1,3,5-trimethoxybenzene (1.0 equiv relative to limiting SM) was added to the crude residue. The resultant mixture was dissolved in $CDCl_3$ (1.0 mL), and a 0.5 mL sample of the resultant solution taken for ^1H NMR analysis. Yields were calculated based on the integrals of known product resonances relative to 1,3,5-trimethoxybenzene.

HRMS (high resolution mass spectra) were recorded on a Bruker Daltonics MicrOTOF II by Electrospray Ionisation (ESI); a Thermo Scientific QExactive by Electron Ionisation (EI); a Thermo Scientific Orbitrap Elite by ESI or Atmospheric Pressure Chemical Ionisation (APCI); or a Bruker UltrafleXtreme by Matrix-assisted Laser Desorption/Ionisation (MALDI). Only molecular ions ($[M+H]^+$, $[M+NH_4]^+$ or $[M+Na]^+$) are reported.

LCMS: Reverse-phase LCMS traces were obtained on Agilent 1260 Infinity II system with Agilent PoroShell 120 EC-C18 column (3.0×50 mm, 2.7 µm) using acetonitrile-water gradients (50→90% or 10→90% MeCN-H_2O) and observing at 254 or 214 nm.

HPLC: enantiomeric ratio was determined by HPLC analysis on chiral stationary phase performed using Daicel Chiralpak IA/IB columns (4.6 mm×250 mm×5 µm)/OD column (4.6 mm×250 mm×10 µm)/AD-H (4.6 mm×250 mm×5 µm) column on an Agilent system and monitored using a diode array detector (DAD).

IR spectra were recorded on neat compounds using a Perkin Elmer (Spectrum One) FT-IR spectrometer (ATR sampling accessory). Selected absorbances (ν_{max}, expressed in cm^{-1}) are reported.

In Situ (IR) spectroscopy (React-IR): the reactions were monitored using Mettler Toledo React-IR 15 mid-infrared spectrometer equipped with a Silver Halide (AgX) FiberConduit with integrated DiComp probe, using the iC IR Reaction Analysis software (version 4.3). Prior to the use of the React-IR machine for data collection, the contrast and align, performance (5 runs) and stability (duration 5 minutes) were tested and saved.

Melting points (m.p.) were recorded in degrees Celsius (°C) using a Stuart SMP30 melting point apparatus.

Optical rotations ($[\alpha]_D^{25}$) were measured on a Bellingham & Stanley Ltd. ADP 220 polarimeter.

ECD were recorded on a JASCO J-815 CD spectrometer.

Naming of compounds: Compound names are generated by ChemDraw Professional 20.0 software (PerkinElmer), following the IUPAC nomenclature.

2. Preparation of Known Compounds

2.1 (*S*)-1-((*S*)-*p*-tolylsulfinyl)but-3-en-1-yl 2,4,6-triisopropylbenzoate (8)

(*S*)-1-((*S*)-*p*-tolylsulfinyl)but-3-en-1-yl 2,4,6-triisopropylbenzoate **8** was prepared from benzoate **10** (10.0 g, 33.0 mmol) following the protocol described in the literature using TMEDA/^{s}BuLi.[6] This protocol gives a 1:1 mixture of diastereoisomers, which are separable using BIOTAGE purification system (Biotage HC-200g, ^{n}Hexane: EtOAc 1-20%). Details for BIOTAGE purification are given below. The purification gives *syn*-(*S*,*S*)-**8** (5.53 g, 38% yield, >20:1 dr, ≥99:1 er) as a crystalline white solid, and the *anti*-diastereoisomer (*R*,*S*)-**8** (4.65 g, 32% yield, >20:1 dr, ≥99:1 er) as a dense colourless oil.

Alternatively, the desired diastereoisomer *syn*-(*S*,*S*)-**8** can be obtained selectively employing (-)-sparteine/^{s}BuLi. This modified protocol is detailed below, along with the BIOTAGE purification protocol.

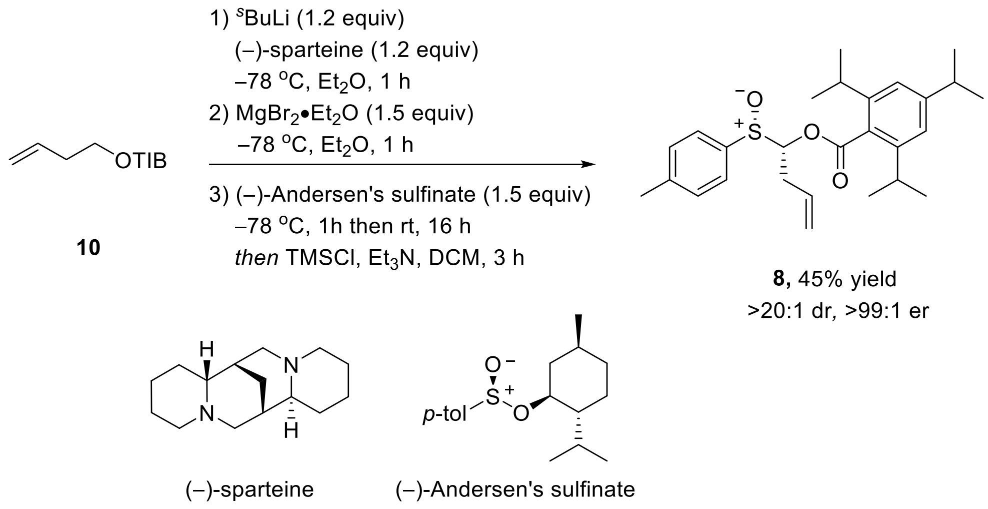

(-)-sparteine (5.00 g, 21.32 mmol, 1.20 equiv) was added to a flame dried 3-necked 250 mL flask under nitrogen, followed by the TIB ester **10** (5.38 g, 17.77 mmol). The mixture was degassed by stirring under vacuum for 30 min, then dry diethyl ether (59.00 mL) was added and the reaction mixture was cooled to –78 °C. To the reaction mixture, ^{s}BuLi (1.30 M hexane, 16.40 mL, 21.32 mmol, 1.20 equiv) was added with a syringe pump (1.00 mL/min). The reaction mixture was stirred 1 h at –78 °C (*purple solution*). In a separate flame dried 100 mL 2-necked flask under nitrogen with a condenser, to a stirred suspension of Mg turnings (1.73 g, 71.08 mmol, 4.00 equiv) in Et_2O (33.00 mL), dibromoethane (5.00 g, 26.66 mmol, 1.50 equiv) was added dropwise (*CAUTION: exothermic reaction*). After reflux and gas evolution ceased, the $MgBr_2$ etherate solution was stirred 30 min at room temperature (*formed 2 layers, top colourless, bottom grey*), then it was transferred to the main reaction vessel with the aid of an addition funnel. The reaction mixture was stirred at –78 °C for 2 h (*orange solution with some solid agglomerate*). To this reaction mixture, (-)-(*S*)-Anderson's sulfinate (7.85 g, 26.66 mmol, 1.50 equiv)

dissolved in THF (27.00 mL) was added dropwise with a syringe pump (0.50 mL/min) and the reaction mixture was stirred 1 h at –78 °C (*orange solution turned yellow*), then 16 h at room temperature. The reaction mixture was quenched with 2 M HCl (100 mL), the layers separated, and the organics washed with 2 M HCl (4 x 50 mL). The combined aqueous phase was washed with Et_2O (3 x 100 mL), then the combined organics were washed with saturated $NaHCO_3$ (150 mL) and brine (150 mL), dried over anhydrous Na_2SO_4, filtered and concentrated under reduced pressure.

(–)-*sparteine was recovered from the acid aqueous phase (90% recovery) by basification, extraction with EtOAc, solvent removal and distillation under vacuum.*

To facilitate the chromatographic separation of sulfoxide **8** from menthol, silylation of menthol was performed as follows. The crude mixture was stirred 2 h under vacuum, then dissolved in CH_2Cl_2 (36.00 mL). Triethyl amine (3.70 mL, 26.70 mmol, 1.50 equiv) was added followed by the dropwise addition of trimethylsilyl chloride TMSCl (2.90 mL, 23.10 mmol, 1.30 equiv) and the mixture was stirred 3 h at room temperature. The reaction mixture was then diluted with Et_2O (70 mL) and water (100 mL), the layers separated and the organics were dried one dried over anhydrous Na_2SO_4, filtered and concentrated under reduced pressure. The crude residue was purified by automated flash column chromatography (Biotage HC-200g, ^{n}Hexane: EtOAc 1-20%, see report below) to afford the title compound major *syn*-diastereoisomer (*S*,*S*)-**8** (3.50 g, 45% yield, >20:1 dr, ≥99:1 er) as a crystalline white solid, and the minor *anti*-diastereoisomer (*R*,*S*)-**8** (0.33 g, 4% yield, >20:1 dr) as a dense colourless oil. All spectroscopic data matched those previously reported.

Enantiomeric ratio ≥99:1 for the major *syn*-diastereoisomer was determined using HPLC analysis with chiral stationary phase, Daicel Chiralcel-IA column, ^{n}Hex: ^{i}PrOH = 95:5, 0.5 mL/min, λ = 210 nm.
t_R = 12.8 [(*S*,*R*), minor], 14.0 [(*S*,*S*), major].

TLC: R_f = 0.4 for major *syn*-diastereoisomer, 0.3 for minor *anti*-diastereoisomer (pentane/ EtOAc 10%, stained with PMA).

Biotage Isolera™ Archive Report - df1-97bis 1.

**User**	Daniele		
**Sample Name**	df1-97bis		
**Date**	2021-Mar-08 12.26		
**Method**	df1-29		
**Cartridge**	Sfar Silica HC D 200g	**Detection Mode**	Lambda-all
**Flowrate**	200 ml/min	**Baseline Correction**	On
**Solvent A**	hexane	**UV1 (Monitor)**	254 nm (Red)
**Solvent B**	Ethyl acetate	**UV2 (Monitor)**	280 nm (Black)
		**Lambda-all (Collect)**	(Brown)
**Rack Type**	16x150 mm	**Start Threshold**	40 mAU
**Max Fraction Volume**	22 ml		
**Dispense Order**	S		

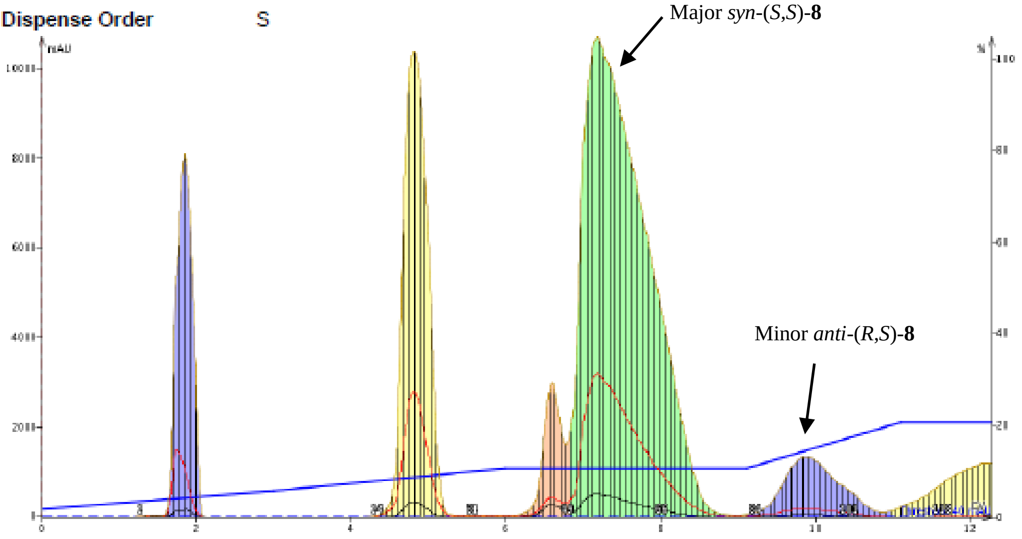

Gradient

	Solvents	Mix	Length (CV)	
Equil.	A/B	1%	3.0	flowrate 200 ml/min
1	A/B	1% - 5%	3.0	
2	A/B	5% - 10%	3.0	
3	A/B	10%	1.8	
4	A/B	10%	1.2	Edit on the fly
5	A/B	10% - 20%	2.0	Edit on the fly
6	A/B	20%	1.1	Edit on the fly

2.2 Compounds (*S*,*S*)-L1, 3, 10, 12, 32

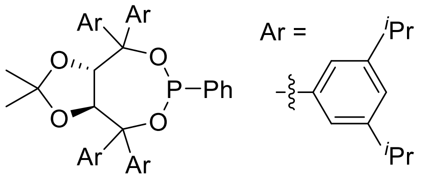

(*S*,*S*)-**L1**

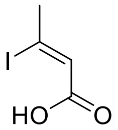

(*Z*)-**3**

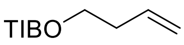

10

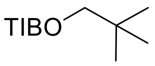

12

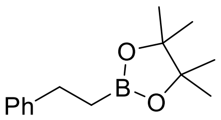

32

Phosphonite (*S*,*S*)-**L1** was prepared following a protocol described in the literature.[7]

Acid **3** was prepared from commercially available 3-butynoic acid following a protocol described in the literature.[8]

Benzoate **10** was prepared from commercially available but-3-en-1-ol following a protocol described in the literature.[6]

Benzoate **12** was prepared from commercially available 2,2-dimethylpropan-1-ol following a protocol described in the literature.[6]

Boronic ester **32** was prepared from commercially available phenethylboronic acid following a protocol described in the literature.[9]

3. Preparation and Characterization Data of New Compounds

(*S*)-2,2-Dimethyl-1-(trimethylstannyl)propyl 2,4,6-triisopropylbenzoate (13)

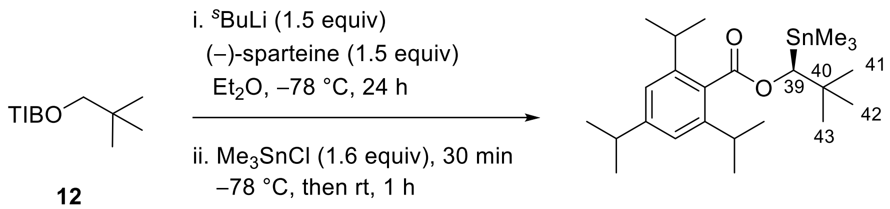

12

13
70% yield, 98:2 er

To a solution of TIB ester **12** (5.10 g, 16.00 mmol, 1.00 equiv) and (–)-sparteine (5.50 mL, 24.00 mmol, 1.5 equiv) in diethyl ether (52.00 mL, 0.30 M), ^{s}BuLi (1.30 M solution in heptane, 18.50 mL, 24.00 mmol, 1.50 equiv) was added dropwise at –78 °C and the reaction mixture was stirred at same temperature for 24 h. Trimethyl tin chloride (1.00 M in hexane, 26.00 mL, 1.60 equiv) was added dropwise (1.00 mL/min) to the reaction mixture at –78 °C, the reaction mixture was stirred for 0.5 h at –78 °C and then it was warmed up to room temperature and stirred 1 h. The reaction was quenched by the addition of 2 M HCl aq (50 mL) and extracted with EtOAc (3x100 mL). The organics were concentrated under reduced pressure and the crude residue was purified by automated flash column chromatography (Biotage HC-100g, pentane: EtOAc 5%) to afford the title compound (*S*)-**13** (5.39 g, 70% yield, 98:2 er) as a colourless oil.

(–)-sparteine was recovered from the acid aqueous phase (90% recovery) by basification, extraction with EtOAc, solvent removal and distillation under vacuum.

TLC: R_f = 0.26 (95:5 pentane/EtOAc, stained with *p*-anisaldehyde)

^{1}H NMR (400 MHz, Chloroform-*d*) δ 7.00 (s, 2H, *H*-Ar), 5.03 (s and d, ^1J ^{1}H-^{119}Sn = 14.5 Hz, 1H, *H*-39), 2.98 – 2.82 (m, 3H, *H*-*i*Pr), 1.25 (d, *J* = 6.9 Hz, 6H, CH_3-*i*Pr), 1.24 (d, *J* = 6.9 Hz, 6H, CH_3-*i*Pr), 1.22 (d, *J* = 6.9 Hz, 6H, CH_3-*i*Pr), 1.03 (s, 9H, *H*-41, 42, 43), 0.25 (s, and d, ^2J ^{1}H-^{119}Sn = 54.1 Hz and d, ^2J ^{1}H-^{117}Sn = 51.8 Hz, 9H, CH_3-Sn). See spectrum.

^{13}C NMR (101 MHz, Chloroform-*d*) δ 171.13 (*C*O-benzoate), 149.93 (*C*-*ipso*), 145.33 (*C*-*ortho*), 130.88 (*C*-*para*), 121.08 (*C*H-Ar), 84.16 (*C*H-39), 36.45 (*C*-40), 34.47 (*C*H-*i*Pr *para*), 31.59 (*C*H-*i*Pr *ortho*), 28.34 (*C*H$_3$-41, 42, 43), 24.68 (*C*H$_3$-*i*Pr), 24.53 (*C*H$_3$-*i*Pr), 24.07 (*C*H$_3$-*i*Pr), -6.44 (s and d, *J* ^{13}C-^{119}Sn = 325.2 Hz and d, *J* ^{13}C-^{117}Sn = 311.1 Hz, *C*H$_3$-Sn). See spectrum.

IR (neat) ν_{max} (cm^{-1}): 2960, 2929, 2869, 1708 (C=O), 1607, 1461, 1363, 1282, 1251, 1188, 1136, 1103, 1074, 877, 767, 713, 525.

HRMS (MALDI) calculated for $C_{24}H_{42}O_{12}SnNa$ $[M+Na]^+$: 505.2103, found: 505.2109.

$[\alpha]_D^{25}$ = +28.0 (c 1.0, CH_2Cl_2)

HPLC on chiral stationary phase Daicel Chiralpak-IB column (25 cm), 100% ^{n}Hexane, flow rate: 0.9 mL/min., rt, 210 nm, Retention times: 4.55 min. (*S*, major) and 5.42 min. (*R*, minor).

*Racemic **13** was prepared by replacing (–)-sparteine for N,N,N',N'-tetramethylethylendiamine (TMEDA) under otherwise identical reaction conditions.*

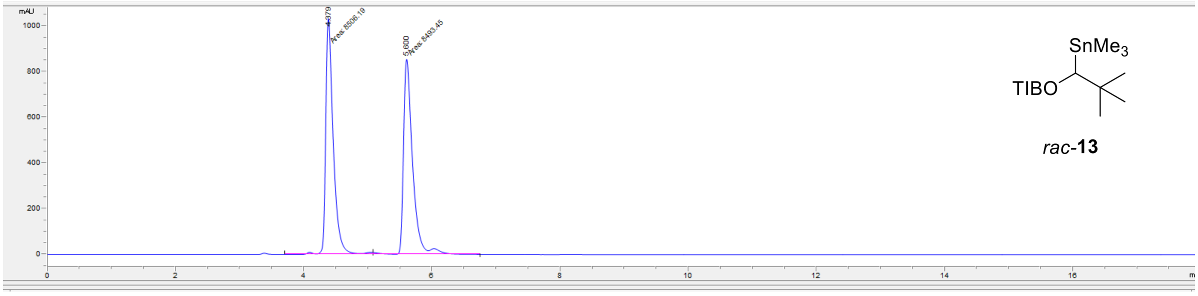

#	Time	Type	Area	Height	Width	Area%	Symmetry
1	4.379	MF	8506.2	1032.3	0.1373	50.037	0.53
2	5.6	FM	8493.4	852.8	0.166	49.963	0.531

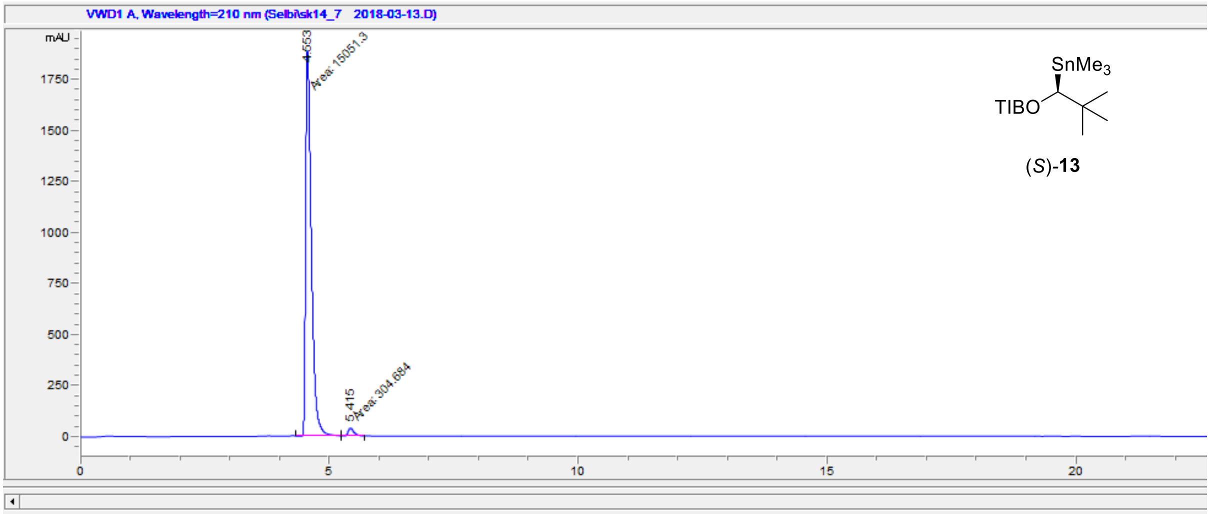

File Information	
LC-File	sk14_7 2018-03-13.D
File Path	C:\Chem32\1\Data\Selbi
Date	13-Mar-18, 10:14:55
Sample	sk14
Sample Info	IB, 100 hexane, flow rate 0.9 mL/min, RT, 210 nm
Barcode	
Operator	SYSTEM
Method	VKA_general.M

#	Time	Type	Area	Height	Width	Area%	Symmetry
1	4.553	MF	15051.3	1893.4	0.1325	98.016	0.44
2	5.415	FM	304.7	39.1	0.13	1.984	0.625

(*S*)-2-(2,2-Dimethylhex-5-en-3-yl)-4,4,5,5-tetramethyl-1,3,2-dioxaborolane (15)

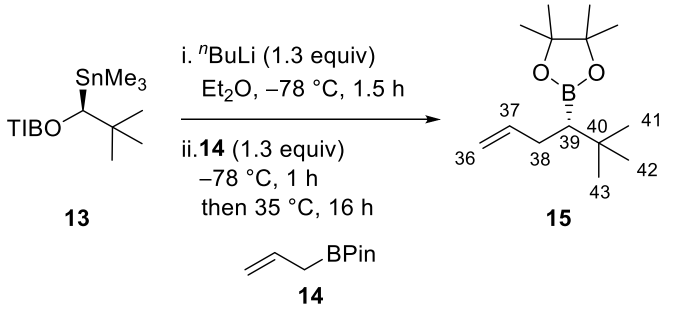

^{n}BuLi (1.60 M in hexane, 8.10 mL, 13.00 mmol, 1.30 equiv) was added dropwise to a solution of stannane (*S*)-**13** (4.80 g, 10.00 mmol, 1.00 equiv) in diethyl ether (0.30 M, 33.00 mL) at –78 °C and the reaction mixture was stirred 1.5 h at the same temperature. A solution of commercially available allyl boronic acid pinacol ester **14** (2.40 mL, 13.00 mmol, 1.30 equiv) in diethyl ether (0.30 M, 43.00 mL) was added dropwise (1.00 mL/min) to the reaction mixture at –78 °C and the resulting mixture was stirred 1 h at –78 °C, then it was warmed up to room temperature and heated 16 h at 35 °C. Diethyl ether (40 mL) and water (30 mL) were added to the mixture. The organic layer was washed with water (3x50 mL) and dried over $MgSO_4$. The solvent was evaporated, and the crude mixture was directly used for the next step.

*Boronic ester **15** is a volatile colourless oil and it is engaged directly in the following step as crude. Spectroscopic information for its identification is given below.*

TLC: R_f = 0.6 (96:4 pentane/Et_2O, stained with *p*-anisaldehyde)

^{1}H NMR (400 MHz, Chloroform-*d*) δ 5.78 (dddd, *J* = 17.2, 10.2, 7.7, 5.9 Hz, 1H, *H*-37), 5.10 – 4.95 (ddd, *J* = 17.2, 3.3, 1.7 Hz, 1H, *H*-36), 4.89 (ddd, *J* = 10.2, 2.2, 1.1 Hz, 1H, *H*-36), 2.28 – 2.04 (m, 2H, *H*-38), 1.22 (s, 12H, CH_3-BPin), 0.94 (s, 12H, *H*-41, 42, 43), 0.91 (d, *J* = 4.3 Hz, 1H, *H*-39). *See spectrum.*

^{13}C NMR (101 MHz, Chloroform-*d*) δ 139.54 (*C*H-37), 114.63 (*C*H_2-36), 83.03 (*C*O-BPin), 32.10 (*C*-40), 31.64 (*C*H_2-38), 29.63 (*C*H_3-41, 42, 43), 25.20 (*C*H_3-BPin), 25.13 (*C*H_3-BPin). Boron-bound *C*H-39 is not detected due to quadrupolar relaxation. *See spectrum.*

IR (neat) ν_{max} (cm^{-1}): 2958, 2933, 2867, 1745, 1455, 1369, 1359, 1316, 1241, 1141, 1108, 975, 908, 864, 809.

$[\alpha]_D^{25}$ = –21.7 (c 0.93, CH_2Cl_2)

The enantiomeric ratio of **15** was determined by oxidation of the boronic ester with NaOH/H_2O_2 and Mosher ester analysis of the resulting alcohol 2,2-dimethylhex-5-en-3-ol **15-ox** as described in the literature.[10] Esterification with (*R*)-Mosher acid was carried out as described in the literature.[11] The resulting Mosher ester was analyzed by ^{19}F NMR (98:2 er).

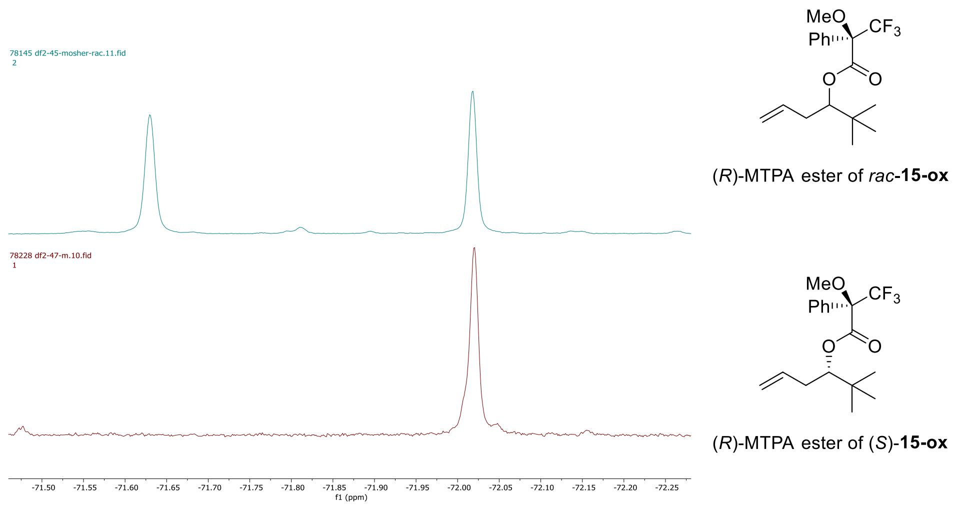

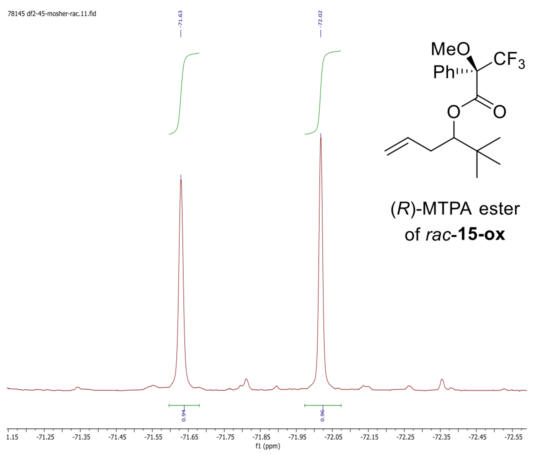

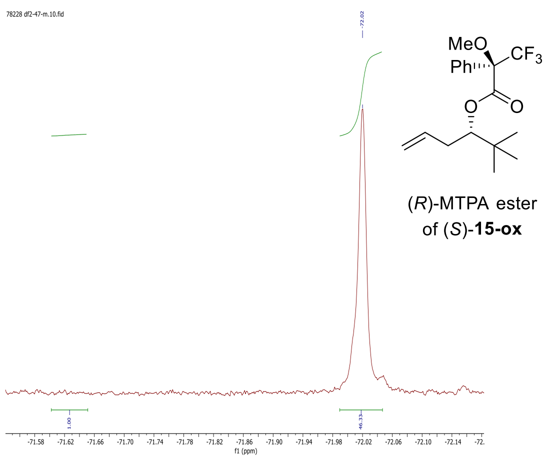

(*S*)-2,2'-(5,5-dimethylhexane-1,4-diyl)bis(4,4,5,5-tetramethyl-1,3,2-dioxaborolane) (16)

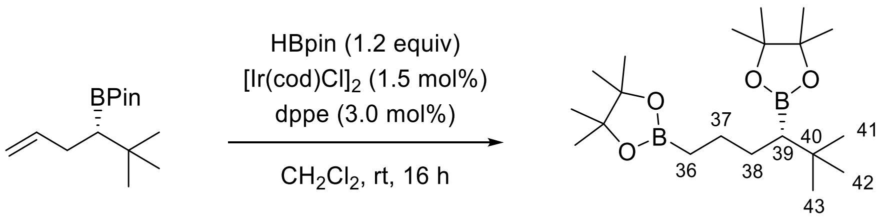

15

16, 72% yield over 2 steps from **13**

Pinacolborane (1.73 mL, 12.00 mmol, 1.20 equiv) and crude alkene **15** (10.00 mmol, 1.00 equiv) were added successively to a solution of $[Ir(cod)Cl]_2$ (0.10 g, 0.15 mmol, 0.015 equiv) and 1,2-bis(diphenylphosphino)ethane (dppe) (0.12 g, 0.30 mmol, 0.03 equiv) in CH_2Cl_2 (0.30 M, 33.00 mL) at room temperature. The reaction mixture was then stirred 16 h at room temperature. The reaction was quenched with methanol (15 mL) and extracted with diethyl ether (3x50 mL). The organics were dried over $MgSO_4$ and evaporated under reduced pressure. The crude residue was purified by column chromatography (4% Et_2O in pentane) to afford the title compound **16** (2.60 g, 72% yield over two steps) as a colourless oil.

TLC: R_f = 0.3 (96:4 pentane/Et_2O, stained with *p*-anisaldehyde)

^{1}H NMR (400 MHz, Chloroform-*d*) δ 1.48 – 1.34 (m, 3H, *H*-37 + *H*-38), 1.32 – 1.27 (m, 1H, *H*-37 *or* *H*-38), 1.24 (s, 12H, CH_3-BPin), 1.23 (s, 12H, CH_3-BPin), 0.91 (s, 9H, s, 12H, *H*-41, 42, 43), 0.83 – 0.74 (m, 3H, *H*-36 + *H*-39). See spectrum.

^{13}C NMR (101 MHz, Chloroform-*d*) δ 82.90 (*C*O-BPin), 82.84 (*C*O-BPin), 32.15 (*C*-40), 29.94 (*C*H_2-38), 29.69 (*C*H_3-41, 42, 43), 25.17 (*C*H_3-BPin), 25.08 (*C*H_3-BPin), 24.99 (*C*H_2-37), 24.96 (*C*H_3-BPin). Boron-bound *C*H_2-36 and *C*H-39 are not detected due to quadrupolar relaxation. See spectrum.

IR (neat) ν_{max} (cm^{-1}): 2977, 2932, 1370, 1312, 1272, 1242, 1214, 1110, 968, 867, 848.

HRMS (ESI) calculated for $C_{20}H_{41}B_2O_4$ $[M+H]^+$: 367.3185, found: 367.3184.

$[\alpha]_D^{25}$ = –8.0 (c 0.93, CH_2Cl_2)

2,2'-((3*S*,7*S*)-2,2-Dimethyldec-9-ene-3,7-diyl)bis(4,4,5,5-tetramethyl-1,3,2-dioxaborolane) (17)

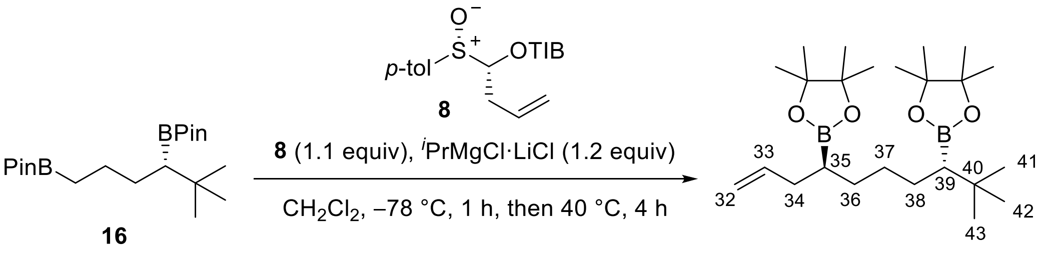

17, 68% yield, > 20:1 dr

^{i}PrMgCl·LiCl (1.14 M in THF, 7.06 mL, 8.10 mmol, 1.20 equiv) was added dropwise to a mixture of bis-boronic ester **16** (2.46 g, 6.72 mmol, 1.00 equiv) and sulfoxide **8** (3.26 g, 7.51 mmol, 1.10 equiv) in CH_2Cl_2 (0.20 M with respect to boronic ester, 33.40 mL) at –78 °C and the resulting solution was stirred 1 h at the same temperature (*pale yellow solution*). After warming to room temperature, the reaction mixture was heated at 40 °C for 4 h (*turbid white solution*). The reaction mixture was then cooled to room temperature, and it was quenched with sat. aq. NH_4Cl (20 mL). The aqueous phase was extracted with Et_2O (50×3 mL). The combined organics were dried over anhydrous Na_2SO_4 and filtered over a short pad of SiO_2 deactivated with Et_2O: Et_3N 1% in order to remove the residual TIB acid. The organics were concentrated under reduced pressure and the crude residue was purified by flash column chromatography (SiO_2, pentane: CH_2Cl_2 4:1) to afford the title compound **17** (1.92 g, 68% yield, >20:1 dr) as a colourless oil.

^{1}H NMR (400 MHz, Chloroform-*d*) δ 5.79 (ddt, *J* = 17.0, 10.1, 6.9 Hz, 1H, *H*-33), 4.99 (ddt, *J* = 17.0, 2.2, 1.1 Hz 1H, *H*-32), 4.91 (ddt, *J* = 10.1, 2.2, 1.1 Hz, 1H, *H*-32), 2.19 – 2.06 (m, 2H, *H*-34), 1.49 – 1.28 (m, 5H, *H*-36, 37, 38), 1.24 (s, 12H, CH_3-BPin), 1.22 (s, 12H, CH_3-BPin), 1.17 – 1.11 (m, 1H, *H*-36 *or* 37 *or* 38), 0.91 (s, 9H, *H*-41, 42, 43), 0.87 (d, *J* = 7.3 Hz, 1H, *H*-35), 0.81 – 0.72 (m, 1H, *H*-39). See spectrum.

^{13}C NMR (101 MHz, Chloroform-*d*) δ 138.89 (*C*H-33), 114.81 (*C*H_2-32), 83.04 (*C*O-BPin), 82.88 (*C*O-BPin), 35.64 (*C*H_2-34), 32.16 (*C*-40), 31.53 (*C*H_2-36), 30.15 (*C*H_2-37), 29.70 (*C*H_3-41, 42, 43), 27.59 (*C*H_2-38), 25.20 (*C*H_3-BPin), 25.11 (*C*H_3-BPin), 24.99 (*C*H_3-BPin), 24.96 (*C*H_3-BPin). Boron-bound *C*H-35, 39 are not detected due to quadrupolar relaxation. See spectrum.

IR (neat) ν_{max} (cm^{-1}): 2977, 2929, 2863, 1371, 1313, 1243, 1214, 1143, 1109, 968, 908, 864

HRMS (MALDI) calculated for $C_{24}H_{46}B_2O_6Na$ $[M+Na]^+$: 443.3483, found: 443.3487.

$[\alpha]_D^{25}$ = –16.0 (c 0.93, CH_2Cl_2)

Rf = 0.2 (60:40 pentane/CH_2Cl_2, stained with *p*-anisaldehyde).

17-mix (1:1 dr) was prepared using racemic sulfoxide **8** under otherwise reaction conditions.

^{13}C NMR spectrum of **17-mix** (dr 1:1) is enclosed for comparison with diastereomerically pure **17** (>20:1 dr). See spectrum.

2,2',2''-((4*R*,8*S*)-9,9-Dimethyldecane-1,4,8-triyl)tris(4,4,5,5-tetramethyl-1,3,2-dioxaborolane) (18)

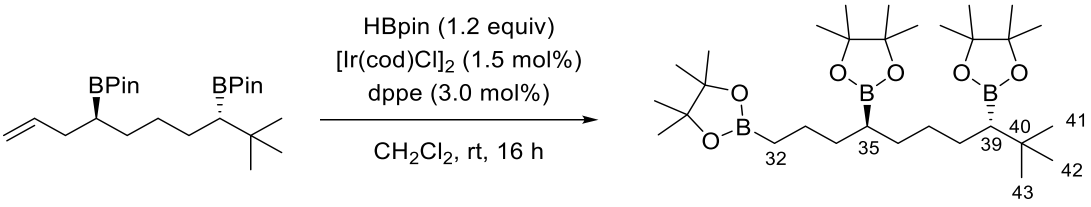

17 **18**, 83% yield

Pinacolborane (0.73 mL, 5.00 mmol, 1.20 equiv) and alkene **17** (1.75 g, 4.16 mmol, 1.00 equiv) were added successively to a solution of $[Ir(cod)Cl]_2$ (42.00 mg, 0.06 mmol, 0.015 equiv) and 1,2-bis(diphenylphosphino)ethane (dppe) (49.00 mg, 0.12 mmol, 0.03 equiv) in CH_2Cl_2 (0.30 M, 14.00 mL) at room temperature. The reaction mixture was then stirred for16 h at room temperature. The reaction was quenched with methanol (5 mL) and extracted with diethyl ether (3x20 mL). The organics were dried over anhydrous Na_2SO_4 and evaporated under reduced pressure. The crude residue was purified by column chromatography (pentane/Et_2O 9:1) to afford the title compound **18** (1.90 g, 83% yield) as a colourless oil.

TLC: R_f = 0.45 (9:1 pentane/Et_2O, stained with *p*-anisaldehyde)

^{1}H NMR (400 MHz, Chloroform-*d*) δ 1.46 – 1.28 (m, 9H), 1.24 (s, 12H, C*H*$_3$-BPin), 1.23 (s, 12H, C*H*$_3$-BPin), 1.22 (s, 12H, C*H*$_3$-BPin), 1.20 – 1.09 (m, 2H), 0.91 (s, 9H, s, 12H, *H*-41, 42, 43), 0.81 – 0.70 (m, 3H). See spectrum.

^{13}C NMR (101 MHz, Chloroform-*d*) δ 82.90 (*C*O-BPin), 82.86 (*C*O-BPin), 82.83 (*C*O-BPin), 34.37 (*C*H$_2$-), 32.15 (*C*-40), 31.95 (*C*H$_2$-), 30.30 (*C*H$_2$-), 29.72 (*C*H$_3$-41, 42, 43), 27.65 (*C*H$_2$-), 25.21 (*C*H$_3$-BPin), 25.11 (*C*H$_3$-BPin), 24.96 (*C*H$_3$-BPin), 23.84 (*C*H$_2$-). Boron-bound *C*H$_2$-32, *C*H-35, 39 are not detected due to quadrupolar relaxation. See spectrum.

IR (neat) ν_{max} (cm^{-1}): 2977, 2927, 2864, 1462, 1371, 1313, 1242, 1144, 968, 848, 671, 578

HRMS (MALDI) calculated for $C_{30}H_{59}B_3O_6Na$ $[M+Na]^+$: 571.4498, found: 571.4489.

$[\alpha]_D^{25}$ = –12.1 (c 0.93, CH_2Cl_2)

2,2',2''-((3*S*,7*R*,11*S*)-2,2-Dimethyltetradec-13-ene-3,7,11-triyl)tris(4,4,5,5-tetramethyl-1,3,2-dioxaborolane) (19)

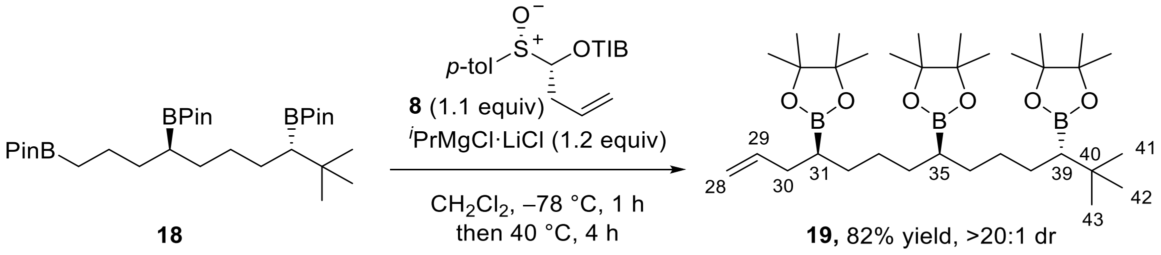

^{i}PrMgCl·LiCl (1.14 M in THF, 3.55 mL, 4.05 mmol, 1.20 equiv) was added dropwise to a mixture of tris(boronic ester) **18** (1.85 g, 3.37 mmol, 1.00 equiv) and sulfoxide **8** (1.64 g, 3.71 mmol, 1.10 equiv) in CH_2Cl_2 (0.20 M with respect to boronic ester, 16.80 mL) at –78 °C and the resulting solution was stirred 1 h at that temperature (*pale yellow solution*). After warming to room temperature, the reaction mixture was heated at 40 °C for 4 h (*turbid white solution*). The reaction mixture was then cooled to room temperature, and it was quenched with sat. aq. NH_4Cl (20 mL). The aqueous phase was extracted with Et_2O (20×3 mL). The combined organics were dried over anhydrous Na_2SO_4 and filtered over a short pad of SiO_2 deactivated with Et_2O: Et_3N 1% in order to remove the residual TIB acid. The organics were concentrated under reduced pressure and the crude residue was purified by flash column chromatography (SiO_2, pentane: CH_2Cl_2 3:2) to afford the title compound **19** (1.63 g, 82% yield, >20:1 dr) as a colourless oil. **TLC**: R_f = 0.15 (30:70 pentane/DCM, stained with *p*-anisaldehyde).

^{1}H NMR (400 MHz, Chloroform-*d*) δ 5.79 (ddt, *J* = 17.1, 10.1, 6.9 Hz, 1H, *H*-29), 4.98 (dt, *J* = 17.1, 1.8 Hz, 1H, *H*-28), 4.91 (ddt, *J* = 10.1, 2.3, 1.1 Hz, 1H, *H*-28), 2.20 – 2.05 (m, 2H, *H*-30), 1.45 – 1.26 (m, 11H, *H*-CH_2), 1.24 (s, 12H, C*H*$_3$-BPin), 1.22 (s, 24H, C*H*$_3$-BPin), 1.18 – 1.09 (m, 1H, *H*-CH_2), 1.09 – 1.00 (m, 1H, *H*-31), 0.91 (s, 10H, s, 9H, *H*-41, 42, 43 + *H*-35), 0.79 – 0.73 (m, 1H, *H*-39). *See spectrum.*

^{13}C NMR (101 MHz, Chloroform-*d*) δ 138.96 (*C*H-29), 114.76 (*C*H-28), 83.01 (*C*O-BPin), 82.86 (*C*O-BPin), 82.83 (*C*O-BPin), 35.57 (*C*H$_2$-30), 32.15 (*C*-40), 31.96 (*C*H$_2$-), 31.75 (*C*H$_2$-), 31.31 (*C*H$_2$-), 30.23 (*C*H$_2$-), 29.71 (*C*H$_3$-41, 42, 43), 28.82 (*C*H$_2$-), 27.64 (*C*H$_2$-), 25.21 (*C*H$_3$-BPin), 25.12 (*C*H$_3$-BPin), 25.01 (*C*H$_3$-BPin), 24.97 (*C*H$_3$-BPin). Boron-bound *C*H-31,-35,-39 are not detected due to quadrupolar relaxation. *See spectrum.*

IR (neat) ν_{max} (cm^{-1}): 2977, 2926, 1371, 1313, 1269, 1241, 1214, 1109, 968, 909, 864

HRMS (MALDI) calculated for $C_{34}H_{65}B_3O_6Na$ $[M+Na]^+$: 625.4969, found: 625.4978.

$[\alpha]_D^{25}$ = –12.0 (c 1.0, CH_2Cl_2)

2,2',2'',2'''-((4*R*,8*R*,12*S*)-13,13-dimethyltetradecane-1,4,8,12-tetrayl)tetrakis(4,4,5,5-tetramethyl-1,3,2-dioxaborolane) (20)

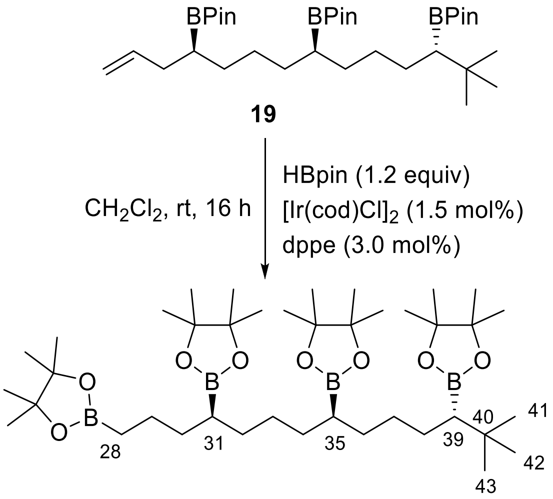

20, 76% yield

Pinacolborane (0.46 mL, 3.18 mmol, 1.20 equiv) and alkene **19** (1.60 g, 2.65 mmol, 1.00 equiv) were added successively to a solution of $[Ir(cod)Cl]_2$ (26.70 mg, 0.04 mmol, 0.015 equiv) and 1,2-bis(diphenylphosphino)ethane (dppe) (31.60 mg, 0.08 mmol, 0.03 equiv) in CH_2Cl_2 (0.30 M, 9.00 mL) at room temperature. The reaction mixture was then stirred 16 h at room temperature. The reaction was quenched with methanol (2 mL) and extracted with diethyl ether (3x20 mL). The organics were dried over anhydrous Na_2SO_4 and evaporated under reduced pressure. The crude residue was purified by column chromatography (pentane/Et_2O 9:1) to afford the title compound **20** (1.49 g, 76% yield) as a colourless oil. **TLC**: R_f = 0.2 (90:10 pentane/Et_2O, stained with *p*-anisaldehyde).

^{1}H NMR (400 MHz, Chloroform-*d*) δ 1.40 – 1.30 (m, 14H, *H*-CH_2), 1.23 (s, 12H, C*H*$_3$-BPin), 1.22 (s, 12H, C*H*$_3$-BPin), 1.21 (s, 12H, C*H*$_3$-BPin), 1.20 (s, 12H, C*H*$_3$-BPin), 1.16 – 1.02 (m, 2H, *H*-CH_2), 0.90 (s, 9H, *H*-41, 42, 43), 0.93 – 0.84 (m, 2H), 0.77 – 0.71 (m, 3H). See spectrum.

^{13}C NMR (101 MHz, Chloroform-*d*) δ 82.87 (*C*O-BPin), 82.84 (*C*O-BPin), 82.79 (*C*O-BPin), 82.79 (*C*O-BPin), 34.30 (*C*H$_2$-), 32.13 (*C*-40), 32.01 (*C*H$_2$-), 31.89 (*C*H$_2$-), 31.74 (*C*H$_2$-), 30.24 (*C*H$_2$-), 29.70 (*C*H$_3$-41, 42, 43), 29.07 (*C*H$_2$-), 27.65 (*C*H$_2$-), 25.20 (*C*H$_3$-BPin), 25.11 (*C*H$_3$-BPin), 24.96 (*C*H$_3$-BPin), 23.82 (*C*H$_2$-). Boron-bound *C*H$_2$-28 and *C*H-31,-35,-39 are not detected due to quadrupolar relaxation. See spectrum.

IR (neat) ν_{max} (cm^{-1}): 2977, 2927, 2861, 1462, 1378, 1371, 1313, 1268, 1214, 1143, 968, 948, 870, 686, 579.

HRMS (MALDI) calculated for $C_{40}H_{78}B_4O_8Na$ $[M+Na]^+$: 753.5986, found: 753.5994.

$[\alpha]_D^{25}$ = –8.0 (c 0.5, CH_2Cl_2)

2,2',2'',2'''-((3*S*,7*R*,11*S*,15*S*)-2,2-dimethyloctadec-17-ene-3,7,11,15-tetrayl)tetrakis(4,4,5,5-tetramethyl-1,3,2-dioxaborolane) (6)

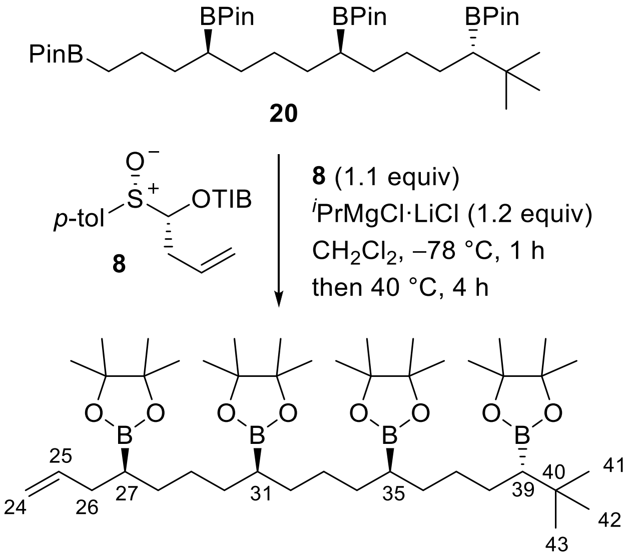

6, 89% yield, >20:1 dr

^{i}PrMgCl·LiCl (1.14 M in THF, 2.13 mL, 2.43 mmol, 1.20 equiv) was added dropwise to a mixture of tetra(boronic ester) **20** (1.45 g, 2.02 mmol, 1.00 equiv) and sulfoxide **8** (0.98 g, 2.23 mmol, 1.10 equiv) in CH_2Cl_2 (0.20 M with respect to boronic ester, 10.00 mL) at –78 °C and the resulting solution was stirred 1 h at the same temperature (*pale yellow solution*). After warming to room temperature, the reaction mixture was heated at 40 °C for 4 h (*turbid white solution*). The reaction mixture was then cooled to room temperature, and it was quenched with sat. aq. NH_4Cl (20 mL). The aqueous phase was extracted with Et_2O (20×3 mL). The combined organics were dried over anhydrous Na_2SO_4 and filtered over a short pad of SiO_2 deactivated with Et_2O: Et_3N 1% in order to remove the residual TIB acid. The organics were concentrated under reduced pressure and the crude residue was purified by flash column chromatography (SiO_2, pentane: CH_2Cl_2 3:2) to afford the title compound **6** (1.41 g, 89% yield, >20:1 dr) as a colourless oil.

TLC: R_f = 0.32 (90:10 pentane/Et_2O, stained with *p*-anisaldehyde)

^{1}H NMR (400 MHz, Chloroform-*d*) δ 5.78 (ddt, *J* = 17.0, 10.1, 6.9 Hz, 1H, *H*-25), 4.98 (dd, *J* = 17.1, 1.9 Hz, 1H, *H*-24), 4.90 (dd, J = 10.2, 2.2 Hz, 1H, *H*-24), 2.12 (m, 2H, *H*-26), 1.42 –1.25 (s, 16H, *H*-CH_2), 1.23 (s, 12H, C*H*$_3$-BPin), 1.21 (s, 36H, C*H*$_3$-BPin), 1.14 – 1.00 (m, 2H, *H*-CH_2), 0.90 (s, 9H, *H*-41, 42, 43), 0.87 – 0.83 (s, 2H), 0.79 – 0.74 (m, 2H). See spectrum.

^{13}C NMR (101 MHz, Chloroform-*d*) δ 138.94 (*C*H-25), 114.73 (*C*H_2-24), 82.99 (*C*O-BPin), 82.83 (*C*O-BPin), 82.79 (*C*O-BPin), 82.79 (*C*O-BPin), 35.58 (*C*H_2-26), 32.13 (*C*-40), 32.03 (*C*H_2-), 31.91 (*C*H_2-), 31.80 (*C*H_2-), 31.69 (*C*H_2-), 31.32 (*C*H_2-), 30.24 (*C*H_2-), 29.70 (*C*H_3-41, 42, 43), 29.03 (*C*H_2-), 28.82

(*C*H_2-), 27.64 (*C*H_2-), 25.19 (*C*H_3-BPin), 25.11 (*C*H_3-BPin), 24.99 (*C*H_3-BPin), 24.96 (*C*H_3-BPin). Boron-bound *C*H-27,-31,-35,-39 are not detected due to quadrupolar relaxation. *See spectrum.*

IR (neat) ν_{max} (cm^{-1}): 2977, 2923, 2854, 1462, 1371, 1312, 1143, 968, 863, 673, 671.

HRMS (MALDI) calculated for $C_{44}H_{84}B_4O_8Na$ $[M+Na]^+$: 807.6458, found: 807.6463.

$[\alpha]_D^{25} = -8.0$ (c 1.0, CH_2Cl_2)

(3*S*,7*R*,11*S*,15*S*)-2,2-Dimethyloctadec-17-ene-3,7,11,15-tetraol (21)

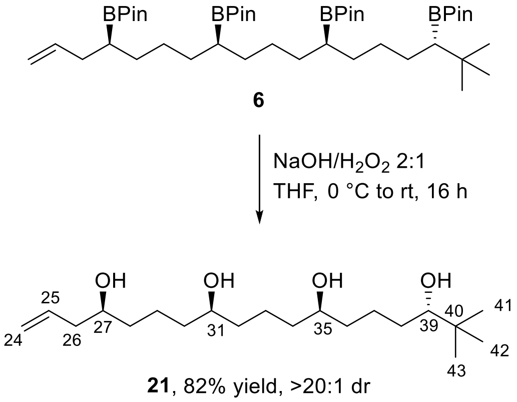

To a solution of tetra(boronic ester) **6** (0.73 g, 0.99 mmol) in THF (10.00 mL, 0.10 M with respect to boronic ester), a preformed solution of NaOH 3.00 M (6.00 mL) and H_2O_2 30% v/v (3.00 mL) was added dropwise at 0 °C. The reaction mixture was vigorously stirred 16 h at room temperature. Sat. aq. $Na_2S_2O_3$ (20 mL) was added dropwise at 0 °C under stirring and the layers were separated. The aqueous phase was extracted with Et_2O (3×20 mL). The combined organics were dried over anhydrous Na_2SO_4, filtered and concentrated under reduced pressure. The crude mixture was purified by flash column chromatography (SiO_2, EtOAc 100% to EtOAc with MeOH 10%) to afford the title compound **21** (0.26 g, 82% yield, >20:1 dr) as a white solid.

TLC: R_f = 0.43 (90:10 ethyl acetate/MeOH, stained with *p*-anisaldehyde)

^{1}H NMR (500 MHz, Methanol-*d*4) δ 5.88 (ddt, *J* = 17.2, 10.2, 7.0 Hz, 1H, *H*-25), 5.08 (dd, *J* = 17.1, 2.1 Hz, 1H, *H*-24’), 5.03 (d, *J* = 10.2, 2.1 Hz, 1H, *H*-24’’), 4.58 (s, 1H, O-*H*), 3.63 – 3.58 (m, 1H, *H*-27), 3.58 – 3.51 (m, 2H, *H*-35, 31), 3.14 (d, *J* = 10.3 Hz, 1H, *H*-39), 2.35 – 2.08 (m, 2H, *H*-26), 1.75 – 1.25 (m, 18H, *H*-CH_2), 0.90 (s, 9H, *H*-41, 42, 43). See spectrum.

^{13}C NMR (126 MHz, Methanol-*d*4) δ 136.55 (*C*H-25), 117.12 (*C*H_2-24), 80.53 (*C*H-39), 72.37 (*C*H-35 *or* 31), 72.36 (*C*H-35 *or* 31), 72.07 (*C*H-27), 43.01 (*C*H_2-26), 38.52 (*C*H_2-), 38.44 (*C*H_2-), 38.38 (*C*H_2-), 38.33 (*C*H_2-), 37.81 (*C*H_2-28), 35.93 (*C*-40), 32.32 (*C*H_2-38), 26.35 (*C*H_3-41, 42, 43), 24.38 (*C*H_2-37), 22.95 (*C*H_2-29 *or* 33), 22.88 (*C*H_2-29 *or* 33). See spectrum.

IR (neat) ν_{max} (cm^{-1}): 3221 (O-H), 2959, 2911, 2863, 1455, 1121, 1089, 1003, 912, 897, 884, 662.

HRMS (MALDI) calculated for $C_{20}H_{41}O_4$ $[M+H]^+$: 345.3005, found: 345.3015.

$[\alpha]_D^{25}$ = –16.0 (c 1.0, CH_2Cl_2)

m.p. (MeOH): 112–114 °C

Tetraol 21 Full Chemical Shift Assignment

For the full chemical shift assignment of tetraol **21** in Pyridine-*d*5, two HSQC-TOCSY (Heteronuclear Single Quantum Coherence-TOtal COrrelation SpectroscopY) spectra were required at mixing times of 30 ms and 60 ms. The spectra recorded with a 60 ms mixing time showed $^{1\text{-}3}J_{CH}$ correlations, which were used to assign the chemical shifts of the stereogenic carbon atoms, and their respective protons. The spectra recorded with a 60 ms mixing time showed $^{1\text{-}4}J_{CH}$ correlations, which were used to assign the chemical shifts of carbon atoms adjacent to stereocenters. Spectra were recorded on a 500 MHz spectrometer fitted cryo-enhanced ^{13}C probe and were aliased to (4505.5, 4401.4) with lowest frequencies at (18.8, 1961.2) for ^{1}H and ^{13}C respectively (values recorded in Hz) achieving a spectral resolution of 4.3 Hz in f1. NMR sample prepared with 5 mg of tetraol **21** dissolved in *ca.* 600 µL pyridine-d5 (≥99.96% D) in a 5 mm NMR tube. *See HSQC-TOCSY spectra*.

^{1}H NMR (500 MHz, Pyridine-*d*5) δ 6.24 – 6.10 (m, 1H, *H*-25), 5.89 (m, 1H, O*H*), 5.78 – 5.67 (m, 3H, O*H*), 5.21 (br. d, J = 17.2, 1H, *H*-24-*trans*), 5.14 (br. d, J = 10.2 Hz, 1H, *H*-24-*cis*), 3.97 (m, 1H, *H*-27), 3.96 (m, 1H, *H*-35), 3.93 (m, 1H, *H*-31), 3.46 (m, 1H, *H*-39), 2.47 (m, 2H, *H*-26’, 26’’), 2.15 (m, 1H, *H*-37’), 2.14 (m, 1H, *H*-29’), 2.14 (m, 1H, *H*-33’), 1.95 (m, 1H, *H*-37’’), 1.86 (m, 1H, *H*-36’), 1.81 (m, 1H, *H*-33’’), 1.79 (m, 1H, *H*-29’), 1.77 (m, 2H, *H*-34’, *H*-34’’), 1.75 (m, 4H, *H*-32’, *H*-32’’, *H*-34’, *H*-34’’), 1.71 (m, 1H, *H*-36’’), 1.67 (m, 2H, *H*-38’, *H*-38’’), 1.77 (m, 2H, *H*-34’, *H*-34’’), 1.08 (s, 9H, *H*-41, 42, 43). *See spectrum.*

^{13}C NMR (126 MHz, Pyridine-*d*5) δ 137.31 (*C*H-25), 116.77 (*C*H_2-24), 79.34 (*C*H-39), 71.58 (*C*H-35), 71.51 (*C*H-31), 71.19 (*C*H-27), 43.52 (*C*H_2-26), 39.25 (*C*H_2-34), 39.17 (*C*H_2-32), 39.07 (*C*H_2-30), 39.06 (*C*H_2-36), 38.47 (*C*H_2-28), 35.88 (*C*-40), 32.73 (*C*H_2-38), 26.77 (*C*H_3-41, 42, 43), 24.67 (*C*H_2-37), 23.35 (*C*H_2-33), 23.23 (*C*H_2-29). *See spectrum.*

(5*S*,9*S*,13*R*,17*S*)-5-Allyl-17-(*tert*-butyl)-3,3,19,19-tetraethyl-9,13-bis((triethylsilyl)oxy)-4,18-dioxa-3,19-disilahenicosane (22)

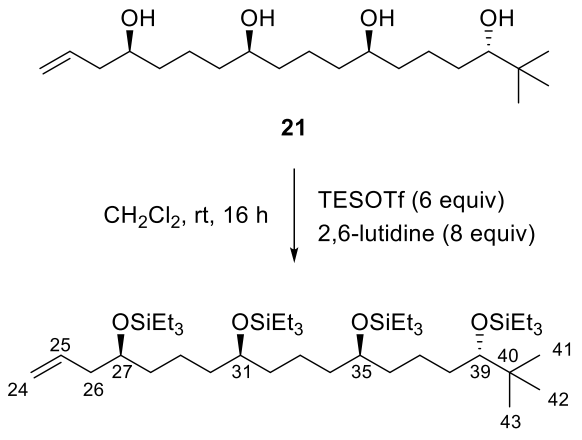

22, 89% yield

To a suspension of alcohol **21** (0.26 g, 0.75 mmol) in CH_2Cl_2 (3.80 mL, 0.20 M), 2,6-lutidine (0.70 mL, 6.00 mmol, 8.00 equiv) was added at room temperature. To the reaction mixture, triethylsilyl trifluoromethanesulfonate TESOTf (1.00 mL, 4.50 mmol, 6.00 equiv) was added dropwise at 0 °C. The resulting clear solution was stirred 16 h at room temperature. Water (2 mL) was added and the layers were separated. The aqueous phase was extracted with Et_2O (3×10 mL). The combined organics were dried over anhydrous Na_2SO_4, filtered and concentrated under reduced pressure. The crude mixture was purified by flash column chromatography (SiO_2, pentane: Et_2O 1% to 2%) to afford the title compound **22** (0.54 g, 89%, >20:1 dr) as a colourless oil. **TLC**: *R*f = 0.30 (pentane: Et_2O 2%, stained with *p*-anisaldehyde).

^{1}H NMR (500 MHz, Chloroform-*d*) δ 5.81 (ddt, *J* = 17.3, 10.3, 7.1 Hz, 1H, *H*-25), 5.06 – 5.01 (m, 2H, *H*-24), 3.69 (m, 1H, C*H*-27), 3.67 – 3.60 (m, 2H, C*H*-35, 31), 3.24 (d, *J* = 6.2 Hz, 1H, *H*-39), 2.23 – 2.19 (m, 2H, *H*-26), 1.49 – 1.34 (m, 14H, CH_2), 1.29 – 1.23 (m, 4H), 0.99 – 0.94 (m, 36H, CH_3-OTES), 0.85 (s, 9H, *H*-41, 42, 43), 0.64 – 0.57 (m, 24H, CH_2-OTES). See spectrum.

^{13}C NMR (126 MHz, Chloroform-*d*) δ 135.49 (*C*H-25), 116.80 (*C*H$_2$-24), 81.44 (*C*H-39), 72.64 (*C*H-35), 72.47 (*C*H-31), 72.17 (*C*H-27), 42.15 (*C*H$_2$-26), 38.02 (*C*H$_2$-), 37.73 (*C*H$_2$-), 37.70 (*C*H$_2$-), 37.51 (*C*H$_2$-), 37.32 (*C*H$_2$-), 35.84 (*C*-40), 33.70 (*C*H$_2$-38), 26.46 (*C*H$_3$-41, 42, 43), 23.85 (*C*H$_2$-37), 21.41 (*C*H$_2$-33), 21.34 (*C*H$_2$-29), 7.32 (*C*H$_3$CH$_2$Si), 7.13 (*C*H$_3$CH$_2$Si), 7.12 (*C*H$_3$CH$_2$Si), 7.08 (*C*H$_3$CH$_2$Si), 5.82 (CH$_3$*C*H$_2$Si), 5.31 (CH$_3$*C*H$_2$Si), 5.29 (CH$_3$*C*H$_2$Si), 5.22 (CH$_3$*C*H$_2$Si). See spectrum.

IR (neat) ν_{max} (cm^{-1}): 2952, 2910, 2875, 1641 (C=C), 1458, 1414, 1363, 1237, 1105, 1006, 911, 725, 671.

HRMS (MALDI) calculated for $C_{44}H_{96}O_4Si_4Na$, $[M+Na]^+$: 823.6278, found: 823.6270

$[\alpha]_D^{25} = -12.0$ (c 1.0, CH_2Cl_2)

(5*S*,9*R*,13*R*,17*R*)-5-(*tert*-Butyl)-3,3,19,19-tetraethyl-17-(3-(4,4,5,5-tetramethyl-1,3,2-dioxaborolan-2-yl)propyl)-9,13-bis((triethylsilyl)oxy)-4,18-dioxa-3,19-disilahenicosane (4, Fragment A)

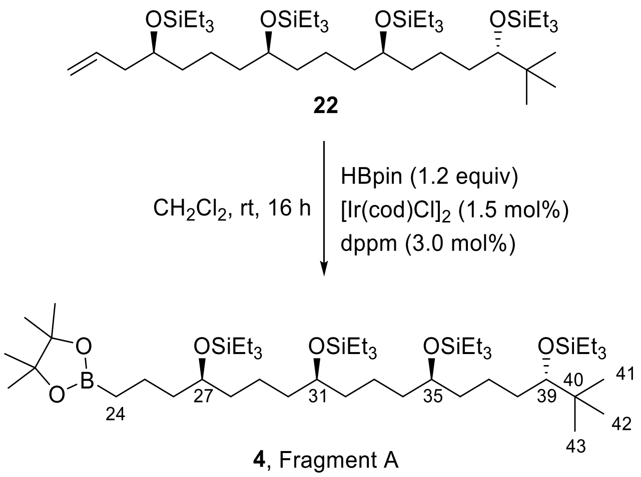

4, Fragment A
66% yield

Pinacolborane (0.10 mL, 0.69 mmol, 1.20 equiv) and alkene **22** (0.46 g, 0.57 mmol, 1.00 equiv) were added successively to a solution of [Ir(cod)Cl]$_2$ (5.80 mg, 0.009 mmol, 0.015 equiv) and 1,2-bis(diphenylphosphino)methane (dppm) (31.60 mg, 0.018 mmol, 0.03 equiv) in CH_2Cl_2 (0.30 M, 1.90 mL) at room temperature. The reaction mixture was then stirred 16 h at room temperature. The reaction was quenched with methanol (1 mL) and extracted with diethyl ether (3x10 mL). The organics were dried over anhydrous Na_2SO_4 and evaporated under reduced pressure. The crude residue was purified by column chromatography (pentane/Et_2O 2%) to afford the title compound **4**, Fragment A (0.35 g, 66% yield, >20:1 dr) as a colourless oil.

TLC: R_f = 0.2 (pentane/Et_2O 2%, stained with *p*-anisaldehyde)

^{1}H NMR (500 MHz, Chloroform-*d*) δ 3.66 – 3.59 (m, 3H, *H*-27, 31, 35), 3.24 (d, *J* = 6.1 Hz, 1H, *H*-39), 1.46 – 1.34 (m, 20H, C*H*$_2$), 1.29 – 1.26 (m, 2H, C*H*$_2$), 1.24 (s, 12H, C*H*$_3$-BPin), 0.98 – 0.94 (m, 36H, C*H*$_3$-OTES), 0.84 (s, 9H, *H*-41, 42, 43), 0.76 (d, *J* = 7.2 Hz, 2H, *H*-24), 0.64 – 0.56 (m, 24H, C*H*$_2$-OTES). See spectrum.

^{13}C NMR (126 MHz, Chloroform-*d*) δ 83.00 (*C*O-BPin), 81.44 (*C*H-39), 72.65 (*C*H-35), 72.55 (*C*H-31), 72.43 (*C*H-27), 40.24 (*C*H$_2$-26), 38.02 (*C*H$_2$-), 37.77 (*C*H$_2$-), 37.73 (*C*H$_2$-), 37.70 (*C*H$_2$-), 37.50 (*C*H$_2$-), 35.84 (*C*-40), 33.70 (*C*H$_2$-38), 26.46 (*C*H$_3$-41, 42, 43), 24.98 (*C*H$_3$-BPin), 24.95 (*C*H$_3$-BPin), 23.85 (*C*H$_2$-), 21.50 (*C*H$_2$-), 21.37 (*C*H$_2$-), 20.06 (*C*H$_2$-), 11.33 (broad, *C*H$_2$-24), 7.32 (*C*H$_3$CH$_2$Si), 7.14 (*C*H$_3$CH$_2$Si), 7.13 (*C*H$_3$CH$_2$Si), 5.82 (CH$_3$*C*H$_2$Si), 5.31 (CH$_3$*C*H$_2$Si), 5.29 (CH$_3$*C*H$_2$Si), 5.26 (CH$_3$*C*H$_2$Si). See spectrum.

IR (neat) ν_{max} (cm^{-1}): 2956, 2921, 2852, 1462, 1377, 1230, 1155, 1082, 887, 793, 468.

HRMS (MALDI) calculated for $C_{50}H_{109}BO_6Si_4Na$, [M+Na]+: 951.7296, found: 951.7288.

$[\alpha]_D^{25} = -8.0$ (c 1.0, CH_2Cl_2)

(*S*)-3,4-bis(4,4,5,5-Tetramethyl-1,3,2-dioxaborolan-2-yl)butyl 2,4,6-triisopropylbenzoate (23)

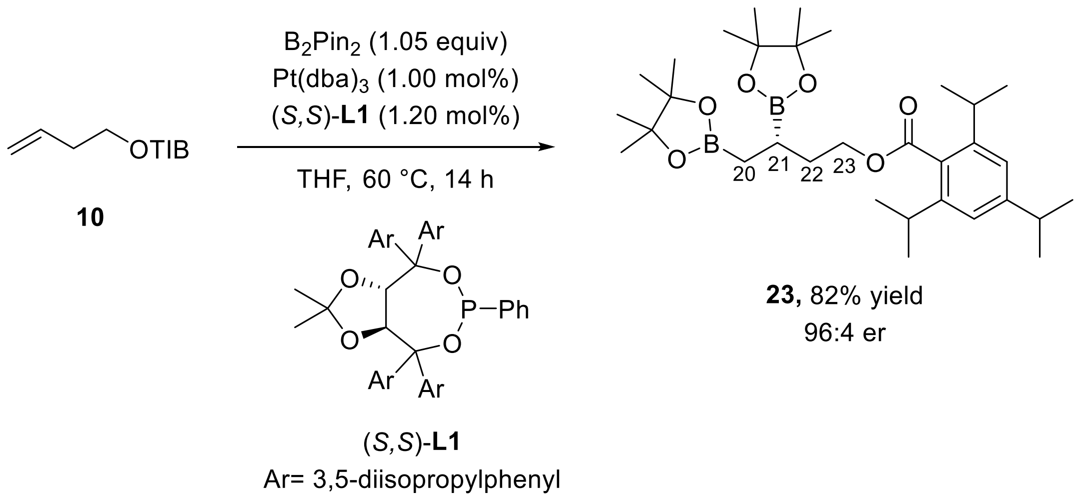

According to the modified literature procedure,[12] to a flame dried Schlenk tube equipped with a magnetic stir bar was added Pt(dba)$_3$ (0.06 g, 0.07 mmol, 1.00 mol%), (*S*,*S*)-**L1** (0.07 g, 0.08 mmol, 1.20 mol%) and B_2Pin_2 (1.78 g, 7.00 mmol, 1.05 equiv) under a nitrogen atmosphere. After addition of solids, the Schlenk was evacuated and refilled with N_2 three times. Then, THF (1.00 M with respect to the alkene **10**, 6.60 mL) was added via syringe. The solution was heated to 80 °C in an oil bath for 30 minutes. The reaction mixture was then cooled to room temperature and alkene **10** (2.00 g, 6.60 mmol, 1.00 equiv) was added neat. The reaction mixture was stirred 14 h at 60 °C. The reaction mixture was then cooled to room temperature and the solvent was evaporated under reduced pressure. The crude mixture was purified by automated flash column chromatography (BIOTAGE HC-100g, pentane: EtOAc 1-20%) to afford the title compound (*S*)-**23** (3.00 g, 82% yield) as a colourless oil. The enantiomeric excess was determined upon oxidation of the bis(boronic ester) (see **23-ox**).

TLC: R_f = 0.40 (85:15 pentane/Et_2O, stained with *p*-anisaldehyde)

^{1}H NMR (400 MHz, Chloroform-*d*) δ 6.98 (s, 2H, *H*-Ar), 4.33 (t, *J* = 6.9 Hz, 2H, *H*-23), 2.86 (hept, *J* = 6.8 Hz, 3H, *H*-*i*Pr), 1.93 – 1.88 (m, 1H, *H*-22), 1.76 – 1.71 (m, 1H, *H*-22), 1.23 (d, *J* = 7.0 Hz, 18H, C*H*$_3$-*i*Pr), 1.21 (m, 24H, C*H*$_3$-BPin), 0.89 (t, *J* = 7.1 Hz, 2H, *H*-20), 0.85 (m, 1H, *H*-21). See spectrum.

^{13}C NMR (101 MHz, Chloroform-*d*) δ 171.13 (*C*O-benzoate), 149.99 (*C*-ipso), 144.88 (*C*-ortho), 131.10 (*C*-para), 120.88 (*C*H-Ar), 83.14 (*C*O-BPin), 83.08 (*C*O-BPin), 64.65 (*C*H$_2$-23), 34.58 (*C*H-*i*Pr para), 32.19 (*C*H$_2$-22), 31.55 (*C*H-*i*Pr ortho), 25.00 (*C*H$_3$-BPin), 24.96 (*C*H$_3$-BPin), 24.88 (*C*H$_3$-BPin), 24.32 (*C*H$_3$-*i*Pr), 24.30 (*C*H$_3$-*i*Pr), 24.12 (*C*H$_3$-*i*Pr). Boron-bound *C*H$_2$-20 and *C*H-21 are not detected due to quadrupolar relaxation. See spectrum.

IR (neat) ν_{max} (cm^{-1}): 2962, 2929, 2870, 1723 (C=O), 1462, 1370 (B-O), 1315, 1250 (B-C), 1138, 1105, 1076, 968, 877, 846, 671.

HRMS (MALDI) calculated for $C_{32}H_{54}B_2O_6Na$, $[M+Na]^+$: 579.4016, found: 463.2276.

$[\alpha]_D^{25}$ = –8.0 (c 1.0, CH_2Cl_2)

*The racemic bis-boronic ester rac-**23** was prepared by diboration of **10** using a method described in the literature for similar compounds.*[13]

(*R*)-3,4-Dihydroxybutyl 2,4,6-triisopropylbenzoate (23-ox)

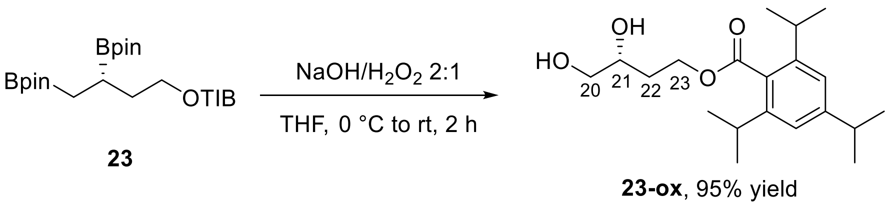

To a solution of bis-boronic ester **23** (0.07 g, 0.125 mmol) in THF (0.62 mL, 0.20 M), a preformed solution of NaOH 3M (0.36 mL) and H_2O_2 30% v/v (0.18 mL) was added dropwise at 0 °C. The reaction mixture was vigorously stirred at room temperature for 2 h. Sat. aq. $Na_2S_2O_3$ (1 mL) was added dropwise at 0 °C under stirring and the layers were separated. The aqueous phase was extracted with Et_2O (3×5 mL). The combined organics were dried over anhydrous Na_2SO_4, filtered, and concentrated under reduced pressure. The crude mixture was purified by flash column chromatography (SiO_2, pentane: Et_2O 4:1) to afford the title compound **23-ox** (40 mg, 95% yield) as a colourless oil.

TLC: R_f = 0.20 (4:1 pentane/Et_2O, stained with $KMnO_4$)

^{1}H NMR (400 MHz, Chloroform-*d*) δ 7.00 (s, 2H, *H*-Ar), 4.53 (ddd, *J* = 11.1, 7.8, 5.9 Hz, 1H, *H*-23), 4.42 (ddd, *J* = 11.1, 5.8 Hz, 1H, *H*-23), 3.88 (m, 1H, *H*-21), 3.68 (dd, *J* = 11.1, 3.2 Hz, 1H, *H*-20), 3.50 (dd, *J* = 11.1, 7.1 Hz, 1H, *H*-20), 2.89 (hept, *J* = 6.8 Hz, 1H, *H*-*i*Pr), 2.83 (hept, *J* = 6.8 Hz, 2H, *H*-*i*Pr), 2.65 (s, 1H, O-*H*), 2.14 (s, 1H, O-*H*), 1.90 – 1.84 (m, 2H, *H*-22), 1.24 (d, *J* = 6.8 Hz, 18H, CH_3-*i*Pr). See spectrum.

^{13}C NMR (101 MHz, Chloroform-*d*) δ 171.30 (*C*O-benzoate), 150.45 (*C*-ipso), 144.91 (*C*-ortho), 130.33 (*C*-para), 121.04 (*C*H-Ar), 69.36 (*C*H-21), 66.75 (*C*H_2-20), 61.99 (*C*H_2-23), 34.56 (*C*H-*i*Pr para), 32.40 (*C*H_2-22), 31.69 (*C*H-*i*Pr ortho), 24.30 (*C*H_3-*i*Pr), 24.26 (*C*H_3-*i*Pr), 24.07 (*C*H_3-*i*Pr). See spectrum.

IR (neat) ν_{max} (cm^{-1}): 3406 (O-H), 2961, 2871, 1725 (C=O), 1575, 1607 (C=C), 1462, 1384, 1363, 1284, 1252, 1139, 1103, 1076, 949, 876.

HRMS (ESI) calculated for $C_{20}H_{32}O_4Na$, $[M+Na]^+$: 359.217854, found: 359.219280.

$[\alpha]_D^{25} = -12.0$ (c 1.0, CH_2Cl_2)

Enantiomeric ratio (er) of 96:4 was determined using HPLC analysis with chiral stationary phase, IB column, ^{n}Hex: ^{i}PrOH = 97:3, 0.3 mL/min, λ = 210 nm.

t_R = 37.98 [(*R*), major], 39.72 [(*S*), minor].

*The racemic diol rac-**23-ox** was prepared by oxidation of rac-**23** under otherwise identical conditions.*

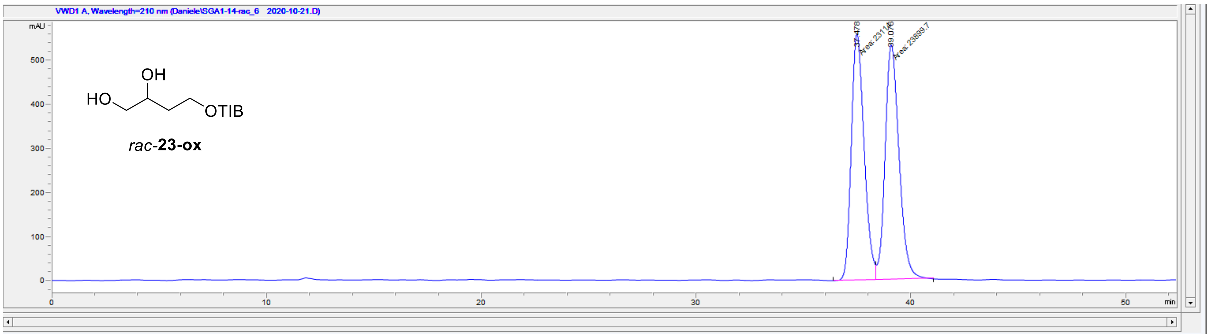

File Information	
LC-File	SGA1-14-rac_6 2020-10-21.D
File Path	C:\Chem32\1\Data\Daniele
Date	21-Oct-20, 11:38:37
Sample	SGA1-14-rac
Sample Info	IB with guard, hex:IPA 97;3 0.3 ml/min, rt, 210 nm, 5 uL injec
Barcode	
Operator	SYSTEM
Method	DF_104.M
Reference	
Analysis Time	52.344 min
Sampling Rate	0.0049 min (0.294 sec) 10786 datapoints

#	Time	Type	Area	Height	Width	Area%	Symmetry
1	37.478	MF	23114	556.9	0.6917	49.164	0.75
2	39.076	FM	23899.7	530.2	0.7513	50.836	0.747

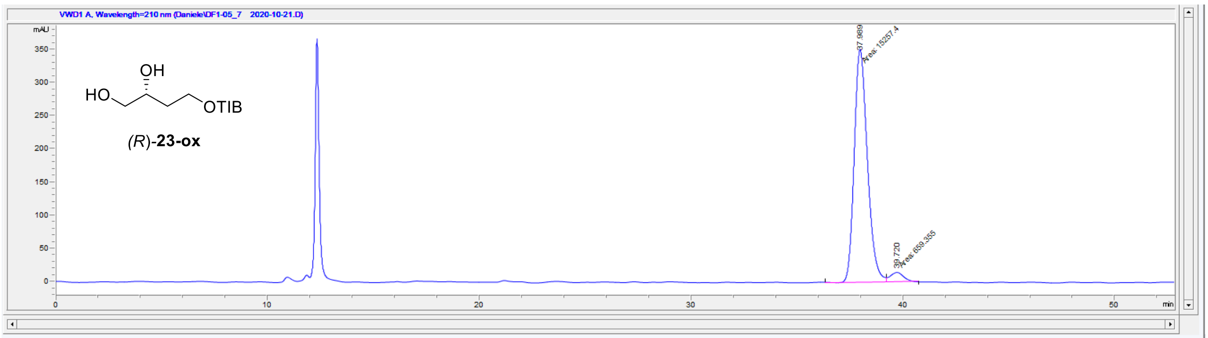

File Information	
LC-File	DF1-05_7 2020-10-21.D
File Path	C:\Chem32\1\Data\Daniele
Date	21-Oct-20, 12:34:18
Sample	DF1-05
Sample Info	IB with guard, hex:IPA 97;3 0.3 ml/min, rt, 210 nm, 5 uL injec
Barcode	
Operator	SYSTEM
Method	DF_104.M
Reference	
Analysis Time	52.82 min
Sampling Rate	0.0049 min (0.294 sec) 10884 datapoints

#	Time	Type	Area	Height	Width	Area%	Symmetry
1	37.989	MF	15257.4	352.7	0.721	95.857	0.772
2	39.72	FM	659.4	14.6	0.7516	4.143	0.943

(3*R*,5*R*)-3,5-Dihydroxyoct-7-en-1-yl 2,4,6-triisopropylbenzoate (24)

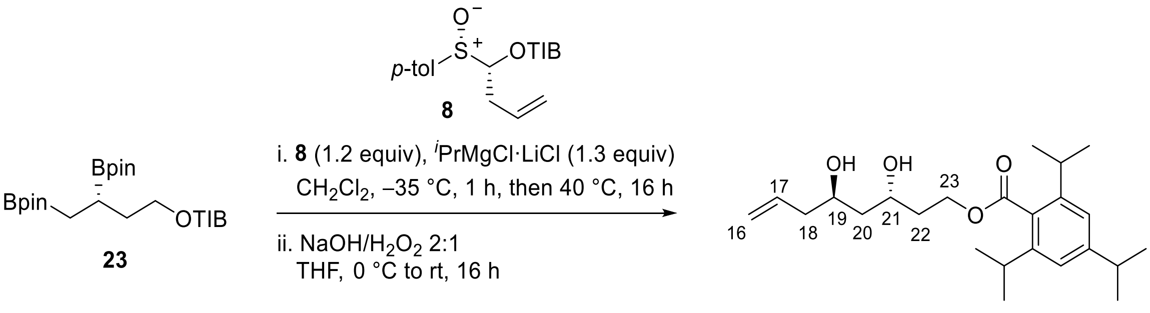

24, 65% yield over 2 steps
>20:1 dr

^{i}PrMgCl·LiCl (1.12 M in THF, 4.17 mL, 4.67 mmol, 1.30 equiv) was added dropwise to a mixture of bis(boronic ester) **23** (2.00 g, 3.60 mmol, 1.00 equiv) and sulfoxide **8** (1.90 g, 4.31 mmol, 1.20 equiv) in CH_2Cl_2 (18 mL, 0.20 M with respect to boronic ester) at –35 °C and the resulting solution was stirred for 1 h at the same temperature (*pale yellow solution*). After warming to room temperature, the reaction mixture was heated at 40 °C for 16 h (*turbid white solution*). The reaction mixture was then cooled to room temperature, and it was quenched with sat. aq. NH_4Cl (20 mL). The aqueous phase was extracted with Et_2O (50×3 mL). The combined organics were dried over anhydrous Na_2SO_4 and filtered over a short pad of SiO_2 deactivated with Et_2O: Et_3N 1% in order to remove the residual TIB acid. The organics were concentrated under reduced pressure and the crude mixture was engaged in the next step without further purification.

To a solution of crude 1,3-bis-boronic ester (3.60 mmol) in THF (18.00 mL, 0.20 M with respect to bis-boronic ester), a preformed solution of NaOH 3M (12.00 mL) and H_2O_2 30% v/v (6.00 mL) was added dropwise at 0 °C. The reaction mixture was vigorously stirred at room temperature for 16 h. Sat. aq. $Na_2S_2O_3$ (50 mL) was added dropwise at 0 °C under stirring and the layers were separated. The aqueous phase was extracted with Et_2O (3×50 mL). The combined organics were dried over anhydrous Na_2SO_4, filtered and concentrated under reduced pressure. The crude mixture was purified by automated flash column chromatography (Biotage HC-50g, ^{n}hexane: EtOAc 10-40%) to afford the title compound **24** (907 mg, 65% yield over 2 steps, >20:1 dr) as a colourless oil.

TLC: R_f = 0.3 (7:3 pentane/EtOAc, stained with *p*-anisaldehyde)

^{1}H NMR (400 MHz, Chloroform-*d*) d 7.00 (s, 2H, *H*-Ar), 5.86 – 5.75 (m, 1H, *H*-17), 5.16 – 5.12 (m, 2H, *H*-16), 4.56 (dt, *J* = 11.1, 6.9 Hz, 1H, *H*-23), 4.40 (dt, *J* = 11.3, 5.7 Hz, 1H, *H*-23), 4.12 (tdd, *J* = 7.9, 5.3, 3.4 Hz, 1H, *H*-21), 4.00 (tdd, *J* = 8.0, 5.2, 3.6 Hz, 1H, *H*-19), 2.89 (hept, *J* = 6.8 Hz, 1H, *H*-*i*Pr), 2.84 (p, *J* = 6.8 Hz, 2H, *H*-*i*Pr), 2.45 (s, 2H, O-*H*), 2.27 (m, 2H, *H*-18), 1.90 (td, *J* = 6.9, 5.5 Hz, 2H, *H*-22), 1.68 (m, 2H, *H*-20), 1.24 (d, *J* = 7.0 Hz, 18H, CH_3-*i*Pr). See spectrum.

^{13}C NMR (101 MHz, Chloroform-*d*) δ 171.37 (*C*O-benzoate), 150.39 (*C*-*ipso*), 144.89 (*C*-*ortho*), 134.57 (*C*H-17), 130.43 (*C*-*para*), 121.02 (*C*H-Ar), 118.71 (*C*H_2-16), 68.23 (*C*H-19), 66.26 (*C*H_2-21),

62.32 (CH_2-23), 42.15 (CH_2-20), 42.11 (CH_2-18), 36.50 (CH_2-22), 34.57 (*C*H-*i*Pr *para*), 31.69 (*C*H-*i*Pr *ortho*), 24.31 (CH_3-*i*Pr), 24.28 (CH_3-*i*Pr), 24.09 (CH_3-*i*Pr). See spectrum.

IR (neat) ν_{max} (cm^{-1}): 3406 (O-H), 2960, 2927, 2870, 1724 (C=O), 1606 (C=C), 1461, 1363, 1251, 1138, 1103, 1076, 915, 877.

HRMS (ESI) calculated for $C_{24}H_{39}O_4$, $[M+H]^+$: 391.2743, found: 391.2845.

$[\alpha]_D^{25}$ = –4.0 (c 1.0, CH_2Cl_2)

2-((4*R*,6*R*)-6-Allyl-2,2-dimethyl-1,3-dioxan-4-yl)ethyl 2,4,6-triisopropylbenzoate (25)

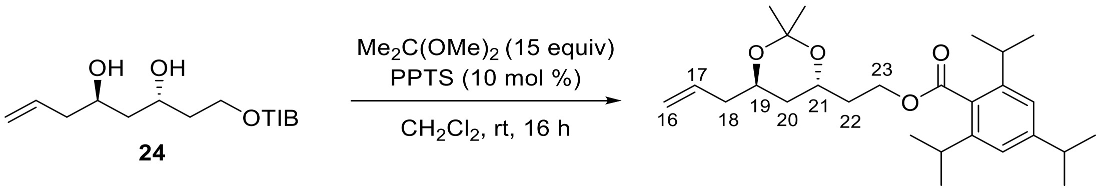

To a solution of diol **24** (0.88 g, 2.25 mmol) in CH_2Cl_2 (0.10 M, 22.50 mL), 2,2-dimethoxypropane (4.14 mL, 33.75 mmol, 15.00 equiv) and pyridinium *p*-toluene sulfonate (0.06 g, 0.225 mmol) were added. The reaction mixture was stirred 16 h at ambient temperature. Sat. aq. $NaHCO_3$ (8 mL) was added and the layers were separated. The aqueous phase was extracted with CH_2Cl_2 (3×40 mL). The combined organics were dried over anhydrous Na_2SO_4, filtered and concentrated under reduced pressure. The crude mixture was purified by flash column chromatography (SiO_2, pentane: EtOAc 95:5 with Et_3N 1%) to afford the title compound **25** (0.88 g, 91% yield, >20:1 dr) as a colourless oil.
Note: due to the acetonide lability under acidic conditions, it is recommended to use $CDCl_3$ *filtered over basic alumina or* CD_2Cl_2 *as solvent for NMR analysis.*

TLC: R_f = 0.5 (pentane: EtOAc 95:5, stained with *p*-anisaldehyde)

^{1}H NMR (400 MHz, Chloroform-*d*) δ 7.01 (s, 2H, *H*-Ar), 5.80 (ddt, *J* = 17.1, 10.2, 6.8 Hz, 1H, *H*-17), 5.12 – 5.04 (m, 2H, *H*-16), 4.44 – 4.34 (m, 2H, *H*-23), 3.96 (m, 1H, *H*-21), 3.88 (m, 1H, *H*-19), 2.94 – 3.80 (m, 3H, *H*-*i*Pr), 2.31 (ddd, *J* = 13.5, 6.7 Hz, 1H, *H*-18), 2.20 (ddd, *J* = 14.0, 6.6 Hz, 1H, *H*-18), 1.90 (ddd, *J* = 12.0, 6.6 Hz, 2H, *H*-22), 1.64 (dd, *J* = 7.7 Hz, 2H, *H*-20), 1.36 (s, 3H, CH_3-acetonide), 1.35 (s, 3H, CH_3-acetonide), 1.25 (d, *J* = 6.9 Hz, 18H, CH_3-*i*Pr). See spectrum.

^{13}C NMR (101 MHz, Chloroform-*d*) δ 171.04 (*C*O-benzoate), 150.28 (*C*-*ipso*), 144.87 (*C*-*ortho*), 134.44 (*C*H-17), 130.66 (*C*-*para*), 120.99 (*C*H-Ar), 117.11 (*C*H_2-16), 100.55 (*C*O *anti*-acetonide), 66.24 (*C*H-19), 63.61 (*C*H-21), 61.81 (*C*H_2-23), 40.25 (*C*H_2-18), 38.02 (*C*H_2-20), 35.02 (*C*H_2-22), 34.56 (*C*H-*i*Pr *para*), 31.66 (*C*H-*i*Pr *ortho*), 24.90 (*C*H_3-*anti*-acetonide), 24.86 (*C*H_3-*anti*-acetonide), 24.35 (*C*H_3-*i*Pr), 24.24 (*C*H_3-*i*Pr), 24.08 (*C*H_3-*i*Pr). See spectrum.

IR (neat) ν_{max} (cm^{-1}): 3076 (C=C-H), 2961, 2936, 2871, 1725 (C=O), 1607 (C=C), 1462, 1379, 1363, 1249, 1223, 1136, 1074, 913, 877.

HRMS (ESI) calculated for $C_{27}H_{42}O_4Na$, $[M+Na]^+$: 453.2975, found: 453.2982.

$[\alpha]_D^{25}$ = –4.0 (c 1.0, CH_2Cl_2)

2-((4*R*,6*R*)-2,2-Dimethyl-6-(3-(4,4,5,5-tetramethyl-1,3,2-dioxaborolan-2-yl)propyl)-1,3-dioxan-4-yl)ethyl 2,4,6-triisopropylbenzoate (26)

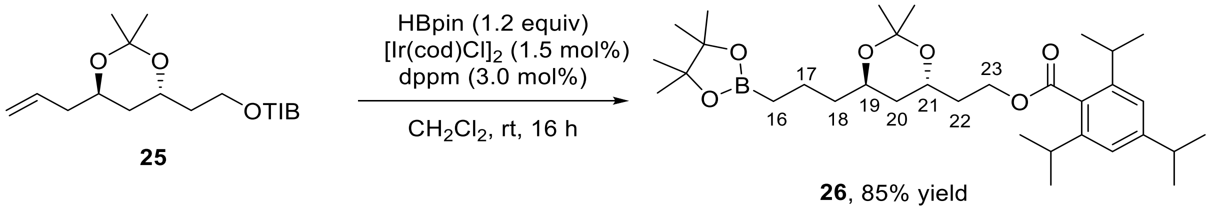

To a Schlenk tube charged with $[Ir(cod)Cl]_2$ (21 mg, 0.031 mmol, 1.5 mol%) and bis(diphenylphosphino) methane (dppm) (24 mg, 0.061 mmol, 3.0 mol%), CH_2Cl_2 (3.8 mL) was added, followed by a solution of the alkene **25** (880 mg, 2.04 mmol, 1.0 equiv) in 3 mL of CH_2Cl_2 (0.30 M overall with respect to the alkene). To this solution, pinacolborane HBPin (0.44 mL, 3.07 mmol, 1.50 equiv) was added at room temperature. The reaction mixture was stirred 16 h at room temperature. MeOH (1 mL) and water (1 mL) were added and the layers were separated. The aqueous phase was extracted with Et_2O (3×15 mL). The combined organics were dried over anhydrous Na_2SO_4, filtered and concentrated under reduced pressure. The crude mixture was purified by automated flash column chromatography (Biotage HC-10g, pentane: EtOAc 2-10%) to afford the title compound **26** (970 mg, 85% yield) as a colourless oil.

TLC: R_f = 0.5 (pentane: EtOAc 10%, stained with *p*-anisaldehyde)

^{1}H NMR (400 MHz, Methylene Chloride-d_2) δ 7.01 (s, 2H, *H*-Ar), 4.41 – 4.27 (m, 2H, *H*-23), 3.95 – 3.85 (m, 1H, *H*-21), 3.82 – 3.70 (m, 1H, *H*-19), 2.89 (hept, *J* = 6.8 Hz, 1H, *H*-*i*Pr), 2.80 (hept, *J* = 6.8 Hz, 1H, *H*-*i*Pr), 1.85 (m, 2H, *H*-22), 1.65 – 1.53 (m, 2H, *H*-20), 1.52 – 1.32 (m, 4H, *H*-28, *H*-17), 1.30 (s, 3H, CH_3-acetonide), 1.28 (s, 3H, CH_3-acetonide), 1.24 (s, 3H, CH_3-BPin), 1.22 (s, 9H, CH_3-BPin), 1.20 (m, 18H, CH_3-*i*Pr), 0.76 – 0.67 (m, 2H, *H*-16). See spectrum.

^{13}C NMR (101 MHz, Methylene Chloride-d_2) δ 171.14 (*C*O-benzoate), 150.89 (*C*-*ipso*), 145.34 (*C*-*ortho*), 131.33 (*C*-*para*), 121.41 (*C*H-Ar), 100.66 (*C*O *anti*-acetonide), 83.37 (*C*O-BPin), 66.91 (*C*H-19), 64.03 (*C*H-21), 62.33 (*C*H_2-23), 39.14 (*C*H_2-18), 39.09 (*C*H_2-20), 35.50 (*C*H_2-22), 35.03 (*C*H-*i*Pr *para*), 32.05 (*C*H-*i*Pr *ortho*), 25.20 (*C*H_3-BPin), 25.18 (*C*H_3-BPin), 25.04 (*C*H_3-*anti*-acetonide), 24.51 (*C*H_3-*i*Pr), 24.40 (*C*H_3-*i*Pr), 24.28 (*C*H_3-*i*Pr), 20.34 (*C*H_2-17). Boron-bound *C*H_2-16 is not detected due to quadrupolar relaxation. See spectrum.

IR (neat) ν_{max} (cm^{-1}): 2961, 2934, 2871, 1726 (C=O), 1606 (C=C), 1462, 1379 (B-O), 1319, 1250 (B-C), 1224, 1144, 1076, 1029, 968, 877.

HRMS (ESI) calculated for $C_{33}H_{55}BO_6Na$, $[M+Na]^+$: 581.3990, found: 581.3974.

$[\alpha]_D^{25}$ = –8.0 (c 1.0, CH_2Cl_2)

hex-5-en-1-yl 2,4,6-triisopropylbenzoate (11)

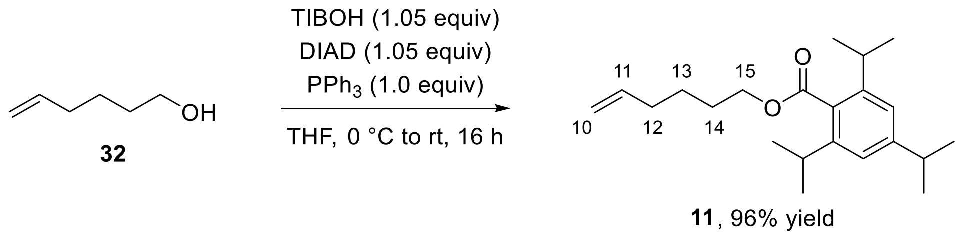

To a solution of alcohol **32** (0.80 g, 8.00 mmol) in THF (0.66 M, 12.00 mL), 2,4,6-triisopropylbenzoic acid TIBOH (2.09 g, 8.40 mmol, 1.05 equiv), PPh_3 (2.10 g, 8.00 mmol, 1.00 equiv) and diisopropyl azodicarboxylate DIAD (1.70 g, 8.40 mmol, 1.05 equiv) were added dropwise at 0 °C and the reaction mixture was stirred at that temperature for 30 min. Then, the reaction mixture was warmed to room temperature and stirred for 16 h. Sat. aq. $NaHCO_3$ (8 mL) was added and the layers were separated. The aqueous phase was extracted with Et_2O (3×40 mL). The combined organics were dried over anhydrous Na_2SO_4, filtered and concentrated under reduced pressure. Pentane was added to the crude residue and the resulting solid was filtered and washed with pentane. The collected pentane was concentrated under reduced pressure and the crude residue was purified by flash column chromatography (SiO_2, pentane: Et_2O 1% to 2%) to afford the title compound **11** (2.54 g, 96% yield) as a colourless oil.

TLC: R_f = 0.4 (pentane: Et_2O 1%, UV visible or stained with $KMnO_4$)

^{1}H NMR (400 MHz, $CDCl_3$) δ 7.05 (s, 2H, *H*-Ar), 5.83 (ddt, *J* = 17.0, 9.9, 6.8 Hz, 1H, *H*-11), 5.05 (dt, *J* = 17.0, 1.8 Hz, 1H, *H*-10), 5.00 (dd, *J* = 9.9, 1.8 Hz, 1H, *H*-10), 4.35 (t, *J* = 6.6 Hz, 2H, *H*-15), 2.91 (m, 3H, *H*-*i*Pr), 2.14 (dt, *J* = 7.2 Hz, 2H, *H*-12), 1.81 – 1.75 (m, 2H *H*-14), 1.60 – 1.54 (m, 2H, *H*-13), 1.28 (dd, J = 7.0, 2.7 Hz, 18H, CH_3-*i*Pr). See spectrum.

^{13}C NMR (101 MHz, $CDCl_3$) d 171.07 (*C*O-benzoate), 150.13 (*C*-*ipso*), 144.83 (*C*-*ortho*), 138.30 (*C*H-11), 130.81 (*C*-*para*), 120.93 (*C*H-Ar), 114.96 (*C*H_2-10), 64.86 (*C*H_2-15), 34.54 (*C*H-*i*Pr *para*), 33.36 (*C*H_2-12), 31.60 (*C*H-*i*Pr *ortho*), 28.19 (*C*H_2-14), 25.43 (*C*H_3-*i*Pr), 24.25 (*C*H_3-*i*Pr), 24.06 (*C*H_2-13). See spectrum.

IR (neat) ν_{max} (cm^{-1}): 2960, 2930, 2769, 1725 (C=O), 1641 (C=C), 1506, 1461, 1432, 1384, 1363, 1283, 1250 (C-O-C), 1137, 1104, 1075, 911, 876.

HRMS (MALDI) calculated for $C_{22}H_{34}NO_2Na$, $[M+Na]^+$: 353.2451, found: 353.2459.

(*R*)-1-((*R*)-*p*-tolylsulfinyl)hex-5-en-1-yl 2,4,6-triisopropylbenzoate (9)

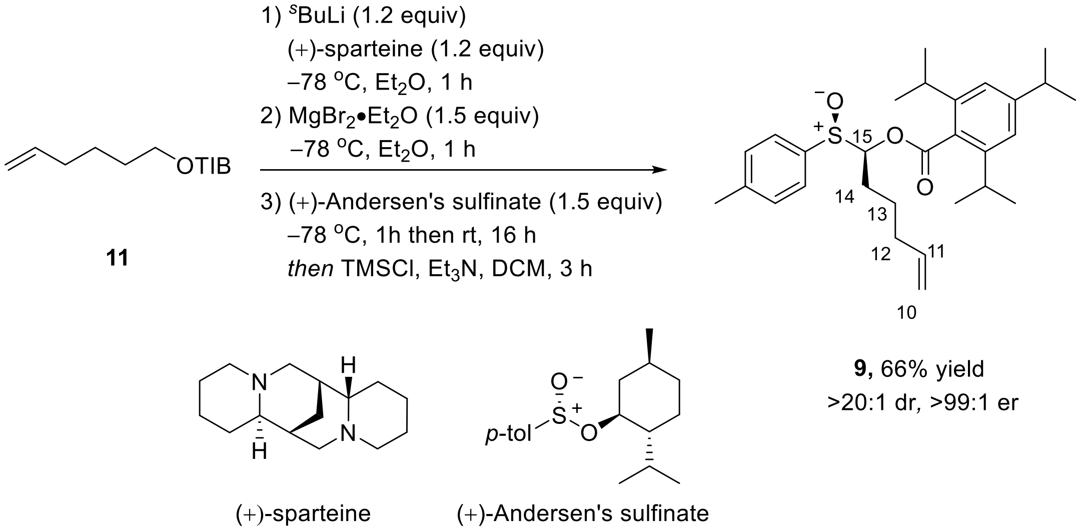

According to the modified literature procedure,[6] (+)-sparteine (2.81 g, 12.00 mmol, 1.20 equiv) was added to a flame dried 3-necked 250 mL flask under nitrogen, followed by the TIB ester **11** (3.30 g, 10.00 mmol). The mixture was degassed by stirring under vacuum for 30 min, then dry diethyl ether (33.00 mL) was added and the reaction mixture was cooled to –78 °C. To the reaction mixture, ^{s}BuLi (1.30 M hexane, 9.23 mL, 12.00 mmol, 1.20 equiv) was added with a syringe pump (1.00 mL/min). The reaction mixture was stirred 1 h at –78 °C (*purple solution*). In a separate flame dried 100 mL 2-necked flask under nitrogen with a condenser, to a stirred suspension of Mg turnings (0.97 g, 40.00 mmol, 4.00 equiv) in Et_2O (18.80 mL), dibromoethane (2.82 g, 15.00 mmol, 1.50 equiv) was added dropwise (*CAUTION: exothermic reaction*). After reflux and gas evolution ceased, the $MgBr_2$ etherate solution was stirred 30 min at room temperature (*formed 2 layers, top colourless, bottom grey*), then it was transferred to the main reaction vessel with the aid of an addition funnel. The reaction mixture was stirred at –78 °C for 2 h (*orange solution with some solid agglomerate*). To this reaction mixture, (+)-(*R*)-Anderson's sulfinate (4.42 g, 15.00 mmol, 1.50 equiv) dissolved in THF (15 mL) was added dropwise with a syringe pump (0.50 mL/min) and the reaction mixture was stirred 1 h at –78 °C (*orange solution turned yellow*), then 16 h at room temperature. The reaction mixture was quenched with 2 M HCl (60 mL), the layers separated, and the organics washed with 2 M HCl (4 x 30 mL). The combined aqueous phase was washed with Et_2O (3 x 60 mL), then the combined organics were washed with saturated $NaHCO_3$ (90 mL) and brine (90 mL), dried over anhydrous Na_2SO_4, filtered and concentrated under reduced pressure.

(+)-*sparteine was recovered from the acid aqueous phase (90% recovery) by basification, extraction with EtOAc, solvent removal and distillation under vacuum.*

To facilitate the chromatographic separation of sulfoxide **9** from menthol, silylation of menthol was performed as follows. The crude mixture was stirred 2 h under vacuum, then dissolved in CH_2Cl_2 (20.0 mL). Triethyl amine (2.1 mL, 15 mmol, 1.5 equiv) was added followed by the dropwise addition of trimethylsilyl chloride TMSCl (1.65 mL, 13.00 mmol, 1.30 equiv) and the mixture was stirred 3 h at room temperature. The reaction mixture was then diluted with Et_2O (20 mL) and water (20 mL), the layers separated and the organics were dried one dried over anhydrous Na_2SO_4, filtered and concentrated under reduced pressure. The crude residue was purified by automated flash column chromatography (Biotage HC-50g, ^{n}Hexane: EtOAc 2-10%, see report below) to afford the title compound **9** (3.10 g, 66% yield, >20:1 dr, ≥99:1 er) as a crystalline white solid.

TLC: R_f = 0.3 (pentane: EtOAc 9:1, stained with PMA)

^{1}H NMR ($CDCl_3$, 400 MHz) d 7.65 (d, *J* = 8.1 Hz, 3H, *H*-tol), 7.37 (d, *J* = 8.0 Hz, 2H, *H*-tol), 7.05 (s, 2H, *H*-Ar), 5.75 – 5.54 (m, 2H, *H*-11 + *H*-15), 4.88 (m, 2H, *H*-10), 2.92 (hept, *J* = 6.8 Hz, 3H, *H*-*i*Pr), 2.44 (s, 3H, CH_3-tol), 2.03 – 1.94 (m, 3H, CH_2), 1.66 – 1.53 (m, 2H, CH_2), 1.40 – 1.33 (m, 1H, CH_2), 1.29 (d, *J* = 6.8 Hz, 6H, CH_3-*i*Pr), 1.27 (d, *J* = 2.8 Hz, 6H, CH_3-*i*Pr), 1.25 (d, *J* = 2.7 Hz, 6H, CH_3-*i*Pr). See spectrum.

^{13}C NMR (101 MHz, 101 MHz, $CDCl_3$) d: 170.63 (*C*O-TIB), 151.01 (*C*-*ipso* TIB), 145.35 (*C*-*ortho* TIB), 141.70 (*C*-tol), 137.72 (*C*-tol), 137.57 (*C*H-11), 130.15 (*C*H-tol), 128.98 (*C*-*para* TIB), 124.53 (*C*H-tol), 121.16 (*C*H-TIB), 115.32 (*C*H-10), 92.52 (*C*H-15), 34.60 (*C*H-*i*Pr *para*), 33.15 (*C*H_2-12), 31.79 (*C*H-*i*Pr *ortho*), 24.54 (*C*H_2-14), 24.34 (*C*H_3-*i*Pr), 24.07 (*C*H_3-*i*Pr), 24.06 (*C*H_3-*i*Pr), 24.05 (*C*H_3-*i*Pr), 22.55 (*C*H_2-13), 21.59 (*C*H_3-tol). See spectrum.

IR (neat) ν_{max} (cm^{-1}): 2961, 2928, 2869, 1731 (C=O), 1606 (C=C), 1460, 1347, 1233, 1089, 1056, 1043 (S=O), 913, 812.

HRMS (ESI) calculated for $C_{29}H_{40}O_3S$, $[M+Na]^+$: 491.2596, found: 491.2592.

$[\alpha]_D^{25}$ = –104.0 (c 1.0, CH_2Cl_2)

m.p.: (^{n}Hexane) 102–104 ºC

Biotage Isolera™ Archive Report - df1-45

**User**	Daniele		
**Sample Name**	df1-45		
**Date**	2020-Dec-15 15.55		
**Method**	df1-29		
**Cartridge**	Sfar Silica HC D 50g	**Detection Mode**	Lambda-all
**Flowrate**	120 ml/min	**Baseline Correction**	On
**Solvent A**	hexane	**UV1 (Monitor)**	254 nm (Red)
**Solvent B**	Ethyl acetate	**UV2 (Monitor)**	280 nm (Black)
		**Lambda-all (Collect)**	(Brown)
**Rack Type**	16x150 mm	**Collect All**	On
**Max Fraction Volume**	20 ml	**Start Threshold**	40 mAU
**Dispense Order**	S		

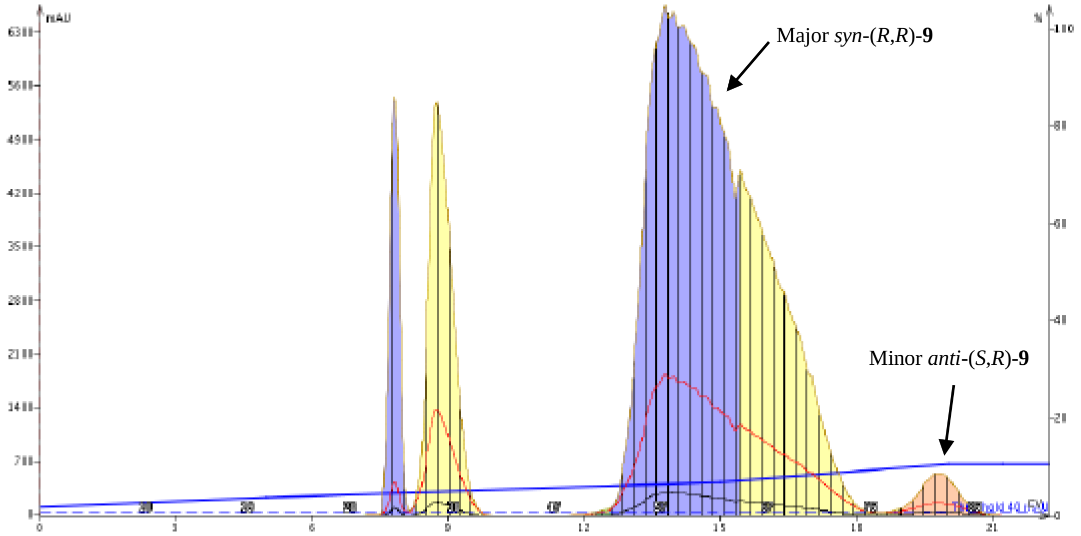

Gradient

	Solvents	Mix	Length (CV)	
Equil.	A/B	1%	3.0	flowrate 120 ml/min
1	A/B	1% - 3%	5.0	
2	A/B	3% - 6%	10.0	
3	A/B	6% - 10%	5.0	
4	A/B	10%	1.2	
5	A/B	10%	1.0	Edit on the fly

Enantiomeric ratio ≥99:1 was determined using HPLC analysis with chiral stationary phase, Daicel Chiralcel-IA column, ^{n}Hex: ^{i}PrOH = 95:5, 0.5 mL/min, λ = 210 nm.
t_R = 11.43 [(*R*), major], 12.99 [(*S*), minor].

The racemic material for HPLC analysis was prepared following the same procedure as above using tetramethylethylendiamine TMEDA instead of (+)-sparteine.

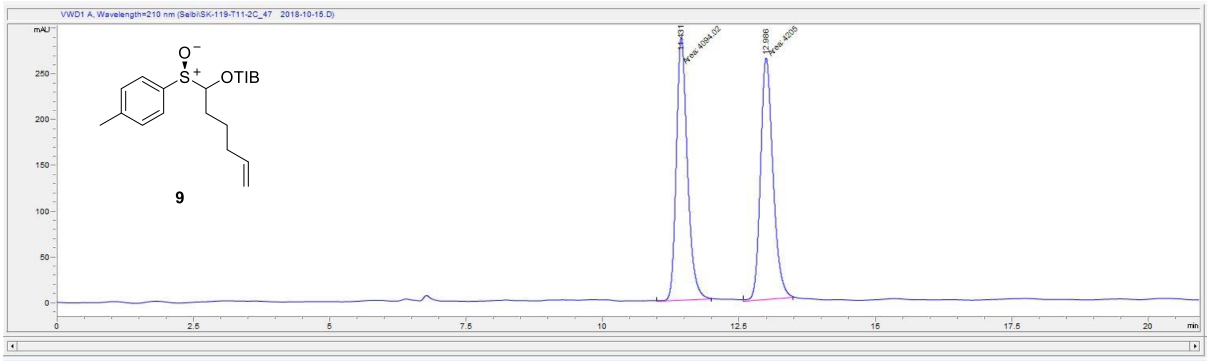

File Information	
LC-File	SK-119-T11-2C_47 2018-10-15.D
File Path	C:\Chem32\1\Data\Selbi
Date	15-Oct-18, 17:53:23
Sample	SK-119-T11-2C
Sample Info	IA guard, 95:5 hexane/IPA, 0.5 mL/min, 0 C, 210 nm,
Barcode	
Operator	SYSTEM
Method	VKA_general.M
Reference	
Analysis Time	20.932 min
Sampling Rate	0.0012 min (0.072 sec), 17252 datapoints

#	Time	Type	Area	Height	Width	Area%	Symmetry
1	11.431	MM	4094	286.4	0.2382	49.331	0.761
2	12.986	MM	4205	263.7	0.2658	50.669	0.794

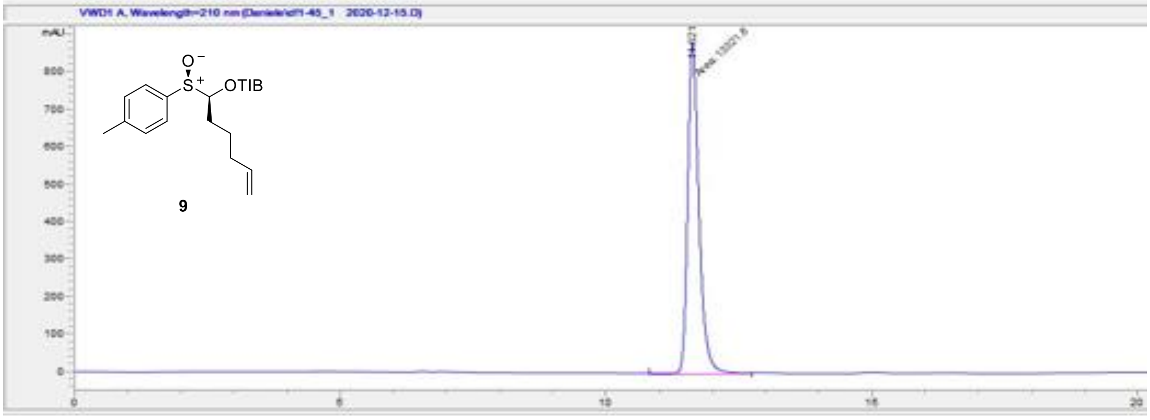

File Information	
LC-File	df1-45_1 2020-12-15.D
File Path	C:\Chem32\1\Data\Daniele
Date	15-Dec-20, 19:01:16
Sample	df1-45
Sample Info	Ia, 95:5 hexane/IPA, 0.5 ml/min, 210 nm.
Barcode	
Operator	SYSTEM
Method	VKA_general_90_10 flush.M
Reference	
Analysis Time	30.137 min
Sampling Rate	0.0012 min (0.072 sec), 24839 datapoints

#	Time	Type	Area	Height	Width	Area%	Symmetry
1	11.621	MM	13321.8	884.6	0.251	100.000	0.736

2-((4*R*,6*R*)-2,2-dimethyl-6-((*S*)-4-(4,4,5,5-tetramethyl-1,3,2-dioxaborolan-2-yl)non-8-en-1-yl)-1,3-dioxan-4-yl)ethyl 2,4,6-triisopropylbenzoate (27)

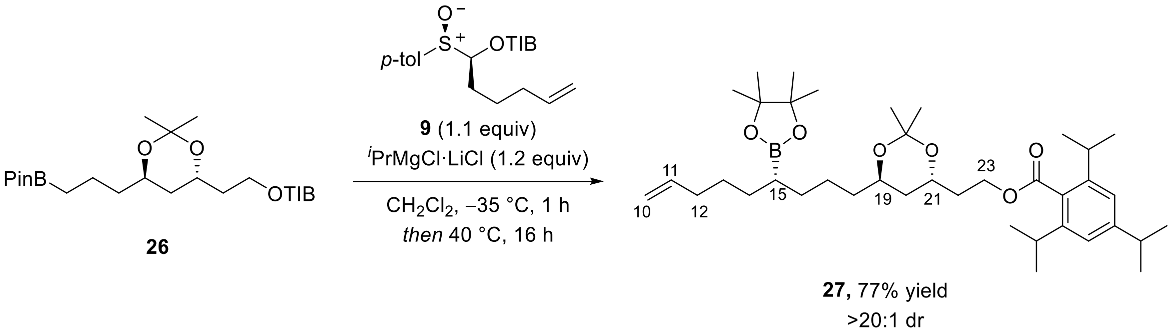

^{i}PrMgCl·LiCl (1.12 M in THF, 1.43 mL, 1.60 mmol, 1.20 equiv) was added dropwise to a mixture of boronic ester **26** (0.74 g, 1.33 mmol, 1.00 equiv) and sulfoxide **9** (0.69 g, 1.46 mmol, 1.10 equiv) in CH_2Cl_2 (6.70 mL, 0.20 M with respect to boronic ester) at –35 °C and the resulting solution was stirred for 1 h at the same temperature (*pale yellow solution*). After warming to room temperature, the reaction mixture was heated at 40 °C for 16 h (*cloudy white solution*). The reaction mixture was then cooled to room temperature and it was quenched with sat. aq. NH_4Cl (10 mL). The aqueous phase was extracted with Et_2O (50×3 mL). The combined organics were dried over anhydrous Na_2SO_4 and filtered over a short pad of SiO_2 deactivated with Et_2O: Et_3N 1% in order to remove the residual TIB acid. The organics were concentrated under reduced pressure and the crude residue was purified by automated flash column chromatography (Biotage HC-25g, ^{n}Hexane: EtOAc 2-10%) to afford the title compound **27** (0.65 g, 77% yield, >20:1 dr) as a colourless oil

TLC: R_f = 0.5 (pentane: EtOAc 8%, stained with *p*-anisaldehyde)

^{1}H NMR (400 MHz, Methylene Chloride-*d*$_2$) d 7.02 (s, 2H, *H*-Ar), 5.81 (ddt, *J* = 16.9, 10.2, 6.7 Hz, 1H, *H*-11), 4.99 (ddt, *J* = 16.9, 2.3, 1.2 Hz, 1H, *H*-10), 4.91 (ddt, *J* = 10.2, 2.3, 1.2 Hz, 1H), 4.40 – 4.29 (m, 2H, *H*-23), 3.95 – 3.88 (m, 1H, *H*-21), 3.78 – 3.73 (m, 1H, *H*-19), 2.89 (hept, *J* = 6.8 Hz, 1H, *H*-*i*Pr), 2.81 (hept, *J* = 6.8 Hz, 2H, *H*-*i*Pr), 2.03 (m, 2H, *H*-12), 1.86 (m, 2H, *H*-22), 1.65 – 1.53 (m, 1H, *H*-20), 1.50 – 1.32 (m, 9H), 1.31 (s, 3H, CH_3-acetonide), 1.29 (s, 3H, CH_3-acetonide), 1.25 (s, 3H, CH_3-BPin), 1.23 (s, 9H, CH_3-BPin), 1.21 (m, 18H, CH_3-*i*Pr), 0.94 – 0.91 (m, 1H, *H*-15). See spectrum.

^{13}C NMR (126 MHz, Methylene Chloride-*d*$_2$) d 171.13 (*C*O-benzoate), 150.89 (*C*-*ipso*), 145.32 (*C*-*ortho*), 139.82 (*C*H-11), 131.31 (*C*-*para*), 121.40 (*C*H-Ar), 114.37 (*C*H_2-10), 100.65 (*C*O *anti*-acetonide), 83.32 (*C*O-BPin), 66.93 (*C*H-19), 64.02 (*C*H-21), 62.32 (*C*H_2-23), 39.15 (*C*H_2-20), 36.75 (*C*H_2-18), 35.49 (*C*H_2-22), 35.02 (*C*H-*i*Pr *para*), 34.63 (*C*H_2-12), 32.04 (*C*H-*i*Pr *ortho*), 31.82 (*C*H_2-17), 31.47 (*C*H_2-13), 29.07 (*C*H_2-14), 25.45 (*C*H_2-16), 25.17 (*C*H_3-BPin), 25.16 (*C*H_3-BPin), 25.01 (*C*H_3-*anti*-acetonide), 24.50 (*C*H_3-*i*Pr), 24.39 (*C*H_3-*i*Pr), 24.27 (*C*H_3-*i*Pr). Boron-bound *C*H-15 is not detected due to quadrupolar relaxation. See spectrum.

IR (neat) ν_{max} (cm^{-1}): 3075 (C=C-H), 2961, 2929, 2869, 1727 (C=O), 1607 (C=C), 1461, 1380 (B-O), 1250 (B-C), 1224, 1143, 1076, 968, 876.

HRMS (ESI) calculated for $C_{39}H_{65}BO_6Na$, $[M+Na]^+$: 663.4773, found: 663.4753.

$[\alpha]_D^{25} = -4.0$ (c 1.0, CH_2Cl_2)

2-((4*R*,6*R*)-2,2-Dimethyl-6-((4*S*,8*S*)-4,8,9-tris(4,4,5,5-tetramethyl-1,3,2-dioxaborolan-2-yl)nonyl)-1,3-dioxan-4-yl)ethyl 2,4,6-triisopropylbenzoate (28)

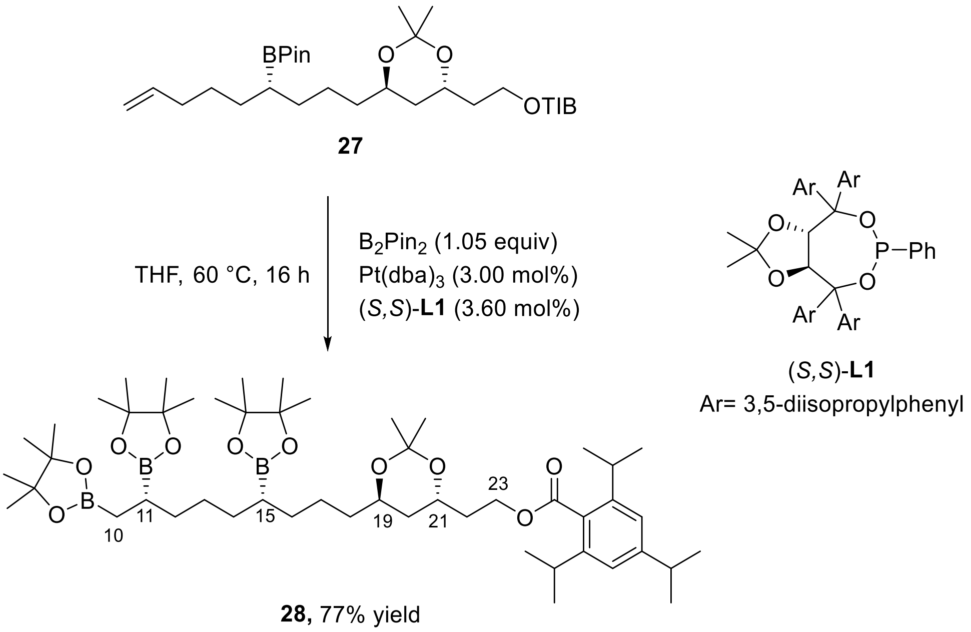

According to the modified literature procedure,[12] to a flame dried Schlenk tube equipped with a magnetic stir bar was added Pt(dba)$_3$ (27 mg, 0.026 mmol, 3.00 mol%), (*S*,*S*)-**L1** (33 mg, 0.036 mmol, 3.60 mol%) and B$_2$Pin$_2$ (267 mg, 1.05 mmol, 1.05 equiv) under a nitrogen atmosphere. After addition of solids, the Schlenk was evacuated and refilled with N$_2$ three times. Then, THF (1.00 mL) was added via syringe. The solution was heated to 80 °C in an oil bath for 30 minutes. The reaction mixture was then cooled to room temperature and alkene **27** (640 mg, 1.00 mmol) was added dissolved in THF (1.00 mL, final concentration 0.50 M). The reaction mixture was stirred overnight at 60 °C. The reaction mixture was then cooled to room temperature and the solvent was evaporated under reduced pressure. The crude mixture was purified by automated flash column chromatography (BIOTAGE HC-25g, pentane: EtOAc 2-22%) to afford the title compound **28** (690 mg, 77% yield, >20:1 dr) as a colourless oil.

TLC: R_f = 0.25 (pentane: EtOAc 9:1, stained with *p*-anisaldehyde)

^{1}H NMR (400 MHz, Methylene Chloride-*d*$_2$) δ 7.02 (s, 2H, *H*-Ar), 4.40 – 4.29 (m, 2H, *H*-23), 3.95 – 3.88 (m, 1H, *H*-21), 3.80 – 3.72 (m, 1H, *H*-19), 2.90 (hept, *J* = 6.8 Hz, 1H, *H*-*i*Pr), 2.81 (hept, *J* = 6.8 Hz, 2H, *H*-*i*Pr), 1.88 – 1.83 (m, 2H, *H*-22), 1.64 – 1.51 (m, 2H, *H*-20), 1.47 – 1.27 (m, 12H), 1.31 (s, 3H, C*H*$_3$-acetonide), 1.29 (s, 3H, C*H*$_3$-acetonide), 1.25 – 1.20 (m, 36H, C*H*$_3$-BPin + 18H, C*H*$_3$-*i*Pr), 1.03 – 0.99 (m, 1H, *H*-15), 0.91 – 0.87 (m, 1H, *H*-11), 0.75 – 0.72 (m, 2H, *H*-10). See spectrum.

^{13}C NMR (126 MHz, Chloroform-*d*) δ 171.07 (*C*O-benzoate), 150.25 (*C*-*ipso*), 144.86 (*C*-*ortho*), 130.69 (*C*-*para*), 120.99 (*C*H-Ar), 100.43 (*C*O *anti*-acetonide), 82.84 (*C*O-BPin), 66.60 (*C*H-19), 63.63 (*C*H-21), 61.93 (*C*H_2-23), 38.84 (*C*H_2-20), 36.35 (*C*H_2-), 35.05 (*C*H_2-), 34.57 (*C*H-*i*Pr *para*), 34.34 (*C*H_2-), 31.80 (*C*H_2-), 31.65 (*C*H-*i*Pr *ortho*), 31.30 (*C*H_2-), 28.63 (*C*H_2-), 25.15 (*C*H_3-BPin), 25.06 (*C*H_3-BPin), 25.00 (*C*H_3-BPin), 24.96 (*C*H_3-BPin), 24.95 (*C*H_3-BPin), 24.93 (*C*H_3-BPin), 24.90 (*C*H_3-*anti*-acetonide), 24.89 (*C*H_3-*anti*-acetonide), 24.80 (*C*H_3-BPin), 24.36 (*C*H_3-*i*Pr), 24.25 (*C*H_3-*i*Pr), 24.10 (*C*H_3-*i*Pr). Boron-bound *C*H-15, *C*H-11 and *C*H_2-10 are not detected due to quadrupolar relaxation. *See spectrum*.

IR (neat) ν_{max} (cm^{-1}): 2975, 2928, 2870, 1727 (C=O), 1607 (C=C), 1462, 1378 (B-O), 1370, 1312, 1251 (B-C), 1222, 1141 (B-C), 968, 877.

HRMS (MALDI) calculated for $C_{51}H_{89}O_{10}B_3Na$, $[M+Na]^+$: 917.6651, found: 917.6635.

$[\alpha]_D^{25}$ = –8.0 (c 1.0, CH_2Cl_2)

2-((4*R*,6*R*)-2,2-Dimethyl-6-((4*S*,8*R*,10*S*)-4,8,10-tris(4,4,5,5-tetramethyl-1,3,2-dioxaborolan-2-yl)pentadec-14-en-1-yl)-1,3-dioxan-4-yl)ethyl 2,4,6-triisopropylbenzoate (7)

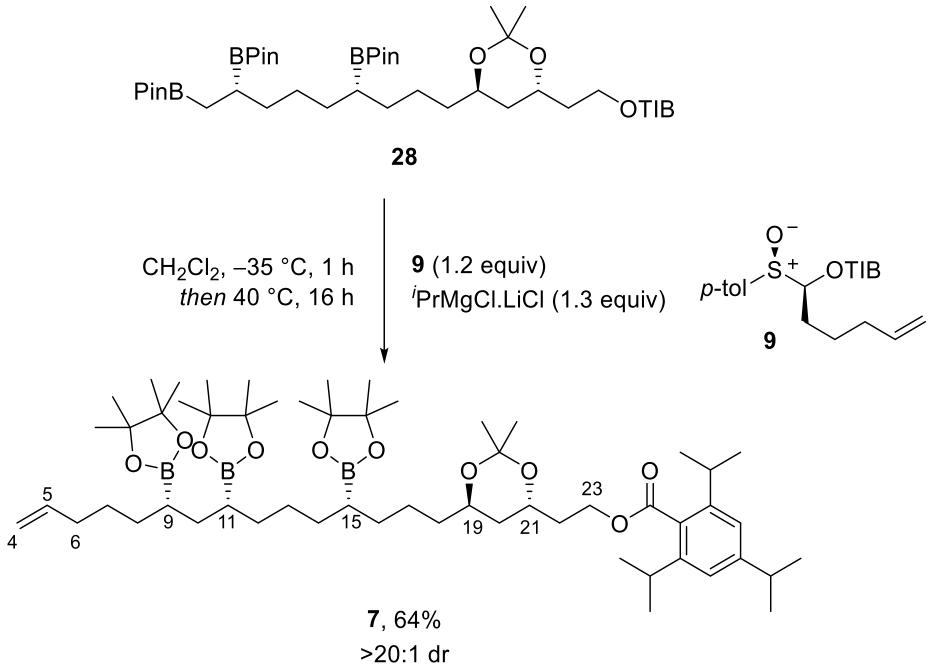

^{i}PrMgCl.LiCl (1.26 M in THF, 0.40 mL, 0.51 mmol, 1.30 equiv) was added dropwise to a mixture of boronic ester **28** (350 mg, 0.39 mmol, 1.00 equiv) and sulfoxide **9** (220 mg, 0.47 mmol, 1.20 equiv) in CH_2Cl_2 (1.95 mL, 0.20 M with respect to boronic ester) at –35 °C and the resulting solution was stirred for 1h at the same temperature (*pale yellow solution*). After warming to room temperature, the reaction mixture was heated at 40 °C for 16 h (*cloudy white suspension*). The reaction mixture was then cooled to room temperature and it was quenched with sat. aq. NH_4Cl (10 mL). The aqueous phase was extracted with Et_2O (50×3 mL). The combined organics were dried over anhydrous Na_2SO_4 and filtered over a short pad of SiO_2 deactivated with Et_2O: Et_3N 1% in order to remove the residual TIB acid. The organics were concentrated under reduced pressure and the crude residue was purified by automated flash column chromatography (Biotage HC-10g, pentane: EtOAc 2-10%) to afford the title compound **7** (220 mg, 64% yield, >20:1 dr) as a colourless oil.

TLC: R_f = 0.40 (pentane: EtOAc 9:1, stained with *p*-anisaldehyde)

^{1}H NMR (500 MHz, Chloroform-*d*) δ 7.00 (s, 2H, *H*-Ar), 5.79 (ddt, J = 16.9, 10.2, 6.6 Hz, 1H, *H*-5), 4.97 (dd, J = 17.2, 1.9 Hz, 1H, *H*-4), 4.90 (dd, J = 10.2, 1.9 Hz, 1H, *H*-4), 4.45 – 4.31 (m, 2H, *H*-23), 3.96 – 3.90 (m, 1H, *H*-21), 3.78 – 3.72 (m, 1H, *H*-21), 2.89 (hept, *J* = 6.8 Hz, 1H, *H*-*i*Pr), 2.83 (hept, J = 6.8 Hz, 2H, *H*-*i*Pr), 2.02 (m, 2H, *H*-6), 1.92 – 1.82 (m, 2H, *H*-22), 1.67 – 1.47 (m, 4H), 1.38 – 1.30 (m, 14H), 1.33 (s, 3H, C*H*$_3$-acetonide), 1.31 (s, 3H, C*H*$_3$-acetonide), 1.24 (m, 12H, C*H*$_3$-BPin), 1.23 (m, 12H, C*H*$_3$-BPin), 1.22 – 1.20 (m, 36H, C*H*$_3$-BPin + 18H, C*H*$_3$-*i*Pr), 1.05 – 1.00 (m, 2H, *H*-9, 15), 0.95 – 0.90 (m, 1H, *H*-11). See spectrum.

^{13}C NMR (126 MHz, Chloroform-*d*) δ 171.06 (*C*O-benzoate), 150.25 (*C*-*ipso*), 144.85 (*C*-*ortho*), 139.39 (*C*H-5), 130.68 (*C*-*para*), 120.98 (*C*H-Ar), 114.12 (CH_2-4), 100.42 (*C*O *anti*-acetonide), 82.86 (*C*O-BPin), 82.84 (*C*O-BPin), 82.79 (*C*O-BPin), 66.60 (*C*H-19), 63.62 (*C*H-21), 61.93 (CH_2-23), 38.84 (CH_2-20), 36.35 (CH_2-), 35.05 (CH_2-), 34.57 (*C*H-*i*Pr *para*), 34.32 (CH_2-), 32.02 (CH_2-), 31.94 (CH_2-), 31.65 (*C*H-*i*Pr *ortho*), 31.56 (CH_2-), 31.29 (CH_2-), 30.77 (CH_2-), 30.46 (CH_2-), 29.02 (CH_2-), 28.77 (CH_2-), 25.15 (CH_3-BPin), 24.98 (CH_3-BPin), 24.96 (CH_3-BPin), 24.94 (CH_3-BPin), 24.92 (CH_3-*anti*-acetonide), 24.88 (CH_3-*anti*-acetonide), 24.79 (CH_3-BPin), 24.35 (CH_3-*i*Pr), 24.25 (CH_3-*i*Pr), 24.10 (CH_3-*i*Pr), 22.84 (*C*H-B). *See spectrum*.

IR (neat) ν_{max} (cm^{-1}): 2975, 2926, 2856, 1726 (C=O), 1606 (C=C), 1461, 1379 (B-O), 1370, 1312, 1251 (B-C), 1222, 1141 (B-C), 1075, 968, 858.

HRMS (ESI) calculated for $C_{57}H_{99}O_{10}B_3Na$, $[M+Na]^+$: 999.7435, found: 999.7432.

$[\alpha]_D^{25}$ = –4.0 (c 1.0, CH_2Cl_2)

2-((4*R*,6*R*)-2,2-Dimethyl-6-((4*S*,8*R*,10*S*)-4,8,10-trihydroxypentadec-14-en-1-yl)-1,3-dioxan-4-yl)ethyl 2,4,6-triisopropylbenzoate (29)

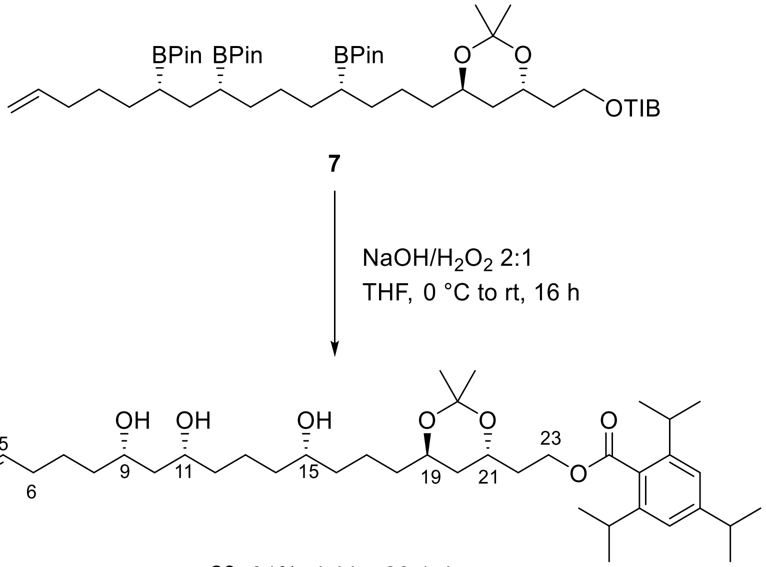

29, 91% yield, >20:1 dr

To a solution of boronic ester **7** (210 mg, 0.215 mmol) in THF (2.00 mL, 0.10 M with respect to boronic ester), a preformed solution of NaOH 3.00 M (1.20 mL) and H_2O_2 30% v/v (0.60 mL) was added dropwise at 0 °C. The reaction mixture was vigorously stirred 16 h at room temperature. Sat. aq. $Na_2S_2O_3$ (2 mL) was added dropwise at 0 °C under stirring and the layers were separated. The aqueous phase was extracted with Et_2O (3×5 mL). The combined organics were dried over anhydrous Na_2SO_4, filtered and concentrated under reduced pressure. The crude mixture was purified by flash column chromatography (SiO_2, CH_2Cl_2: MeOH 10%) to afford the title compound **29** (127 mg, 91% yield, >20:1 dr) as a colourless oil.

TLC: R_f = 0.30 (CH_2Cl_2: MeOH 5%, stained with *p*-anisaldehyde)

^{1}H NMR (500 MHz, Chloroform-*d*) δ 7.00 (s, 2H, *H*-Ar), 5.80 (ddt, J = 16.9, 10.3, 6.7 Hz, 1H, *H*-5), 5.01 (dd, J = 17.1, 1.8 Hz, 1H, *H*-4), 4.96 (dd, J = 10.3, 1.8 Hz, 1H, *H*-4), 4.47 – 4.31 (m, 2H, *H*-23), 3.94 (m, 1H, *H*-21), 3.87 (m, 2H, *H*-9, *H*-11), 3.78 (m, 1H, *H*-19), 3.61 (m, 1H, *H*-15), 3.14 (bs, 1H, O*H*), 2.90 (bs, 1H, O*H*), 2.88 (hept, *J* = 6.8 Hz, 1H, *H*-*i*Pr), 2.83 (hept, *J* = 6.8 Hz, 1H, *H*-*i*Pr), 2.07 (m, 2H, *H*-6), 1.96 – 1.83 (m, 2H, *H*-22), 1.68 (d, *J* = 4.4 Hz, 1H, O*H*-15), 1.64 – 1.38 (m, 20H), 1.35 (s, 3H, C*H*$_3$-acetonide), 1.33 (s, 3H, C*H*$_3$-acetonide), 1.24 (d, J = 6.8 Hz, 18H, C*H*$_3$-*i*Pr). See spectrum.

^{13}C NMR (126 MHz, Chloroform-*d*) δ 171.08 (*C*O-benzoate), 150.30 (*C*-*ipso*), 144.86 (*C*-*ortho*), 138.71 (*C*H-5), 130.64 (*C*-*para*), 121.00 (*C*H-Ar), 114.87 (*C*H$_2$-4), 100.56 (*C*O *anti*-acetonide), 73.19 (*C*H-9 or *C*H-11), 73.14 (*C*H-9 or *C*H-11), 71.78 (*C*H-15), 66.76 (*C*H-19), 63.65 (*C*H-21), 61.85 (*C*H$_2$-23), 43.03 (*C*H$_2$-10), 38.81 (*C*H$_2$-20), 38.18 (*C*H$_2$-), 37.84 (*C*H$_2$-), 37.45 (*C*H$_2$-), 37.38 (*C*H$_2$-), 35.84

(*C*H_2-), 35.02 (*C*H_2-22), 34.57 (*C*H-*i*Pr *para*), 33.78 (*C*H_2-6), 31.67 (*C*H-*i*Pr *ortho*), 24.85 (*C*H_3-*anti*-acetonide), 24.82 (*C*H_3-*anti*-acetonide), 24.72 (*C*H_2-), 24.36 (*C*H_3-*i*Pr), 24.25 (*C*H_3-*i*Pr), 24.10 (*C*H_3-*i*Pr), 21.83 (*C*H_2-), 21.43 (*C*H_2-). See spectrum.

IR (neat) ν_{max} (cm^{-1}): 3356 (O-H), 3076 (C=C-H), 2959, 2932, 2868, 1725 (C=O), 1607 (C=C), 1460, 1380 1363, 1251, 1223, 1135, 1104, 1075, 907, 877.

HRMS (ESI) calculated for $C_{39}H_{66}O_7Na$, $[M+Na]^+$: 669.4701, found: 669.4669.

$[\alpha]_D^{25}$ = –4.0 (c 1.0, CH_2Cl_2)

2-((4*R*,6*R*)-2,2-Dimethyl-6-((4*R*,8*R*,10*S*)-4,8,10-tris((triethylsilyl)oxy)pentadec-14-en-1-yl)-1,3-dioxan-4-yl)ethyl 2,4,6-triisopropylbenzoate (5, Fragment B)

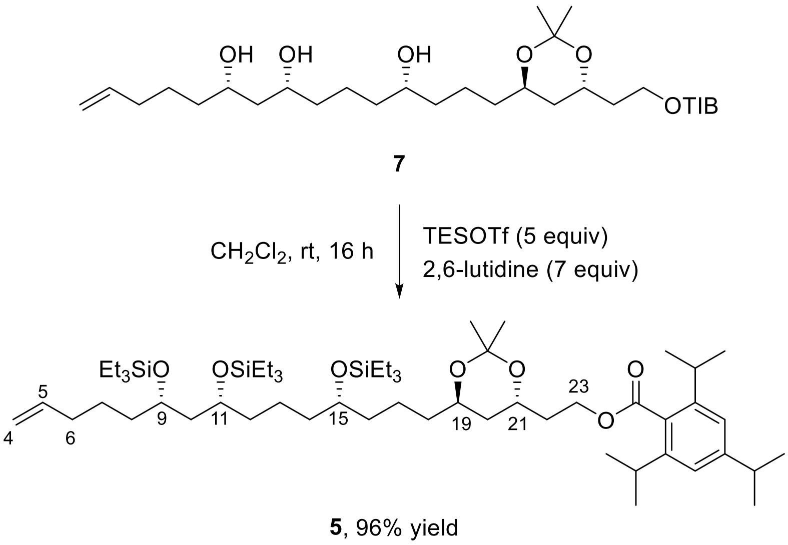

To a solution of alcohol **7** (100 mg, 0.155 mmol) in CH_2Cl_2 (1.55 mL, 0.10 M), 2,6-lutidine (0.126 mL, 1.09 mmol, 7.00 equiv) was added dropwise at room temperature. To the reaction mixture, triethylsilyl trifluoromethanesulfonate TESOTf (0.175 mL, 0.76 mmol, 5.00 equiv) was added dropwise at 0 °C. The reaction mixture was stirred 16 h at room temperature. Water (1 mL) was added and the layers were separated. The aqueous phase was extracted with Et_2O (3×5 mL). The combined organics were dried over anhydrous Na_2SO_4, filtered and concentrated under reduced pressure. The crude mixture was purified by flash column chromatography (SiO_2, pentane: Et_2O 95:5) to afford the title compound **5** (147 mg, 96% yield, >20:1 dr) as a colourless oil.

TLC: R_f = 0.40 (pentane: EtOAc 4%, stained with *p*-anisaldehyde)

^{1}H NMR (500 MHz, Chloroform-*d*) δ 7.00 (s, 2H, *H*-Ar), 5.80 (ddt, J = 16.9, 10.2, 6.6 Hz, 1H, *H*-5), 5.00 (dd, J = 17.1, 1.8 Hz, 1H, *H*-4), 4.95 (dd, J = 10.2, 1.8 Hz, 1H, *H*-4), 4.46 – 4.31 (m, 2H, *H*-23), 3.94 (m, 1H, *H*-21), 3.78 – 3.73 (m, 2H, *H*-9, *H*-11, *H*-19), 3.62 (m, 1H, *H*-15), 2.89 (hept, *J* = 6.8 Hz, 1H, *H*-*i*Pr), 2.84 (hept, *J* = 6.8 Hz, 1H, *H*-*i*Pr), 2.04 (m, 2H, *H*-6), 1.91 – 1.85 (m, 2H, *H*-22), 1.65 – 1.57 (m, 4H), 1.54 – 1.34 (m, 16H), 1.34 (s, 3H, C*H*3-acetonide), 1.33 (s, 3H, C*H*3-acetonide), 1.24 (d, J = 7.0 Hz, 18H, C*H*3-*i*Pr), 0.95 (m, 27H, C*H*3-OTES), 0.67 – 0.55 (m, 18H, C*H*2-OTES). See spectrum.

^{13}C NMR (126 MHz, Chloroform-*d*) δ 171.07 (*C*O-benzoate), 150.28 (*C*-*ipso*), 144.86 (*C*-*ortho*), 139.03 (*C*H-5), 130.67 (*C*-*para*), 121.00 (*C*H-Ar), 114.56 (*C*H2-4), 100.50 (*C*O *anti*-acetonide), 72.52 (*C*H-15), 69.77 (*C*H-9 or *C*H-11), 69.69 (*C*H-9 or *C*H-11), 66.71 (*C*H-19), 63.63 (*C*H-21), 61.89 (*C*H2-23), 45.17 (*C*H2-10), 38.89 (*C*H2-20), 38.00 (*C*H2-), 37.89 (*C*H2-), 37.21 (*C*H2-), 36.96 (*C*H2-), 36.25 (*C*H2-), 35.05 (*C*H2-), 34.57 (*C*H-*i*Pr *para*), 34.11 (*C*H2-6), 31.66 (*C*H-*i*Pr *ortho*), 24.84 (*C*H3-*anti*-acetonide), 24.71 (*C*H3-*anti*-acetonide), 24.59 (*C*H2-), 24.35 (*C*H3-*i*Pr), 24.24 (*C*H3-*i*Pr), 24.09 (*C*H3-

*i*Pr), 21.62 (*C*H_2-), 21.18 (*C*H_2-), 7.11 (*C*H_3CH_2Si), 5.32 (CH_3*C*H_2Si), 5.29 (CH_3*C*H_2Si), 5.26 (CH_3*C*H_2Si). *See spectrum*.

IR (neat) ν_{max} (cm^{-1}): 2954, 2937, 2875, 1728 (C=O), 1607 (C=C), 1459, 1379, 1239, 1224, 1102, 1075, 1005 (Si-O), 876, 724 (Si-C), 671.

HRMS (ESI) calculated for $C_{57}H_{108}O_7Si_3Na$, $[M+Na]^+$: 1011.729506, found: 1011.725581.

$[\alpha]_D^{25}$ = –4.0 (c 1.0, CH_2Cl_2)

(2*R*,6*R*,10*R*,14*R*,18*S*)-1-((4*R*,6*R*)-2,2-Dimethyl-6-((4*R*,8*R*,10*S*)-4,8,10-tris((triethylsilyl)oxy)pentadec-14-en-1-yl)-1,3-dioxan-4-yl)-19,19-dimethyl-6,10,14,18-tetrakis((triethylsilyl)oxy)icosan-2-ol (2)

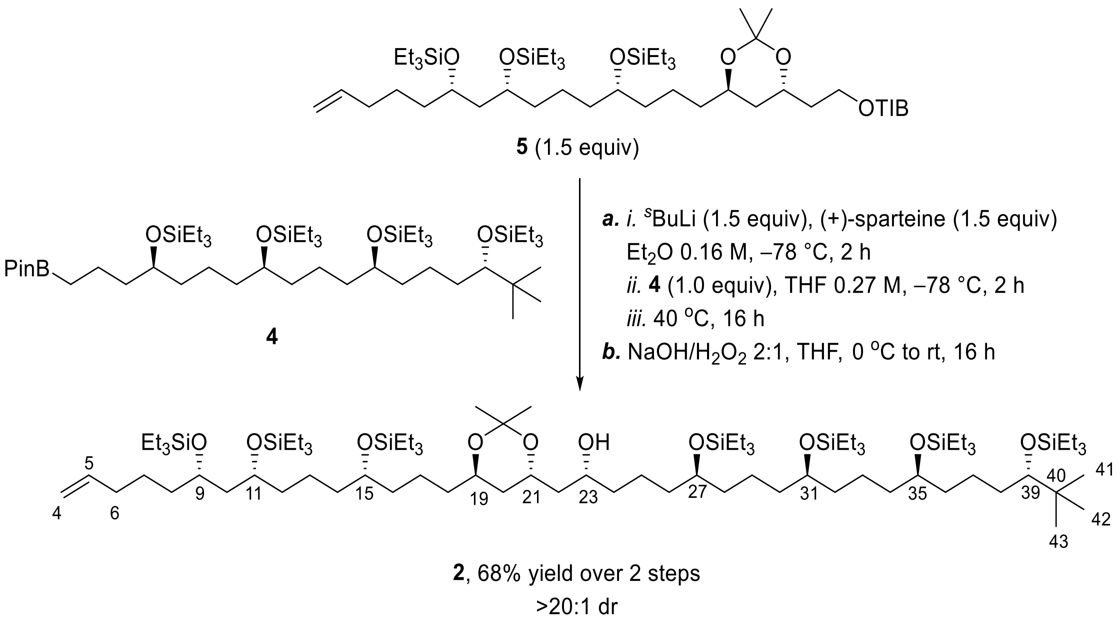

To a solution of TIB ester **5** (76 mg, 0.11 mmol, 1.50 equiv) and (+)-sparteine (26 mg, 0.11 mmol, 1.50 equiv) in diethyl ether (0.80 mL, 0.16 M), ^{s}BuLi (1.30 M solution in heptane, 0.085 mL, 0.11 mmol, 1.50 equiv) was added dropwise at –78 °C and the reaction mixture was stirred at same temperature for 2 h. Boronic ester **4** (70 mg, 0.075 mmol, 1.00 equiv) was rapidly added as a solution in THF (0.27 mL, 0.27 M) to the reaction mixture at –78 °C, and the reaction mixture was stirred for 2 h at –78 °C and then it was warmed up to room temperature and stirred 16 hour at 40 °C. The reaction was quenched by the addition of saturated aqueous NH_4Cl (1 mL) and extracted with Et_2O (3x5 mL). The organics were concentrated under reduced pressure and the crude residue was directly engaged in the oxidation step. To a solution of crude residue (0.11 mmol) in THF (1.1 mL, 0.1 M with respect to boronic ester), a preformed solution of NaOH 3 M (0.40 mL) and H_2O_2 30% v/v (0.20 mL) was added dropwise at 0 °C. The reaction mixture was vigorously stirred 16 h at room temperature. Sat. aq. $Na_2S_2O_3$ (2 mL) was added dropwise at 0 °C under stirring and the layers were separated. The aqueous phase was extracted with Et_2O (3×5 mL). The combined organics were dried over anhydrous Na_2SO_4, filtered and concentrated under reduced pressure. The crude mixture was purified by flash column chromatography (SiO_2, pentane: EtOAc 4-5%) to afford the title compound **2** (80 mg, 68% yield over two steps, >20:1 dr) as a colourless oil and recovered TIB ester **5** (35 mg).

TLC: R_f = 0.18 (pentane: EtOAc 4%, stained with *p*-anisaldehyde)

^{1}H NMR (500 MHz, Chloroform-*d*) δ 5.80 (ddt, *J* = 16.9, 10.2, 6.6 Hz, 1H, *H*-5), 5.00 (dt, J = 17.1, 1.8 Hz, 1H, *H*-4), 4.95 (ddt, J = 10.2, 2.4, 1.2 Hz, 1H, *H*-4), 4.07 – 4.02 (m, 1H, *H*-23), 3.79 – 3.73 (m, 4H,

C*H*-), 3.68 – 3.59 (m, 4H, C*H*-), 3.52 (s, 1H, O-*H*), 3.24 (d, *J* = 6.2 Hz, 1H, *H*-39), 2.04 (dt, *J* = 6.8 Hz, 2H, *H*-6), 1.66 – 1.57 (m, 4H, CH_2-), 1.48 – 1.35 (m, 36H, CH_2- + s, 3H, CH_3-acetonide), 1.34 (s, 3H, CH_3-acetonide), 1.27 (m, 6H, CH_2-), 0.98 – 0.94 (m, 63H, CH_3-OTES), 0.84 (s, 9H, *H*-41, 42, 43), 0.64 – 0.54 (m, 42H, CH_2-OTES). *See spectrum*.

^{13}C NMR (126 MHz, Chloroform-*d*) δ 139.03 (*C*H-5), 114.57 (*C*H_2-4), 100.68 (*C*O *anti*-acetonide), 81.44 (*C*H-39), 72.64 (*C*H-35), 72.53 (*C*H-31), 72.51 (*C*H-27), 72.27 (*C*H-15), 69.77 (*C*H-9 or *C*H-11), 69.69 (*C*H-9 or *C*H-11), 68.42 (*C*H-23), 66.71 (*C*H-19 + *C*H-21), 45.17 (*C*H_2-10), 42.54 (*C*H_2-), 39.16 (*C*H_2-), 38.02 (*C*H_2-), 37.99 (*C*H_2-), 37.96 (*C*H_2-), 37.91 (*C*H_2-), 37.74 (*C*H_2-), 37.72 (*C*H_2-), 37.66 (*C*H_2-), 37.62 (*C*H_2-), 37.50 (*C*H_2-), 37.19 (*C*H_2-), 36.96 (*C*H_2-), 36.18 (*C*H_2-), 35.84 (*C*-40), 34.11 (*C*H_2-6), 33.70 (*C*H_2-38), 26.46 (*C*H_3-41, 42, 43), 25.08 (*C*H_3-*anti*-acetonide), 24.73 (*C*H_3-*anti*-acetonide), 24.60 (*C*H_2-), 23.85 (*C*H_2-), 21.59 (*C*H_2-), 21.48 (*C*H_2-), 21.44 (*C*H_2-), 21.36 (*C*H_2-), 21.17 (*C*H_2-), 7.32 (*C*H_3CH_2Si), 7.14 (*C*H_3CH_2Si), 7.13 (*C*H_3CH_2Si), 7.11 (*C*H_3CH_2Si), 5.82 (CH_3*C*H_2Si), 5.32 (CH_3*C*H_2Si), 5.31 (CH_3*C*H_2Si), 5.29 (CH_3*C*H_2Si), 5.27 (CH_3*C*H_2Si), 5.26 (CH_3*C*H_2Si). *See spectrum*.

IR (neat) n_{max} (cm^{-1}): 3535 (O-H), 2951, 2937, 2875, 2911, 1458, 1415, 1378, 1237, 1095, 1044, 1004 (Si-O), 907, 801, 721 (Si-C), 671.

HRMS (ESI) calculated for $C_{85}H_{183}O_{10}Si_7$, $[M+H]^+$: 1560.2196, found: 1560.2191.

$[\alpha]_D^{25}$ = –12.0 (c 1.0, CH_2Cl_2)

(2*R*,6*R*,10*R*,14*R*,18*S*)-1-((4*S*,6*R*)-2,2-Dimethyl-6-((4*R*,8*R*,10*S*)-4,8,10-tris((triethylsilyl)oxy)pentadec-14-en-1-yl)-1,3-dioxan-4-yl)-19,19-dimethyl-6,10,14,18-tetrakis((triethylsilyl)oxy)icosan-2-yl (*Z*)-3-iodobut-2-enoate (34)

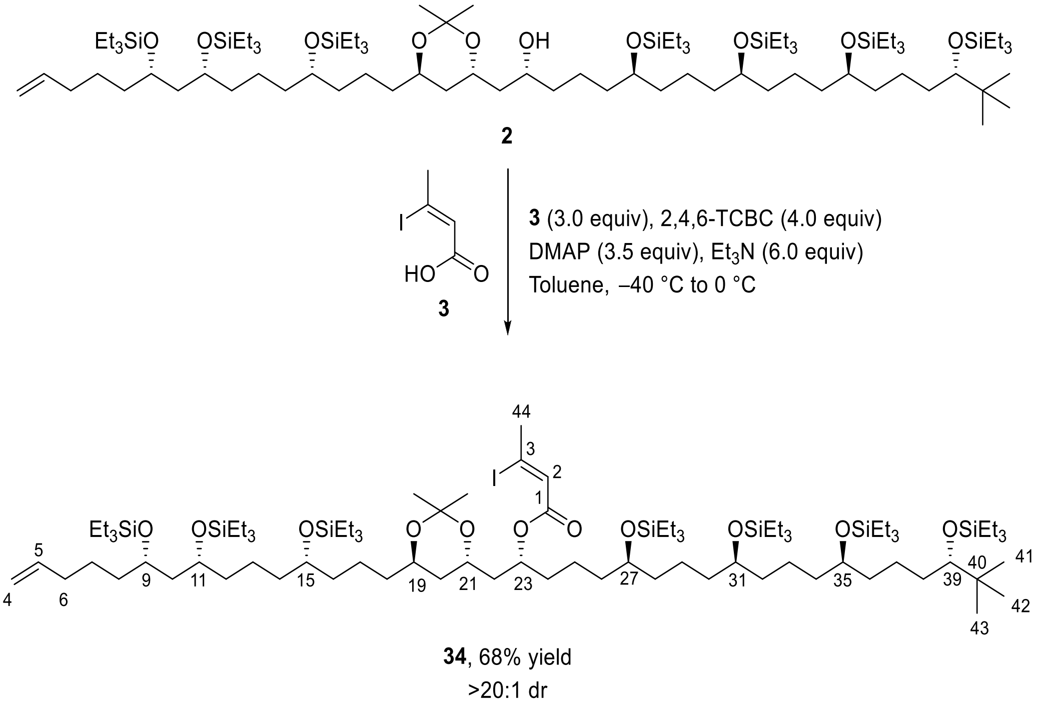

According to a modified procedure,[14] to a solution of alcohol **2** (60 mg, 0.38 mmol, 1.00 equiv) in toluene (0.60 mL), triethylamine (0.032 mL, 0.23 mmol, 6.00 equiv) and acid **3** (24 mg, 0.11 mmol, 3.00 equiv) were added as a solution in toluene (0.25 mL). The reaction mixture was cooled to –40 °C. At this temperature, 4-dimethylamino pyridine (16 mg) was added as a solution in toluene (0.25 mL), followed by 2,4,6-trichlorobenzoyl chloride (37 mg, 0.15 mmol, 4.00 equiv) dissolved in toluene (0.25 mL). The resulting reaction mixture (*white suspension*) was stirred 1 h at –40 °C, then 1 h at –20 °C, then 1 h at 0 °C (*brown solution*). The reaction mixture was quenched by the addition of saturated aqueous $NaHCO_3$ (1 mL) and extracted with EtOAc (3x5 mL). The combined organics were dried over anhydrous Na_2SO_4, filtered and concentrated under reduced pressure. The crude mixture was purified by flash column chromatography (SiO_2, pentane: EtOAc 2% to 6%) to afford the title compound **34** (45 mg, 68% yield, >20:1 dr) as a colourless oil.

TLC: R_f = 0.50 (pentane: EtOAc 4%, stained with *p*-anisaldehyde)

^{1}H NMR (500 MHz, Chloroform-*d*) δ 6.26 (d, *J* = 1.4 Hz, 1H, *H*-2), 5.80 (ddt, J = 16.9, 10.2, 6.6 Hz, 1H, *H*-5), 5.14 – 5.09 (m, 1H, *H*-23), 5.00 (dt, *J* = 17.1, 1.7 Hz, 1H, *H*-4), 4.95 (ddt, J = 10.2, 2.4, 1.2 Hz, 1H, *H*-4), 3.88 (ddd, J = 14.2, 8.5, 6.2 Hz, 1H, C*H*-), 3.79 – 3.70 (m, 3H, C*H*-), 3.66 – 3.57 (m, 4H, C*H*-), 3.24 (d, *J* = 6.2 Hz, 1H, *H*-39), 2.73 (d, *J* = 1.4 Hz, 3H, *H*-44), 2.04 (dt, *J* = 6.5 Hz, 2H, *H*-6),

1.89 (dt, J = 14.9, 7.7 Hz, 1H, C*H*$_2$-), 1.70 – 1.50 (m, 6H, C*H*$_2$-), 1.51 – 1.31 (m, 33H, C*H*$_2$-), 1.31 (s, 3H, C*H*$_3$-acetonide), 1.30 (s, 3H, C*H*$_3$-acetonide), 1.30 – 1.24 (m, 6H, C*H*$_2$-), 0.98 – 0.93 (m, 63H, C*H*$_3$-OTES), 0.84 (s, 9H, *H*-41, 42, 43), 0.64 – 0.55 (m, 42H, C*H*$_2$-OTES). See spectrum.

^{13}C NMR (126 MHz, Chloroform-*d*) δ 163.84 (*C*-1), 139.05 (*C*H-5), 126.00 (*C*H-2), 114.56 (*C*H$_2$-4), 113.09 (*C*-3), 100.37 (*C*O *anti*-acetonide), 81.44 (*C*H-39), 72.64 (*C*H-35), 72.56 (*C*H-31), 72.52 (*C*H-27), 72.31 (*C*H-15), 71.85 (*C*H-23), 69.78 (*C*H-9 or *C*H-11), 69.69 (*C*H-9 or *C*H-11), 66.66 (*C*H-19), 64.12 (*C*H-21), 45.19 (*C*H$_2$-10), 40.18 (*C*H$_2$-), 39.07 (*C*H$_2$-), 38.02 (*C*H$_2$-), 37.91 (*C*H$_2$-), 37.75 (*C*H$_2$-), 37.73 (*C*H$_2$-), 37.65 (*C*H$_2$-), 37.26 (*C*H$_2$-), 36.96 (*C*H$_2$-), 36.66 (*C*H$_3$-44), 36.20 (*C*H$_2$-), 35.84 (*C*-40), 34.58 (*C*H$_2$-), 34.12 (*C*H$_2$-6), 33.70 (*C*H$_2$-38), 26.46 (*C*H$_3$-41, 42, 43), 24.87 (*C*H$_3$-*anti*-acetonide), 24.74 (*C*H$_3$-*anti*-acetonide), 24.60 (*C*H$_2$-), 23.85 (*C*H$_2$-), 21.72 (*C*H$_2$-), 21.51 (*C*H$_2$-), 21.35 (*C*H$_2$-), 21.20 (*C*H$_2$-), 21.04 (*C*H$_2$-), 7.33 (*C*H$_3$CH$_2$Si), 7.15 (*C*H$_3$CH$_2$Si), 7.14 (*C*H$_3$CH$_2$Si), 7.13 (*C*H$_3$CH$_2$Si), 7.12 (*C*H$_3$CH$_2$Si), 5.82 (CH$_3$*C*H$_2$Si), 5.33 (CH$_3$*C*H$_2$Si), 5.31 (CH$_3$*C*H$_2$Si), 5.30 (CH$_3$*C*H$_2$Si), 5.28 (CH$_3$*C*H$_2$Si), 5.27 (CH$_3$*C*H$_2$Si). See spectrum.

IR (neat) ν_{max} (cm^{-1}): 2953, 2936, 2876, 1730 (C=O), 1628 (C=C), 1459, 1378, 1238, 1174, 1111, 1008 (Si-O), 740 (Si-C), 670.

HRMS (MALDI) calculated for $C_{89}H_{185}O_{11}Si_7INa$, [M+Na]$^+$: 1777.1263, found: 1777.1268.

$[\alpha]_D^{25}$ = –12.0 (c 0.33, CH_2Cl_2)

Protected bastimolide B (35)

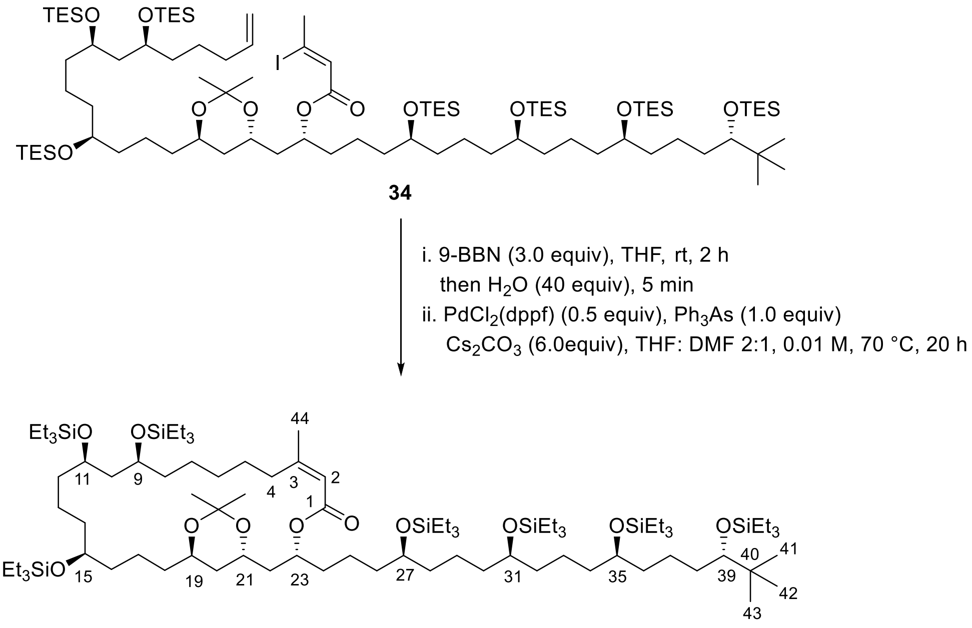

35, 42% yield

To a solution of alkene **34** (5.0 mg, 0.0029 mmol, 1.00 equiv) in dry and degassed THF (0.06 mL, 0.05 M), 9-BBN (0.50 M THF solution, 0.0087 mmol, 17 μL) was added and the reaction mixture was stirred 2 h at room temperature. To the reaction mixture, distilled and degassed H_2O (2 μL) was added and the reaction mixture was stirred 5 min, then dry and degassed THF (0.30 mL) was added. The resulting solution was added dropwise over 5 minutes to a mixture of $PdCl_2$(dppf) (1.1 mg, 0.0015 mmol, 0.5 equiv), Ph_3As (0.9 mg, 0.0029 mmol, 1.0 equiv), Cs_2CO_3 (6.0 mg, 0.057 mmol, 6.0 equiv) in a dry and degassed mixture of THF: DMF 10:1 (1.0 mL) at 70 °C. The resulting reaction mixture was stirred 20 h at 70 °C, then it was quenched at room temperature by addition of sodium phosphate buffer solution (1.0 mL) and H_2O_2 30% (0.5 mL). The resulting mixture was stirred at room temperature 30 min, then a 1:1 mixture of ^{n}Hexane: Et_2O (5mL) was added and the resulting mixture stirred 10 min. The phases were separated and the aqueous phase was extracted with a 1:1 mixture of ^{n}Hexane: Et_2O (3x5mL). The combined organics were dried over anhydrous Na_2SO_4, filtered and concentrated under reduced pressure. The crude mixture was purified by flash column chromatography (SiO_2, pentane: Et_2O 8%) to afford the title compound **35** (2.0 mg, 42% yield, >20:1 *Z*/*E*, >20:1 dr) as a waxy solid.

TLC: R_f = 0.34 (pentane: EtOAc 4%, stained with *p*-anisaldehyde) or 0.44 (pentane: Et_2O 10%, stained with *p*-anisaldehyde)

^{1}H NMR (500 MHz, Chloroform-*d*) δ 5.63 (d, *J* = 1.4 Hz, 1H, *H*-2), 5.06 – 4.99 (m, 1H, *H*-23), 3.83 – 3.73 (m, 3H, C*H*-), 3.72 – 3.57 (m, 5H, C*H*-), 3.24 (d, *J* = 6.2 Hz, 1H, *H*-39), 3.21 – 3.12 (m, 1H, *H*-4), 2.13 – 2.03 (m, 1H, *H*-4), 1.88 (d, *J* = 1.4 Hz, 3H, *H*-44), 1.74 – 1.65 (m, 2H), 1.50 – 1.33 (m, 40H), 1.31 (s, 3H, C*H*$_3$-acetonide), 1.30 (s, 3H, C*H*$_3$-acetonide), 1.28 – 1.24 (m, 8H), 1.00 – 0.92 (m, 63H, C*H*$_3$-OTES), 0.84 (s, 9H, *H*-41, 42, 43), 0.64 – 0.53 (m, 42H, C*H*$_2$-OTES). *See spectrum*.

^{13}C NMR (126 MHz, Chloroform-*d*) δ 165.85 (*C*-1), 160.90 (*C*-3), 116.20 (*C*H-2), 100.12 (*C*O *anti*-acetonide), 81.44 (*C*H-39), 72.64 (*C*H-35), 72.52 (*C*H-), 72.34 (*C*H-), 71.45 (*C*H-), 70.22 (*C*H-), 69.35 (*C*H-), 69.33 (*C*H-), 66.70 (*C*H-19), 64.05 (*C*H-21), 44.12 (*C*H$_2$-), 40.74 (*C*H$_2$-), 38.39 (*C*H$_2$-), 38.01 (*C*H$_2$-), 37.85 (*C*H$_2$-), 37.74 (*C*H$_2$-), 37.71 (*C*H$_2$-), 37.64 (*C*H$_2$-), 37.62 (*C*H$_2$-), 37.26 (*C*H$_2$-), 36.79 (*C*H$_2$-), 36.41 (*C*H$_2$-), 36.29 (*C*H$_2$-), 36.13 (*C*H$_2$-), 35.83 (*C*-40), 35.51 (*C*H$_2$-), 33.69 (*C*H$_2$-38), 32.03 (*C*H$_2$-), 30.23 (*C*H$_2$-), 29.85 (*C*H$_2$-), 29.17 (*C*H$_2$-), 28.62 (*C*H$_2$-), 26.46 (*C*H$_3$-41, 42, 43), 25.72 (*C*H$_2$-), 25.60 (*C*H$_3$-*anti*-acetonide), 25.14 (*C*H$_3$-*anti*-acetonide), 24.93 (*C*H$_3$-44), 23.84 (*C*H$_2$-), 21.51 (*C*H$_2$-), 21.42 (*C*H$_2$-), 21.35 (*C*H$_2$-), 7.32 (*C*H$_3$CH$_2$Si), 7.14 (*C*H$_3$CH$_2$Si), 7.12 (*C*H$_3$CH$_2$Si), 7.11 (*C*H$_3$CH$_2$Si), 7.10 (*C*H$_3$CH$_2$Si), 7.07 (*C*H$_3$CH$_2$Si), 5.82 (CH$_3$*C*H$_2$Si), 5.34 (CH$_3$*C*H$_2$Si), 5.30 (CH$_3$*C*H$_2$Si), 5.28 (CH$_3$*C*H$_2$Si), 5.26 (CH$_3$*C*H$_2$Si), 5.23 (CH$_3$*C*H$_2$Si), 5.17 (CH$_3$*C*H$_2$Si). *See spectrum*.

IR (neat) ν_{max} (cm^{-1}): 2952, 2934, 2875, 1716 (C=O), 1648 (C=C), 1459, 1377, 1237, 1106, 1007 (Si-O), 801, 724 (Si-C), 671.

HRMS (MALDI) calculated for $C_{89}H_{190}O_{11}Si_7N$, $[M+NH_4]^+$: 1645.2724, found: 1645.2711.

$[\alpha]_D^{25}$ = –20.0 (c 0.60, CH_2Cl_2)

4. Preparation and Characterization Data of Bastimolide B (1)

4.1 Bastimolide B Synthesis

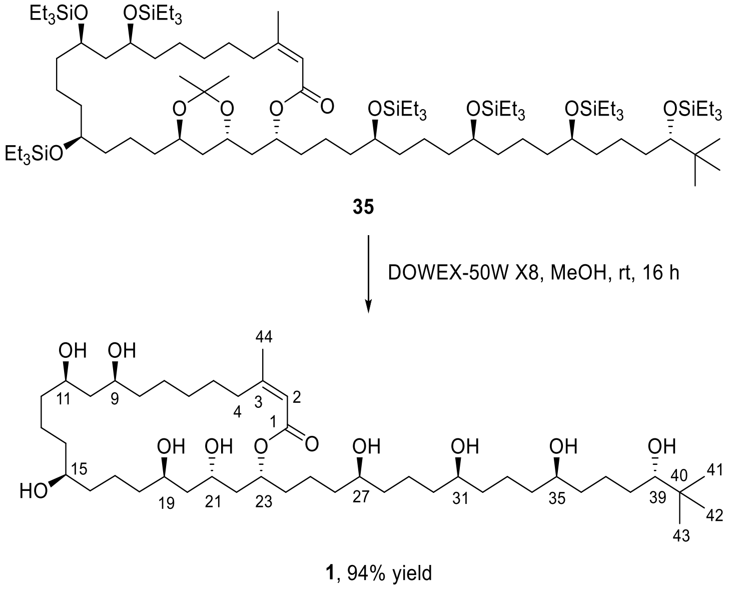

To a suspension of protected bastimolide B **35** (2.00 mg, 0.0012 mmol, 1.00 equiv) in HPLC grade MeOH (0.4 mL, 0.003 M), acidic resin DOWEX-50W X8 (8 mg), previously washed with HPLC grade MeOH, was added. The resulting reaction mixture was stirred 16 h at room temperature, then the mixture was filtered through a PTFE syringe filter and the filter washed with HPLC grade MeOH (5 mL). The combined organics were concentrated under reduced pressure and the solid obtained was dried under high vacuum to afford analytically pure bastimolide B (1.0 mg, 94% yield) as a white powder.

4.2 Bastimolide B Characterization

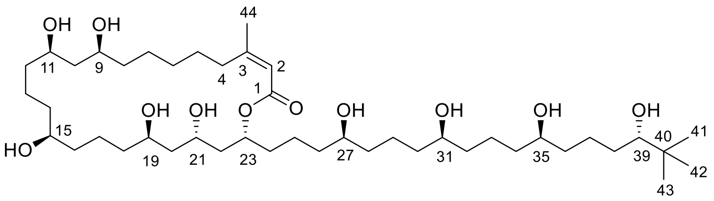

bastimolide B, **1**

^{1}H NMR (500 MHz, Methanol-*d*4) comparison table. *See spectrum*.

δH natural **1** (500 MHz, CD_3OD)[5]	δH synthetic **1** (500 MHz, CD_3OD)	Δ
5.70, s, 1H	5.68, 1H, s	0.02
5.06, m, 1H	5.07, 1H, m	-0.01
3.90, m, 1H	3.91, 1H, m	-0.01
3.85, m, 1H	3.86, 1H, m	-0.01
3.78, m, 1H	3.78, m, 1H	0
3.73, m, 1H	3.74, m, 1H	-0.01
3.62, m, 1H	3.62, m, 1H	0
3.54, m, 3H, overlapped	3.55, m, 3H, overlapped	0
3.12, d, *J* = 10.5 Hz, 1H	3.14, d, *J* = 10.3 Hz, 1H	-0.02
2.89, m, 1H	2.89, dd, *J* =11.9, 7.9 Hz, 1H	0
2.40, m, 1H	2.42, m, 1H	-0.02
1.91, s, 3H	1.92, *J* = 1.1 Hz, 3H	-0.01
1.83, m, 1H	1.84, ddd, *J* = 14.2, 7.1 Hz, 1H	0
1.72, m, 1H	1.73, m, 1H	-0.01
1.65, m, 1H	1.64, m, 1H	0.01
1.56, m, 2H	1.55, m, 2H	0.01
1.51, m, 1H	1.51, m, 1H	0
1.29 – 1.73, 44H overlapped	1.29 – 1.73, 44H overlapped	-
0.89, s, 9H	0.90, s, 9H	-0.01

Referenced to CD_3OD at 4.87 ppm. *a) n.a.*: not assigned.

^{1}H NMR (500 MHz, Py-*d*5). *See spectrum*. *See table of comparison and full chemical shift reassignment*.

^{13}C NMR (126 MHz, CD_3OD) comparison table. *See spectrum*.

The chemical shifts of **1** (126 MHz, CD_3OD) in the original publication were reported at the first decimal place.[5] Therefore, the comparison (Δ) takes into consideration this approximation.

δC natural **1** (126 MHz, CD_3OD)[5]	δC synthetic **1** (126 MHz, CD_3OD)	Δ
167.8	167.8	0.0
162.6	162.5	0.1
117.1	117.2	-0.1
80.5	80.5	0.0
72.4 (x2)	72.4 (x2)	0.0
72.2	72.2	0.0
71.8	71.8	0.0
71.5	71.5	0.0
71.1	71.1	0.0
70.8	70.8	0.0
68.8	68.8	0.0
66.7	66.7	0.0
44.3	44.4	-0.1
44.1	44.1	0.0
43.5	43.5	0.0
38.5	38.5	0.0
38.4 (x4)	38.5 (x4)	-0.1
38.3	38.3	0.0
38.2	38.2	0.1
38.1	38.1	0.0
37.9	37.9	0.0
37.0	37.0	0.0
36.9	36.9	0.0
35.9	35.9	0.0
35.8	35.8	0.0
34.2	34.2	0.0
32.3	32.3	0.0
30.6	30.6	0.0
29.3	29.3	0.0
26.4 (x3)	26.4 (x3)	0.0
26.0	26.0	0.0
25.4	25.4	0.0
24.4	24.4	0.0
23.0 (x2)	23.0 (x2)	0.0
22.5	22.5	0.0
21.9	22.0	-0.1
21.4	21.5	-0.1

Referenced to CD_3OD at 49.00 ppm.

^{13}C NMR (126 MHz, Py-*d*5). *See spectrum*. *See comparison spectra*. *See full chemical shift assignment.*

LC-MS of the synthetic sample of bastimolide B (**1**): $[M+H]^+$: 789.5, $[M+Na]^+$: 811.6.

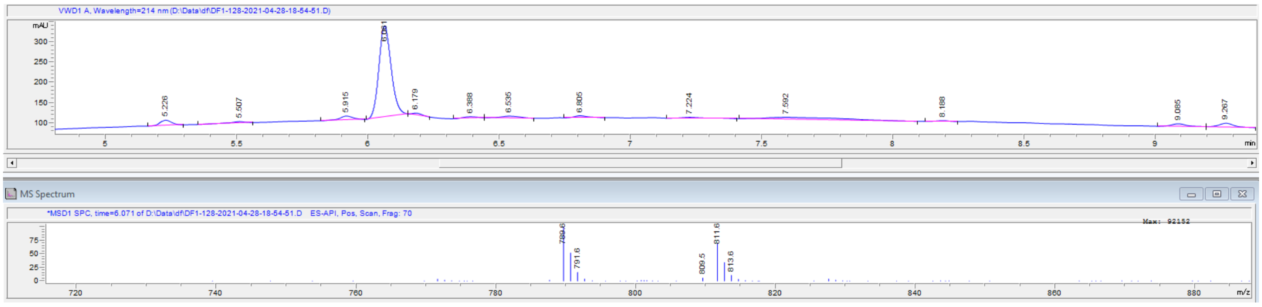

HRMS (ESI) calculated for $C_{44}H_{84}O_{11}Na$, $[M+Na]^+$: 811.5906, found: 811.5875.

[lit. **HRMS** (ESI) found 811.5907 $[M + Na^+]$.[5]

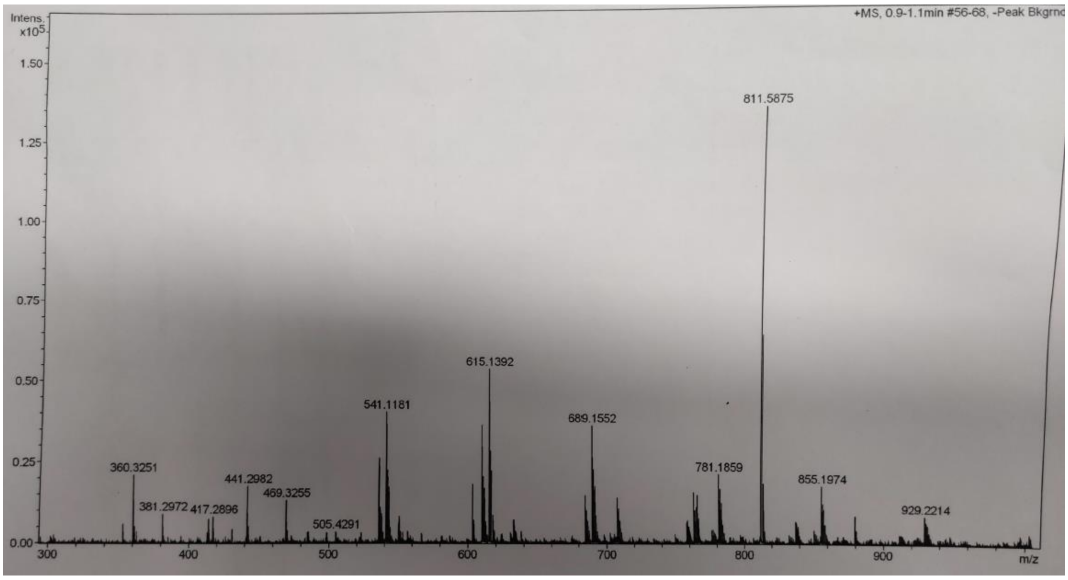

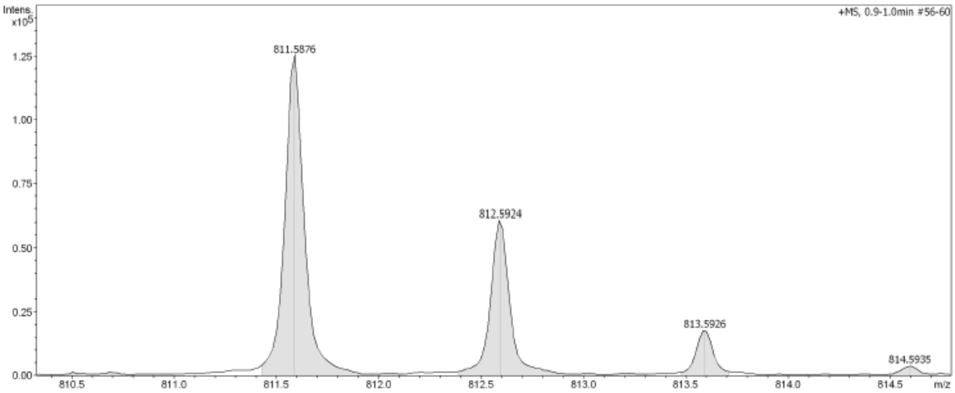

Compound Verification Results

Expected Formula: C44H84O11

#	meas. m/z	theo. m/z	|err| [ppm]	mSigma	Formula	Modification
2	811.5875	811.5906	3.7	2	C44 H84 Na O11	(M+Na)+

IR (neat) ν_{max} (cm^{-1}): 3358, 2921, 2853, 1693, 1633, 1457, 1156, 1237, 1102, 1033.

[lit. **IR** (KBr) ν_{max} (cm^{-1}): 3399, 2931, 1700, 1383, 1114 cm^{-1}][5]

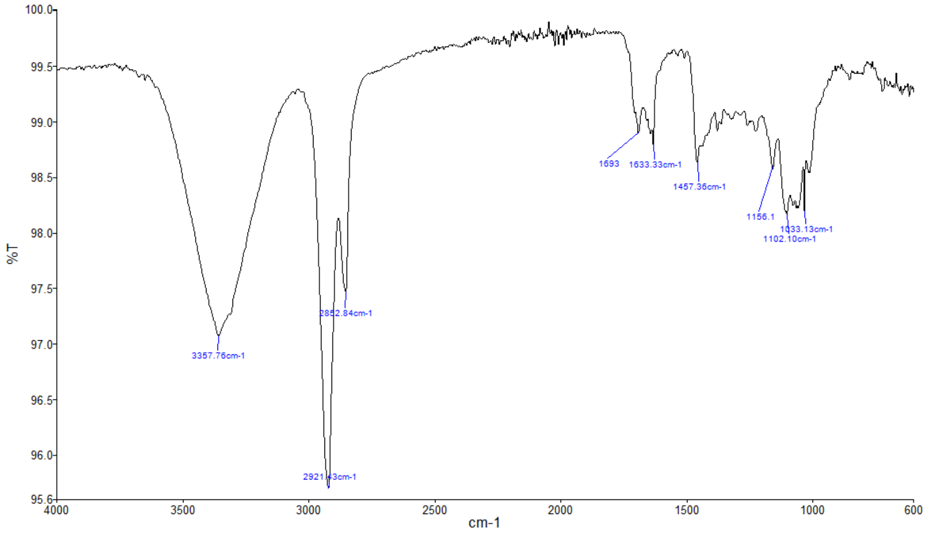

ECD: λ_{max} = 223 nm, $\Delta\varepsilon$ = –6.58 (0.63 mM, MeOH)

[lit. **ECD**: λ_{max} = 222 nm, $\Delta\varepsilon$ = –5.12 (0.63 mM, MeOH)][5]

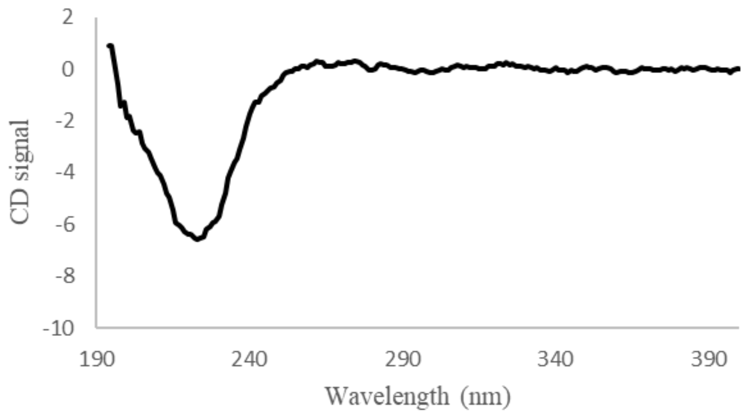

$[\alpha]_D^{25}$ = –23.5 (c 0.17, MeOH)

[lit. $[\alpha]_D^{25}$ = –11.3 (c 0.75, MeOH)][5]

4.3. Bastimolide B Full Chemical Shift Assignment (Py-*d*5)

The HSQC-TOCSY (Heteronuclear Single Quantum Coherence-TOtal COrrelation SpectroscopY) experiment is a 2D hybrid inverse gated experiment consisting of a HSQC pulse train, followed by a TOCSY mixing period. This provides a 2D spectrum which shows $^{n}J_{CH}$ correlations for a given spin system, where n is highly dependent upon the duration of the TOCSY mixing period (**D9** in the pulse program parameters). For the full chemical shift assignment of Bastimolide B, two HSQC-TOCSY spectra were required at mixing times of 30 ms and 60 ms. The spectra recorded with a 60 ms mixing time showed $^{1\text{-}3}J_{CH}$ correlations, which were used to assign the chemical shifts of the stereogenic carbon atoms, and their respective protons. The spectra recorded with a 60 ms mixing time showed $^{1\text{-}4}J_{CH}$ correlations, which were used to assign the chemical shifts of carbon atoms adjacent to stereocenters. Spectra were recorded on a 700 MHz spectrometer fitted with a 1.7mm cryo-enhanced micro coil probe and were aliased to (7704.2, 5988.0) with lowest frequencies at (-676.0, 2401.0) for ^{1}H and ^{13}C respectively (values recorded in Hz). This achieved a spectral resolution of 10.0 Hz in f2 and 2.92 Hz in f1. NMR sample prepared with 0.5 mg of synthetic Bastimolide B were dissolved in *ca.* 40 µL pyridine-d5 (≥99.96% atom % D) in a 1.7 mm NMR tube.

^{1}H NMR (700 MHz, Py-*d*5). *See spectra*.

Position	δH natural **1** (800 MHz, Py-*d*5)[5]	δH synthetic **1** (700 MHz, Py-*d*5)
2	5.83, s	5.83, s
4	3.14, dd (8, 4)<br>2.41, m	3.14, ddd (11.7, 9.2, 6.5)<br>2.41, ddd (11.5, 8.9, 5.3)
5	1.52, m	1.53, m
6	1.46, m; 1.42, m	1.42, m
7	1.65, m; 1.60, m	1.65, m; 1.59, m
8	1.65−1.98, m	1.76, m, 1.70, m
9	4.23, br s	4.23, br s
10	2.04, m, 1.96, m	2.04, m; 1.93, m
11	4.32, br s	4.33 br s
12	1.65−1.98, m	1.95, m; 1.88, m
13	1.77, m	1.95, m; 1.88, m
14	1.65−1.98, m	1.88, m
15	3.96, m	4.07, br s
16	1.65−1.98, m	1.88, m
17	1.77, m	1.93, m; 1.90, m
18	1.65−1.98, m	1.93, m; 1.81, m
19	4.51, br s	4.50, br s
20	2.01, m	2.01, m
21	4.59, br s	4.59, br s
22	2.34, ddd (8, 4, 4)<br>2.05, m	2.34, ddd (14.16, 7.25, 7.25)<br>2.05, m
23	5.63, m	5.64, m
24	1.87, m	1.87, m
25	1.70, m	1.87, m; 1.78, m
26	1.65−1.98, m	1.72, m; 1.65, m
27	4.07, m	3.89, br s
28	1.65−1.98, m	1.72, m
29	1.77, m	1.78, m; 2.13, m
30	1.65−1.98, m	1.75, m
31	3.94, m	3.94, br s
32	1.65−1.98, m	1.76, m
33	1.77, m	2.15, m; 1.81, m
34	1.65−1.98, m	1.78, m
35	3.88, br s	3.97, br s
36	1.65−1.98, m	1.85, m; 1.72, m
37	2.16, mb, 1.94, m	1.91, m; 2.17, m
38	1.62, m	1.67, m
39	3.47, m	3.47, br s
41−43	1.08, s	1.08, s
44	1.77, s	1.77, s

Referenced to Pyr-*d*5 at 8.74 ppm.

Previous chemical assignments or non-unique assignments highlighted in red have been revised.

^{13}C NMR (126 MHz, Py-*d*5). See spectra. See HSQC-TOCSY.

The chemical shifts of **1** (200 MHz, Py-*d*5) in the original publication were reported at the first decimal place[5] Herein, two significant decimal digits are reported for the comparison (Δ) based on the reanalysis of the original NMR file, kindly provided by the isolation team. Moreover, the peak originally described at 38.3 ppm was revised (corrected value: 38.74 ppm).

δC natural **1**	δC synthetic **1**	Δ	Originally Assigned Position, type	Reassignment	
(200 MHz, Py-*d*5)	(126 MHz, Py-*d*5)			δC	Position, type
166.68	166.69	-0.01	1 C	166.69	1 C
160.72	160.72	0	3 C	160.72	3 C
117.48	117.48	0	2 CH	117.48	2 CH
79.35	79.34	0.01	39 CH	79.34	39 CH
71.74	71.74	0	9,11 CH	71.74	23 CH
71.66	71.66	0	9,11 CH	71.66	9 CH
71.59	71.59	0	23, 31 CH	71.59	35 CH
71.55	71.55	0	23, 31 CH	71.55	31 CH
71.38	71.38	0	15 CH	71.38	11 CH
71.24	71.23	0.01	35 CH	71.23	27 CH
70.72	70.72	0	27 CH	70.72	15 CH
68.34	68.34	0	19 CH	68.34	19 CH
66.32	66.32	0	21 CH	66.32	21 CH
44.90	44.89	0.01	20 CH$_2$	44.89	20 CH$_2$
44.53	44.53	0	10 CH$_2$	44.53	10 CH$_2$
44.02	44.02	0	22 CH$_2$	44.02	22 CH$_2$
39.25	39.25	0	8 CH$_2$	39.25	34 CH$_2$
39.20	39.19	0.01	12 CH$_2$	39.19	32 CH$_2$
39.14	39.14	0	14, 16, 18 CH$_2$	39.14	30 CH$_2$
39.13	39.12	0.01	14, 16, 18 CH$_2$	39.12	28 CH$_2$
39.07	39.06	0.01	14, 16, 18 CH$_2$	39.06	36 CH$_2$
38.98	38.98	0	34 CH$_2$	38.98	18 CH$_2$
38.91	38.90	0.01	32 CH$_2$	38.90	8 CH$_2$
38.77	38.77	0	30 CH$_2$	38.77	26 CH$_2$
38.74	38.74	0	28 CH$_2$	38.74	12 CH$_2$
37.61	37.61	0	36 CH$_2$	37.61	14 CH$_2$
37.45	37.45	0	26 CH$_2$	37.45	16 CH$_2$
35.89	35.88	0.01	24 CH$_2$	35.88	40 C
35.77	35.77	0	40 C	35.77	24 CH$_2$
33.69	33.69	0	4 CH$_2$	33.69	4 CH$_2$
32.74	32.73	0.01	38 CH$_2$	32.73	38 CH$_2$
30.22	30.21	0.01	6 CH$_2$	30.21	6 CH$_2$
28.87	28.86	0.01	5 CH$_2$	28.86	5 CH$_2$
26.78	26.77	0.01	41-43 CH$_3$	26.77	41-43 CH$_3$
26.06	26.05	0.01	7 CH$_2$	26.05	7 CH$_2$
25.44	25.43	0.01	44 CH$_2$	25.43	44 CH$_3$
24.68	24.68	0	37 CH$_2$	24.68	37 CH$_2$
23.37	23.36	0.01	29, 33 CH$_2$	23.36	33 CH$_2$
23.35	23.36	-0.01	29, 33 CH$_2$	23.36	29 CH$_2$
22.74	22.74	0	25 CH$_2$	22.74	25 CH$_2$
22.42	22.41	0.01	13 CH$_2$	22.41	17 CH$_2$
21.50	21.49	0.01	17 CH$_2$	21.49	13 CH$_2$

Referenced to Pyr-*d*5 at 123.87 ppm. Previous chemical assignments or non-unique assignments highlighted in red have been revised.

5. Optimization of Fragment Coupling on Model Compounds

Deuteration experiment

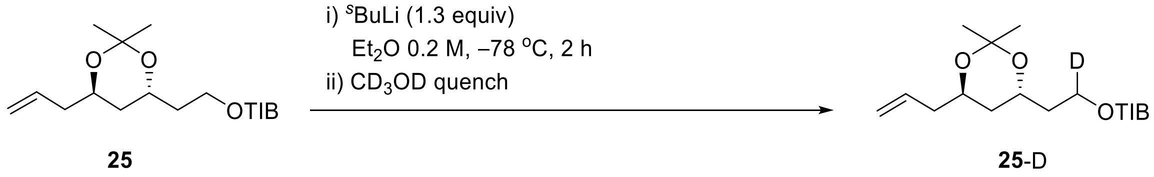

with (+)-sparteine (1.3 equiv) >99% D incorporation

without (+)-sparteine (1.3 equiv) 0% D incorporation

React-IR and ^{11}B NMR reaction monitoring

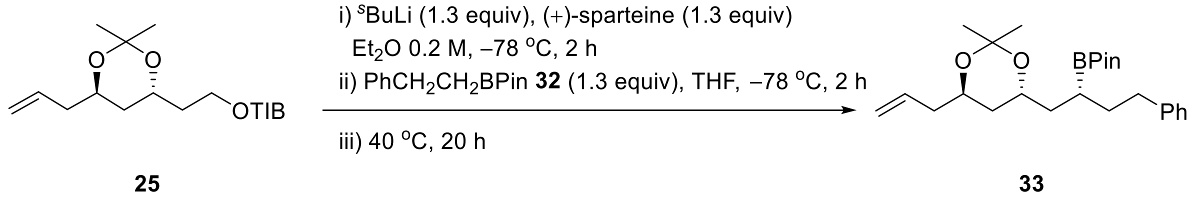

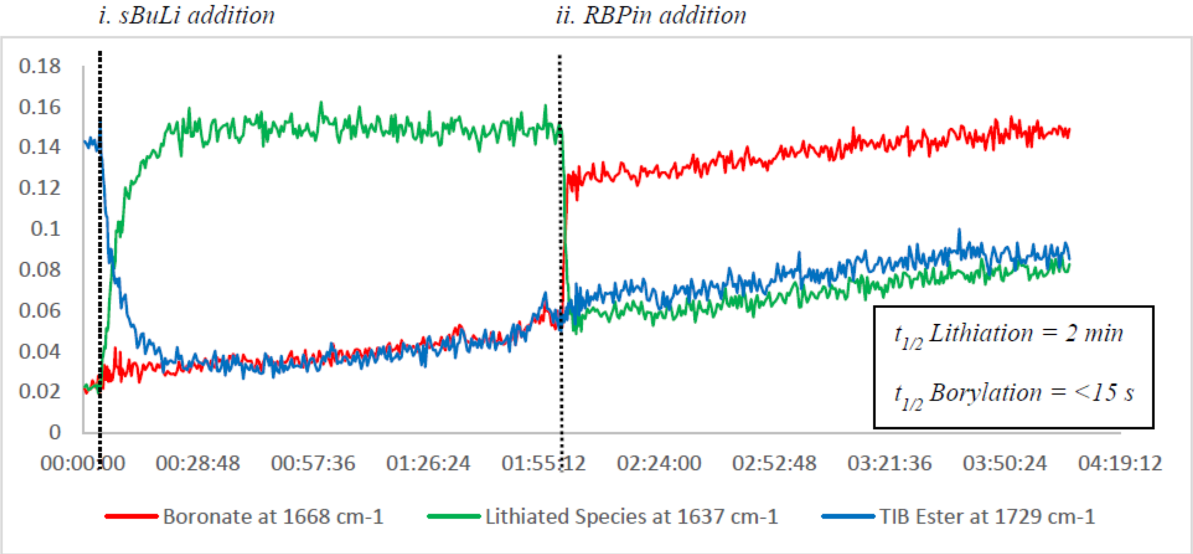

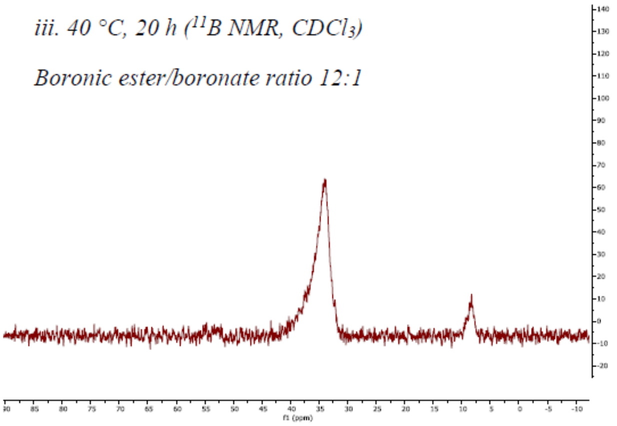

Study on substrate-controlled diastereoselective lithiation/borylation

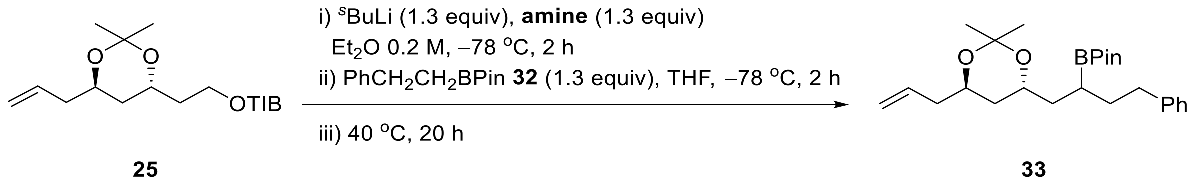

^{13}C NMR (101 MHz, CD_2Cl_2) of compound **33** using either TMEDA, (-)-sparteine or (+)-sparteine.

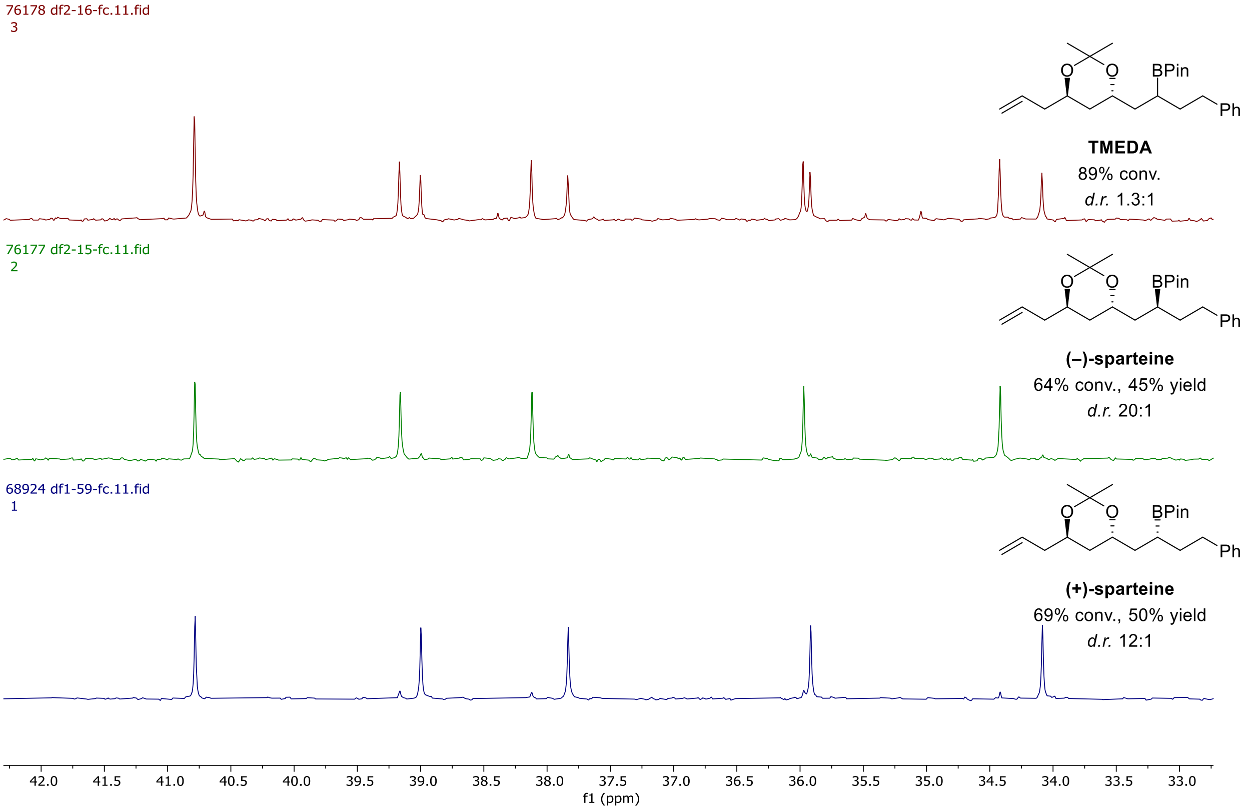

Reaction optimization: excess of boronic ester **32**

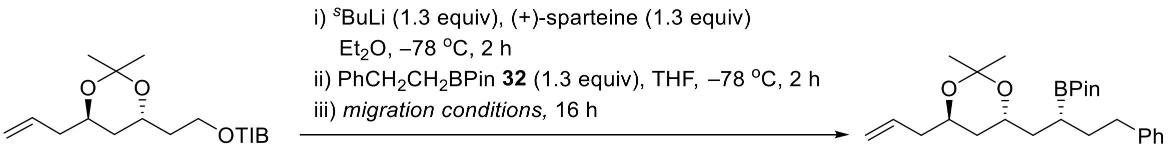

25 **33**

no additive<br>40 °C<br>**69% conv., 50% yield**	$MgBr_2$ Et_2O (1.5 equiv)<br>40 °C<br>**69% conv., 55% yield**	$MgBr_2$ in MeOH 1M (1.5 equiv)<br>40 °C<br>**41% conv.**
solvent switch to $CHCl_3$<br>65 °C<br>**24% conv.**	solvent switch to CPME<br>110 °C<br>**55% conv.**	solvent switch to toluene<br>110 °C<br>**51% conv.**

Reaction optimization: excess of benzoate **25**

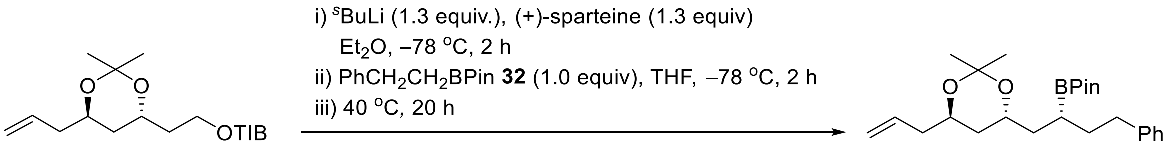

25

33
89% conv., 65% yield
12:1 dr
*27% recovered benzoate **25***

2-((*R*)-1-((4*R*,6*R*)-6-Allyl-2,2-dimethyl-1,3-dioxan-4-yl)-4-phenylbutan-2-yl)-4,4,5,5-tetramethyl-1,3,2-dioxaborolane (33)

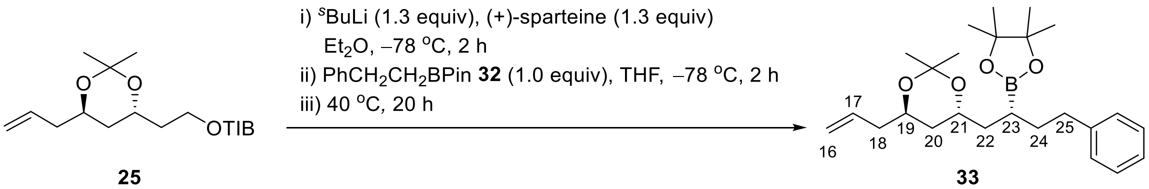

To a solution of TIB ester **25** (56 mg, 0.13 mmol, 1.30 equiv) and (+)-sparteine (27 mg, 0.13 mmol, 1.30 equiv) in diethyl ether (0.65 mL, 0.20 M), ^{s}BuLi (1.30 M solution in heptane, 0.10 mL, 0.13 mmol, 1.30 equiv) was added dropwise at –78 °C and the reaction mixture was stirred at same temperature for 2 h. Boronic ester **32** (23 mg, 0.10 mmol, 1.00 equiv) was rapidly added as a solution in THF (0.10 mL, 1.00 M) to the reaction mixture at –78 °C, and the reaction mixture was stirred for 2 h at –78 °C and then it was warmed up to room temperature and stirred 20 hour at 40 °C. The reaction was quenched by the addition of saturated aqueous NH_4Cl (1 mL) and extracted with Et_2O (3x5 mL). The organics were concentrated under reduced pressure and the crude residue was purified by automated flash column chromatography (Biotage HC-10g, pentane: EtOAc 1-10%) to afford the title compound **33** (27 mg, 65% yield, 12:1 dr) as a waxy solid and recovered OTIB ester **25** (15 mg).

^{1}H NMR (400 MHz, Methylene Chloride-*d*$_2$) δ 7.28 – 7.21 (m, 2H, *H*-Ar), 7.20 – 7.11 (m, 3H, *H*-Ar), 5.79 (ddt, *J* = 17.1, 10.3, 6.8 Hz, 1H, *H*-17), 5.06 (dd, *J* = 17.1, 1.9 Hz, 1H, *H*-16), 5.01 (ddt, *J* = 10.2, 2.3, 1.2 Hz, 1H, *H*-16), 3.86 – 3.72 (m, 2H, *H*-19, 21), 2.58 (ddd, *J* = 13.5, 9.6, 6.4 Hz, 2H, *H*-25), 2.31 – 2.07 (m, 2H, *H*-18), 1.75 – 1.46 (m, 5H, *H*-20, 22, 24), 1.41 (ddd, *J* = 13.4, 6.3, 5.2 Hz, 1H, *H*-24), 1.28 (s, 3H, C*H*$_3$-acetonide), 1.26 (s, 3H, C*H*$_3$-acetonide), 1.24 (s, 12H, C*H*$_3$-BPin), 1.11 – 0.99 (m, 1H, *H*-23). See spectrum.

^{13}C NMR (101 MHz, Methylene Chloride-*d*$_2$) δ ^{13}C NMR (101 MHz, CD_2Cl_2) δ 143.68 (*C*-Ar), 135.53 (*C*H-17), 128.94 (*C*H-Ar), 128.76 (*C*H-Ar), 126.11 (*C*H-Ar), 116.84 (*C*H$_2$-16), 100.69 (*C*O *anti*-acetonide), 83.52 (*C*H$_3$-BPin), 66.79 (*C*H-19), 66.47 (*C*H-21), 40.78 (*C*H$_2$-18), 39.00 (*C*H$_2$-20), 37.83 (*C*H$_2$-24), 35.92 (*C*H$_2$-25), 34.08 (*C*H$_2$-22), 25.35 (*C*H$_3$-BPin), 25.27 (*C*H$_3$-BPin), 25.19 (*C*H$_3$-*anti*-acetonide), 25.09 (*C*H$_3$-*anti*-acetonide). See spectrum.

IR (neat) ν_{max} (cm^{-1}): 2981, 2929, 1455, 1375, 1315, 1222, 1143, 1165, 1110, 996, 911, 747, 698.

HRMS (ESI) calculated for $C_{25}H_{39}BO_4Na$ $[M+Na]^+$: 437.2838, found: 437.2846.

$[\alpha]_D^{25} = -1.5$ (c 0.27, CH_2Cl_2)

6. NMR Spectra of New Compounds

^{1}H NMR (400 MHz, $CDCl_3$) of compound 13. See procedure.

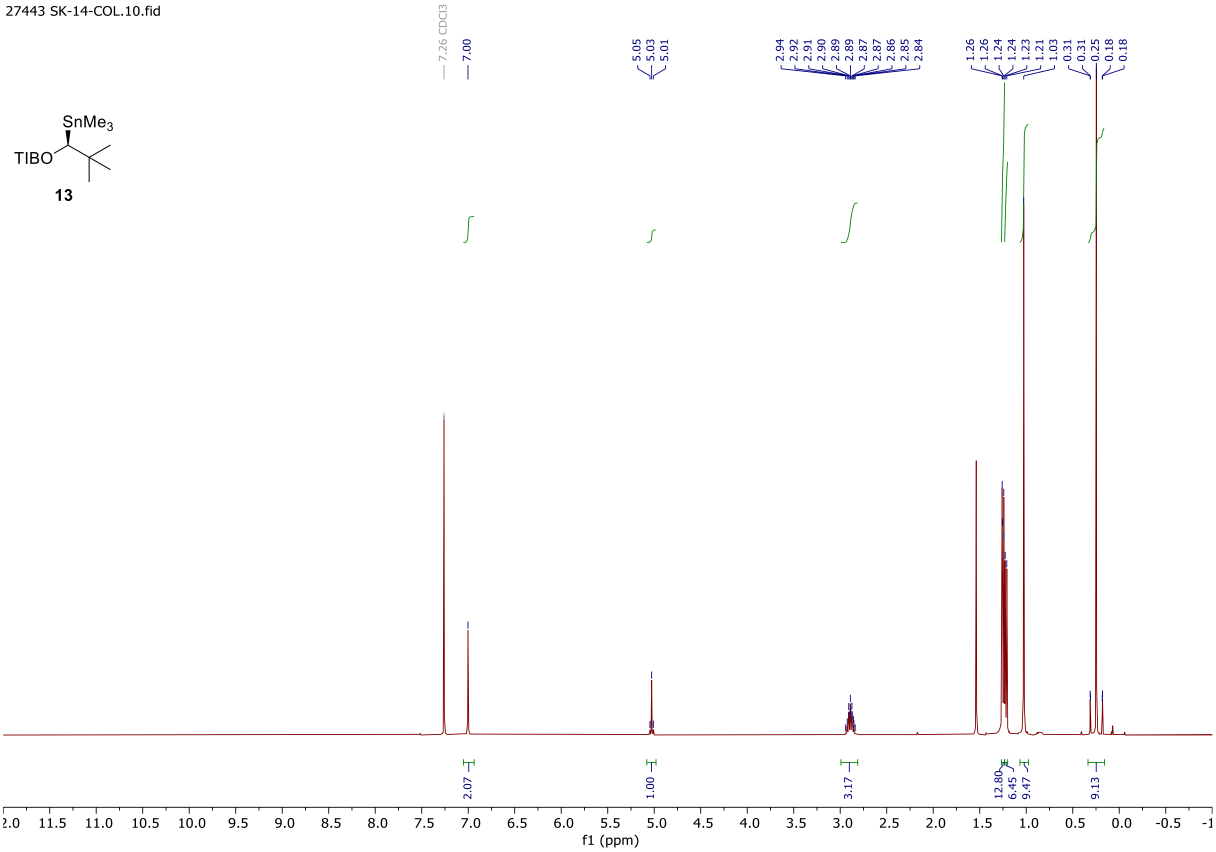

^{13}C NMR (101 MHz, $CDCl_3$) of compound 13. See procedure.

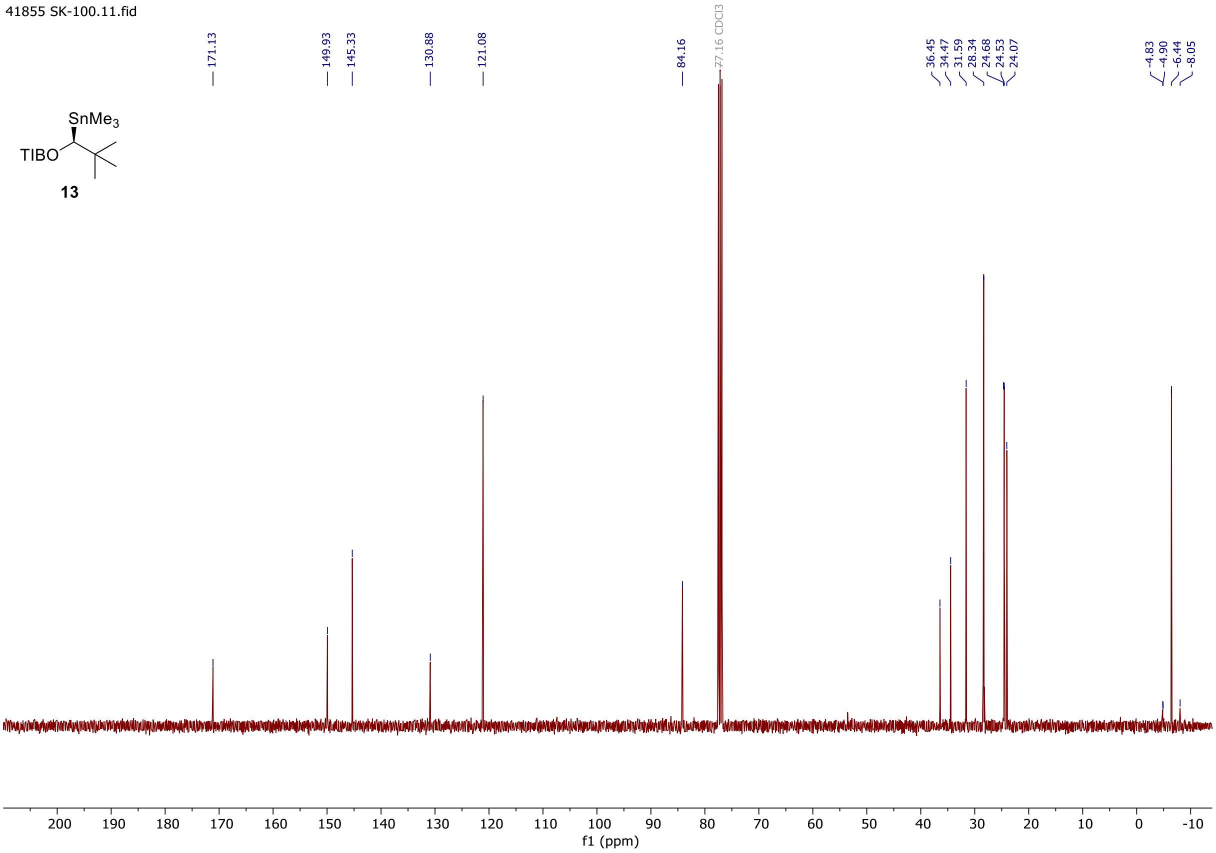

^{1}H NMR (400 MHz, $CDCl_3$) of compound 15. *See procedure.*

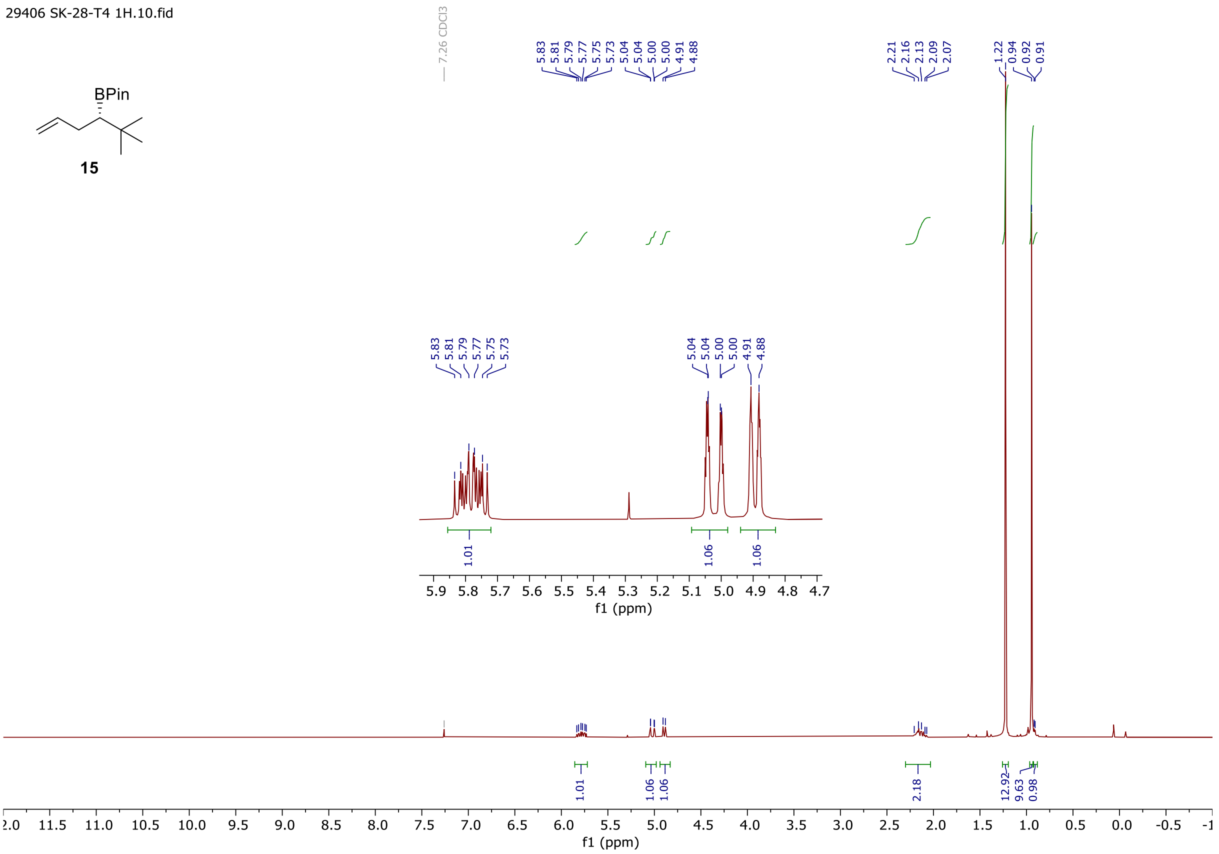

^{13}C NMR (101 MHz, $CDCl_3$) of compound 15. *See procedure*.

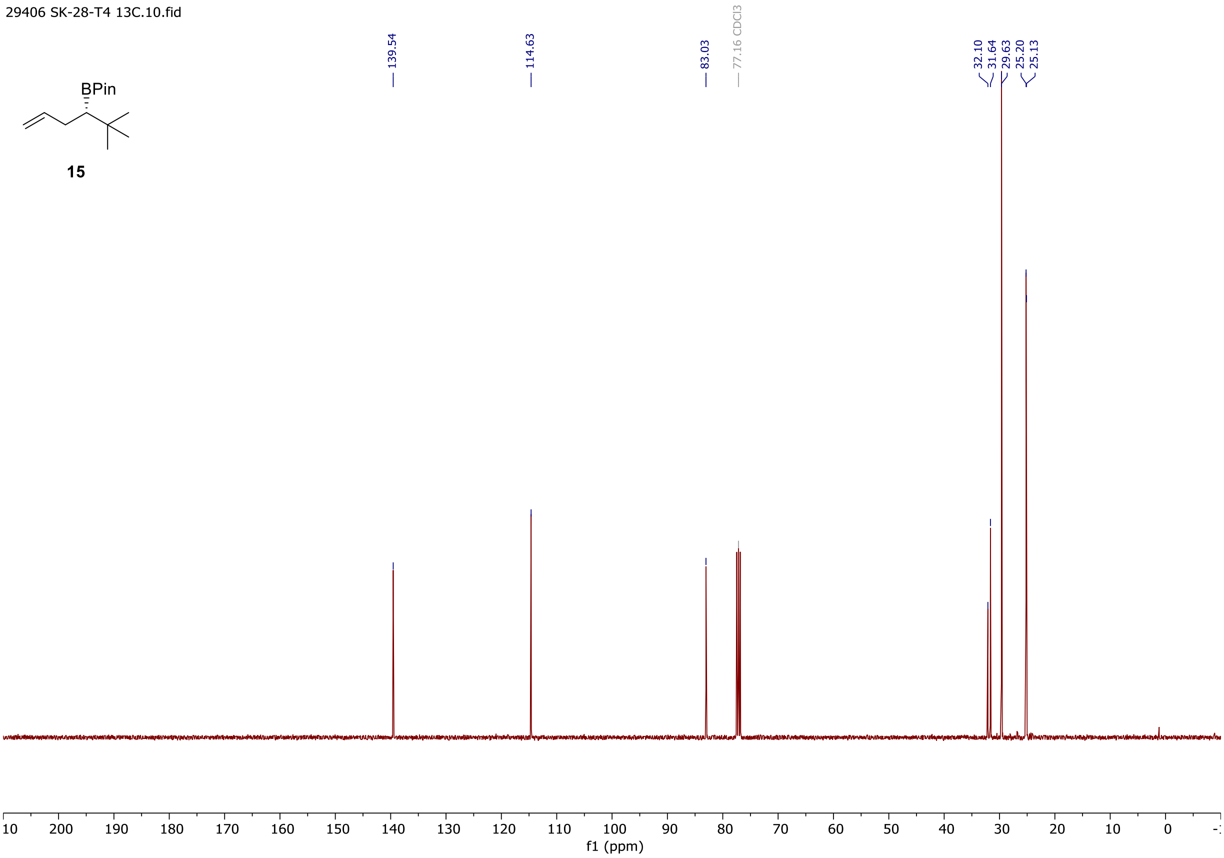

^{1}H NMR (400 MHz, CDCl$_3$) of compound 16. *See procedure.*

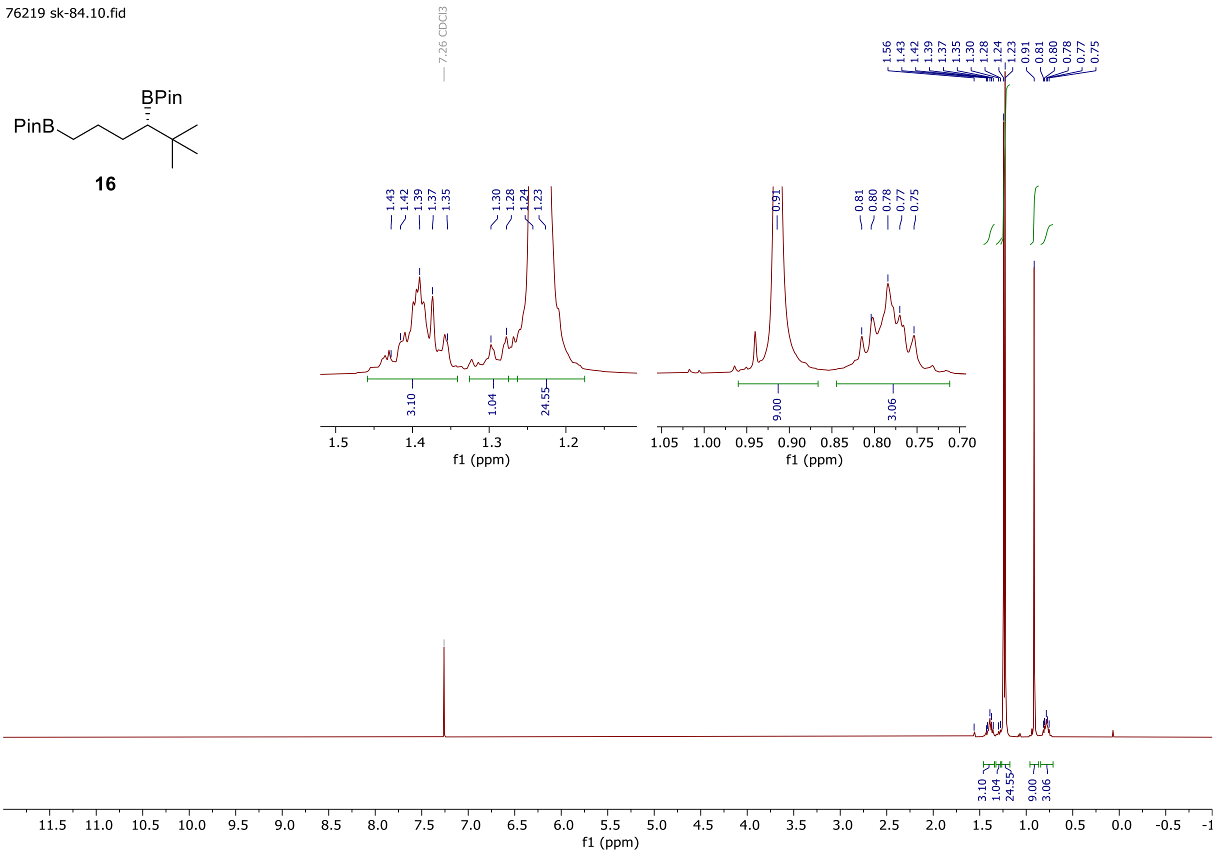

^{13}C NMR (101 MHz, CDCl3) of compound 16. *See procedure.*

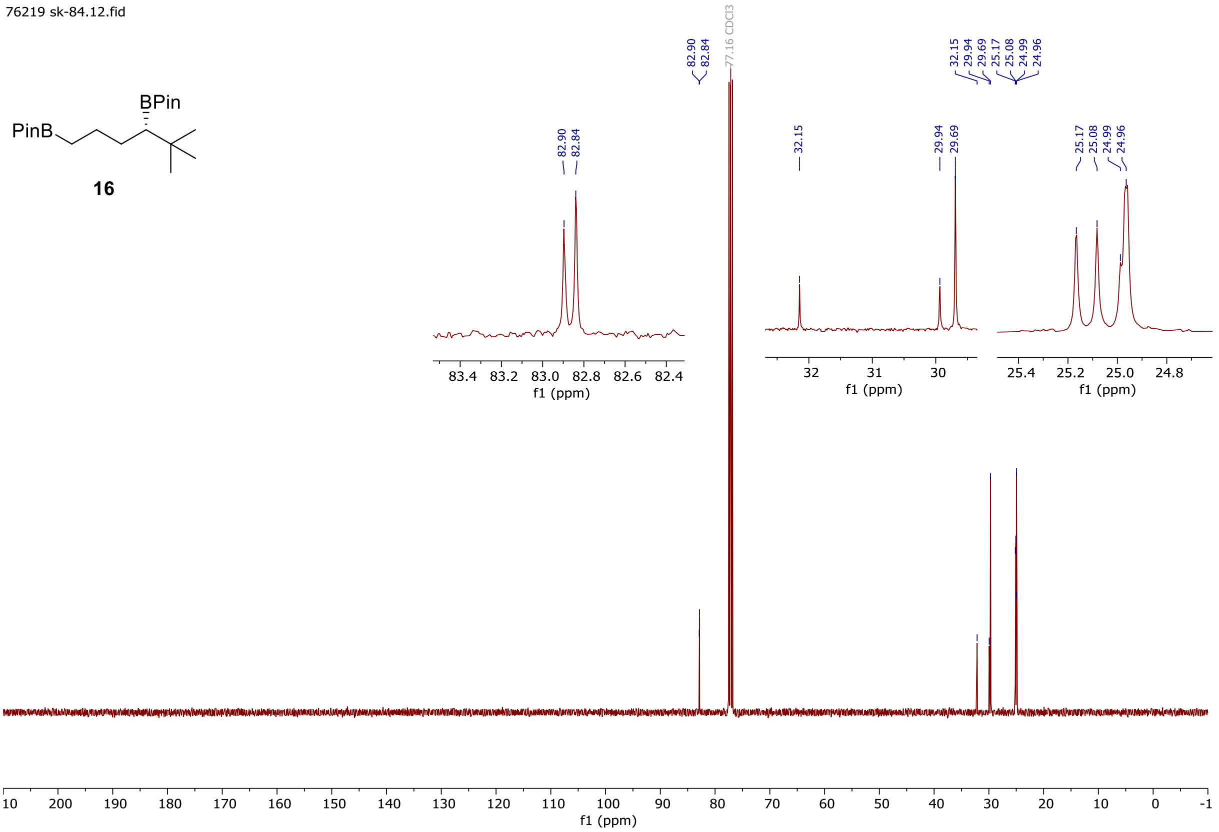

^{1}H NMR (400 MHz, $CDCl_3$) of compound 17. See procedure.

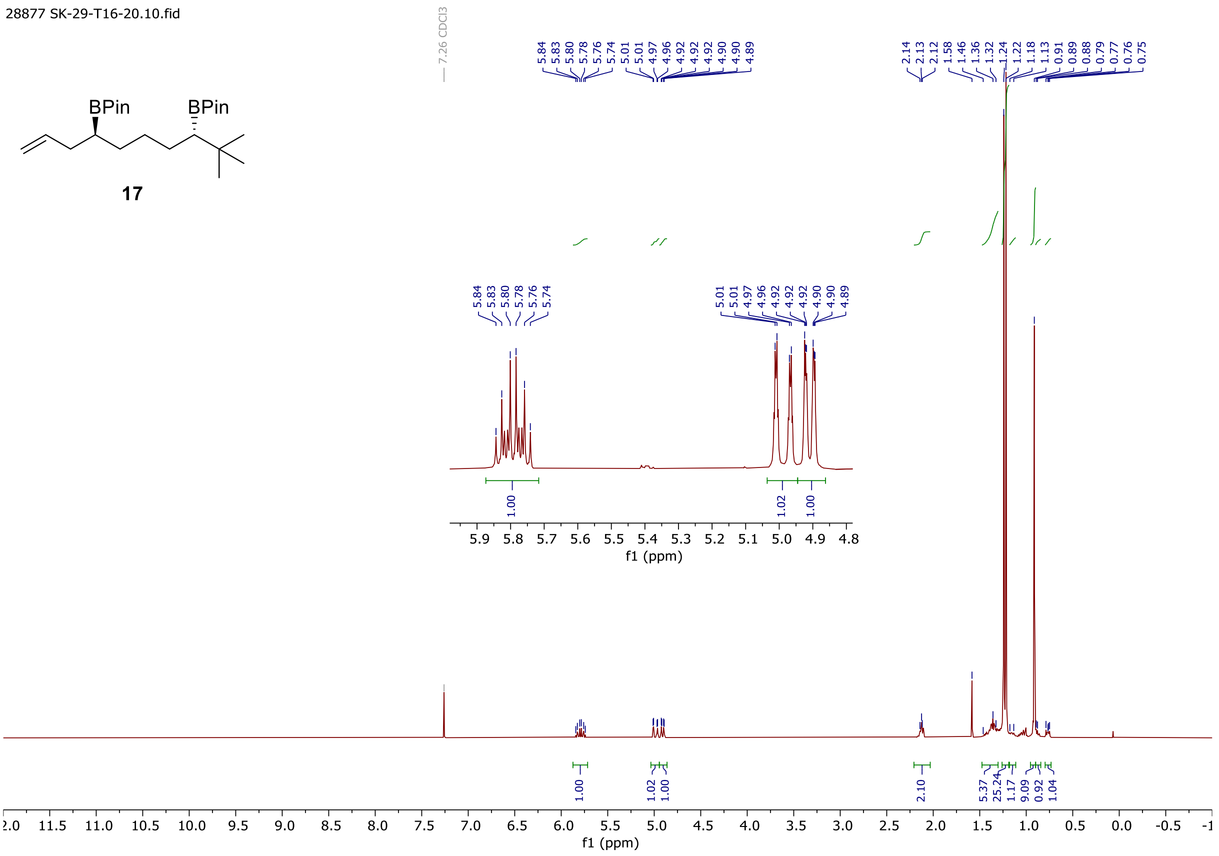

^{13}C NMR (101 MHz, $CDCl_3$) of compound 17. See procedure.

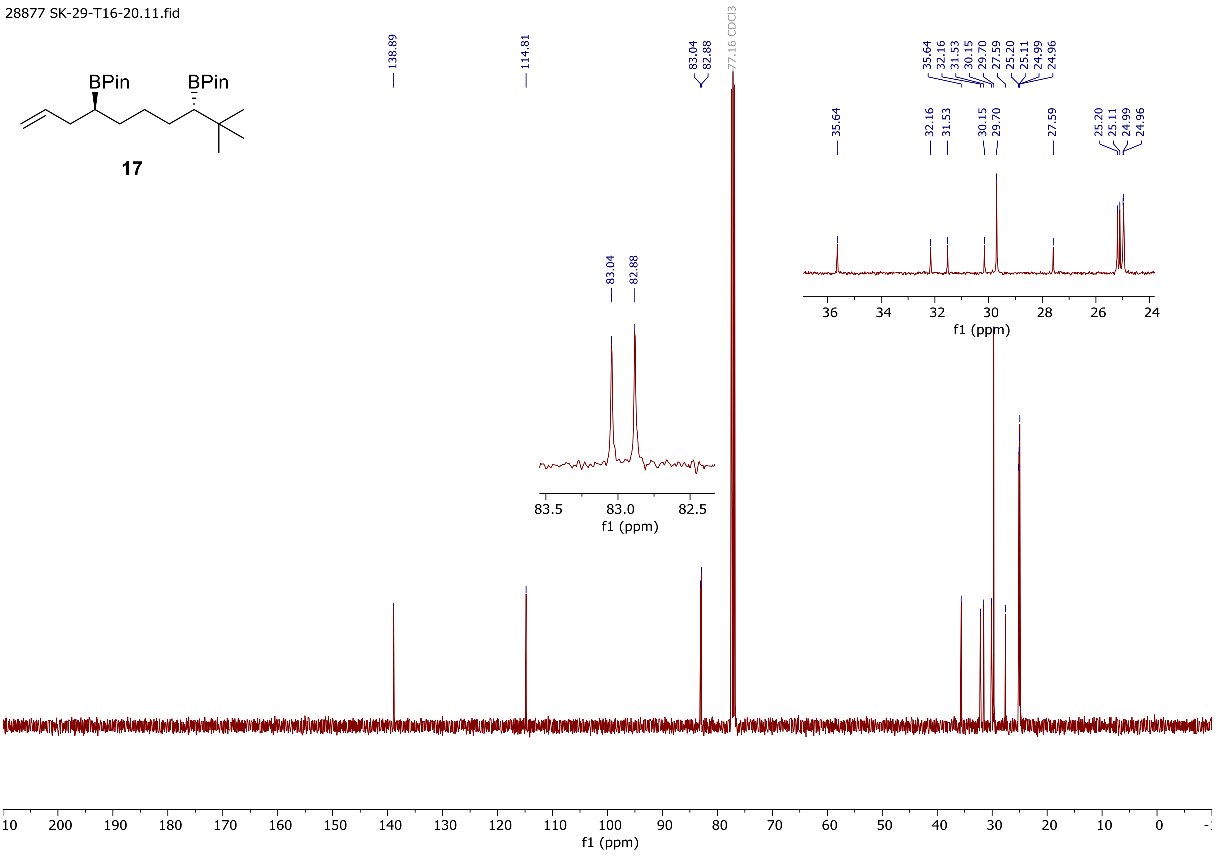

^{13}C NMR (101 MHz, CDCl$_3$) of compound 17-mix (1:1 dr). See procedure.

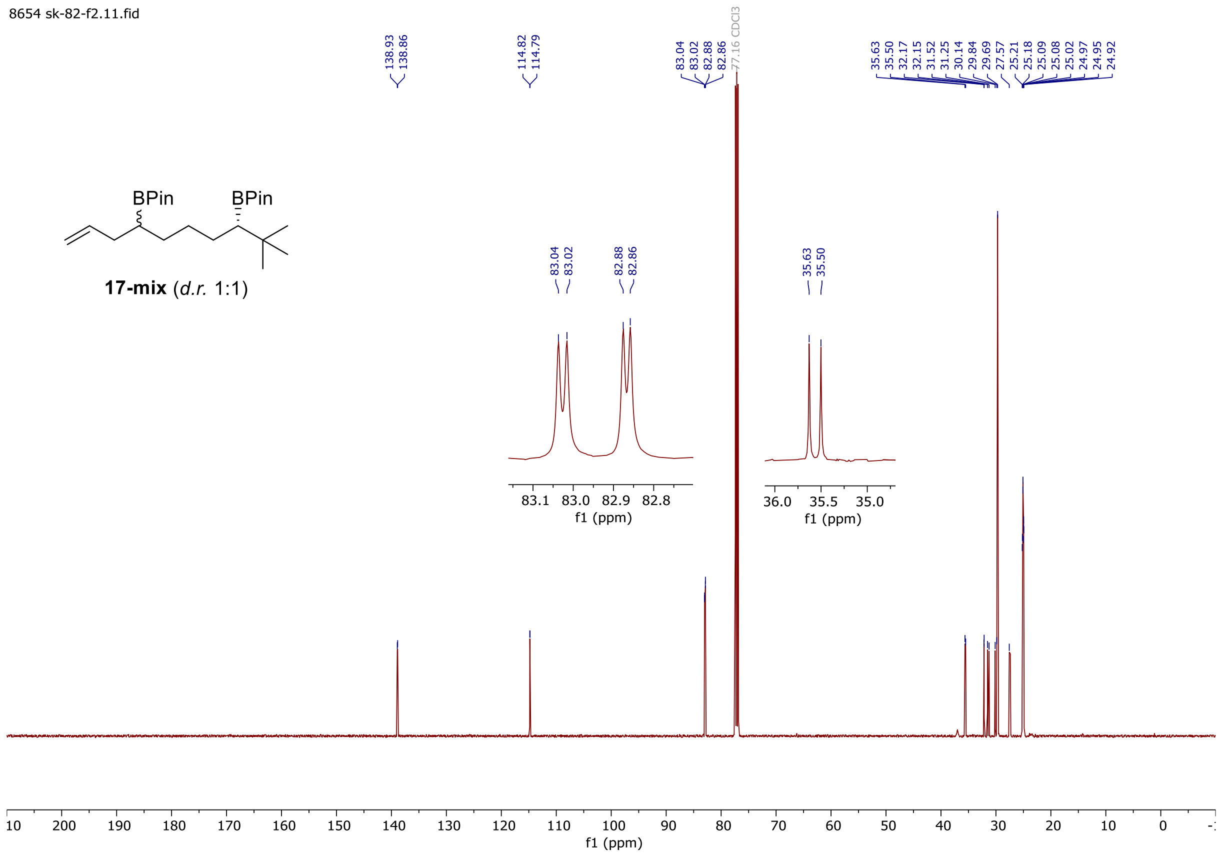

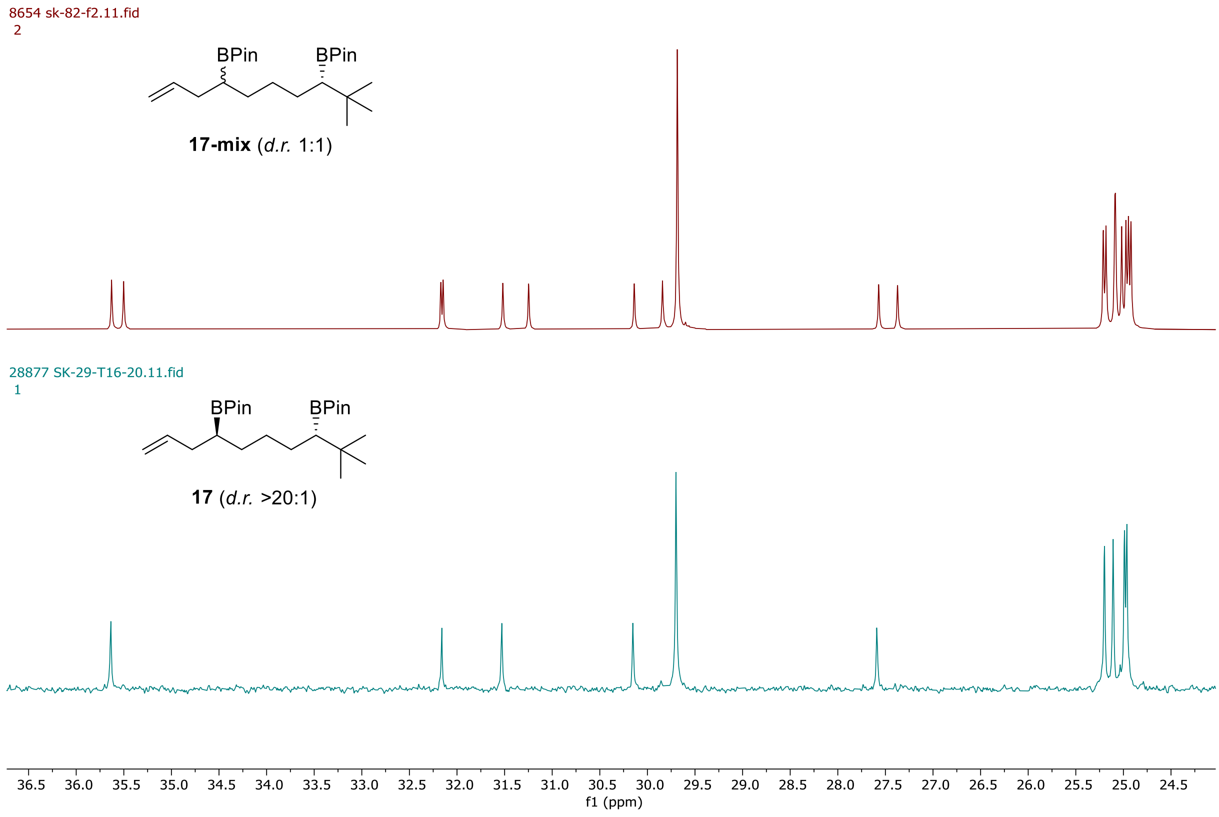

^{1}H NMR (400 MHz, $CDCl_3$) of compound 18. See procedure.

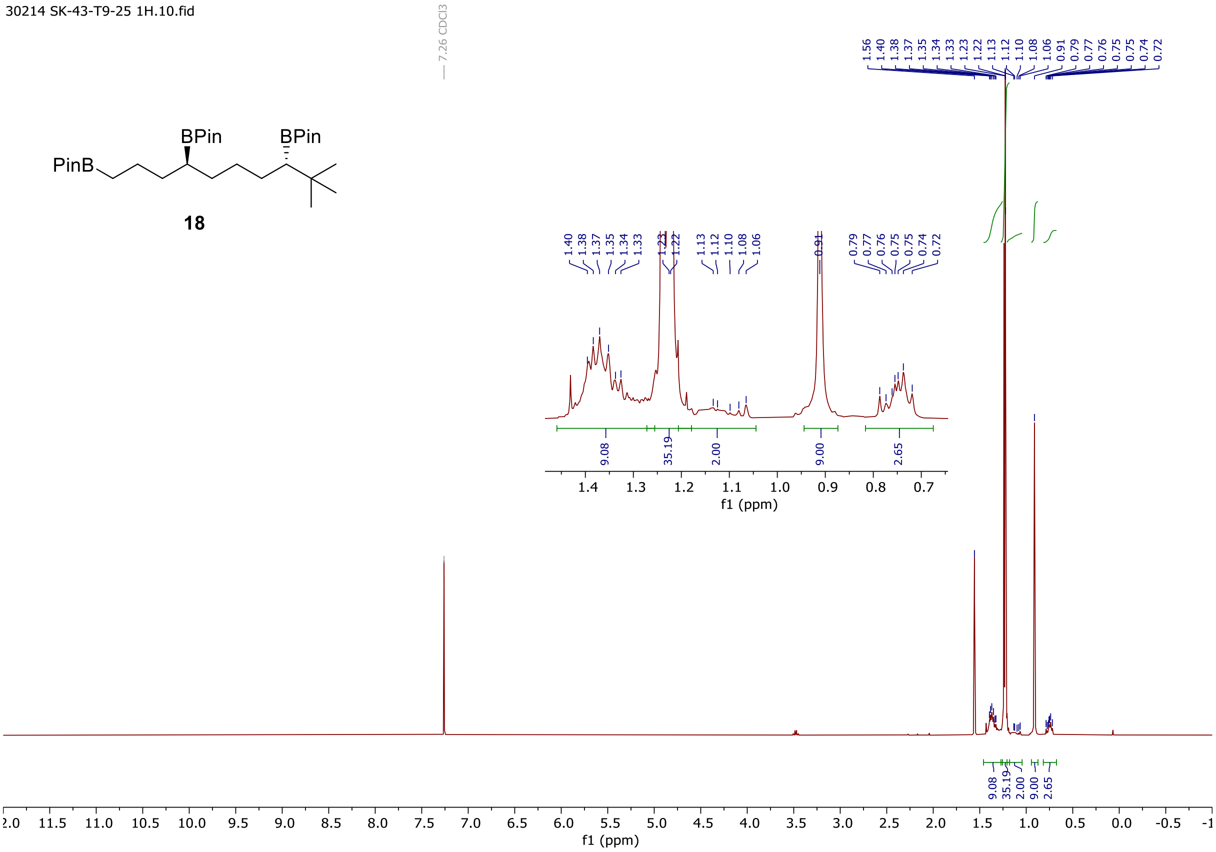

^{13}C NMR (101 MHz, $CDCl_3$) of compound 18. See procedure.

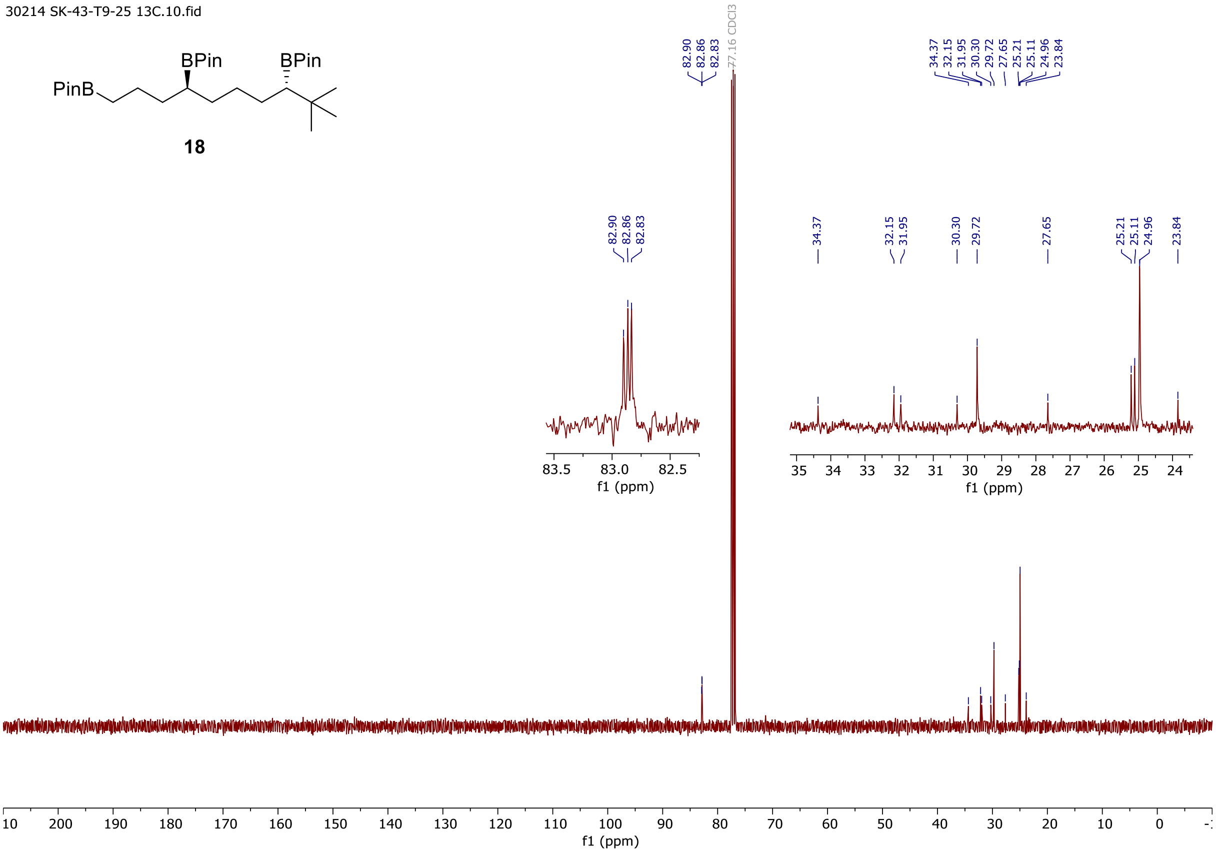

^{1}H NMR (400 MHz, $CDCl_3$) of compound 19. See procedure.

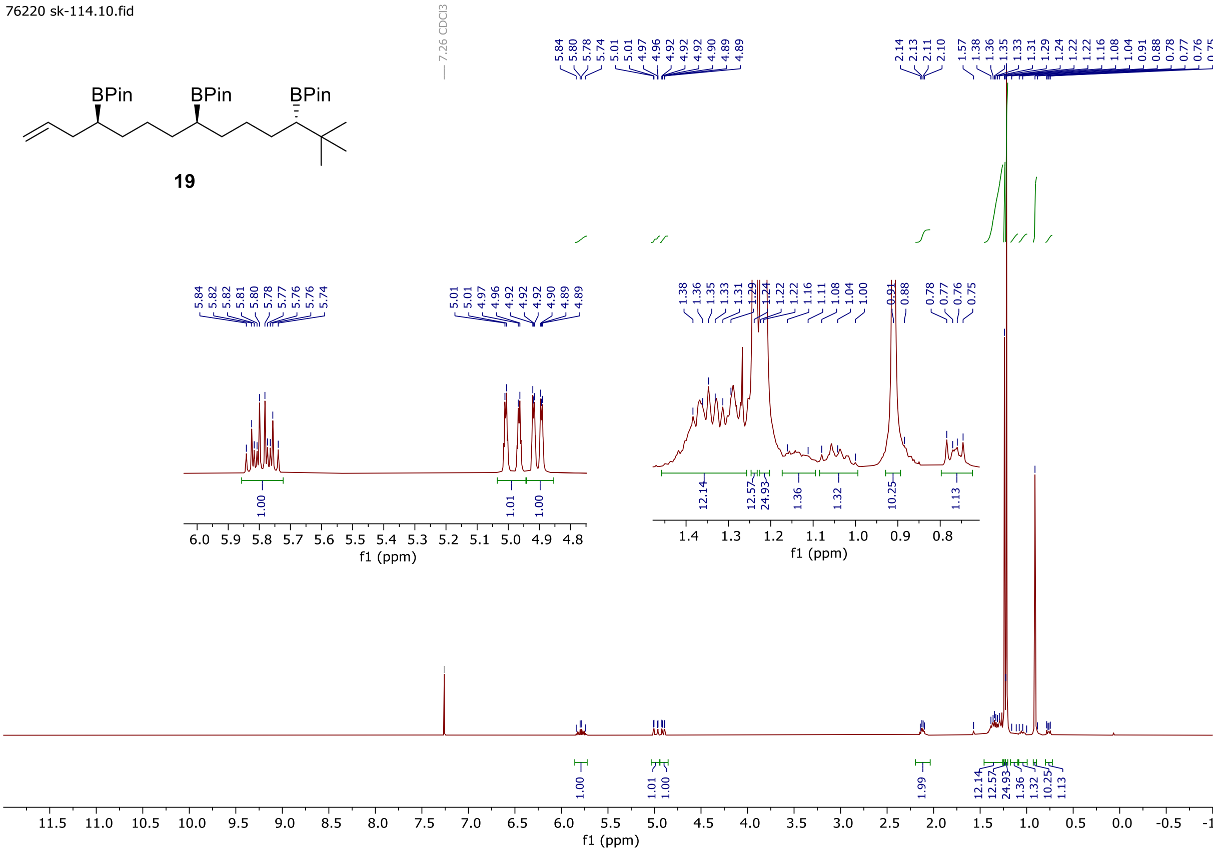

^{13}C NMR (101 MHz, $CDCl_3$) of compound 19. See procedure.

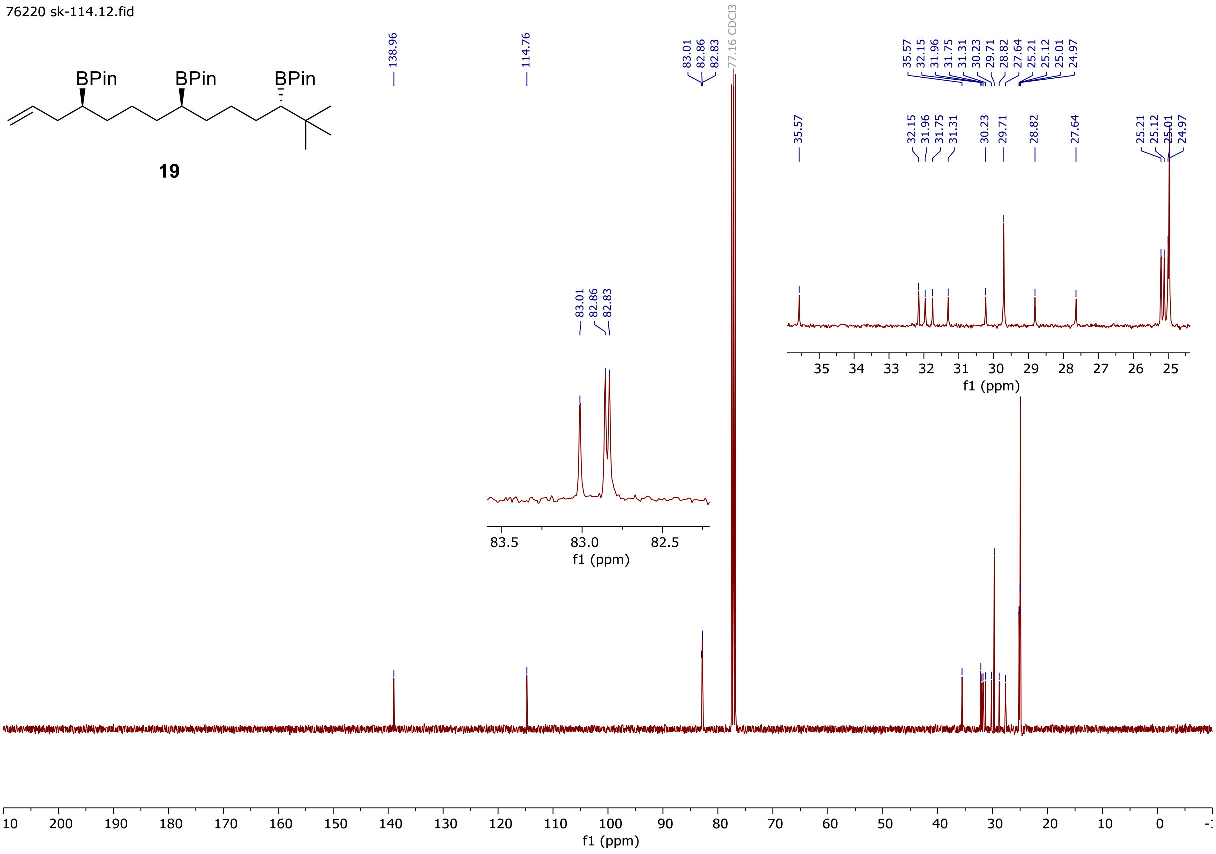

^{1}H NMR (400 MHz, $CDCl_3$) of compound 20. *See procedure.*

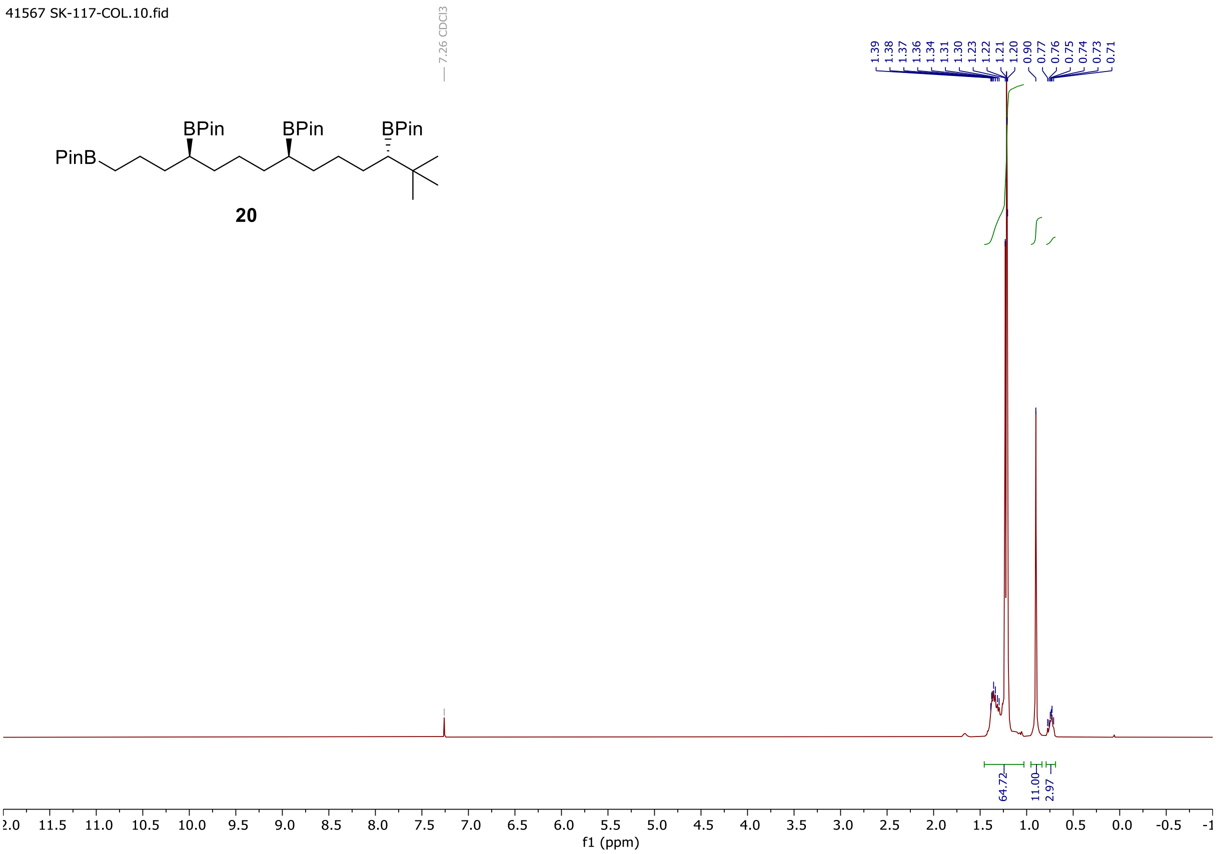

^{13}C NMR (101 MHz, $CDCl_3$) of compound 20. *See procedure.*

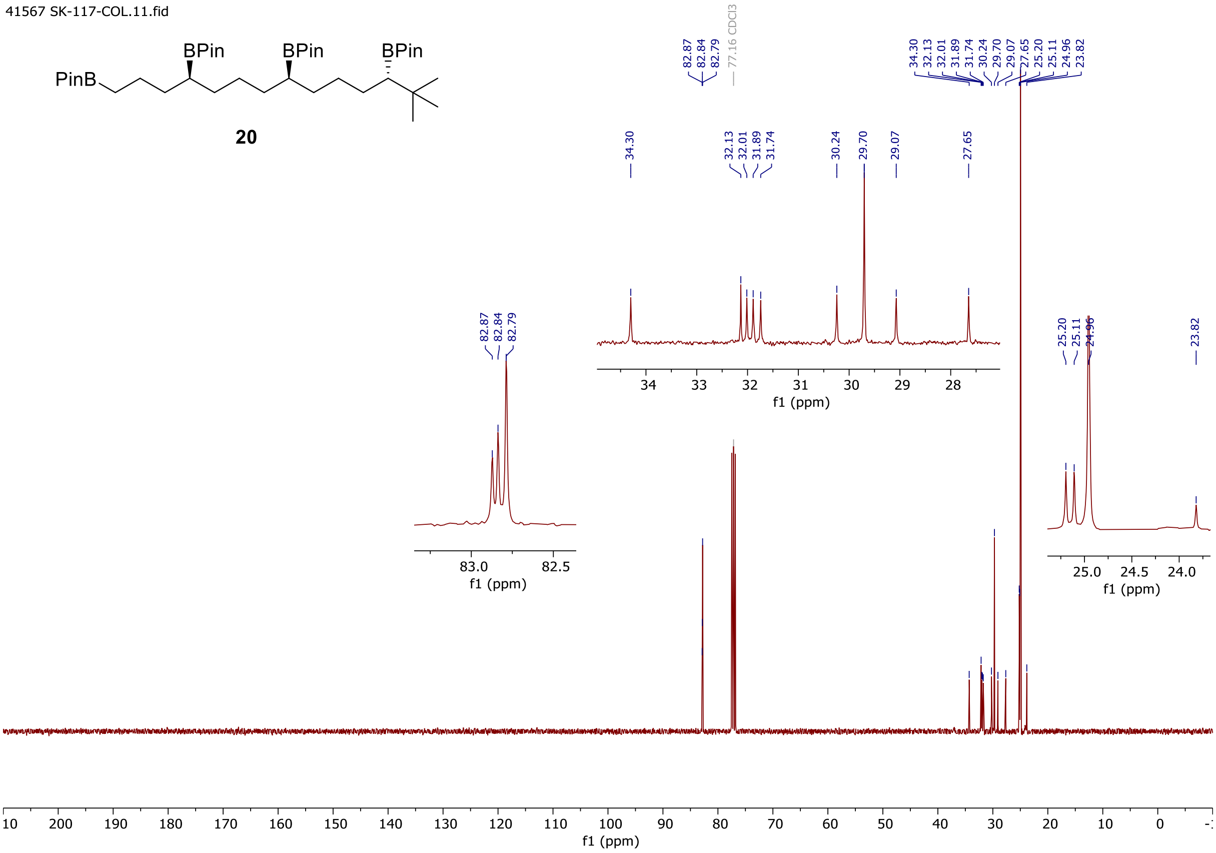

^{1}H NMR (400 MHz, $CDCl_3$) of compound 6. See procedure.

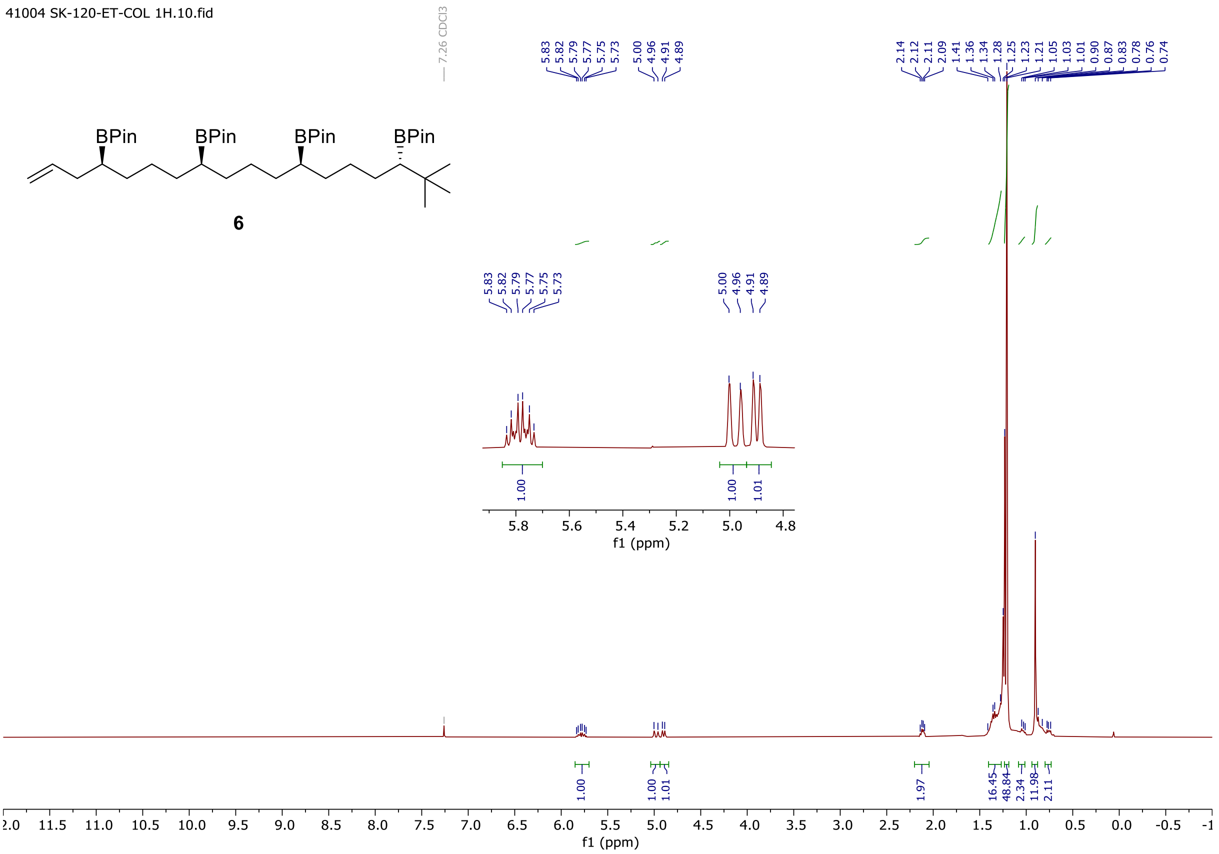

^{13}C NMR (101 MHz, $CDCl_3$) of compound 6. See procedure.

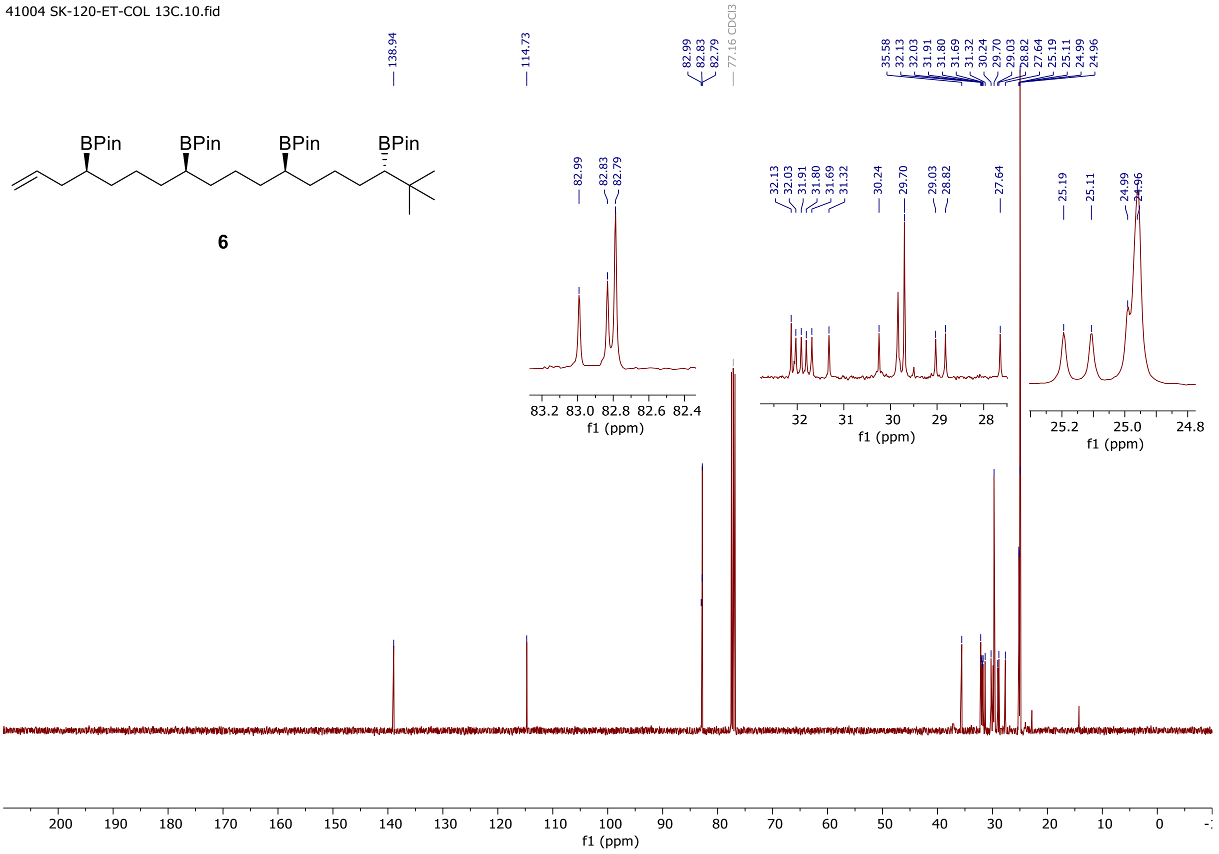

^{1}H NMR (500 MHz, CD_3OD) of compound 21. See procedure.

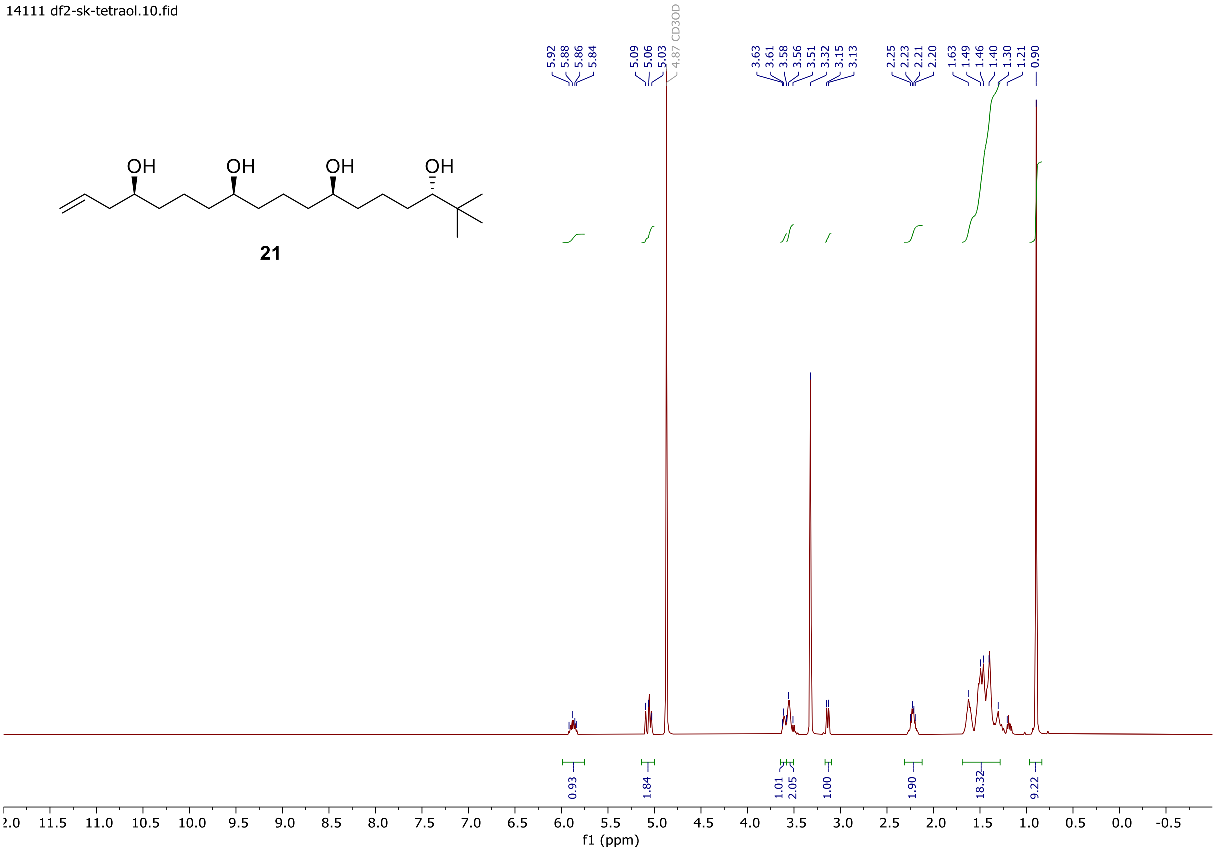

^{13}C NMR (126 MHz, CD_3OD) of compound 21 (*full spectrum*). See procedure.

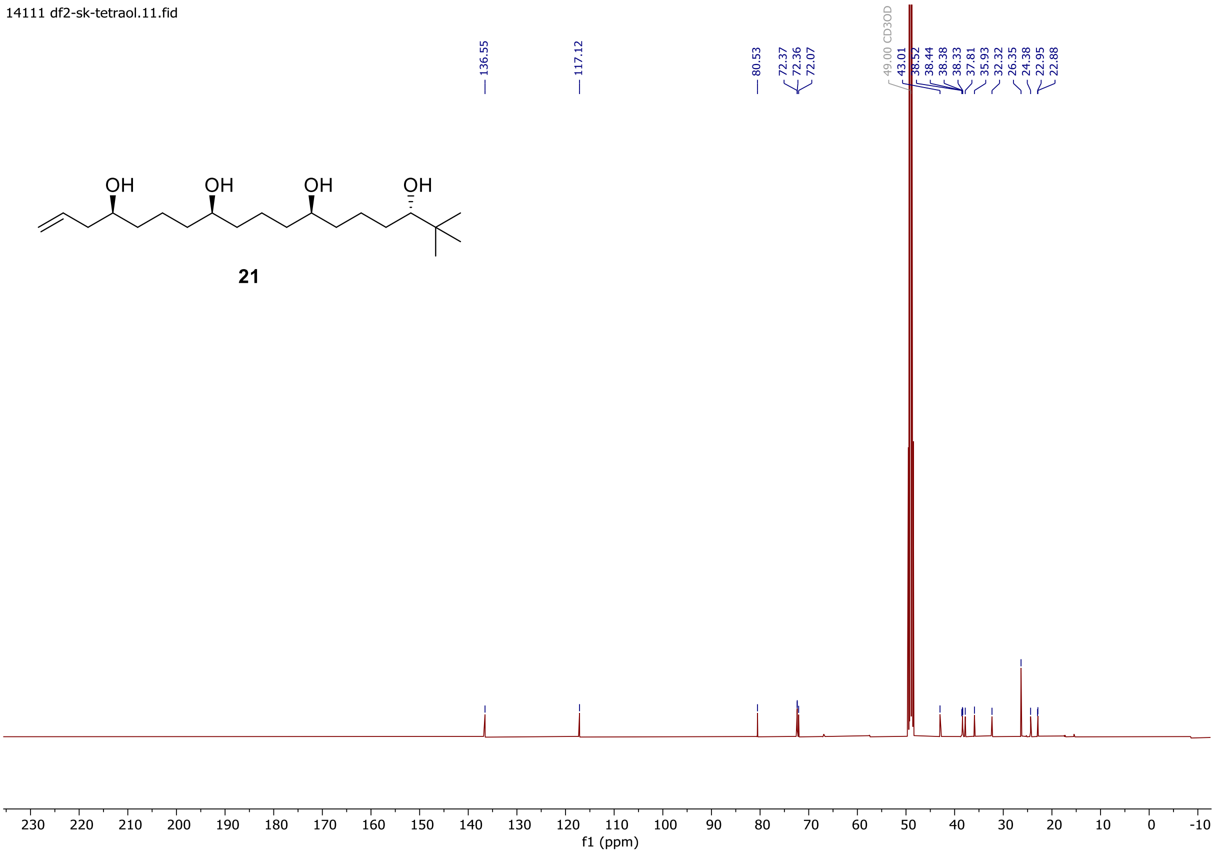

^{13}C NMR (126 MHz, CD_3OD) of compound 21 (*partial spectrum*).

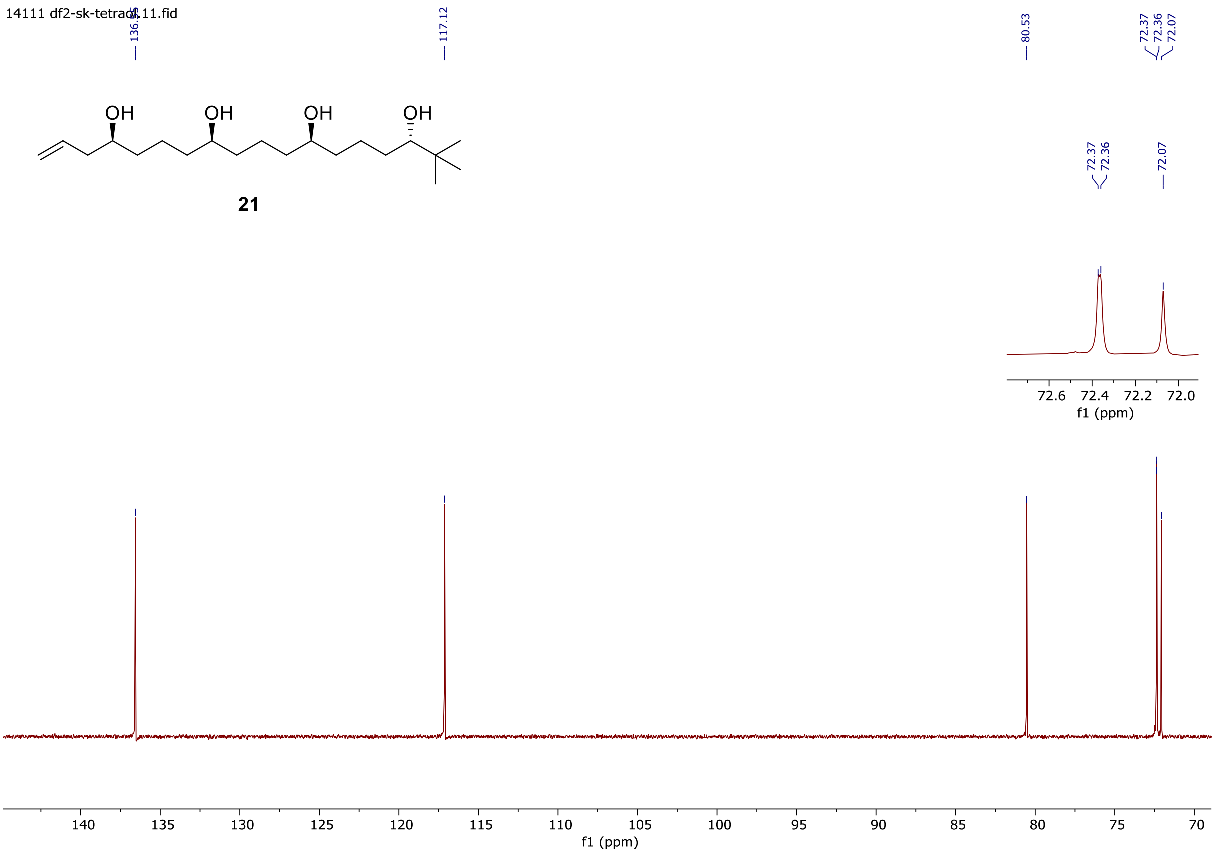

^{13}C NMR (126 MHz, CD_3OD) of compound 21 (*partial spectrum*).

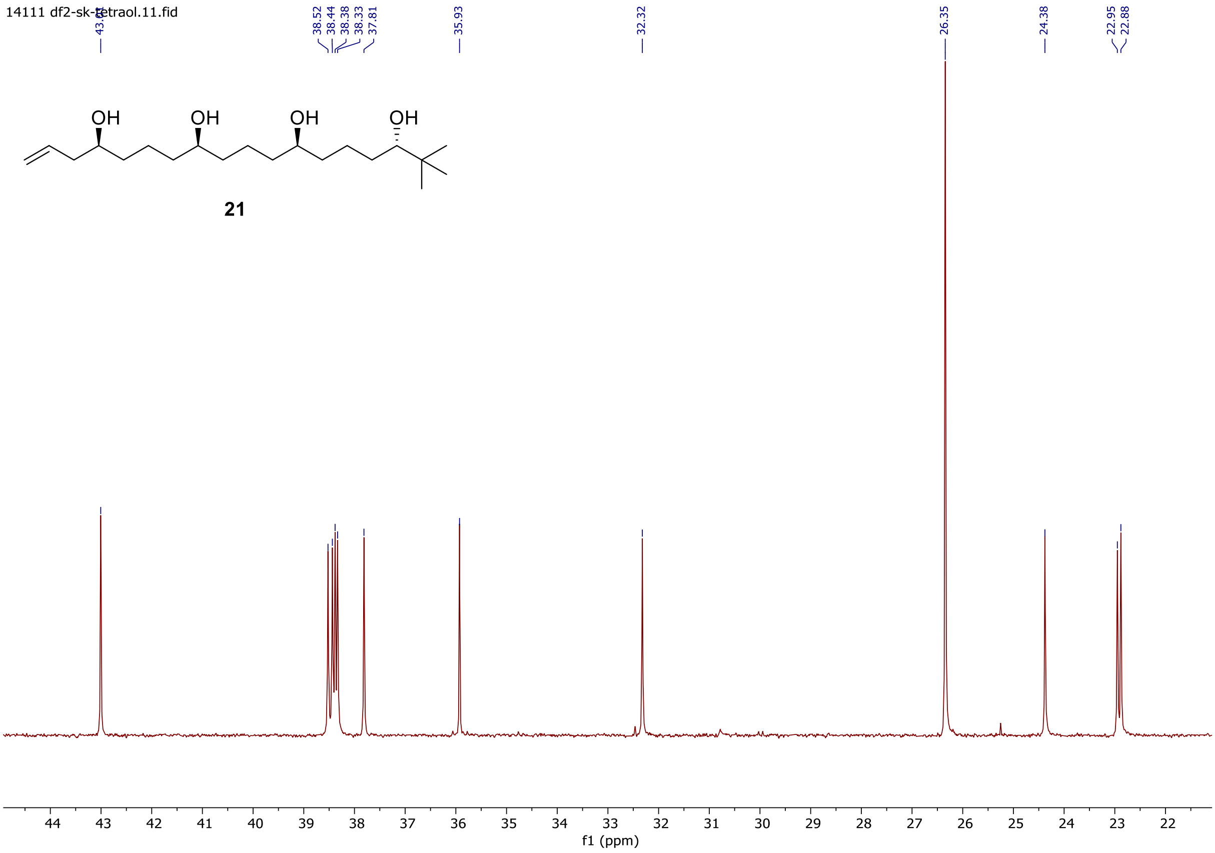

^{1}H NMR (500 MHz, Py-*d*5) of compound 21. See procedure.

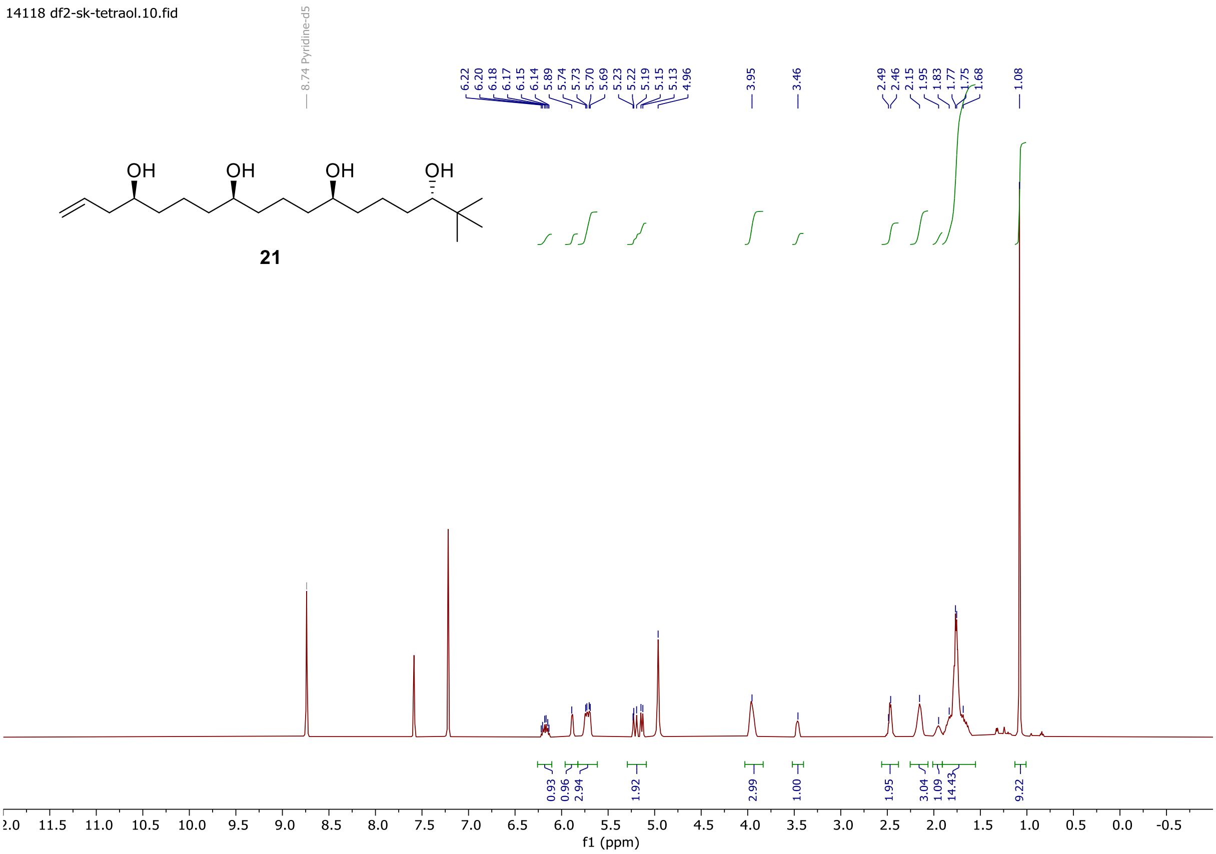

^{13}C NMR (126 MHz, Py-*d*5) of compound 21 (*full spectrum*). See procedure.

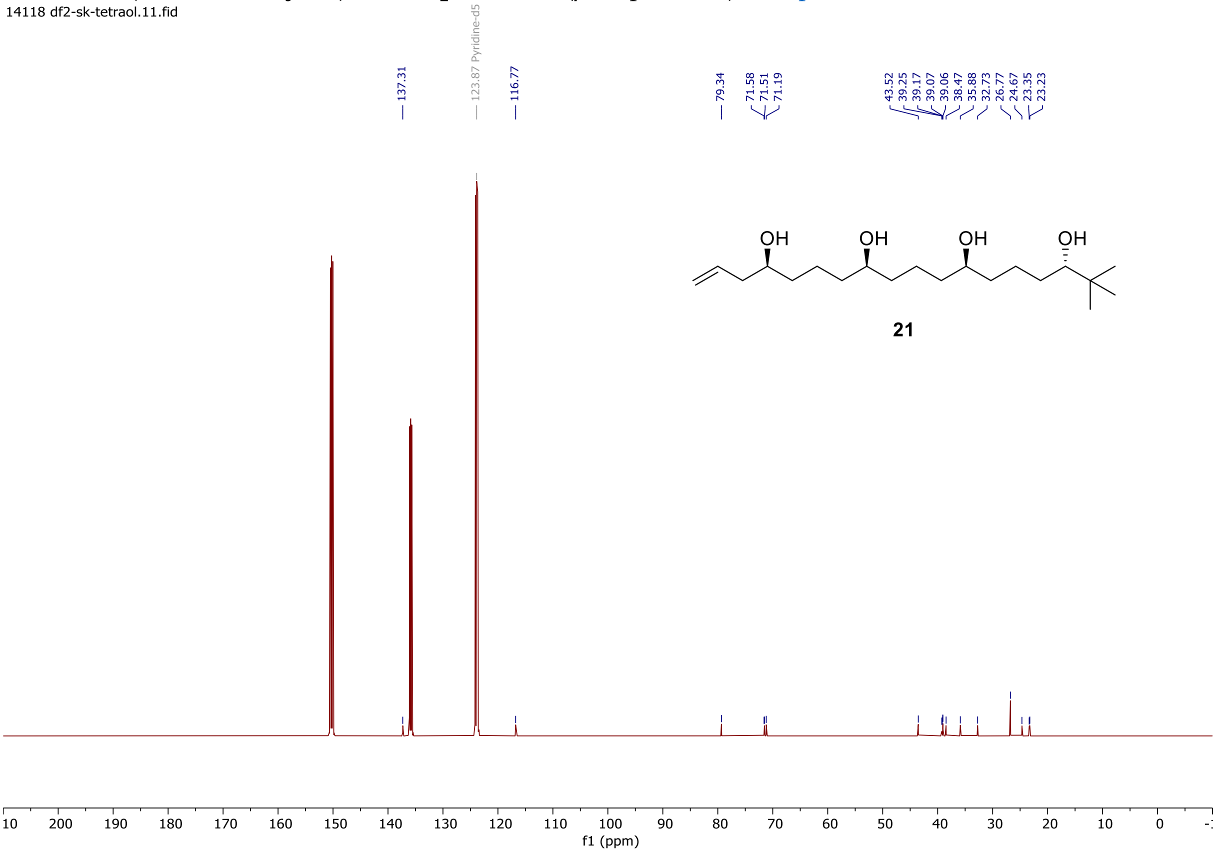

^{13}C NMR (126 MHz, Py-*d*5) of compound 21 (*partial spectrum*).

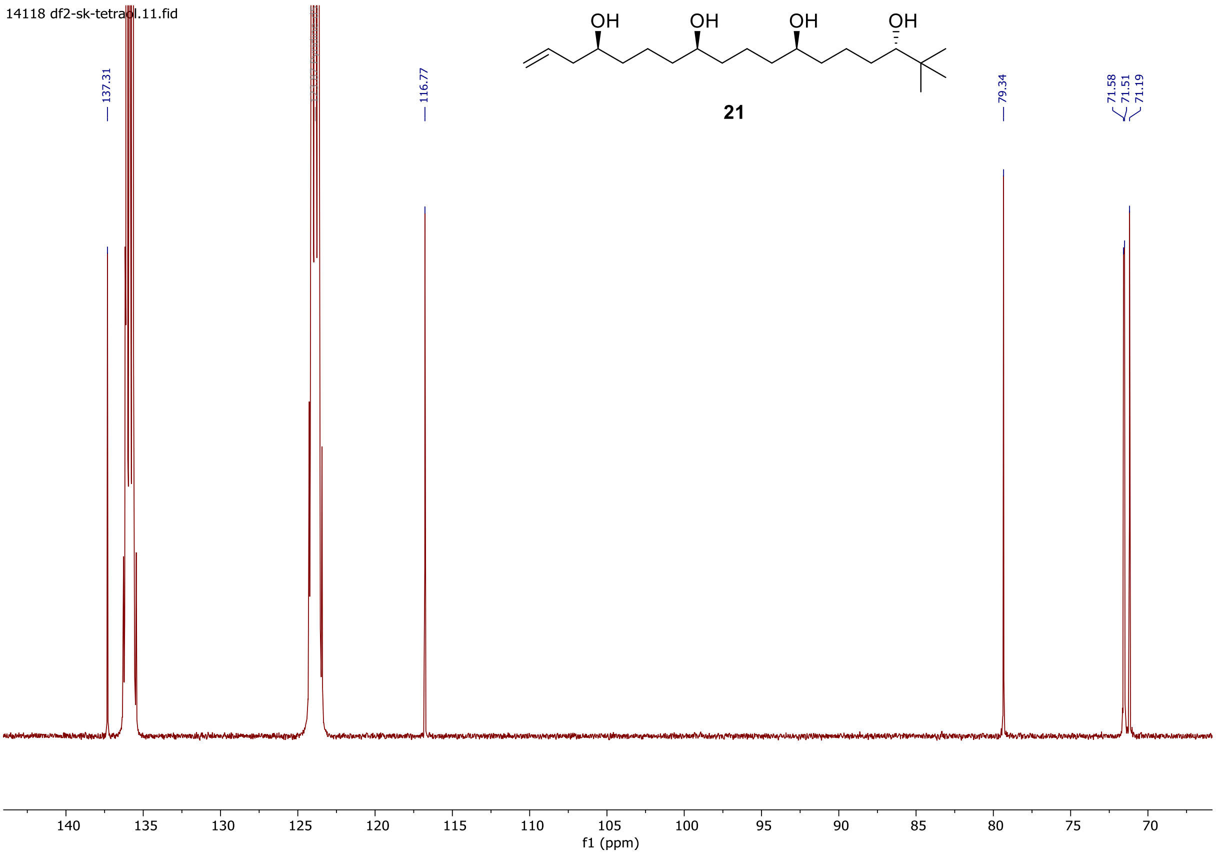

^{13}C NMR (126 MHz, Py-*d*5) of compound 21 (*partial spectrum*).

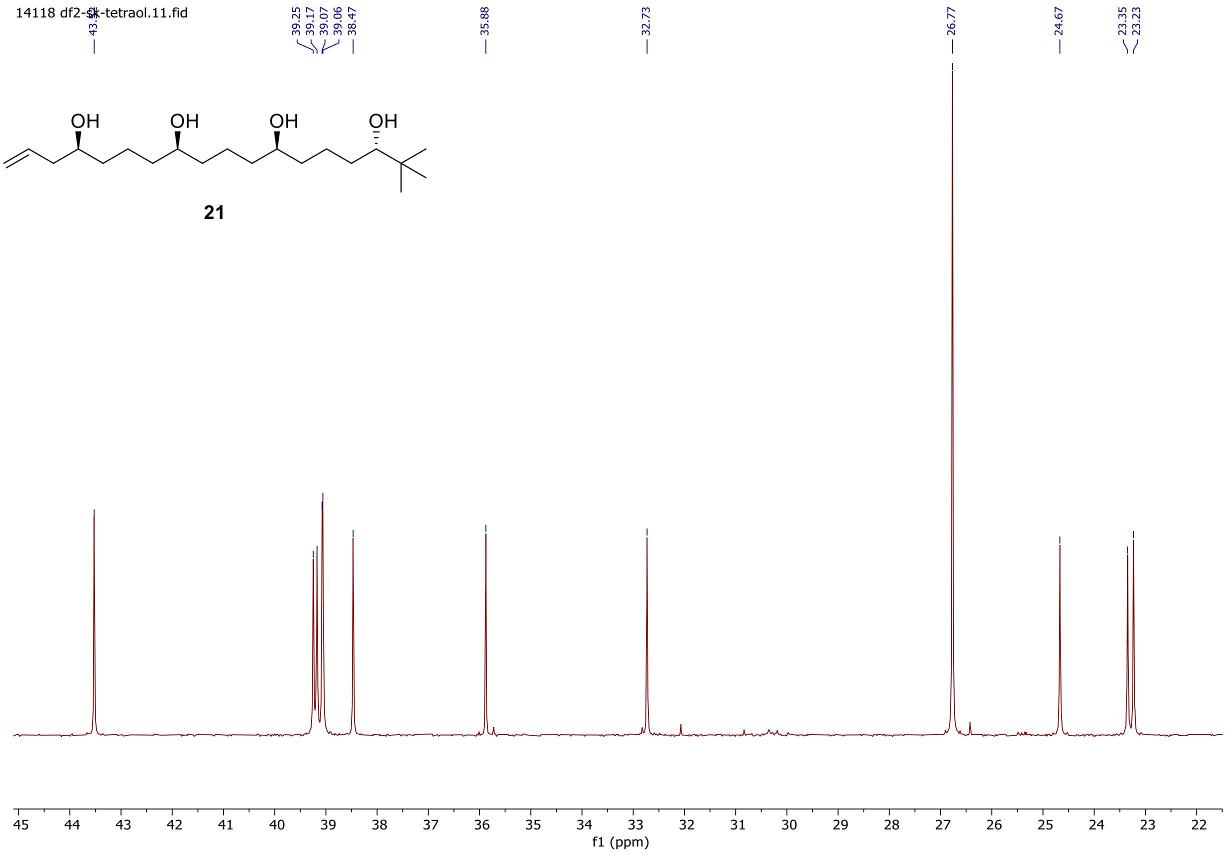

HSQC (500 MHz (^{1}H), 126 MHz (^{13}C), py-*d*5) of compound 21 (*aliased to 35 ppm centred at 32.5 ppm*). [*See full chemical shift assignment.*]

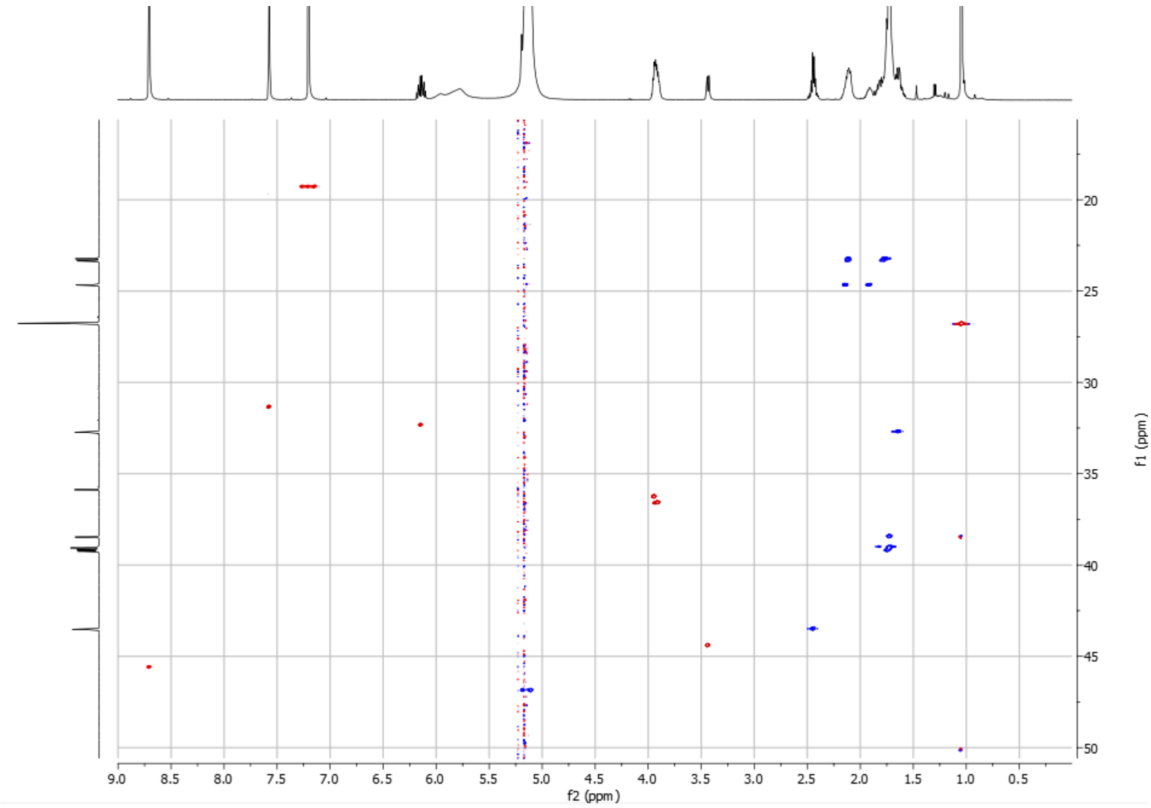

HSQC-TOCSY (30 ms mixing time) (500 MHz (^{1}H), 126 MHz (^{13}C), py-*d*5) of compound 21 (*aliased to 35 ppm centred at 32.5 ppm)*

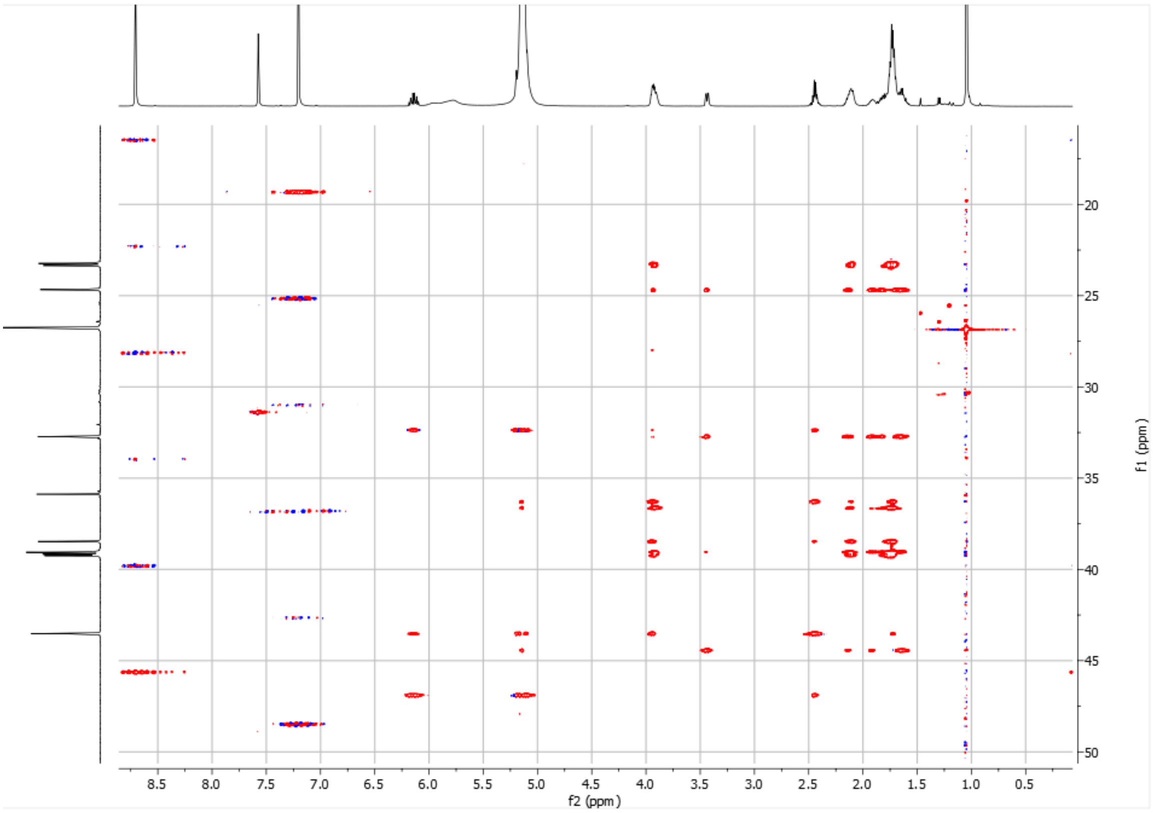

HSQC-TOCSY (60 ms mixing time) (500 MHz (^{1}H), 126 MHz (^{13}C), py-*d*5) of compound 21

(aliased to 35 ppm centred at 32.5 ppm)

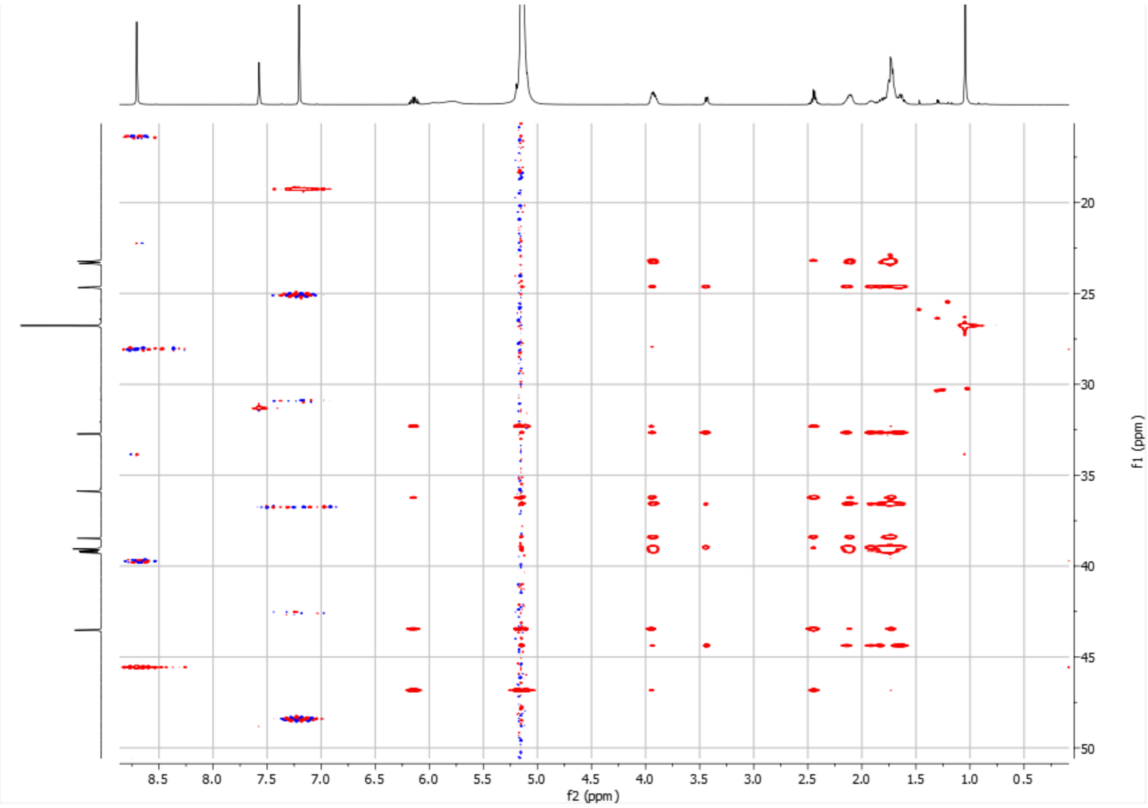

^{1}H NMR (500 MHz, $CDCl_3$) of compound 22. *See procedure.*

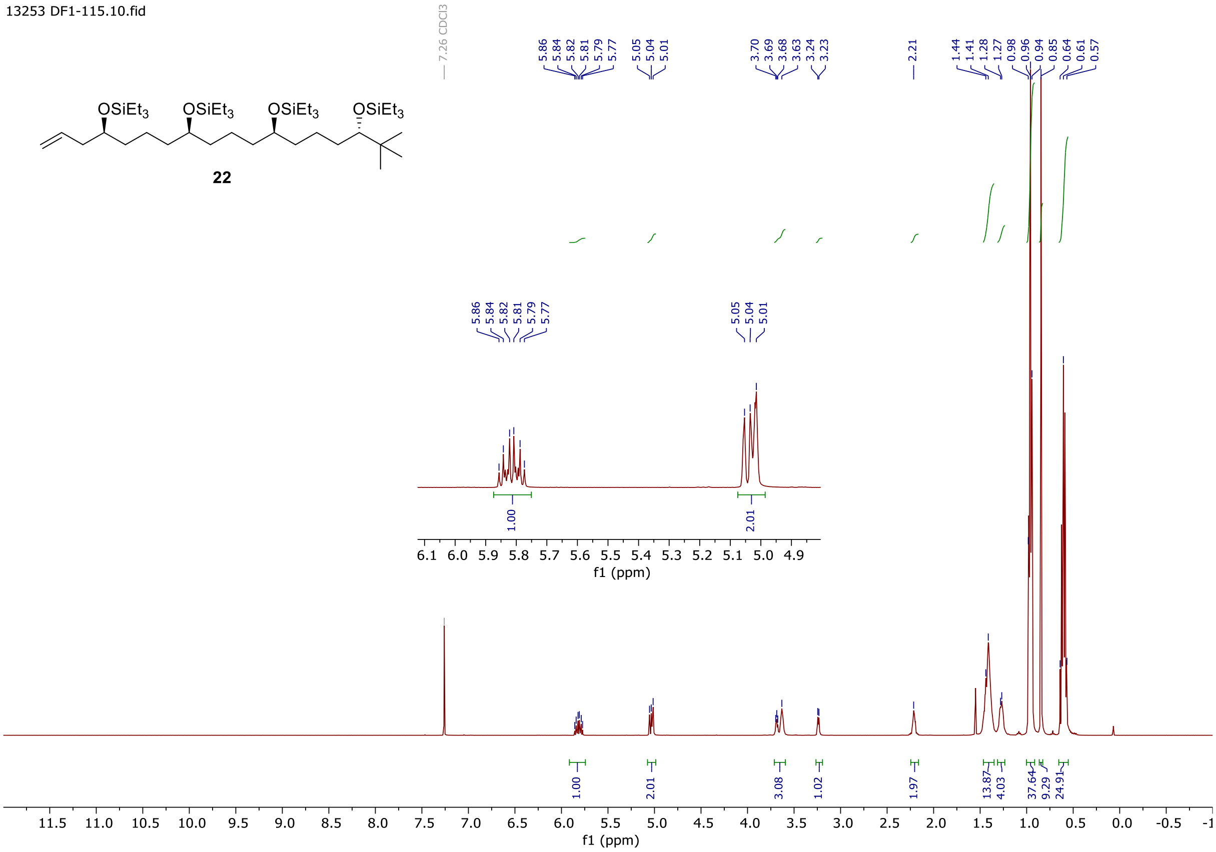

^{13}C NMR (126 MHz, $CDCl_3$) of compound 22. *See procedure.*

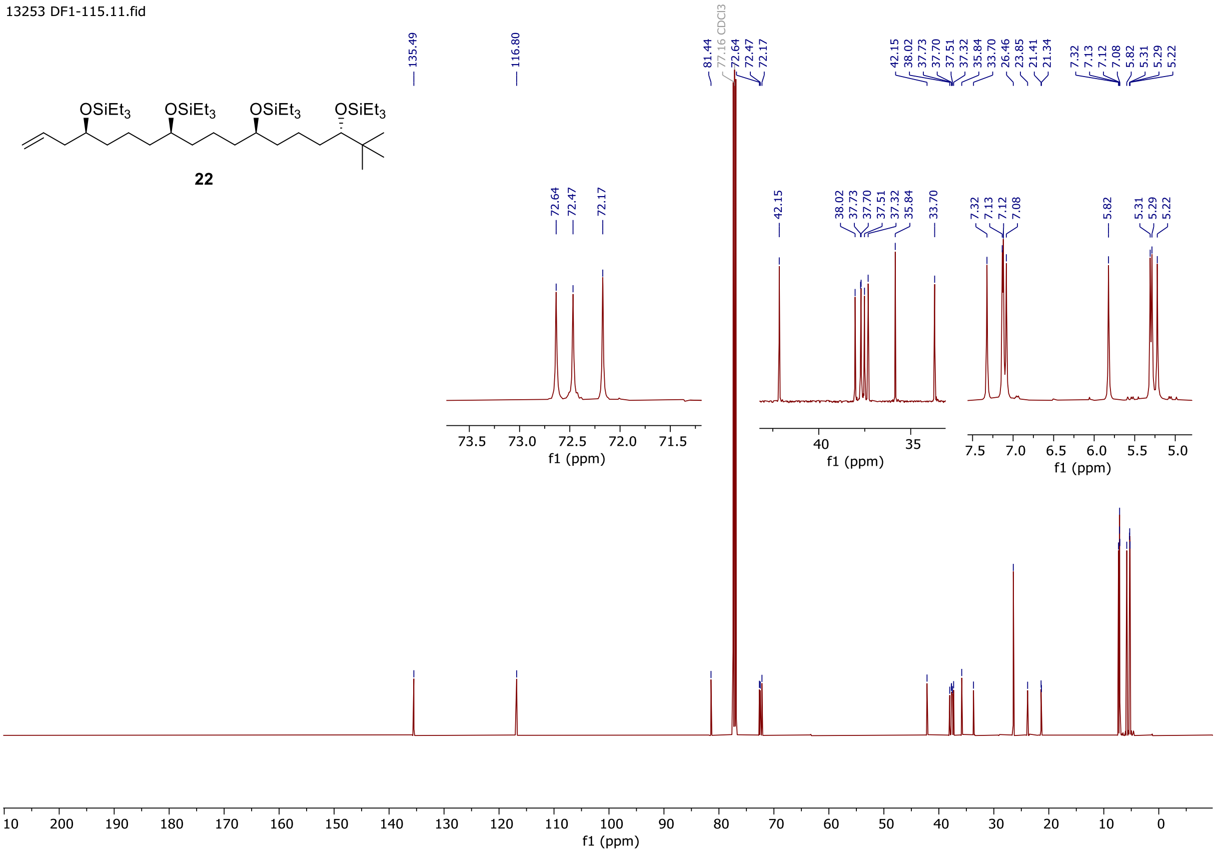

^{1}H NMR (500 MHz, CDCl$_3$) of compound 4 (*full spectrum*). See procedure.

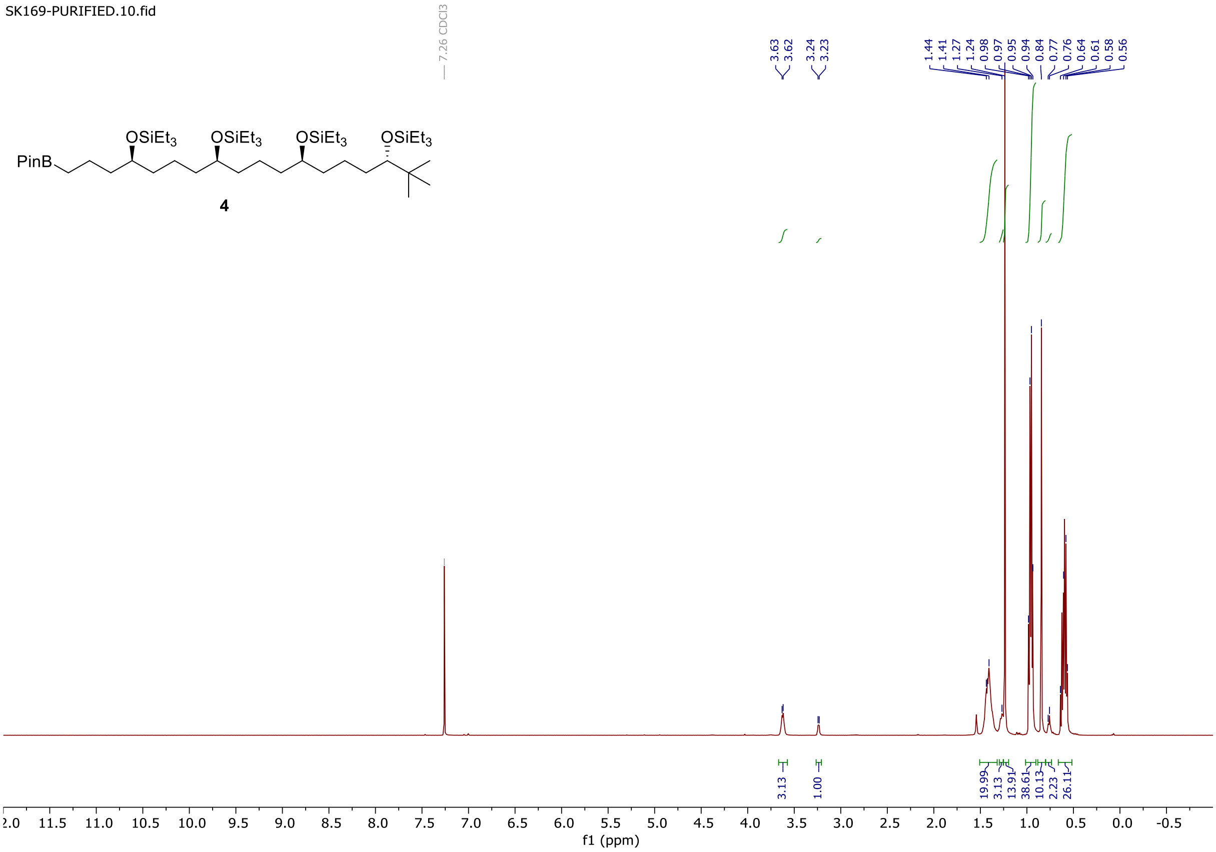

^{1}H NMR (500 MHz, CDCl$_3$) of compound 4 (*partial spectrum*).

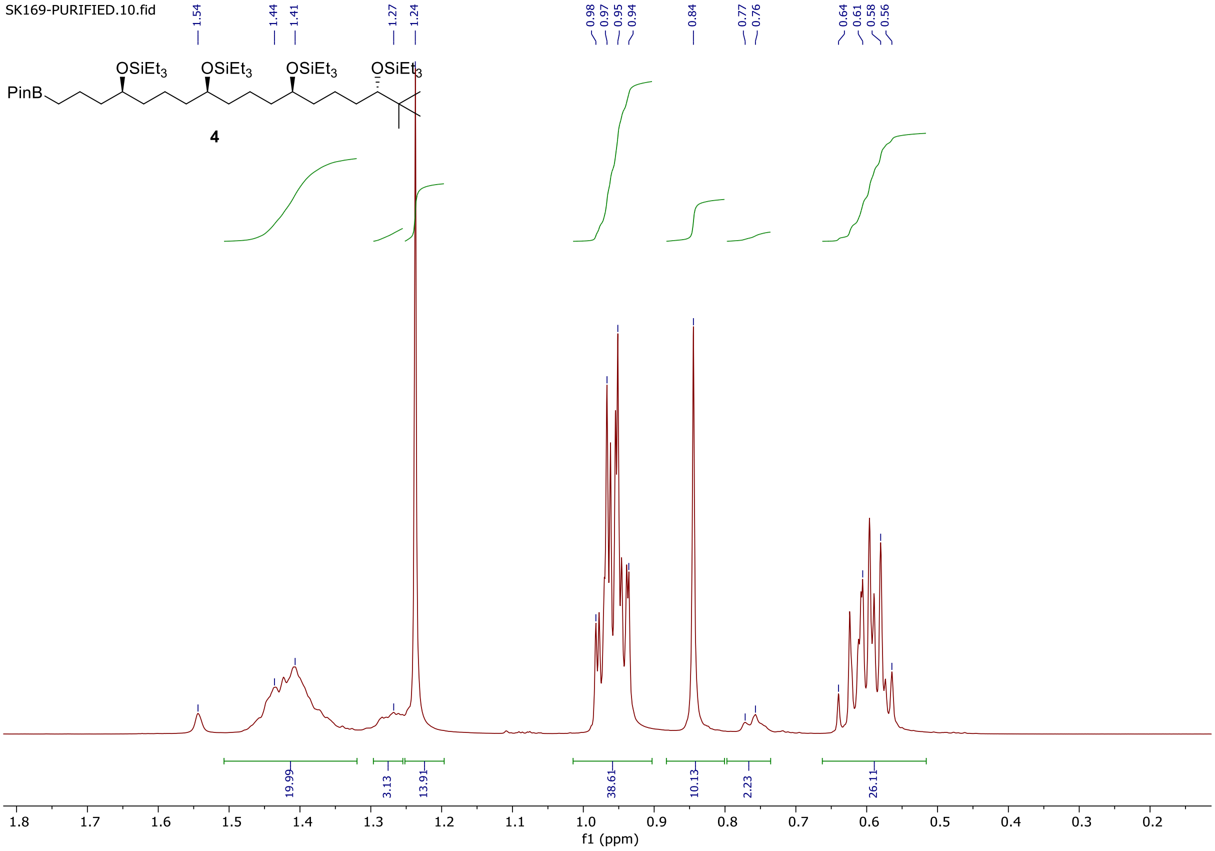

^{13}C NMR (126 MHz, CDCl$_3$) of compound 4 (*full spectrum*). See procedure.

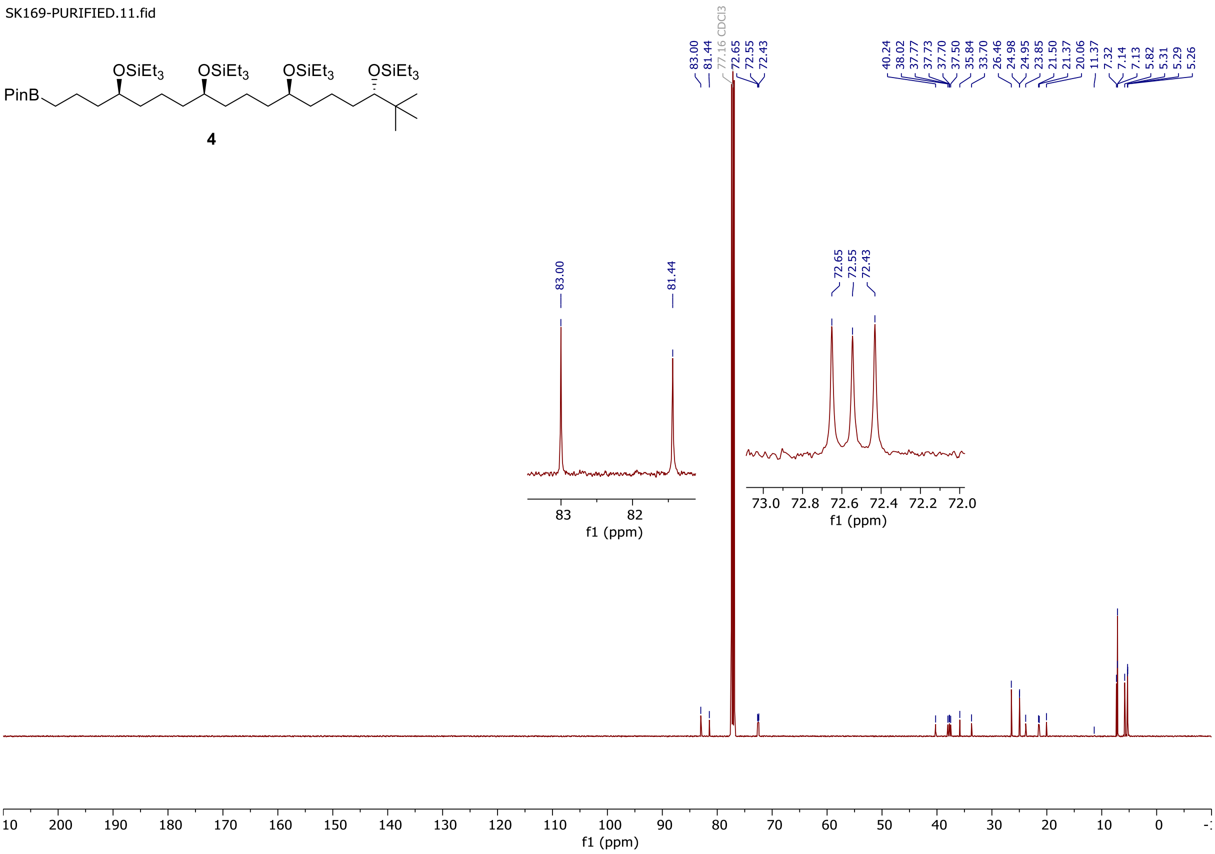

^{13}C NMR (126 MHz, CDCl$_3$) of compound 4 (*partial spectrum*).

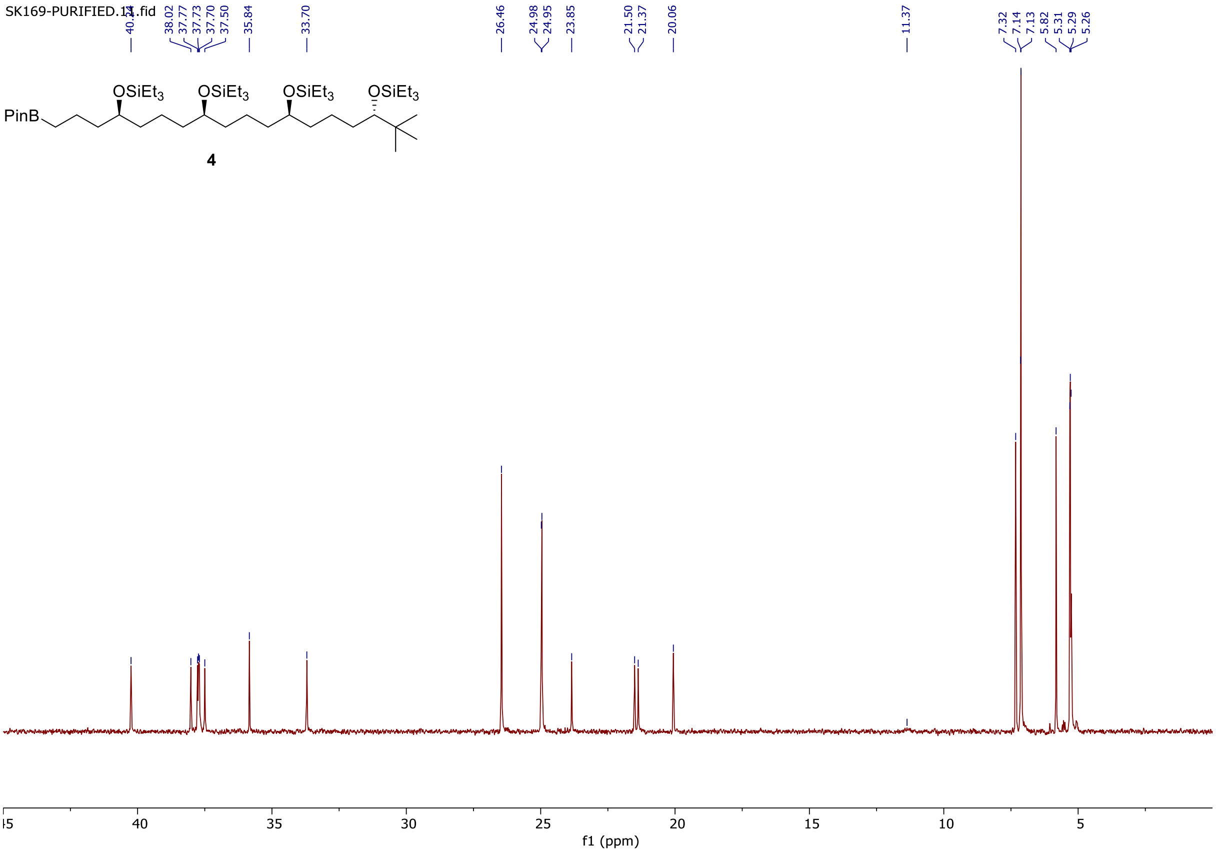

^{1}H NMR (400 MHz, $CDCl_3$) of compound 23. *See procedure*.

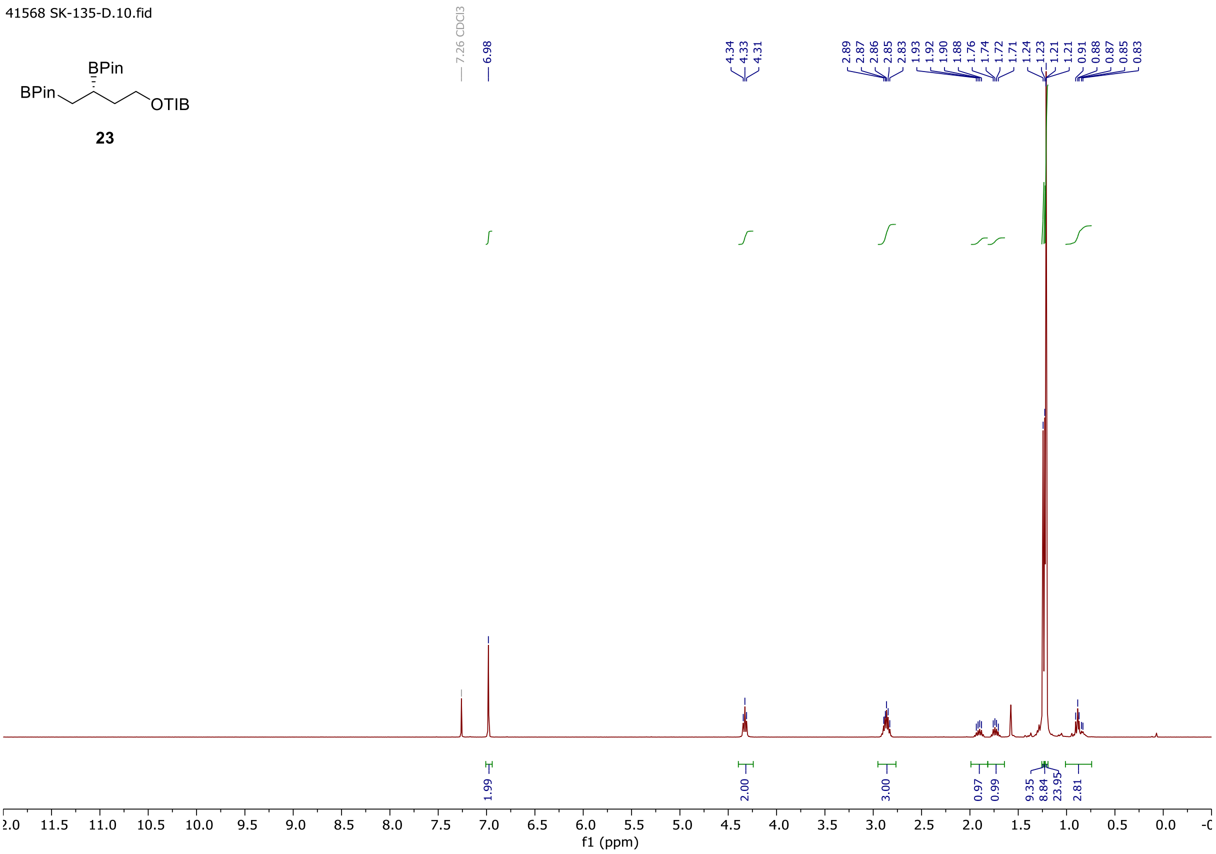

^{13}C NMR (101 MHz, $CDCl_3$) of compound 23. *See procedure.*

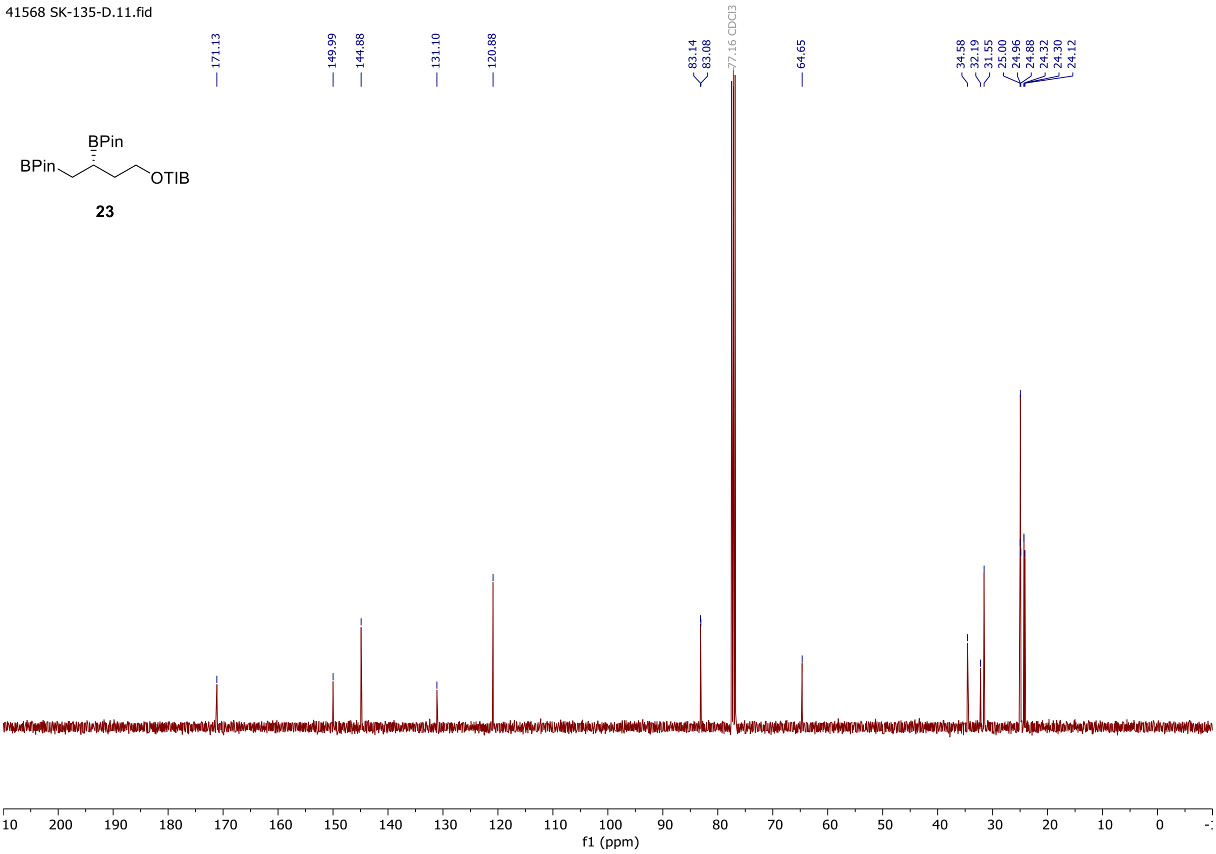

^{1}H NMR (400 MHz, $CDCl_3$) of compound 23-ox. See procedure.

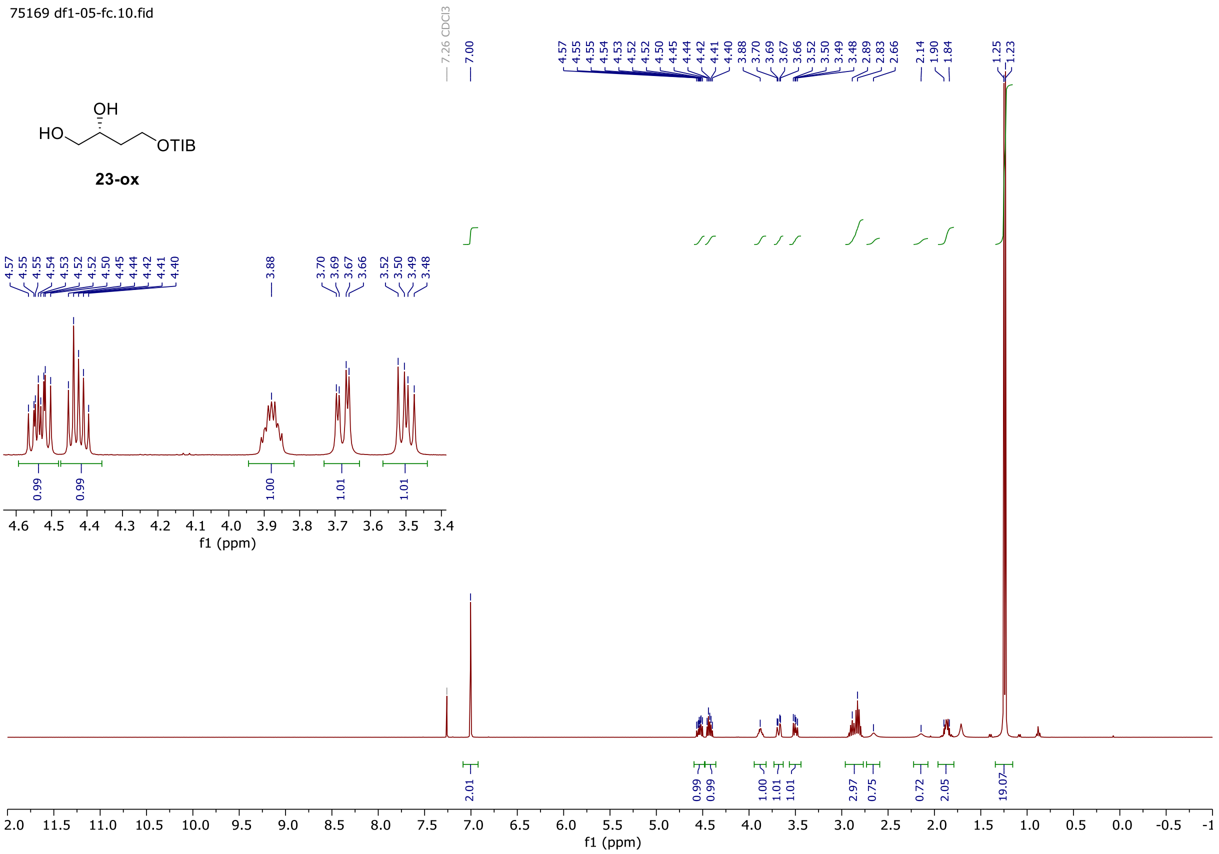

^{13}C NMR (101 MHz, $CDCl_3$) of compound 23-ox. See procedure.

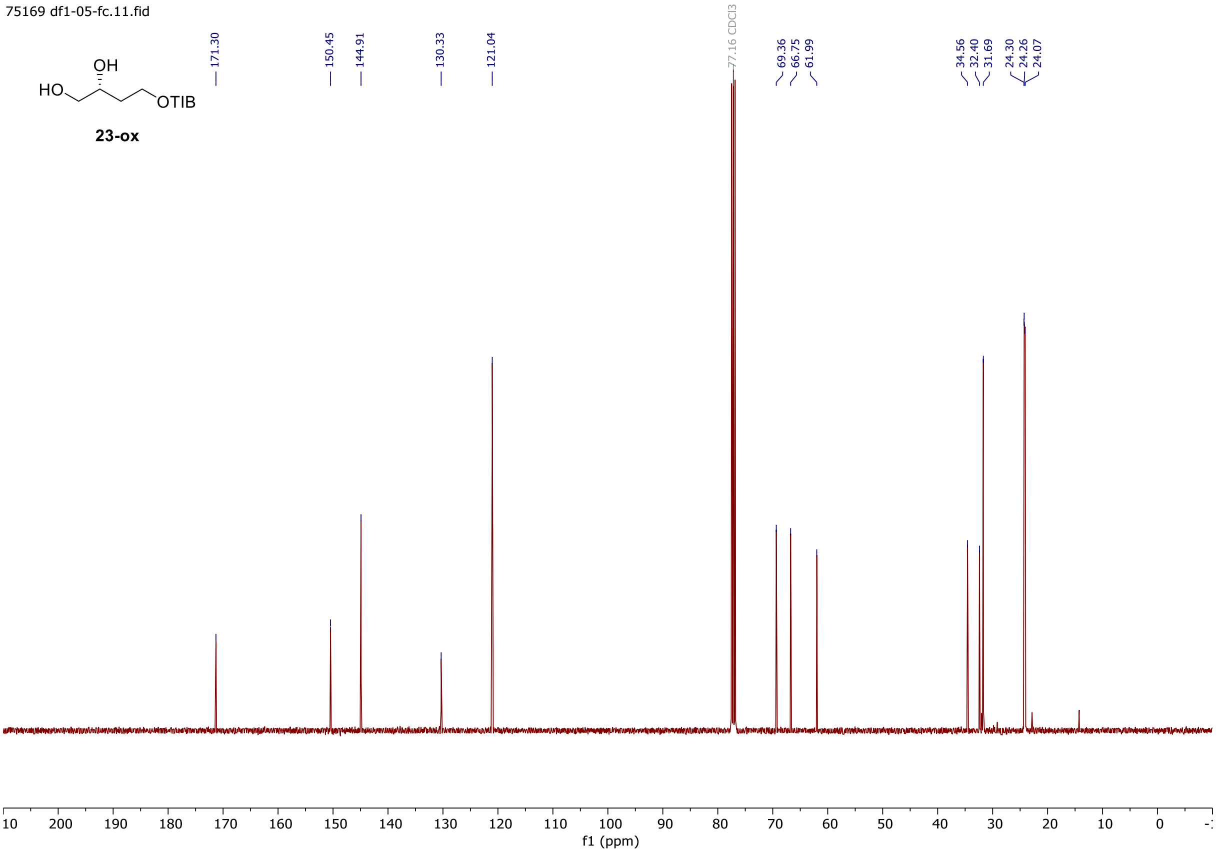

^{1}H NMR (400 MHz, $CDCl_3$) of compound 24. See procedure.

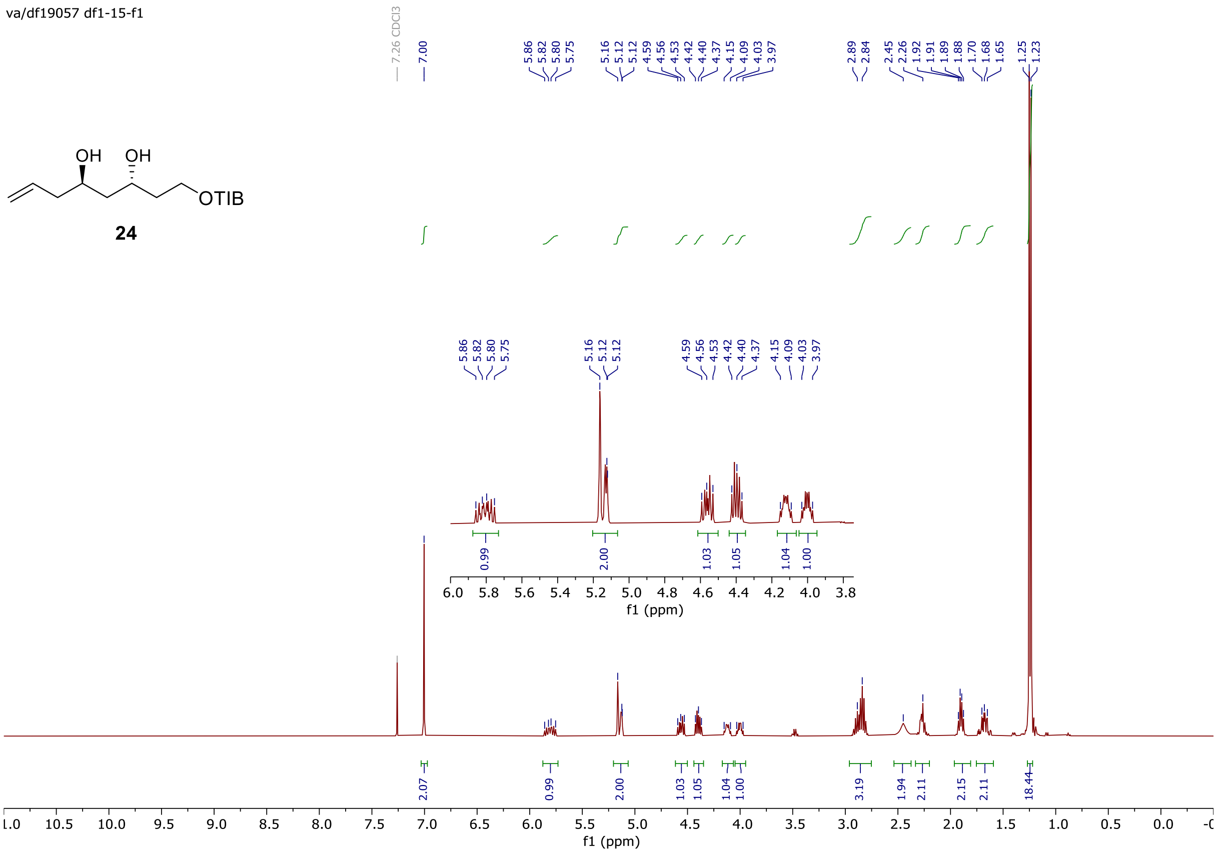

^{13}C NMR (101 MHz, $CDCl_3$) of compound 24. See procedure.

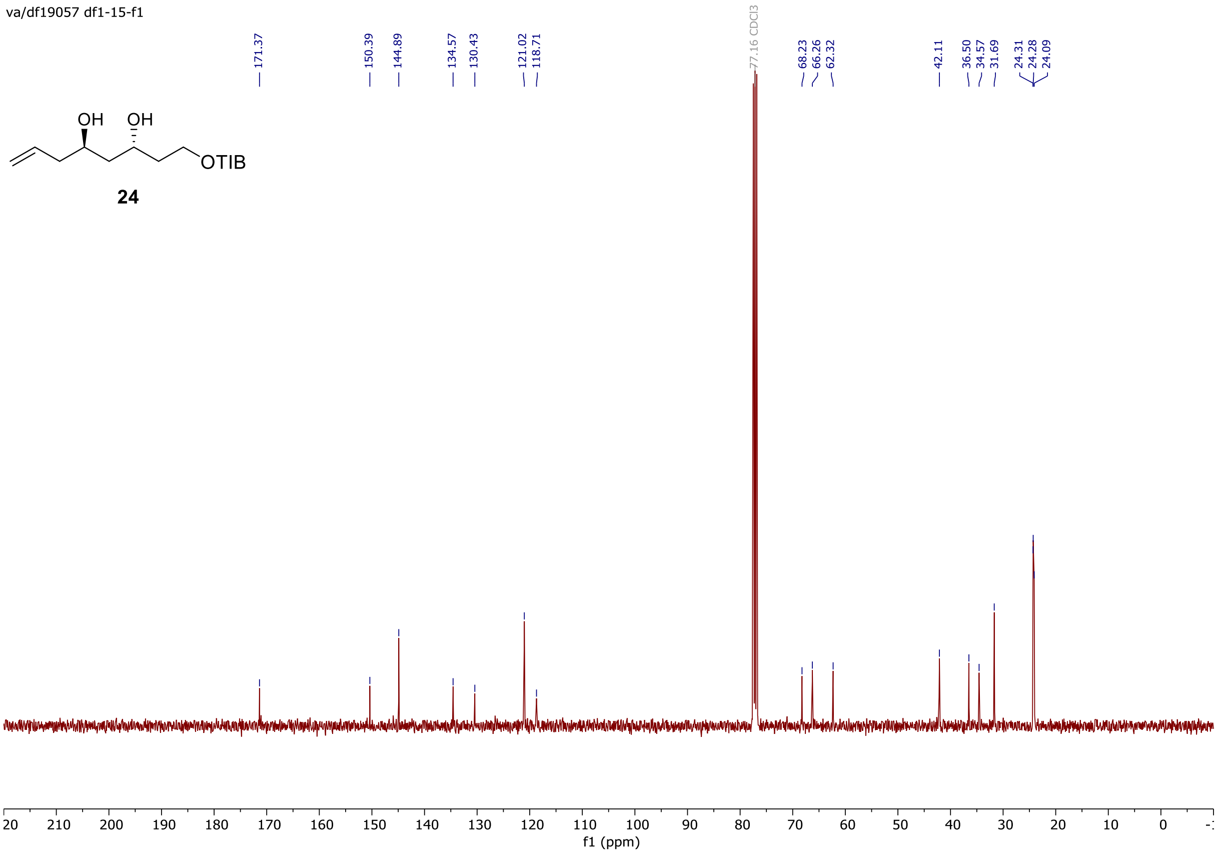

^{1}H NMR (400 MHz, $CDCl_3$) of compound 25. See procedure.

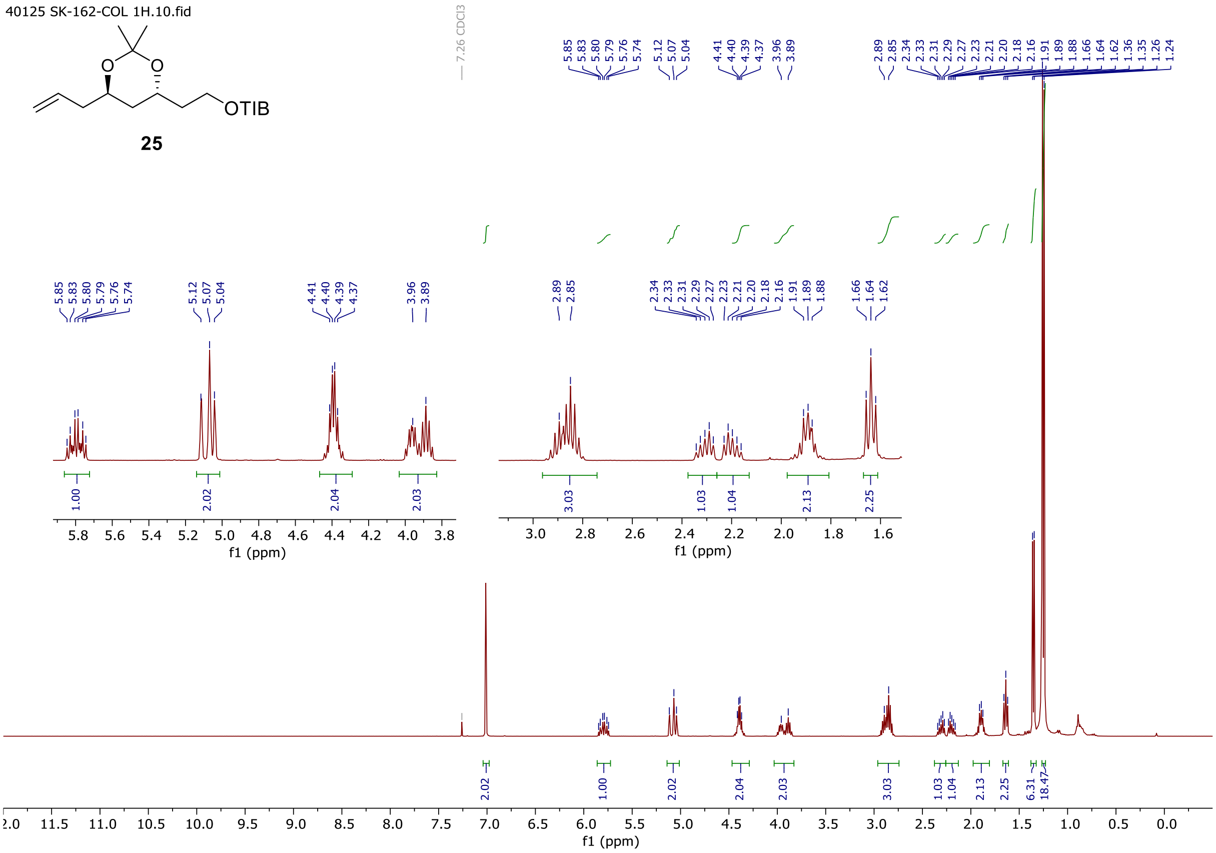

^{13}C NMR (101 MHz, $CDCl_3$) of compound 25. See procedure.

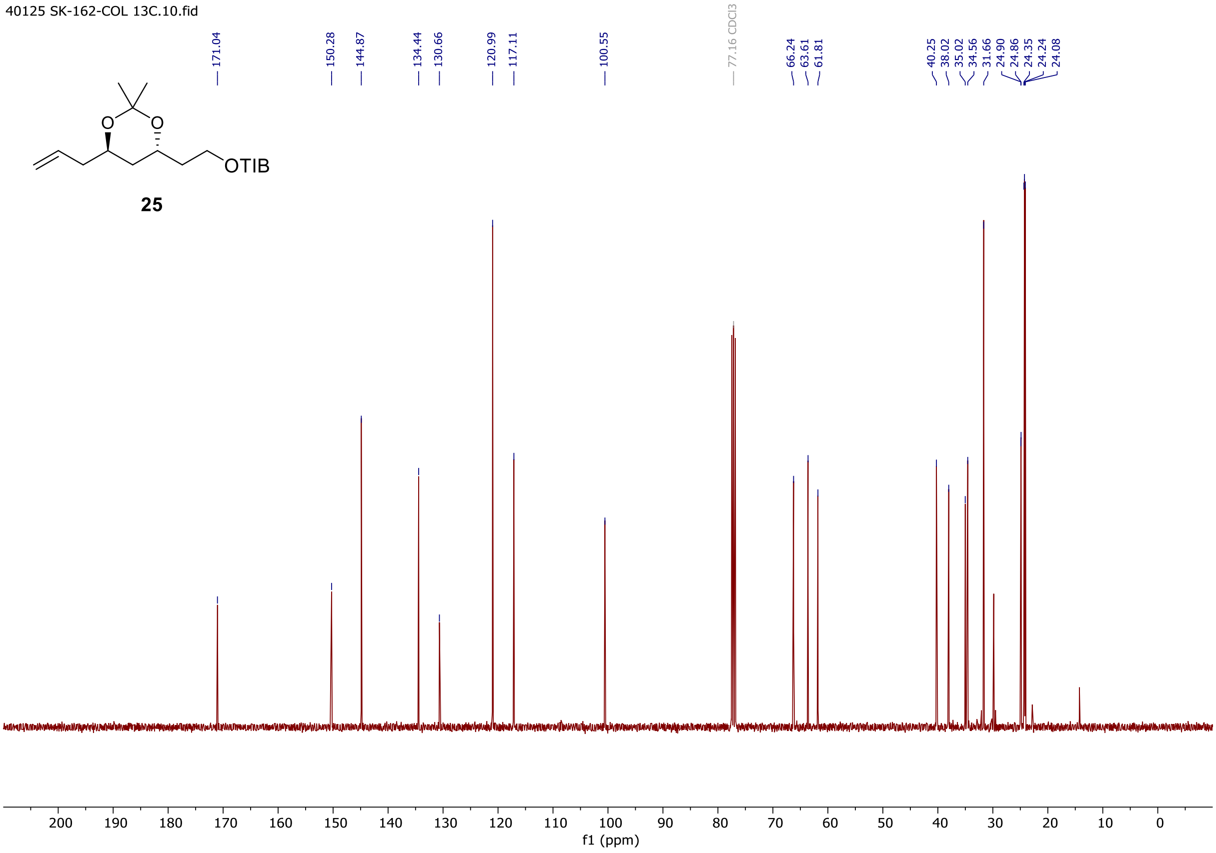

^{1}H NMR (400 MHz, CD_2Cl_2) of compound 26. See procedure.

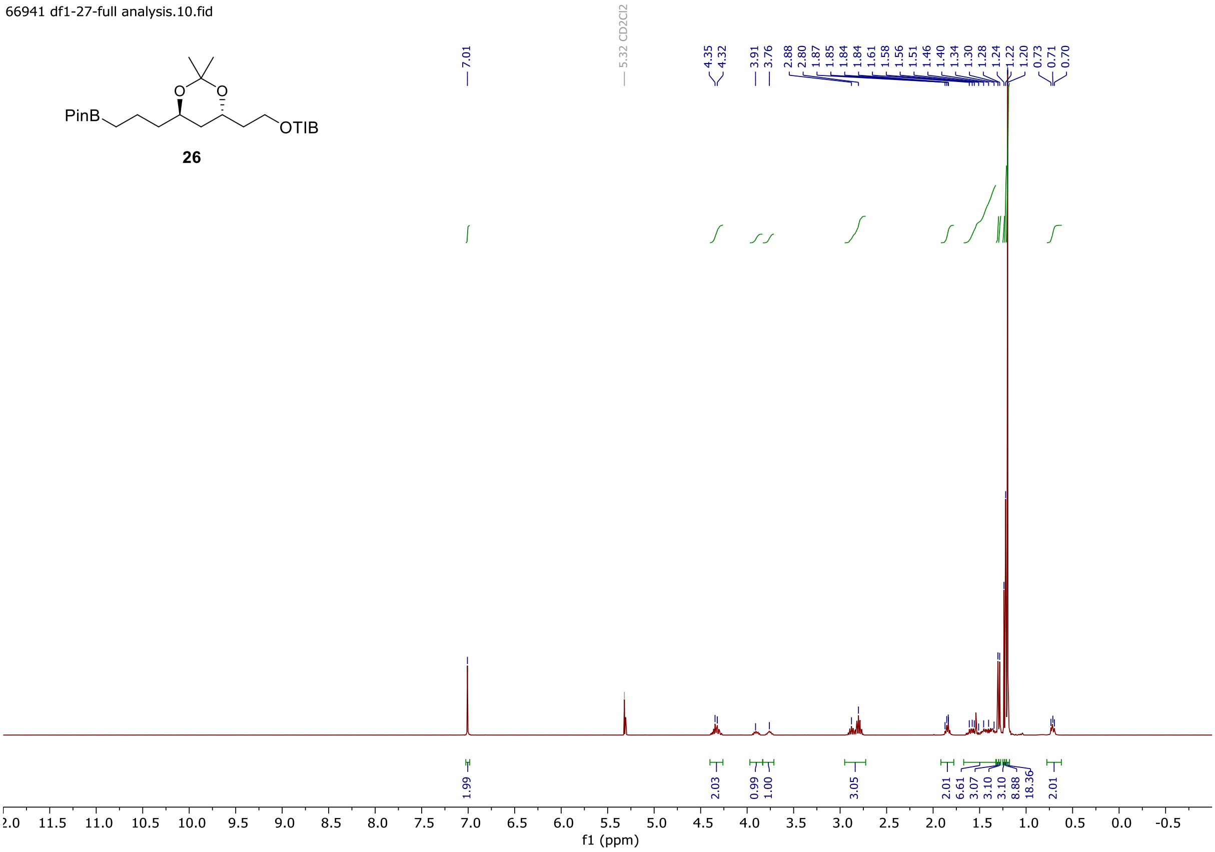

^{13}C NMR (101 MHz, CD_2Cl_2) of compound 26. See procedure.

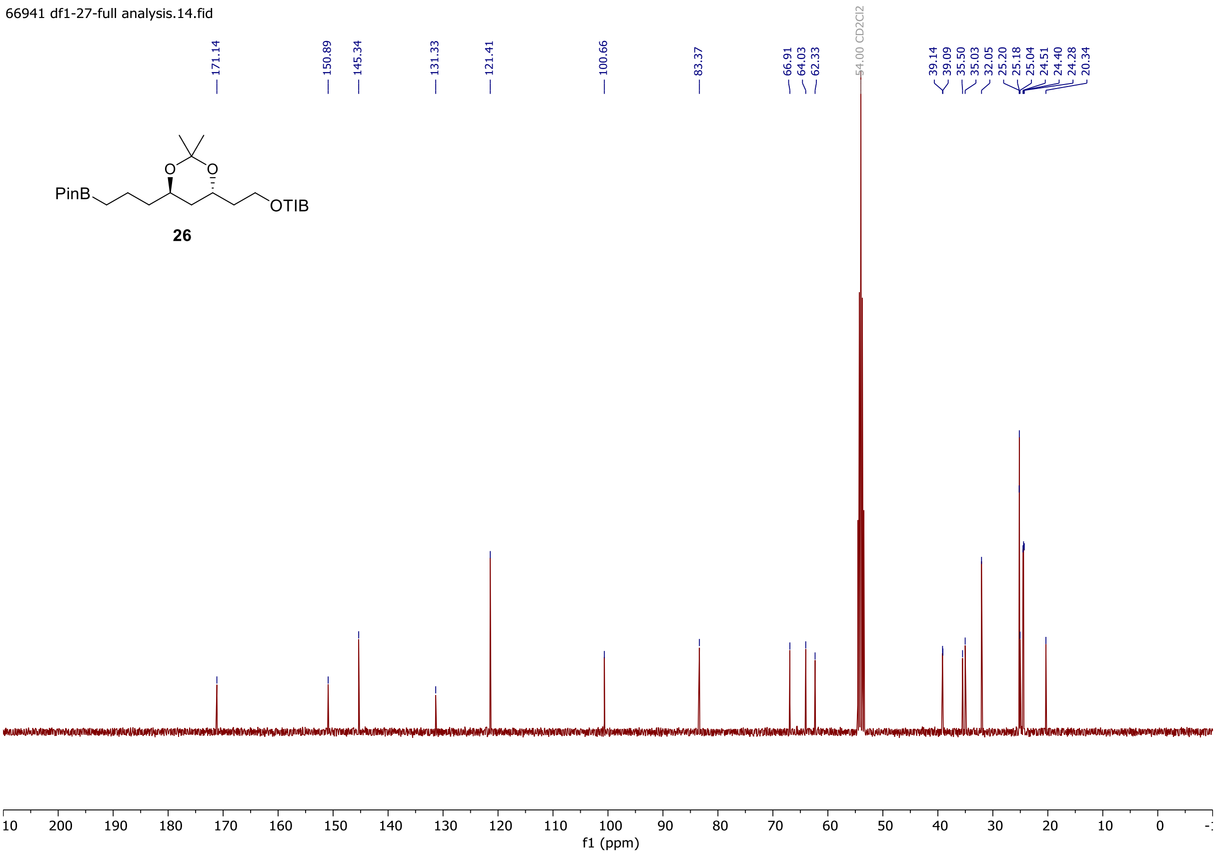

^{1}H NMR (400 MHz, $CDCl_3$) of compound 11. See procedure.

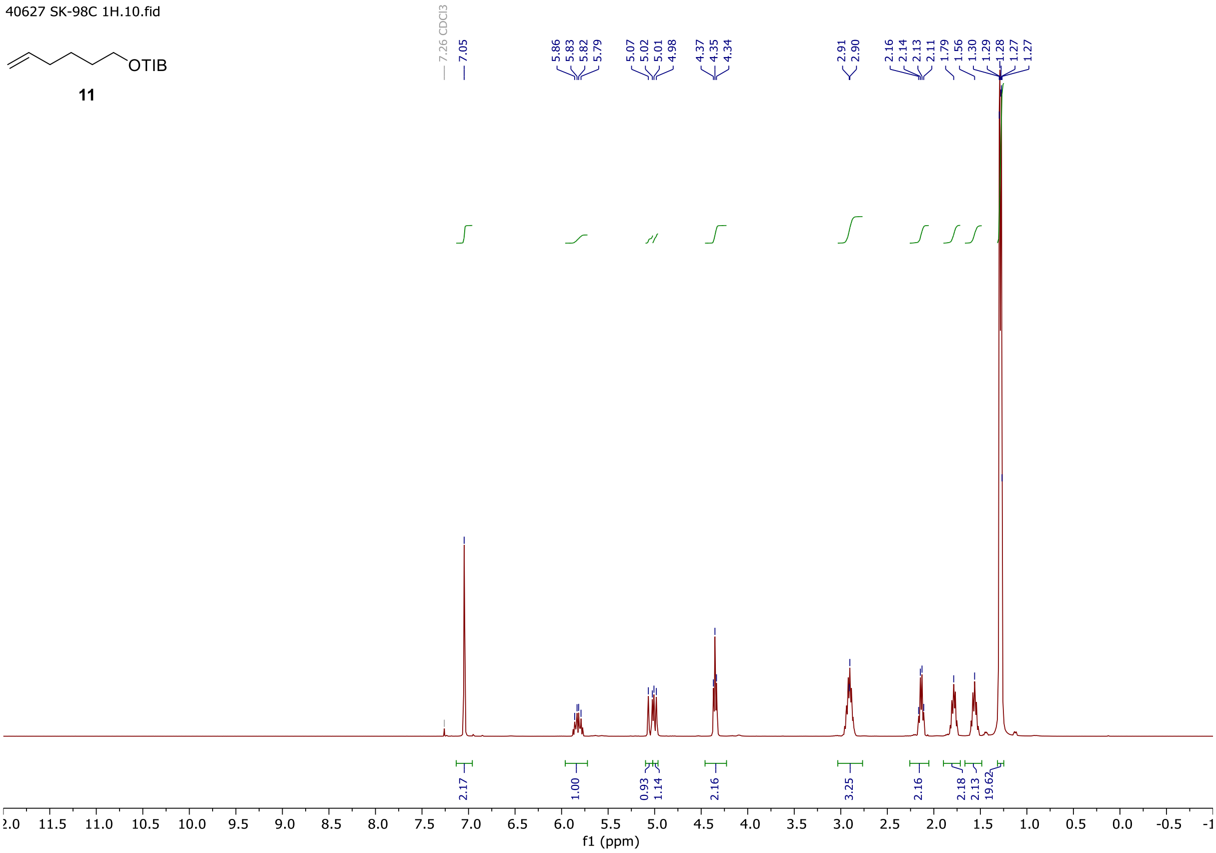

^{13}C NMR (101 MHz, $CDCl_3$) of compound 11. See procedure.

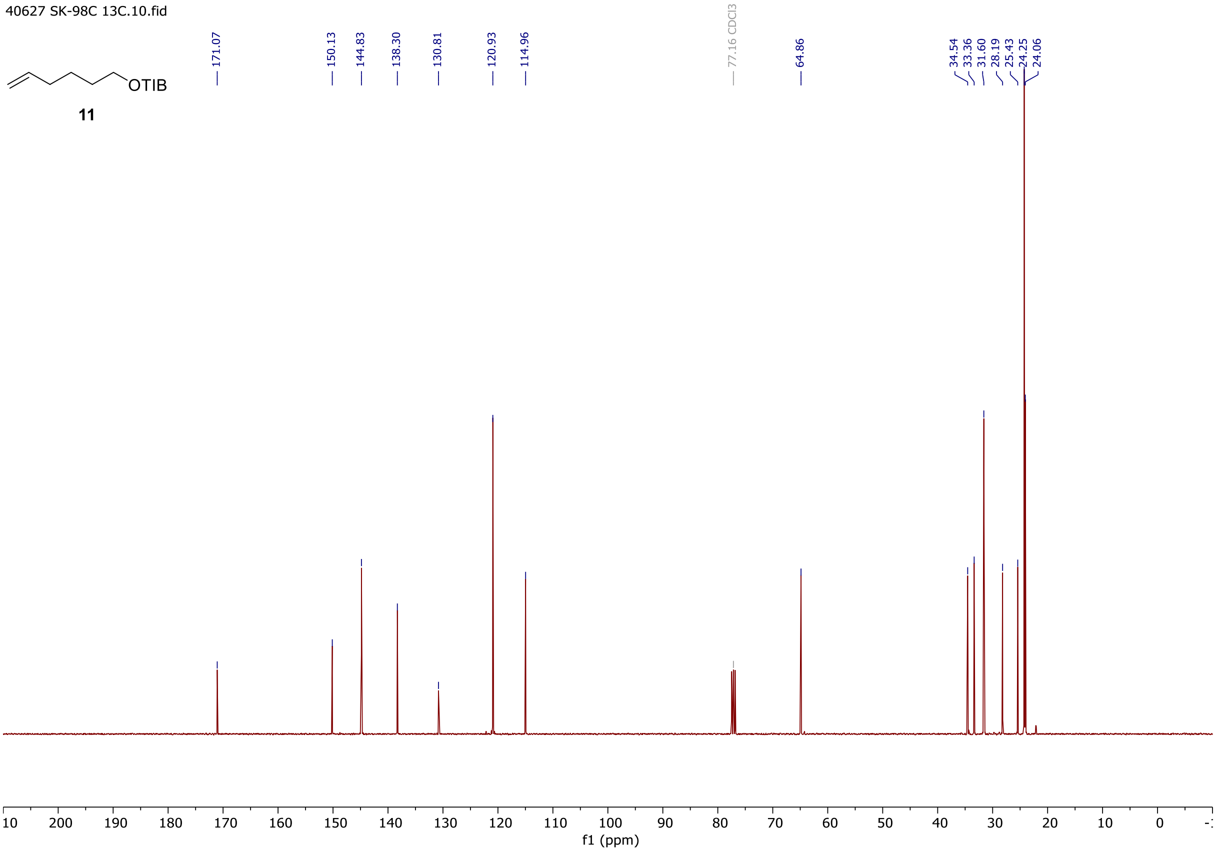

^{1}H NMR (400 MHz, $CDCl_3$) of compound 9. See procedure.

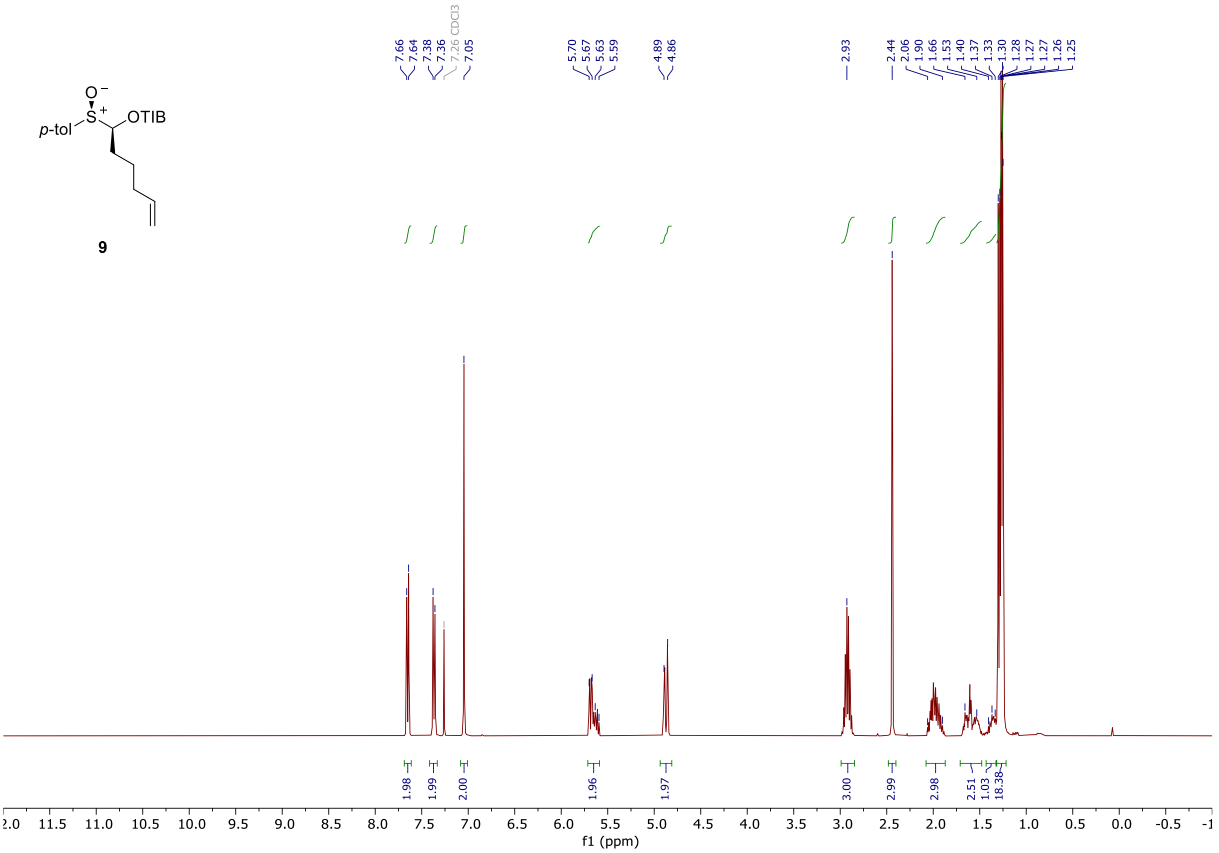

^{13}C NMR (101 MHz, $CDCl_3$) of compound 9. See procedure.

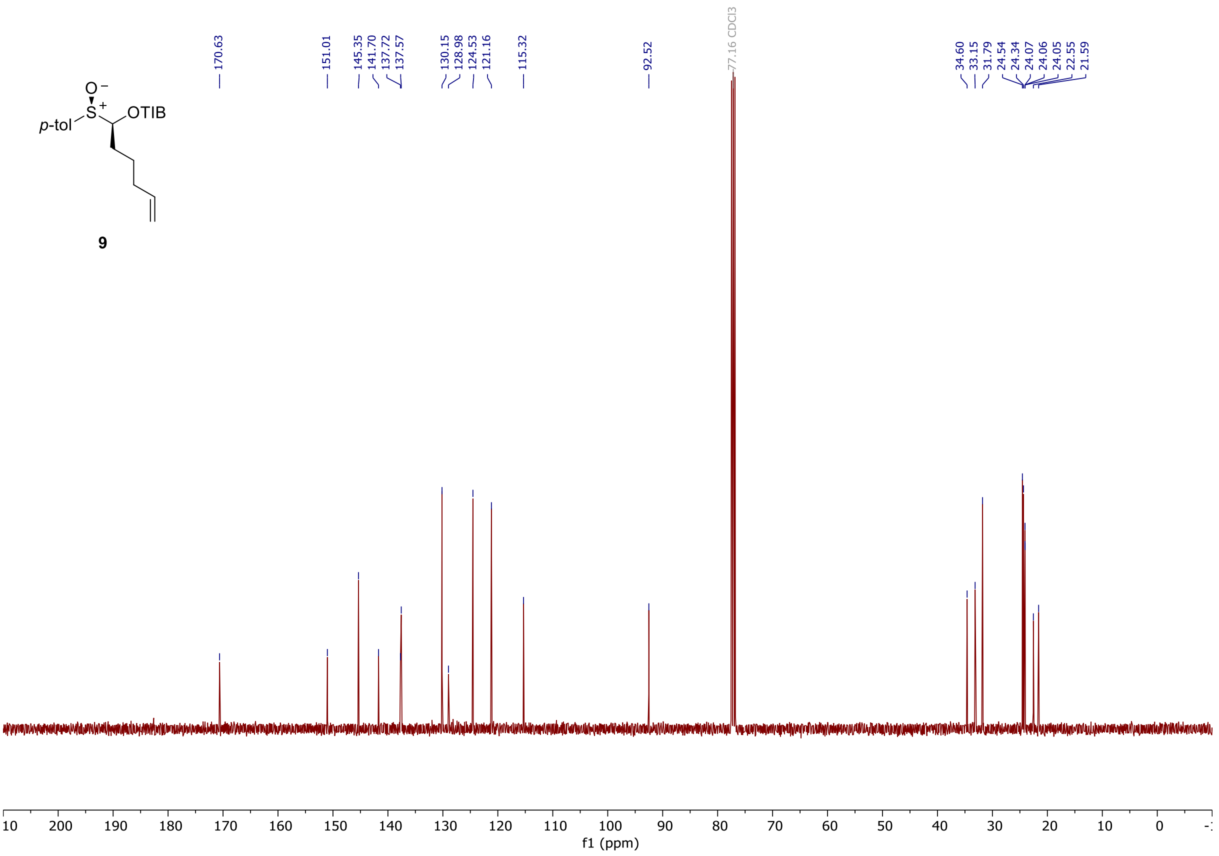

^{1}H NMR (400 MHz, CD_2Cl_2) of compound 27. See procedure.

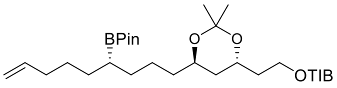

27

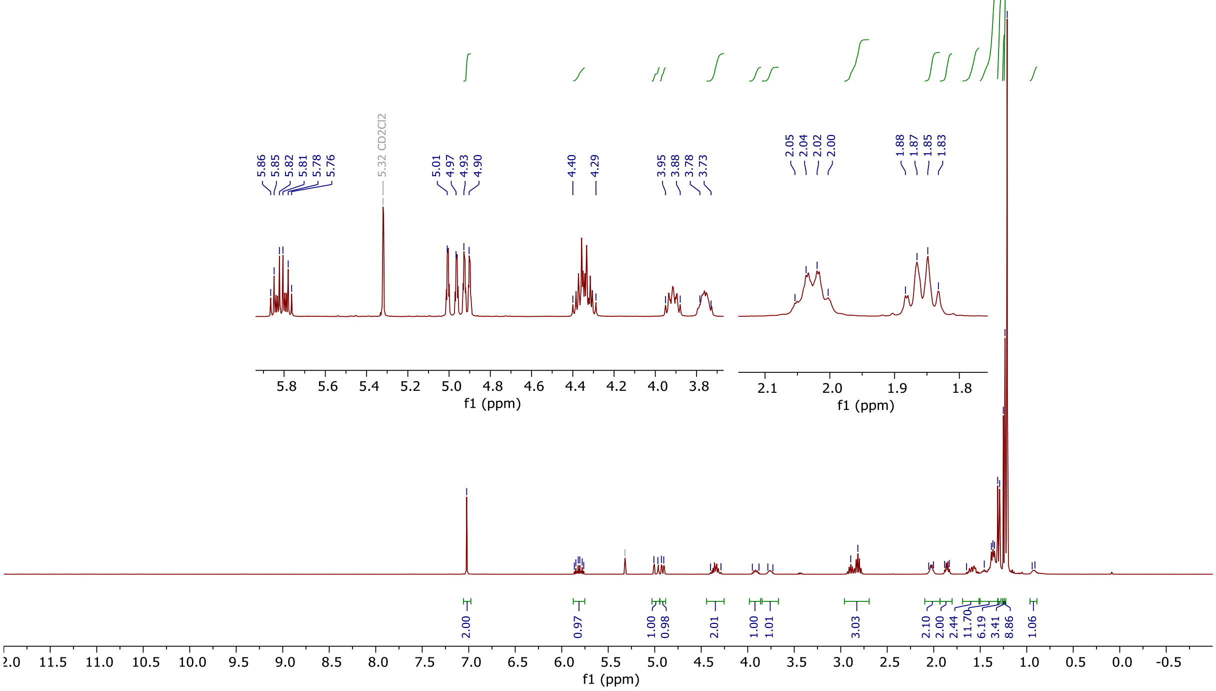

^{13}C NMR (126 MHz, CD_2Cl_2) of compound 27. See procedure.

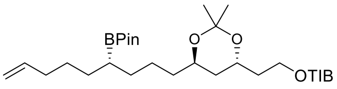

27

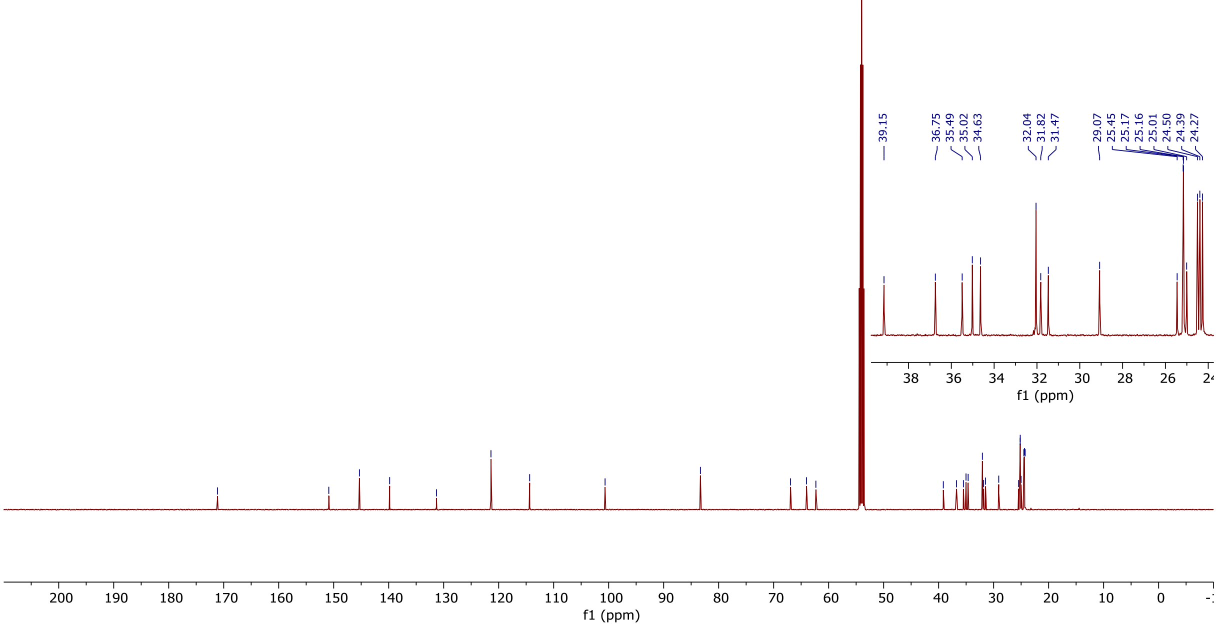

^{1}H NMR (400 MHz, CD_2Cl_2) of compound 28. *See procedure*.

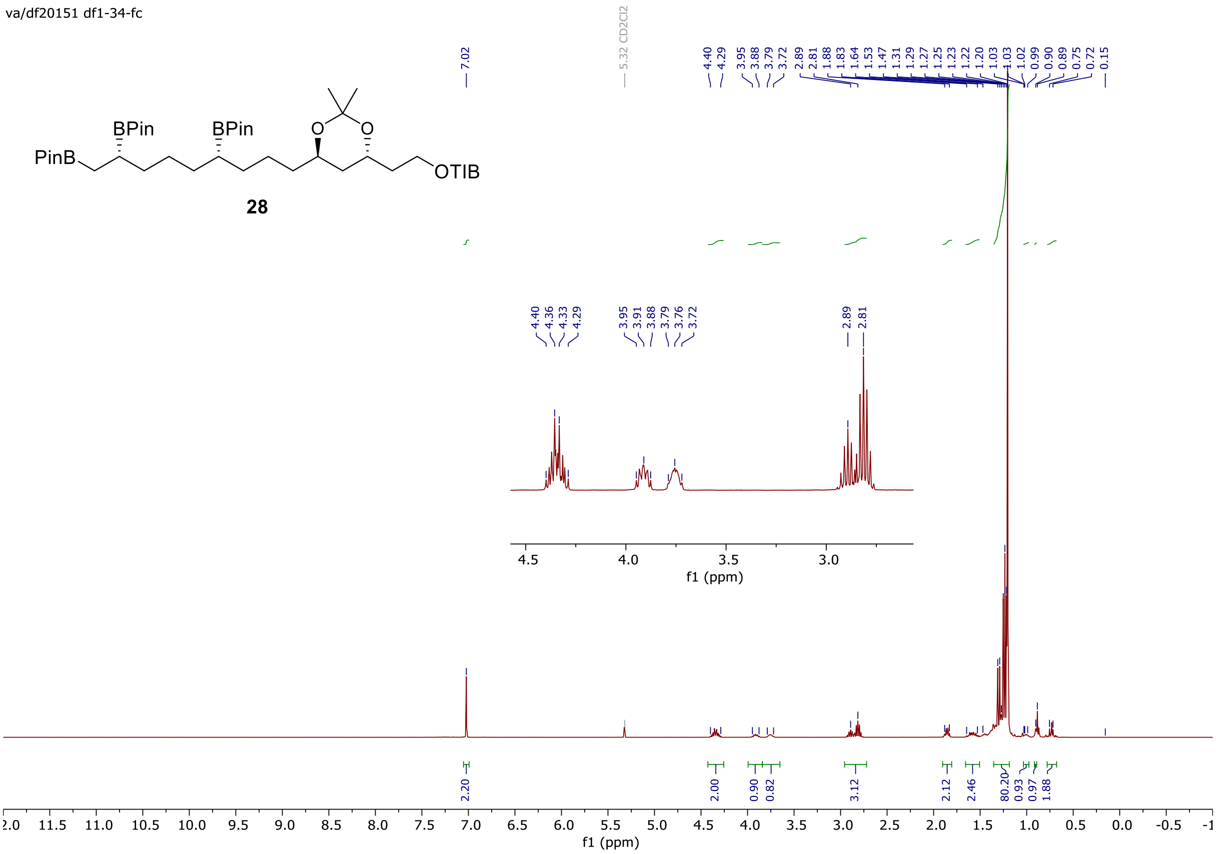

^{13}C NMR (126 MHz, CD_2Cl_2) of compound 28. *See procedure*.

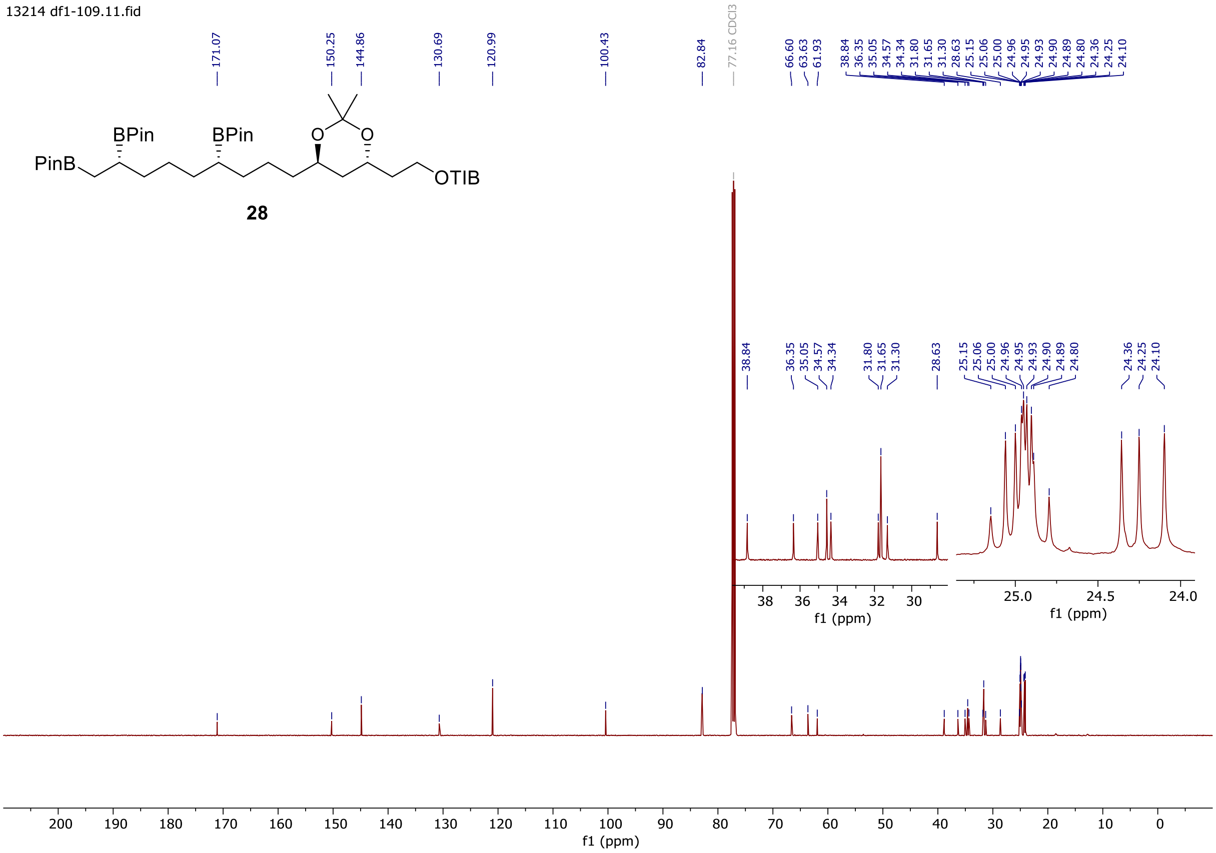

^{1}H NMR (500 MHz, $CDCl_3$) of compound 7. <u>See procedure</u>**.**

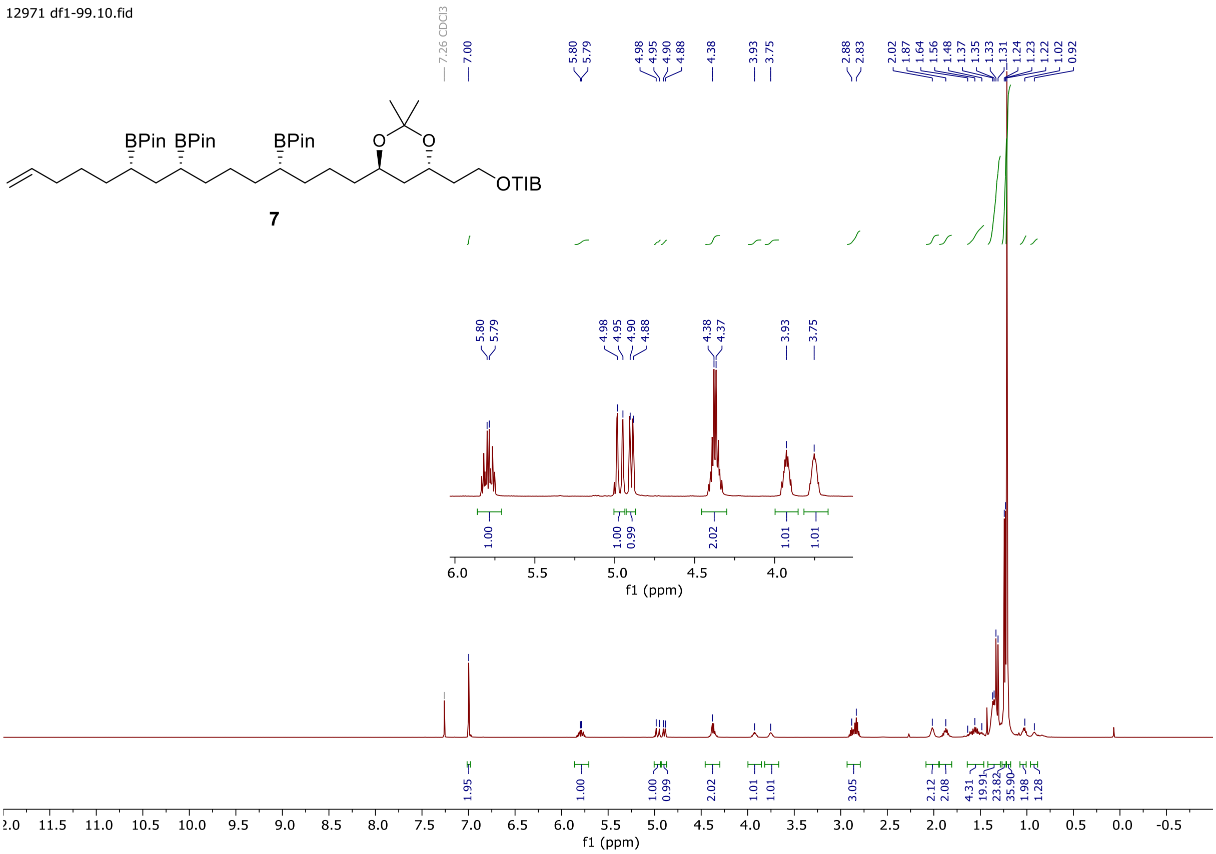

^{13}C NMR (126 MHz, $CDCl_3$) of compound 7. <u>See procedure.</u>

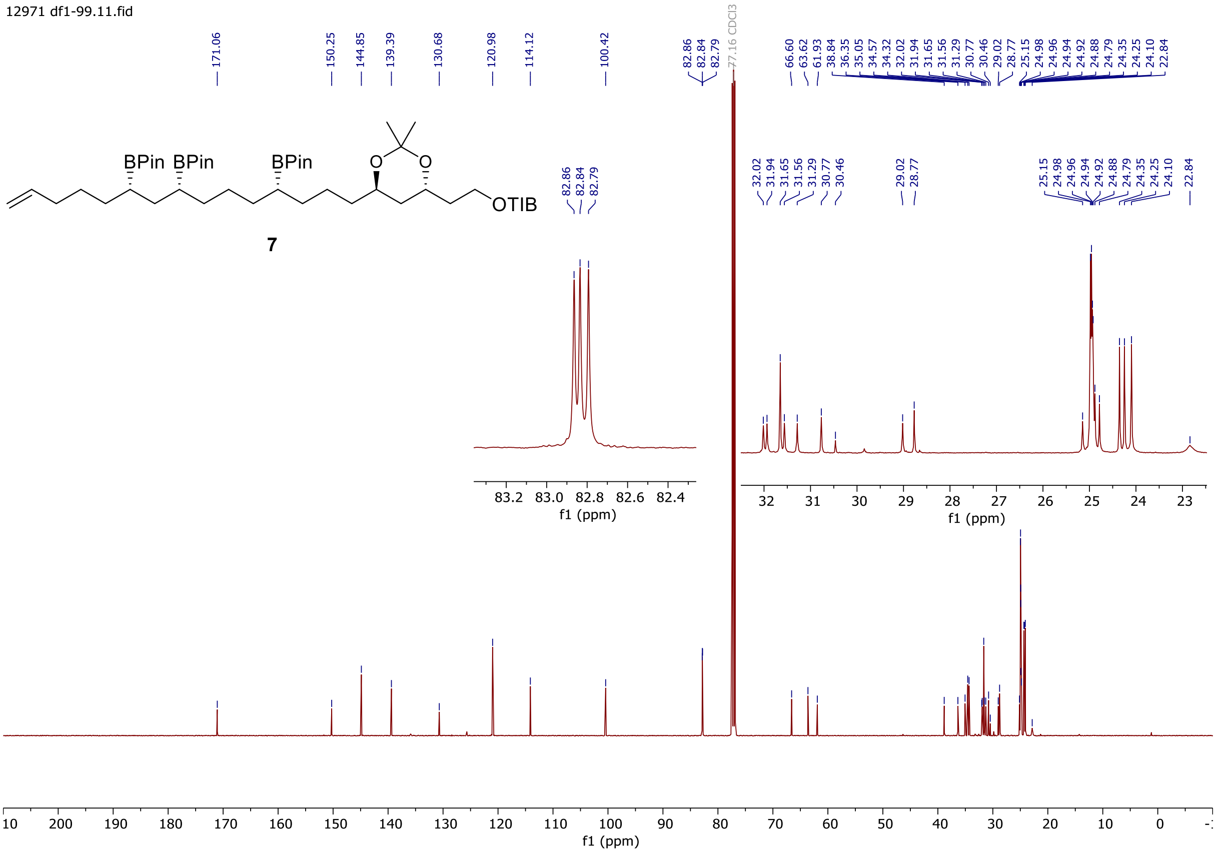

^{1}H NMR (500 MHz, $CDCl_3$) of compound 29. See procedure.

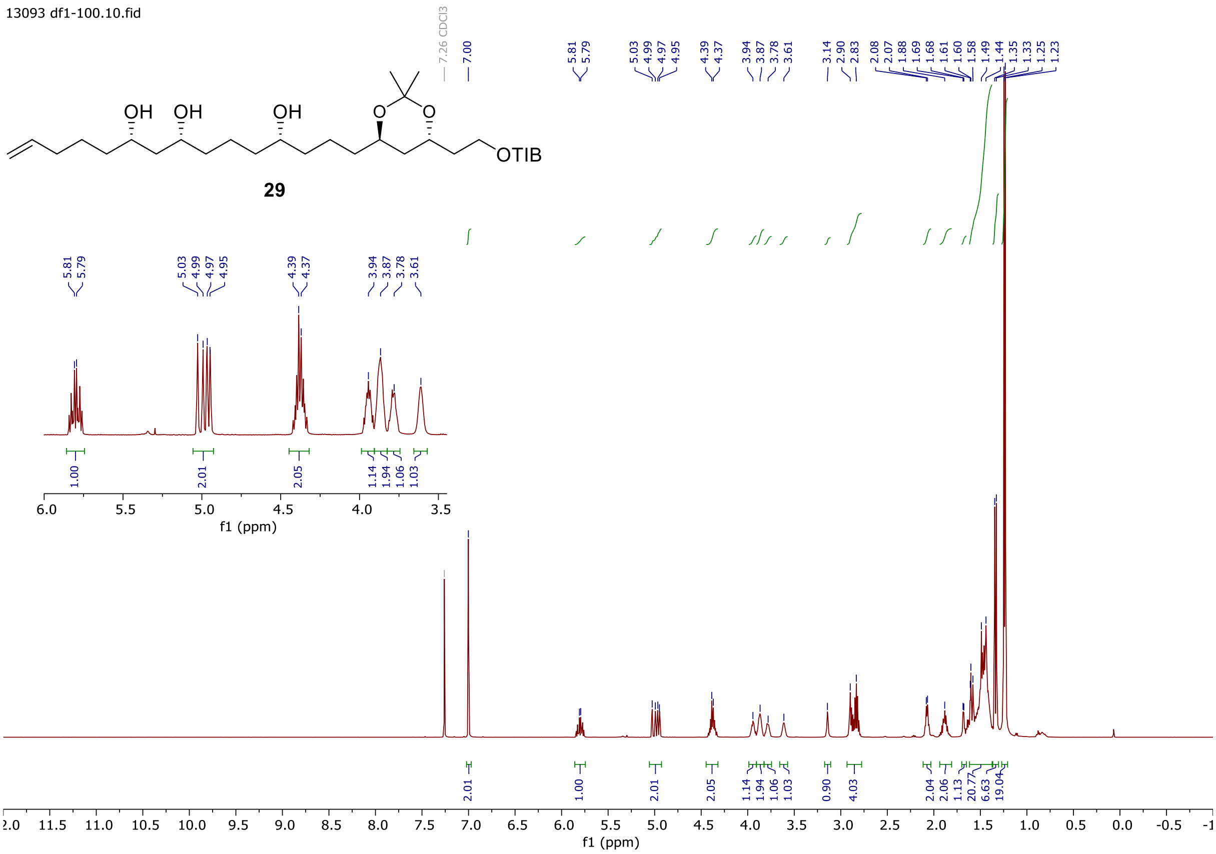

^{13}C NMR (126 MHz, $CDCl_3$) of compound 29 (*full spectrum*). See procedure.

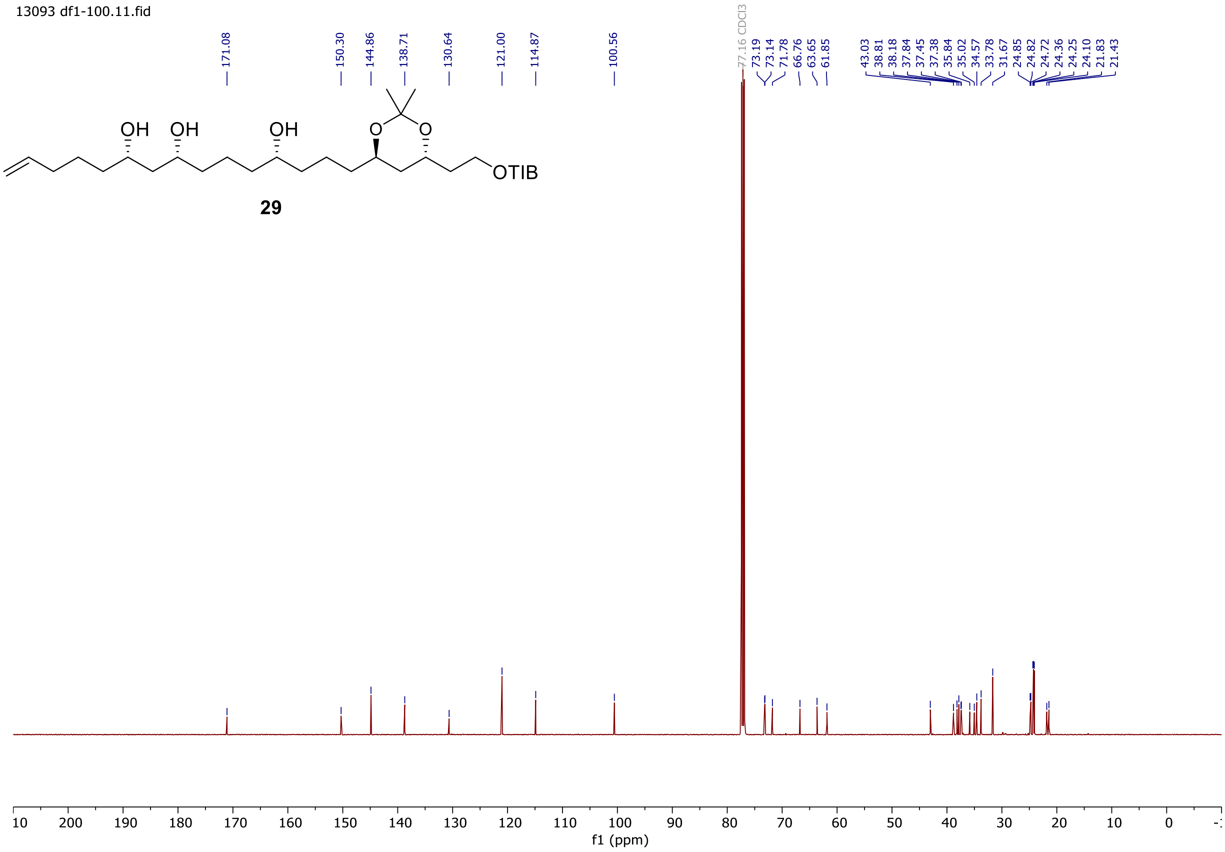

^{13}C NMR (126 MHz, CDCl$_3$) of compound 29 (*partial spectrum*).

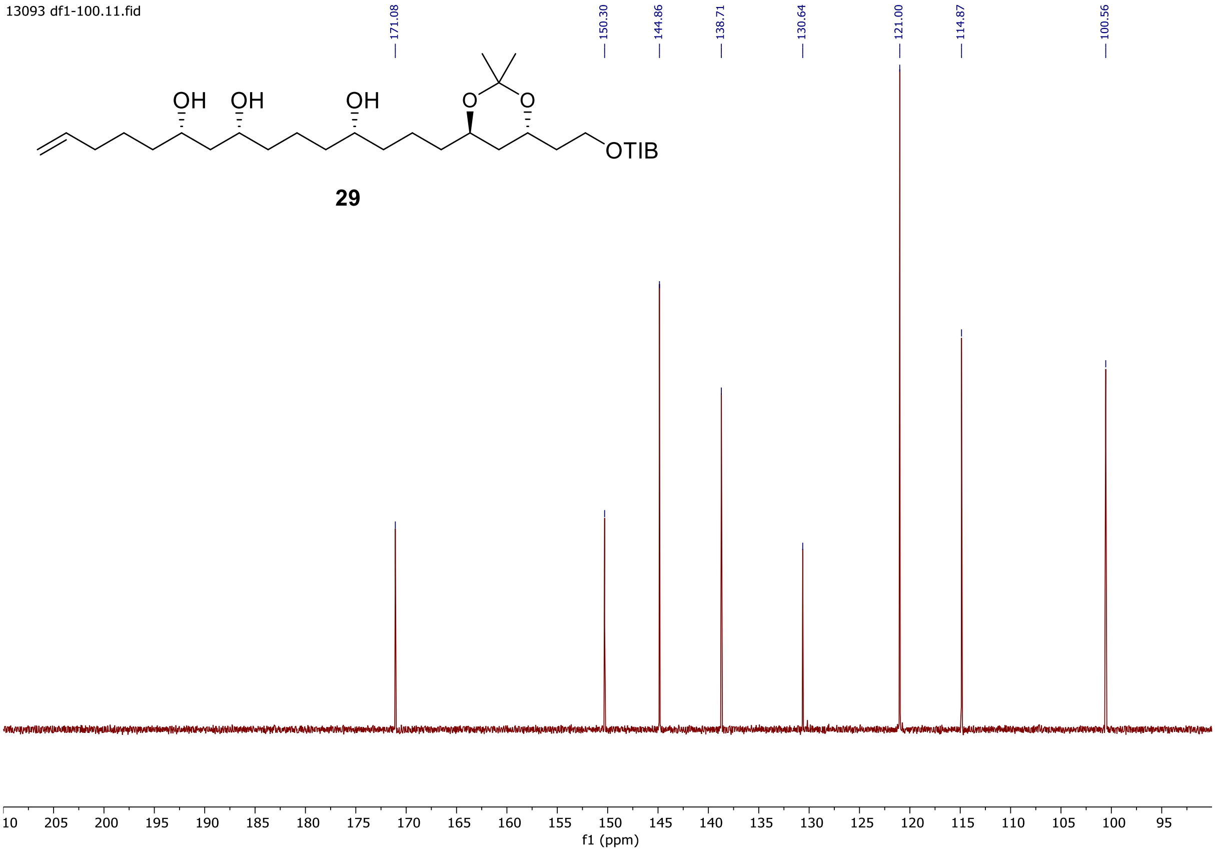

^{13}C NMR (126 MHz, CDCl$_3$) of compound 29 (*partial spectrum*).

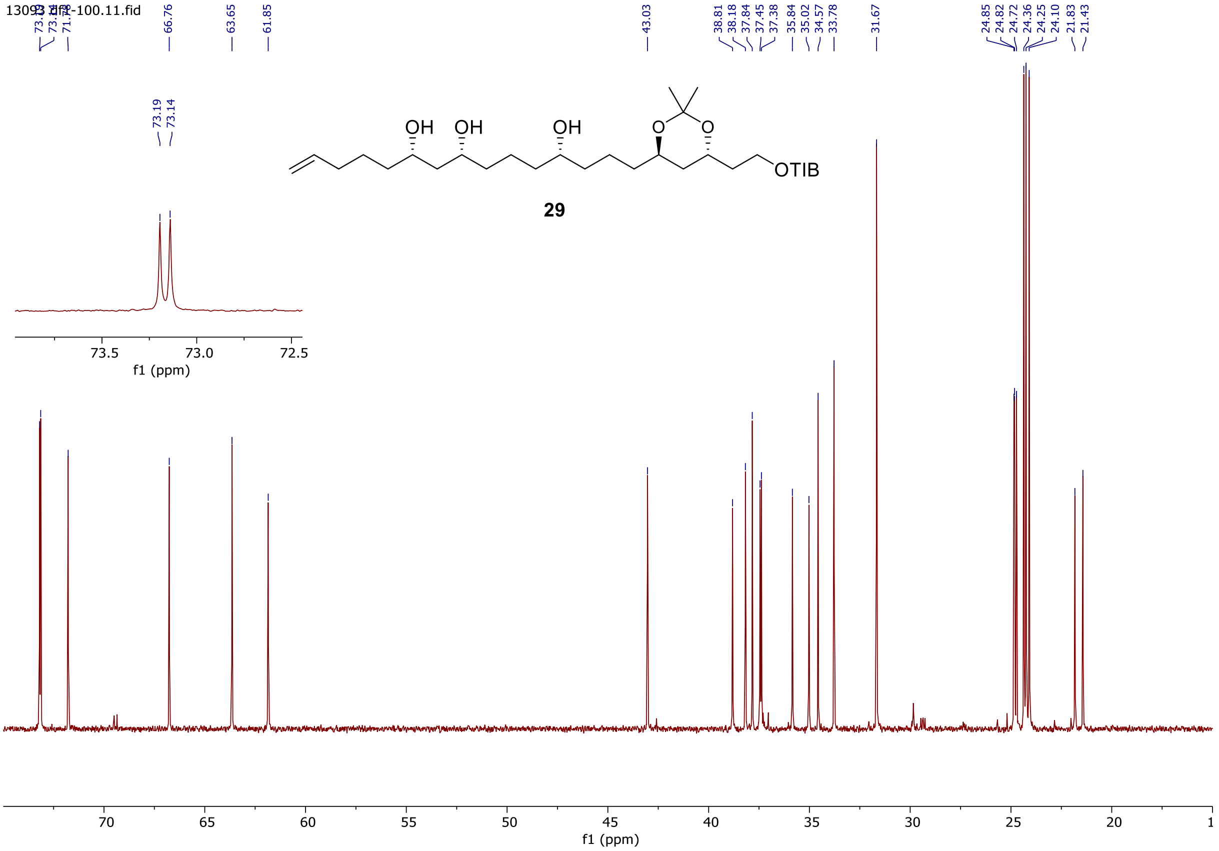

^{1}H NMR (500 MHz, $CDCl_3$) of compound 5 (*full spectrum*). See procedure.

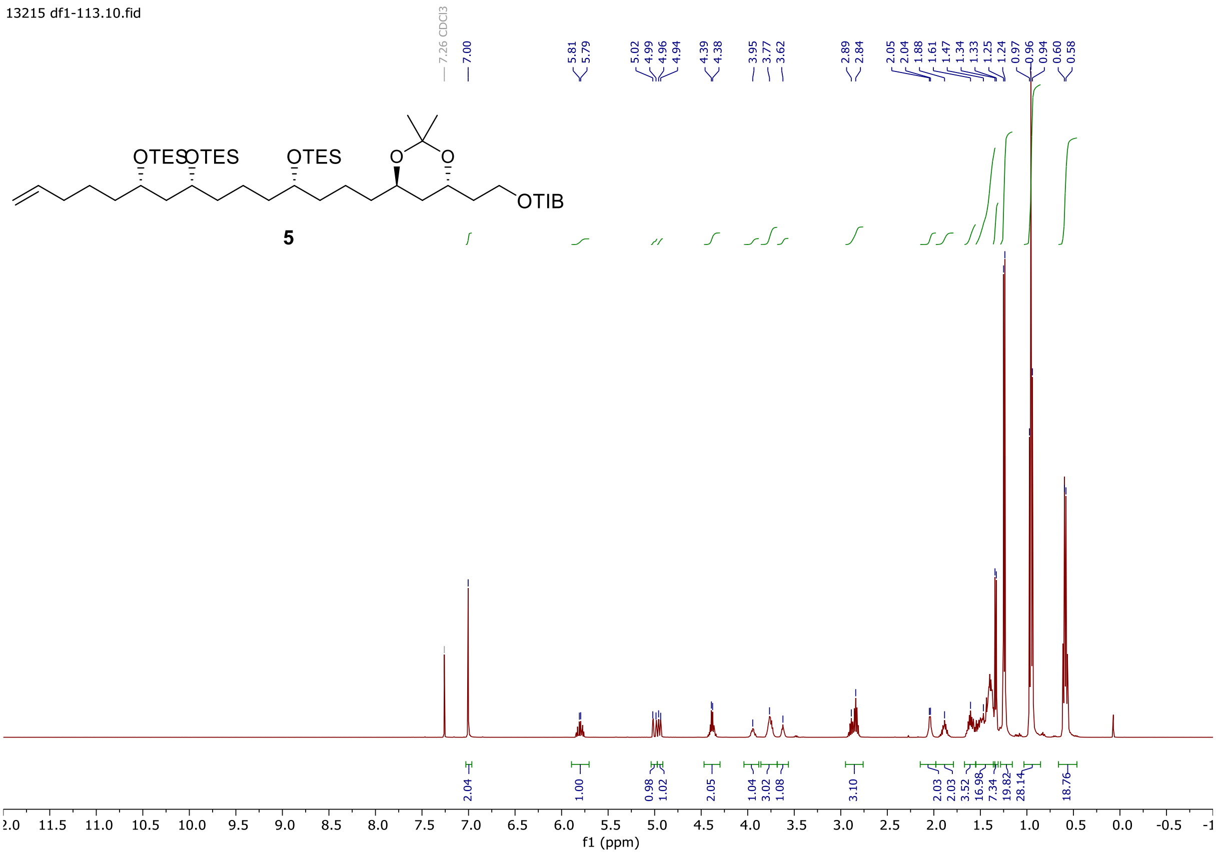

^{1}H NMR (500 MHz, $CDCl_3$) of compound 5 (*partial spectrum*).

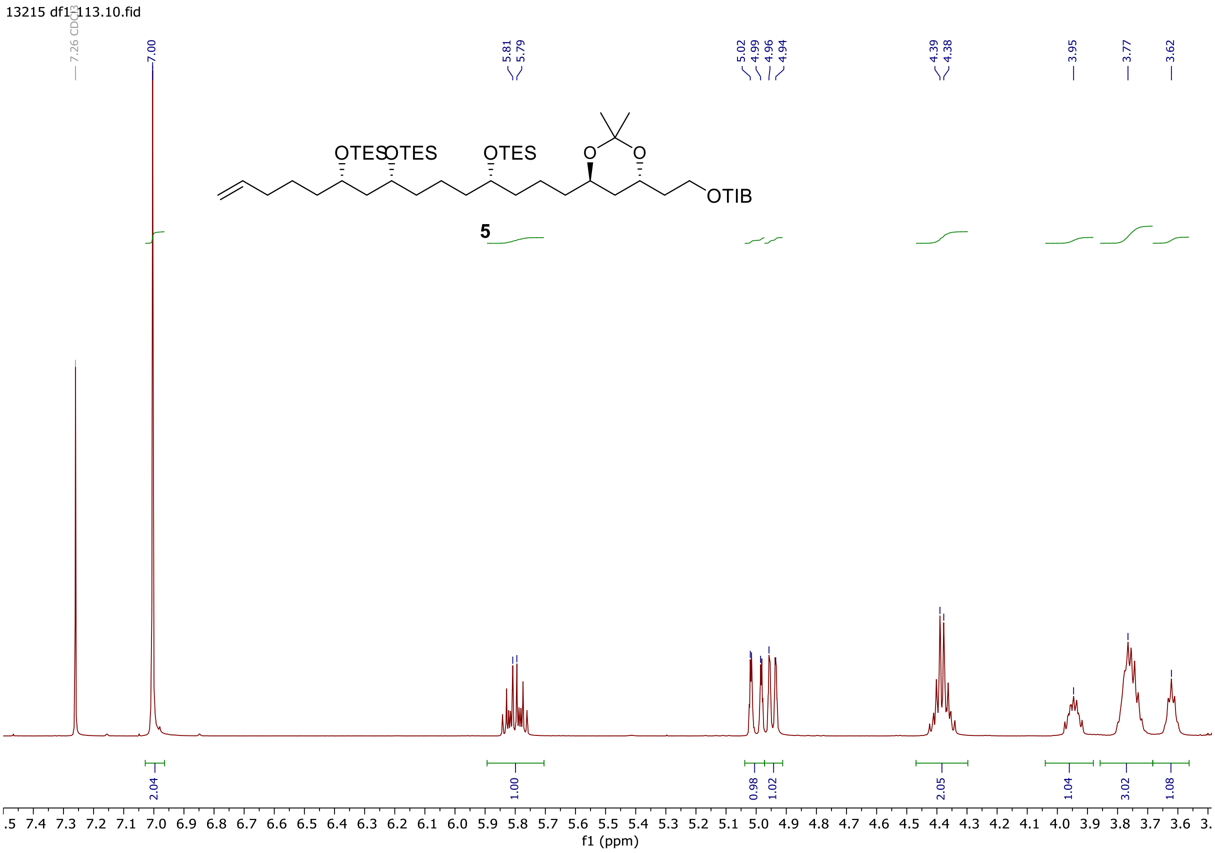

^{1}H NMR (500 MHz, CDCl$_3$) of compound 5 (*partial spectrum*).

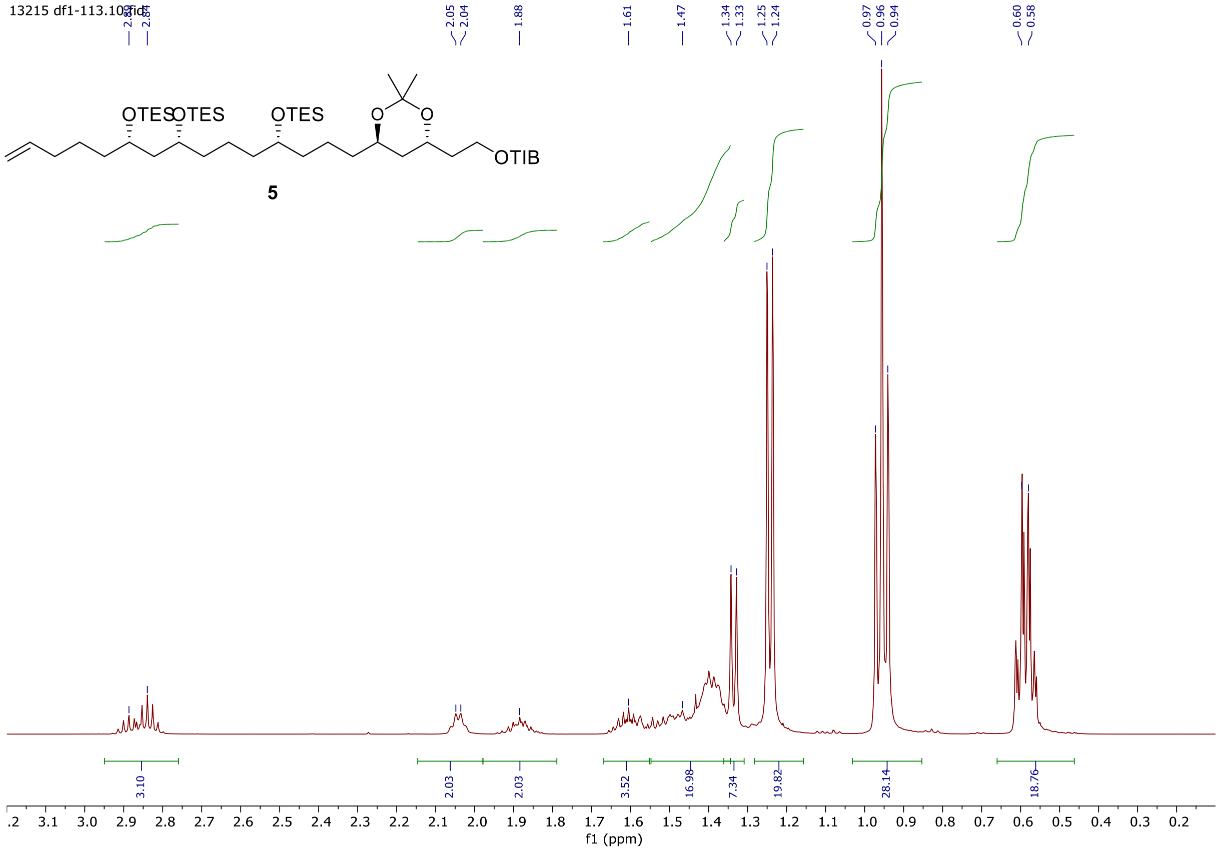

^{13}C NMR (126 MHz, CDCl$_3$) of compound 5 (*full spectrum*). See procedure.

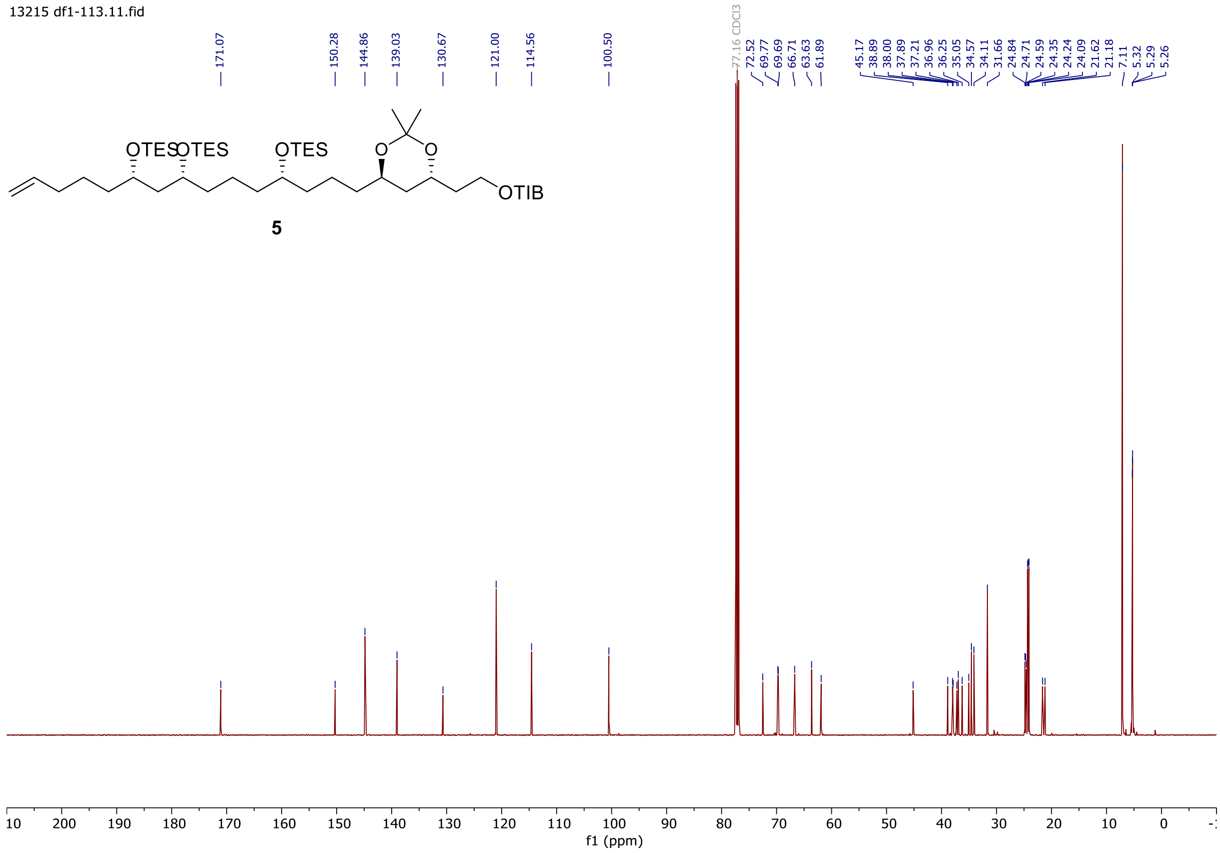

^{13}C NMR (126 MHz, $CDCl_3$) of compound 5 (*partial spectrum*).

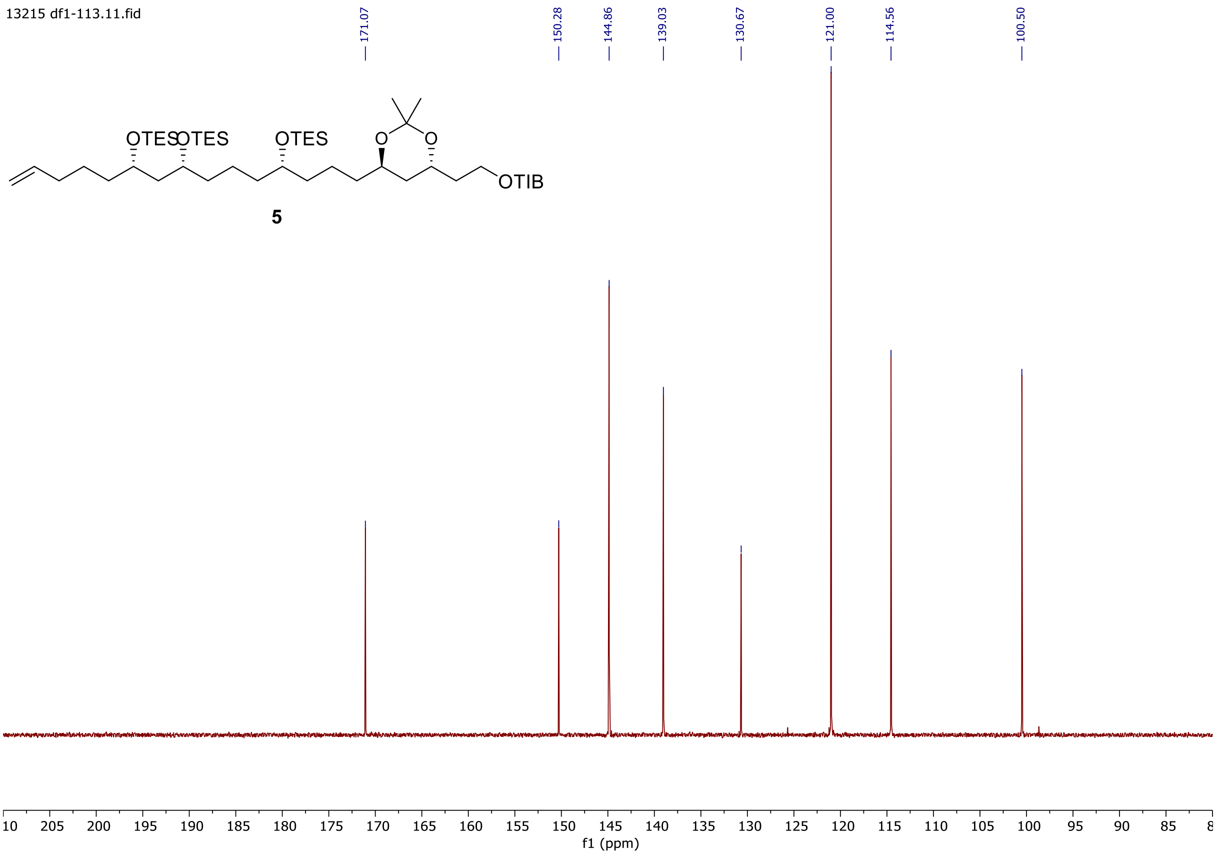

^{13}C NMR (126 MHz, $CDCl_3$) of compound 5 (*partial spectrum*).

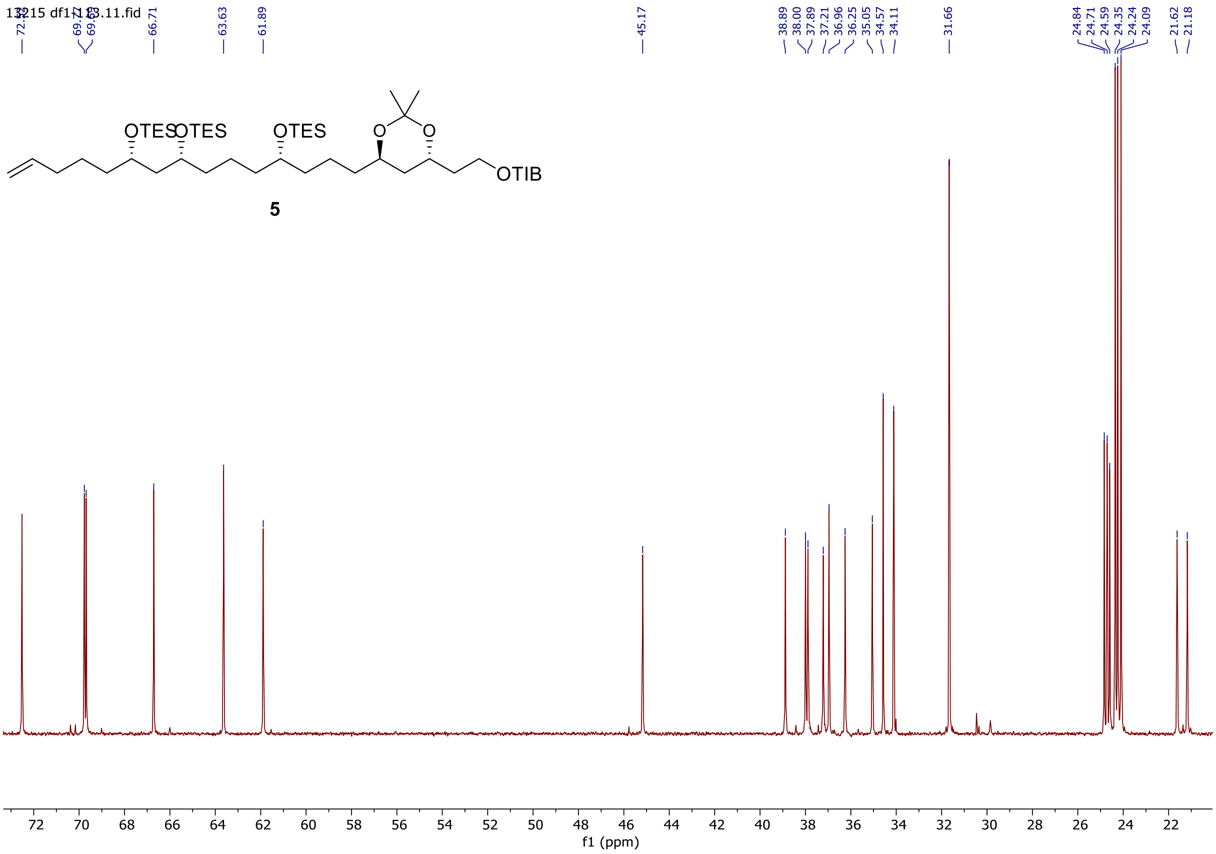

^{1}H NMR (500 MHz, $CDCl_3$) of compound 2 (full spectrum). *See procedure.*

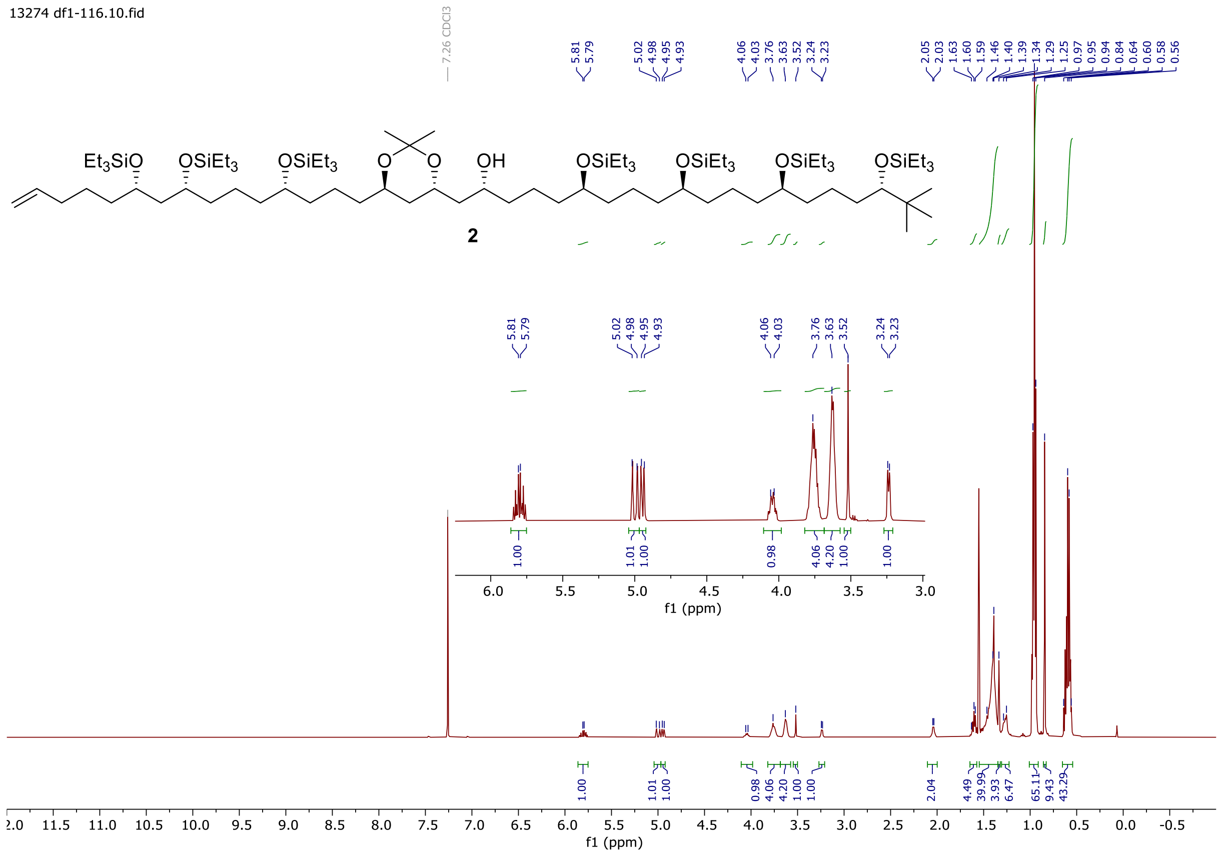

^{1}H NMR (500 MHz, $CDCl_3$) of compound 2 (*partial spectrum*)

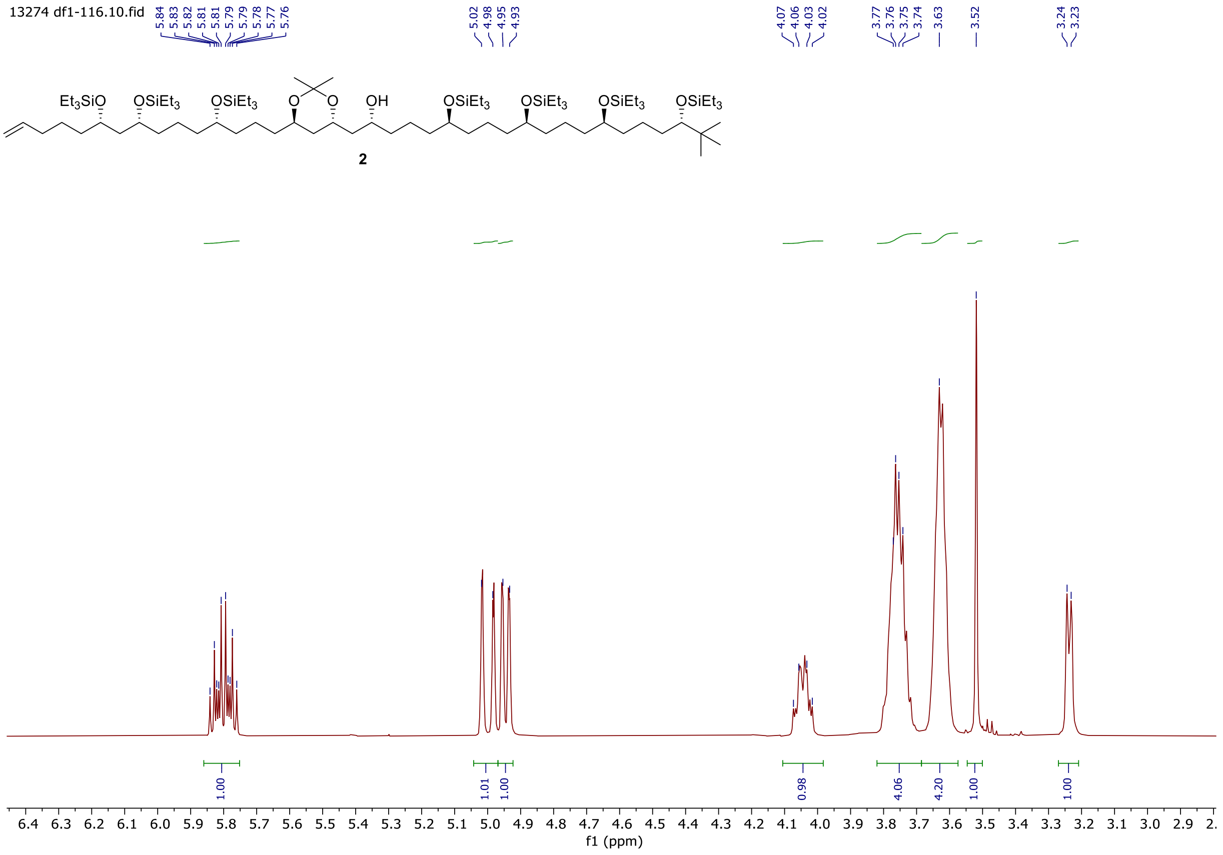

^{1}H NMR (500 MHz, CDCl$_3$) of compound 2 (*partial spectrum*)

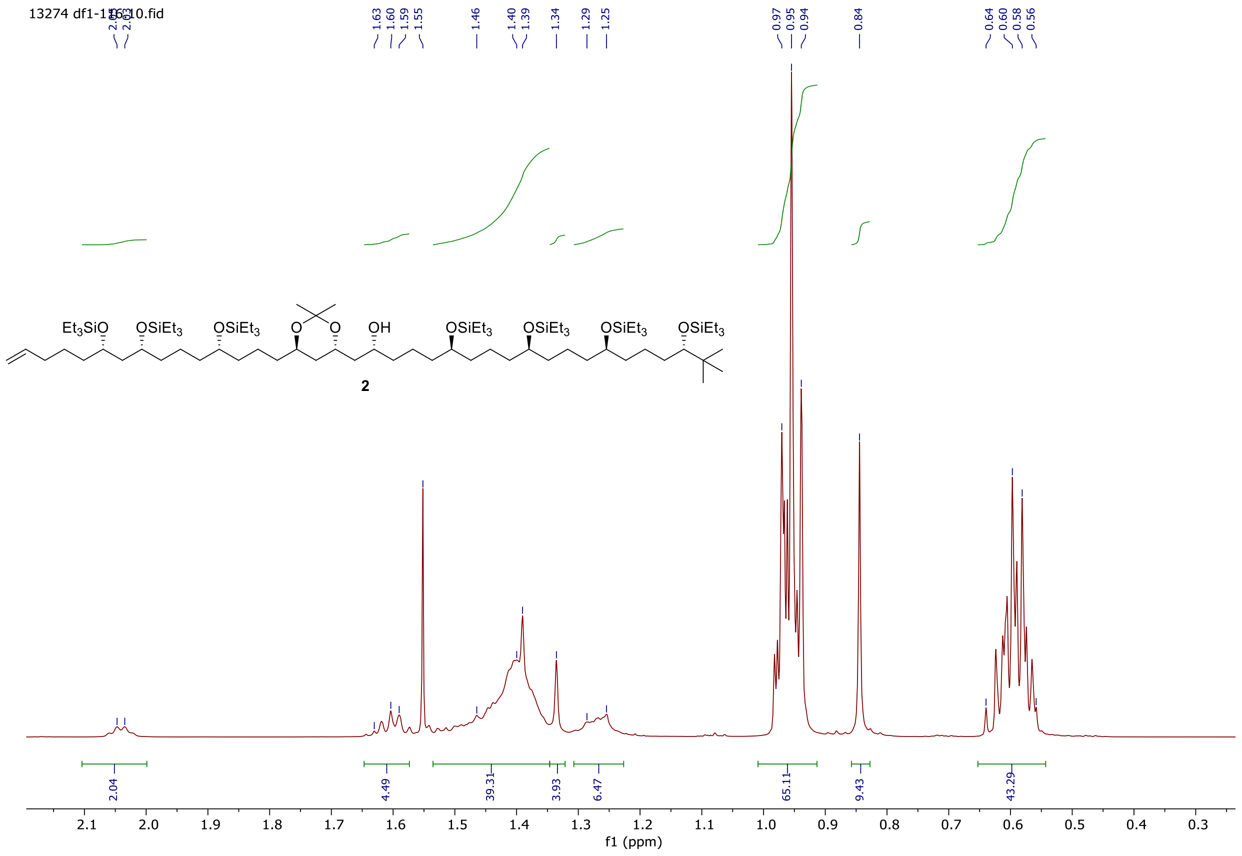

^{13}C NMR (126 MHz, CDCl$_3$) of compound 2 (full spectrum). <u>*See procedure.*</u>

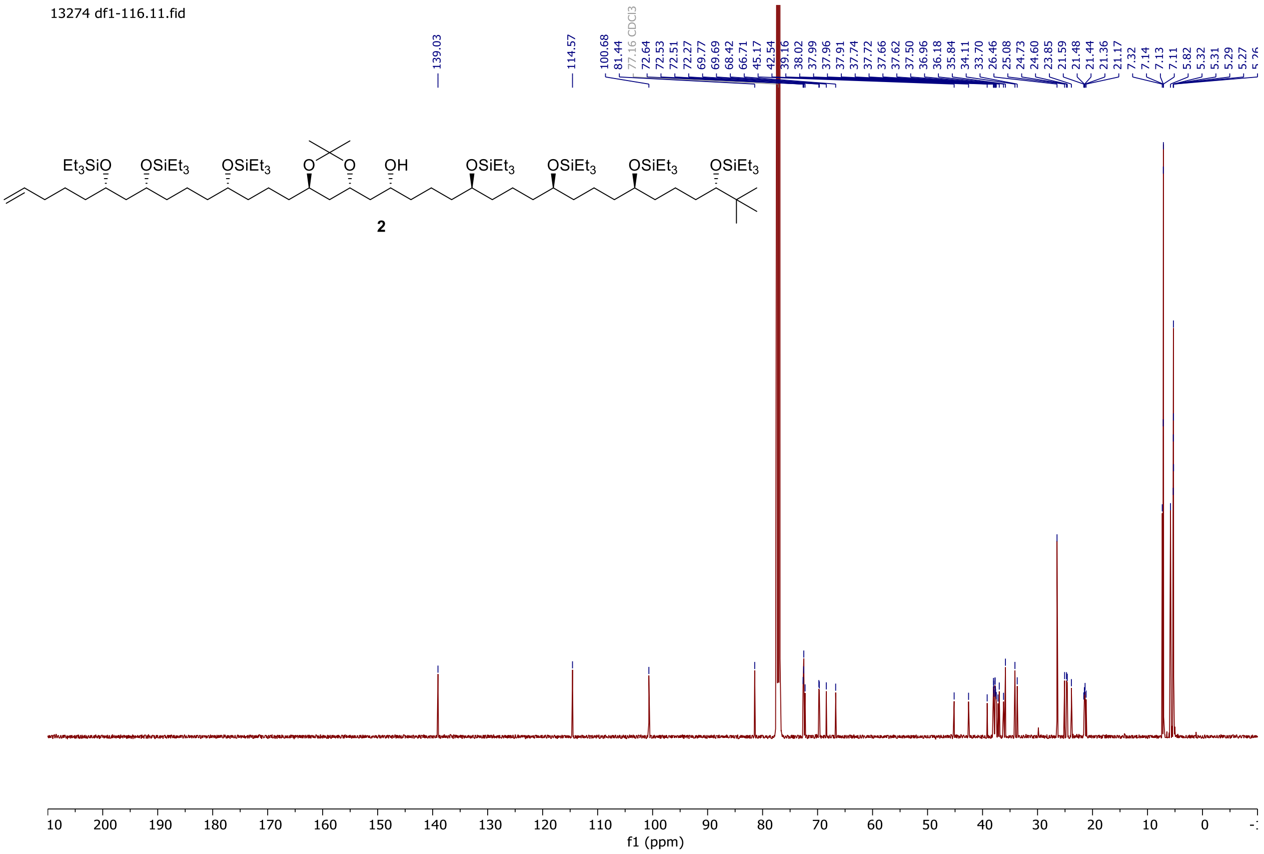

^{13}C NMR (126 MHz, CDCl$_3$) of compound 2 (*partial spectrum*).

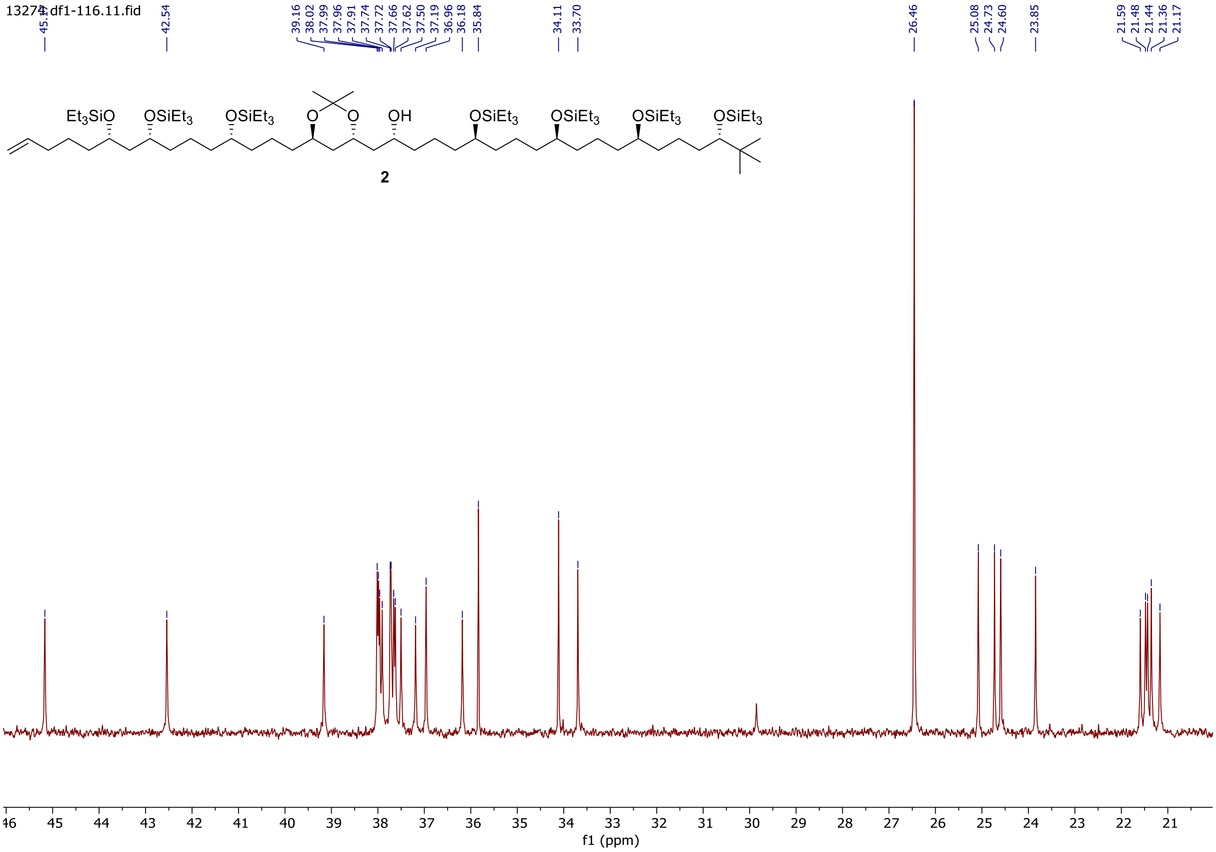

^{13}C NMR (126 MHz, CDCl$_3$) of compound 2 (*partial spectrum*).

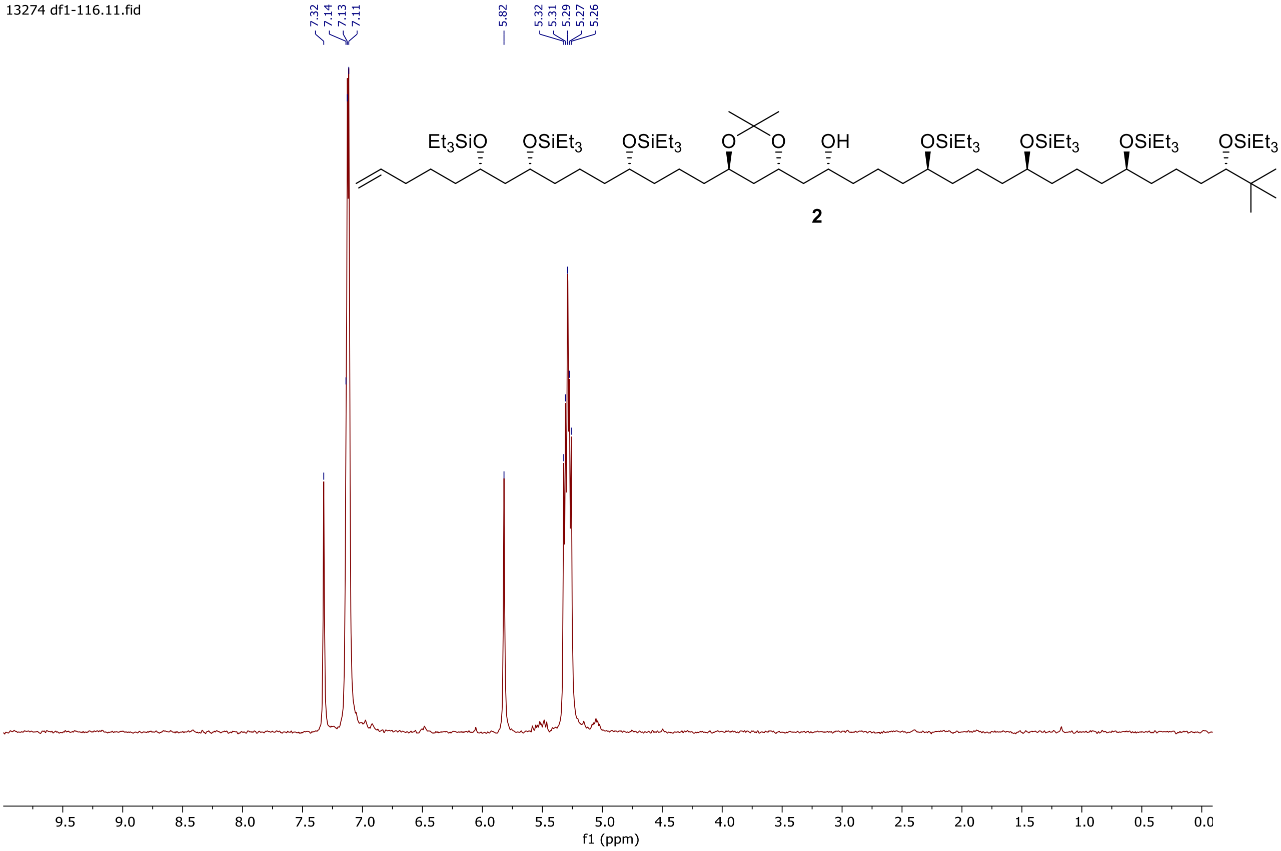

^{1}H NMR (400 MHz, CD_2Cl_2) of compound 33. See procedure.

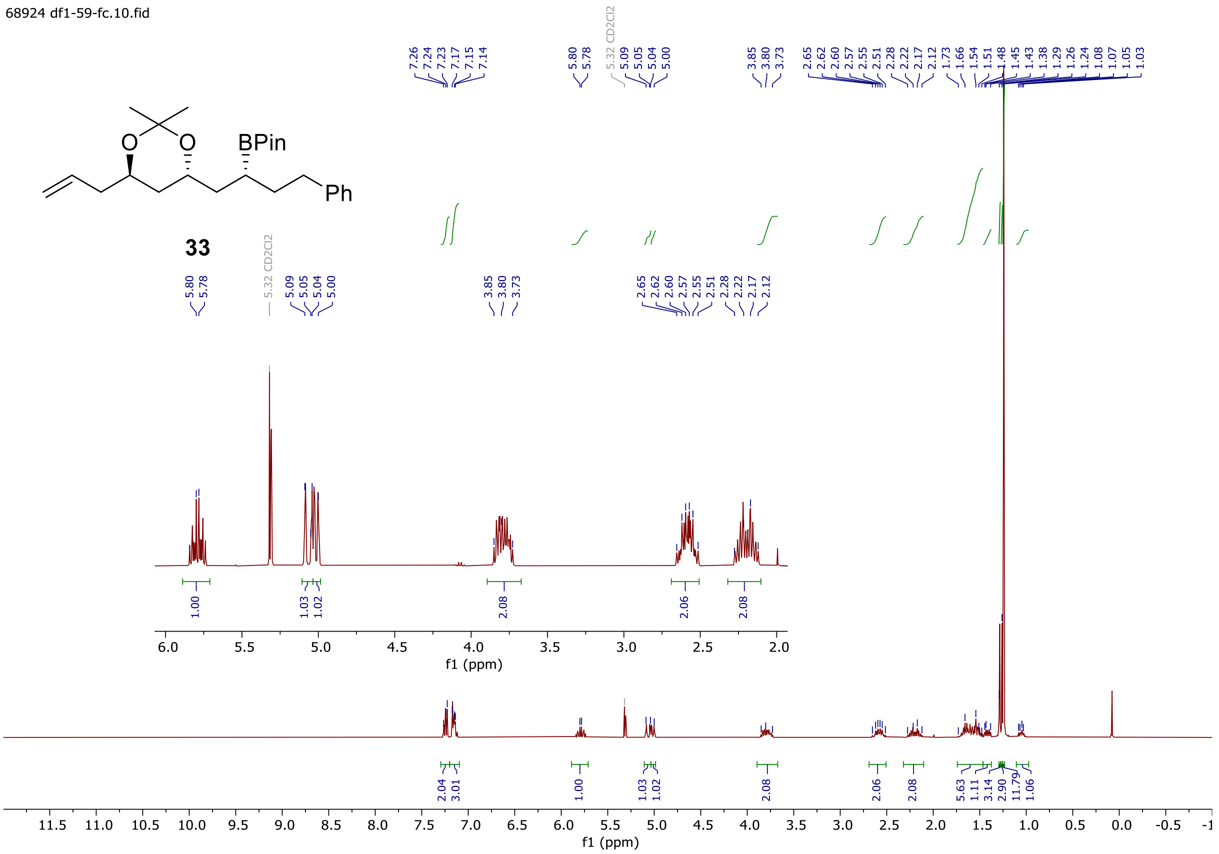

^{13}C NMR (101 MHz, CD_2Cl_2) of compound 33. See procedure.

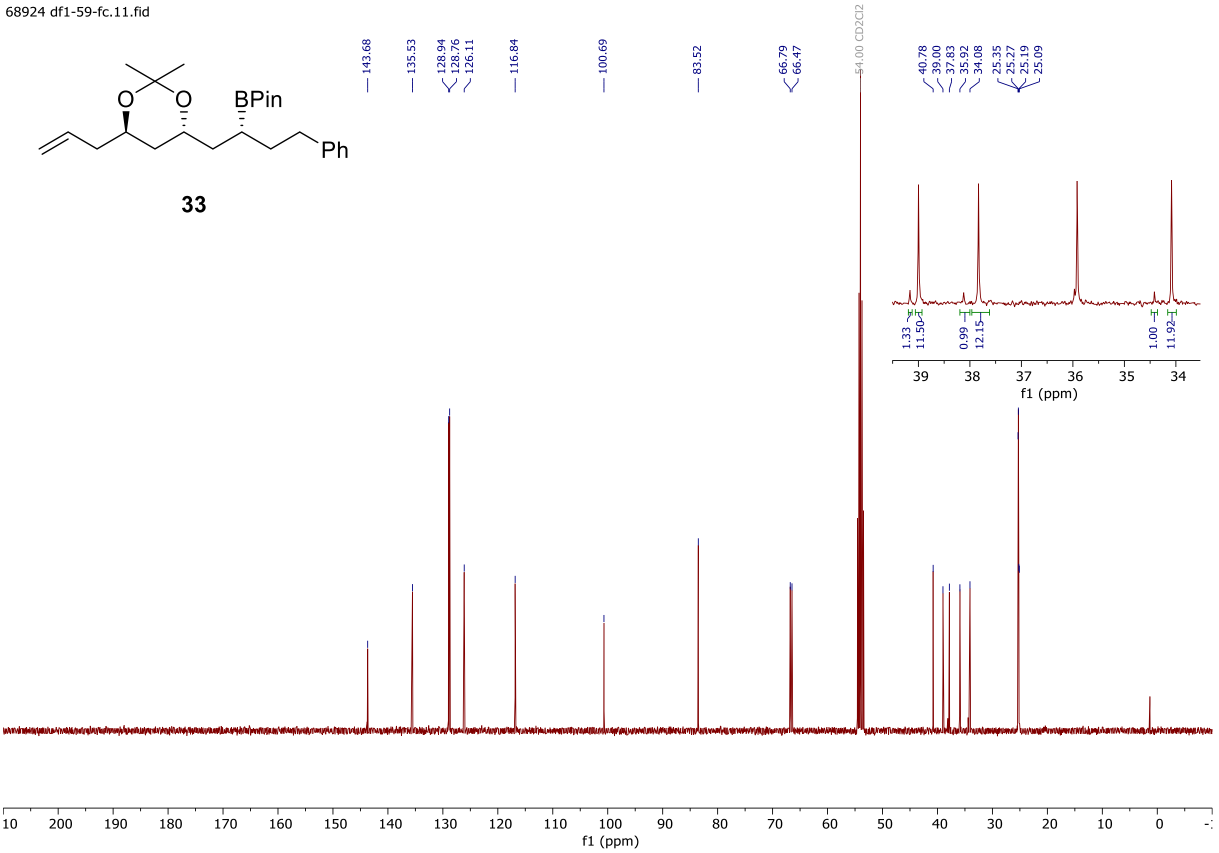

^{1}H NMR (500 MHz, CDCl$_3$) of compound 34 (full spectrum). See procedure.

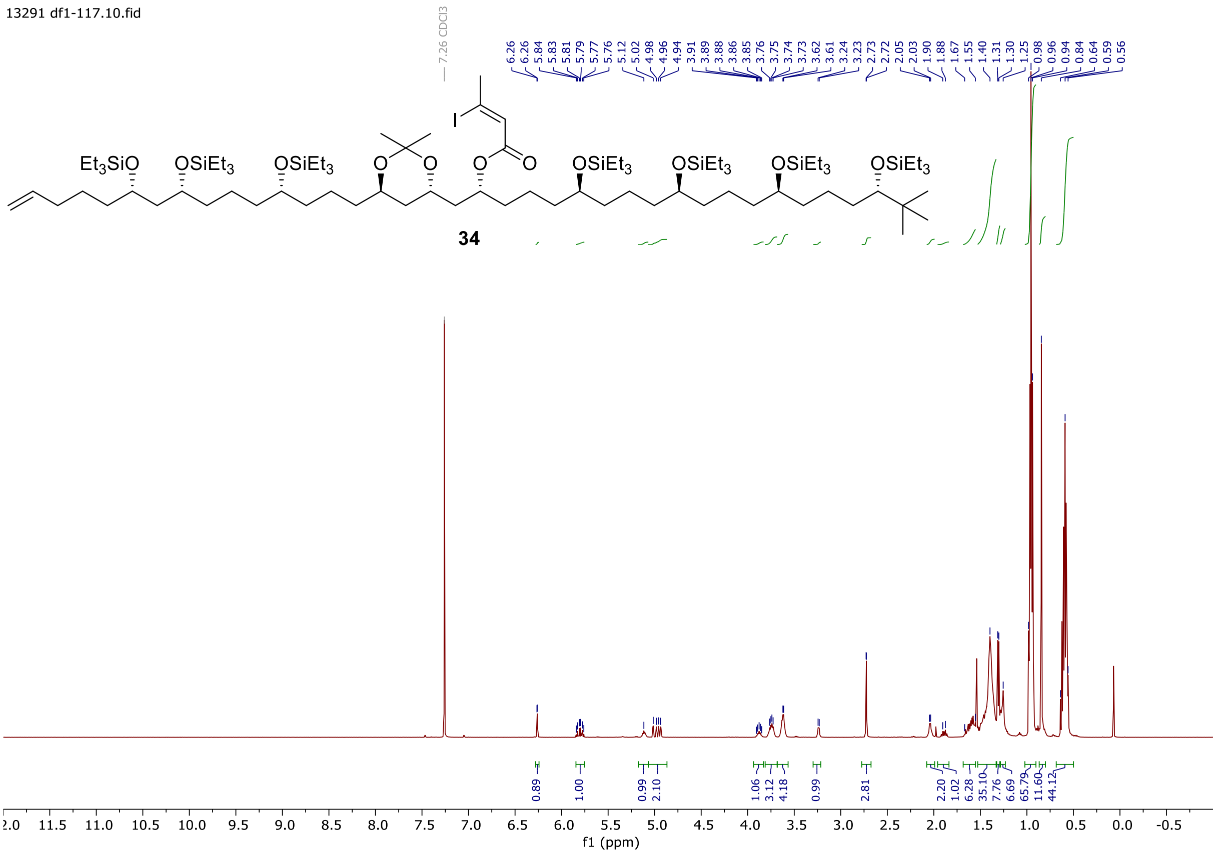

^{1}H NMR (500 MHz, CDCl$_3$) of compound 34 (*partial spectrum*).

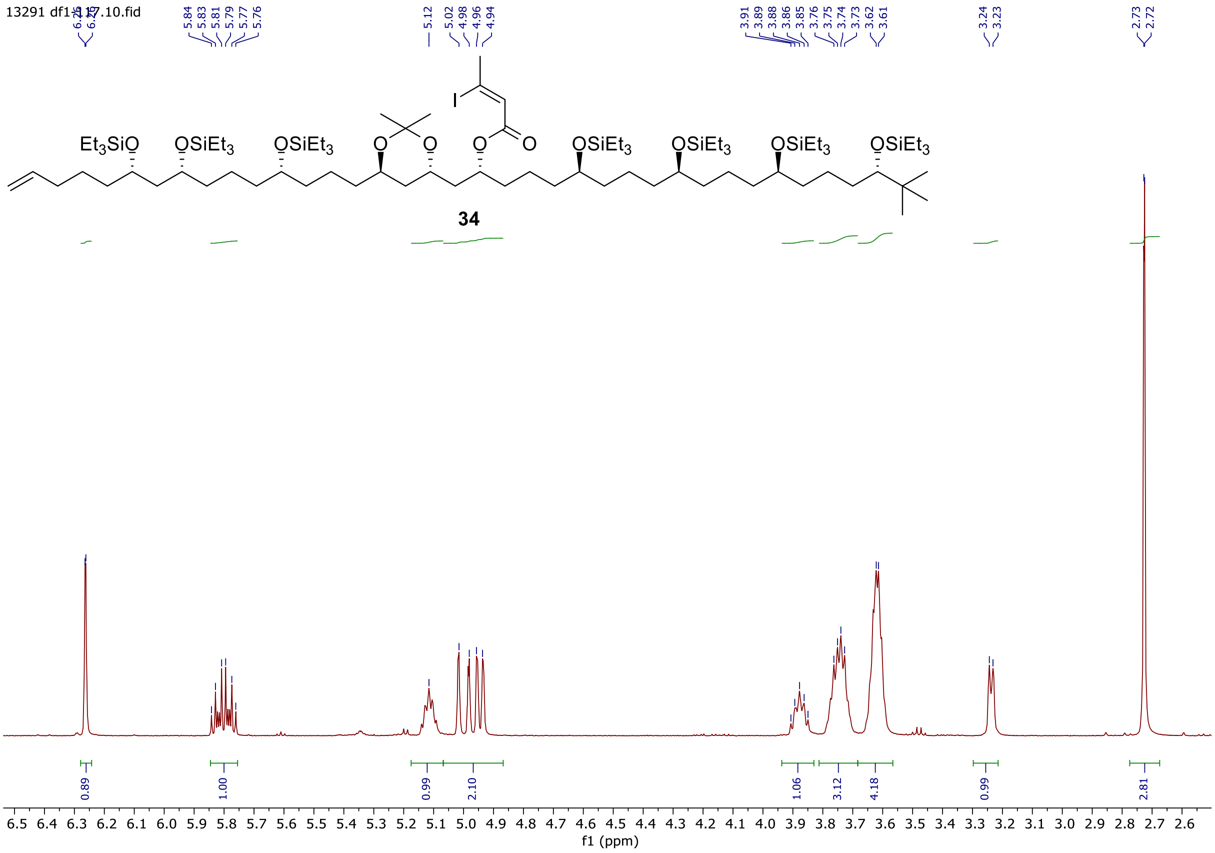

^{1}H NMR (500 MHz, $CDCl_3$) of compound 34 (*partial spectrum*).

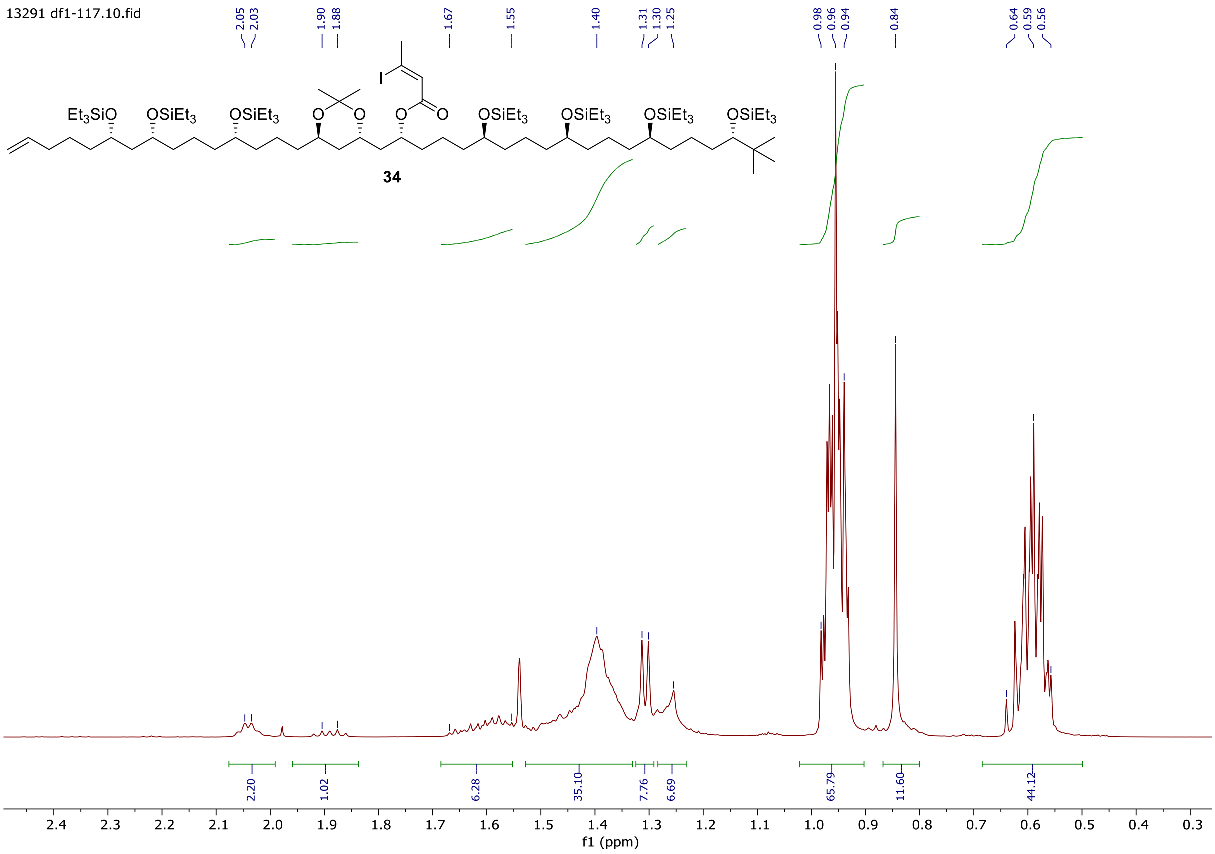

^{13}C NMR (126 MHz, $CDCl_3$) of compound 34 (full spectrum). *See procedure*.

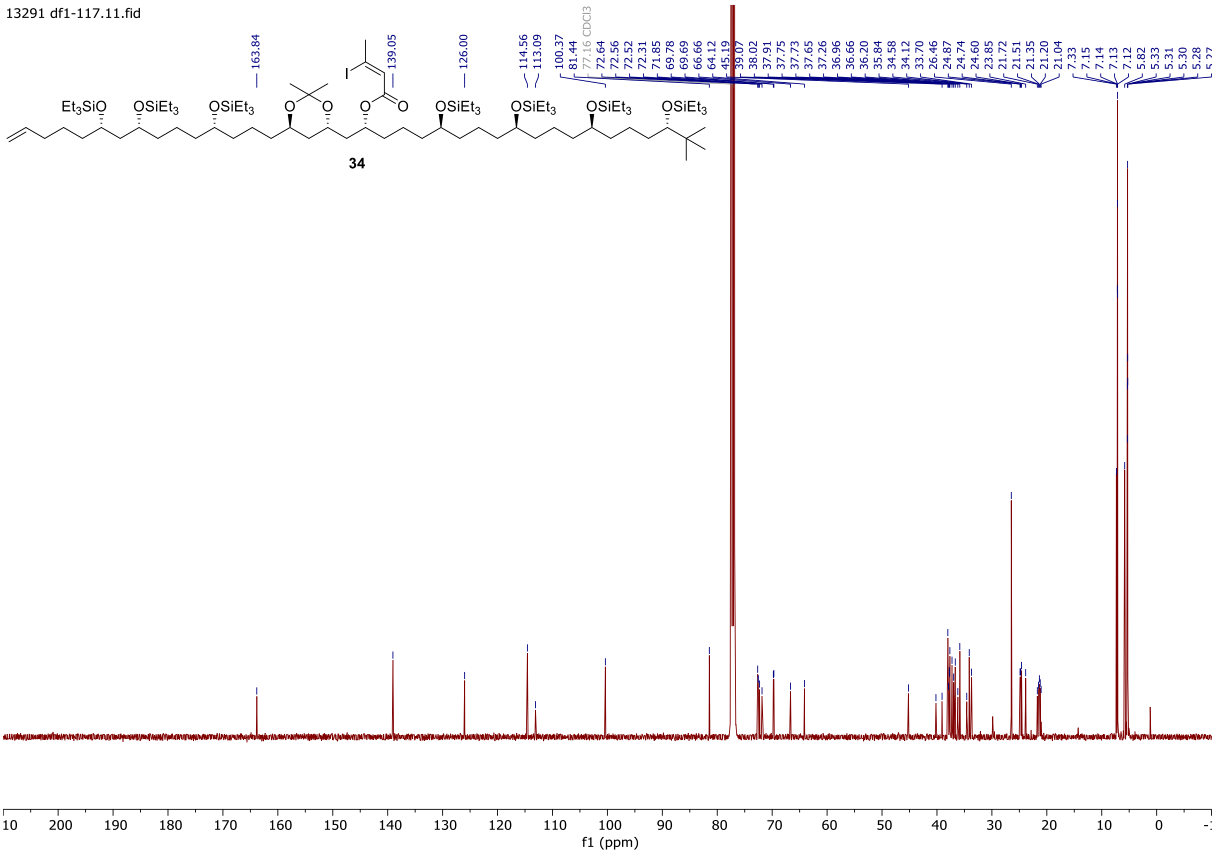

^{13}C NMR (126 MHz, $CDCl_3$) of compound 34 (*partial spectrum*).

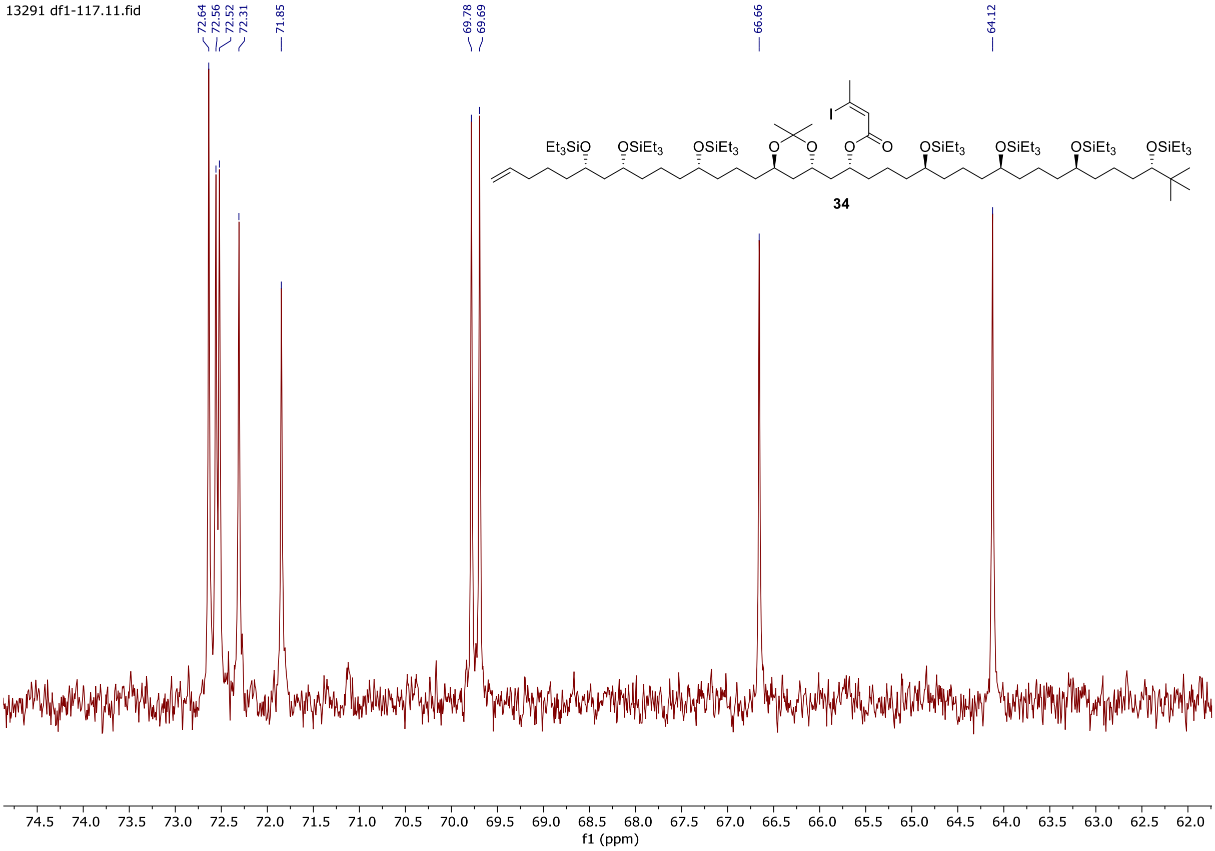

^{13}C NMR (126 MHz, $CDCl_3$) of compound 34 (*partial spectrum*).

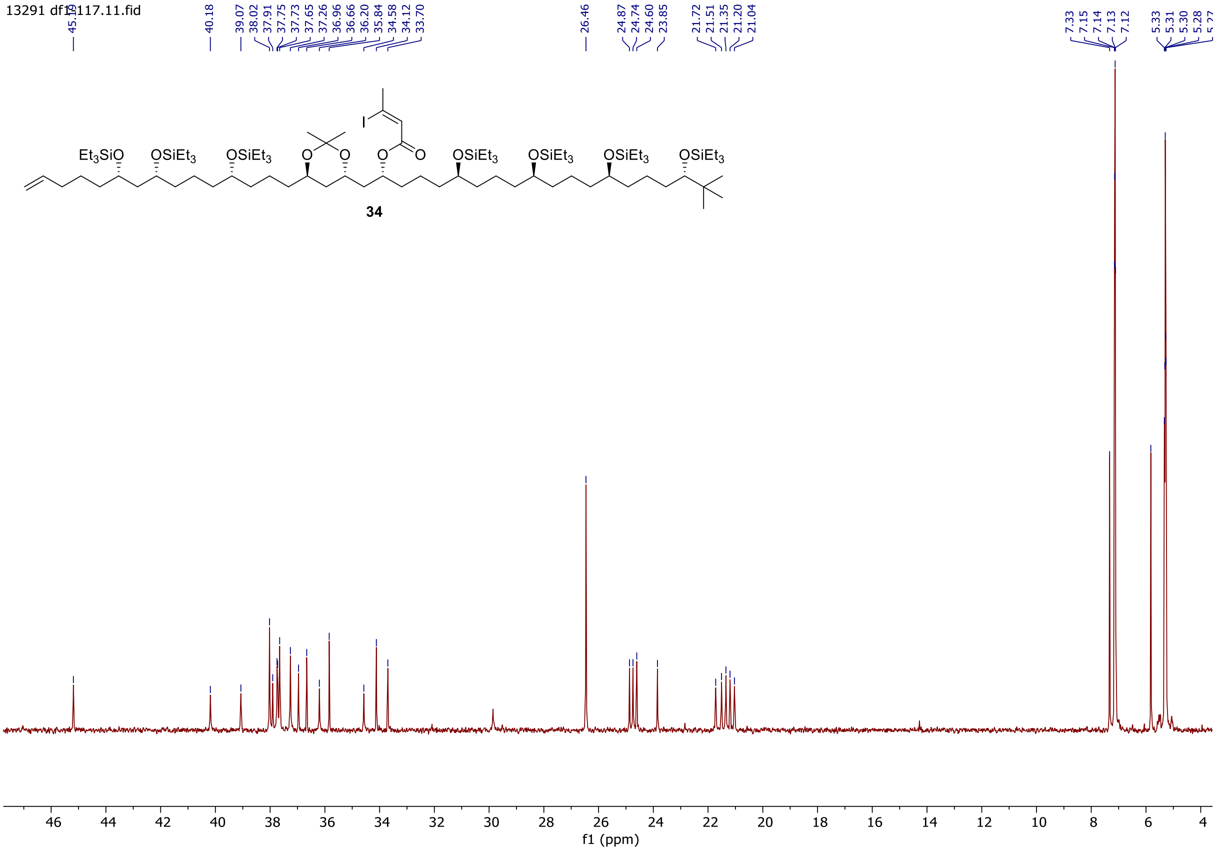

^{1}H NMR (500 MHz, CDCl3) of compound 35 (full spectrum). See procedure.

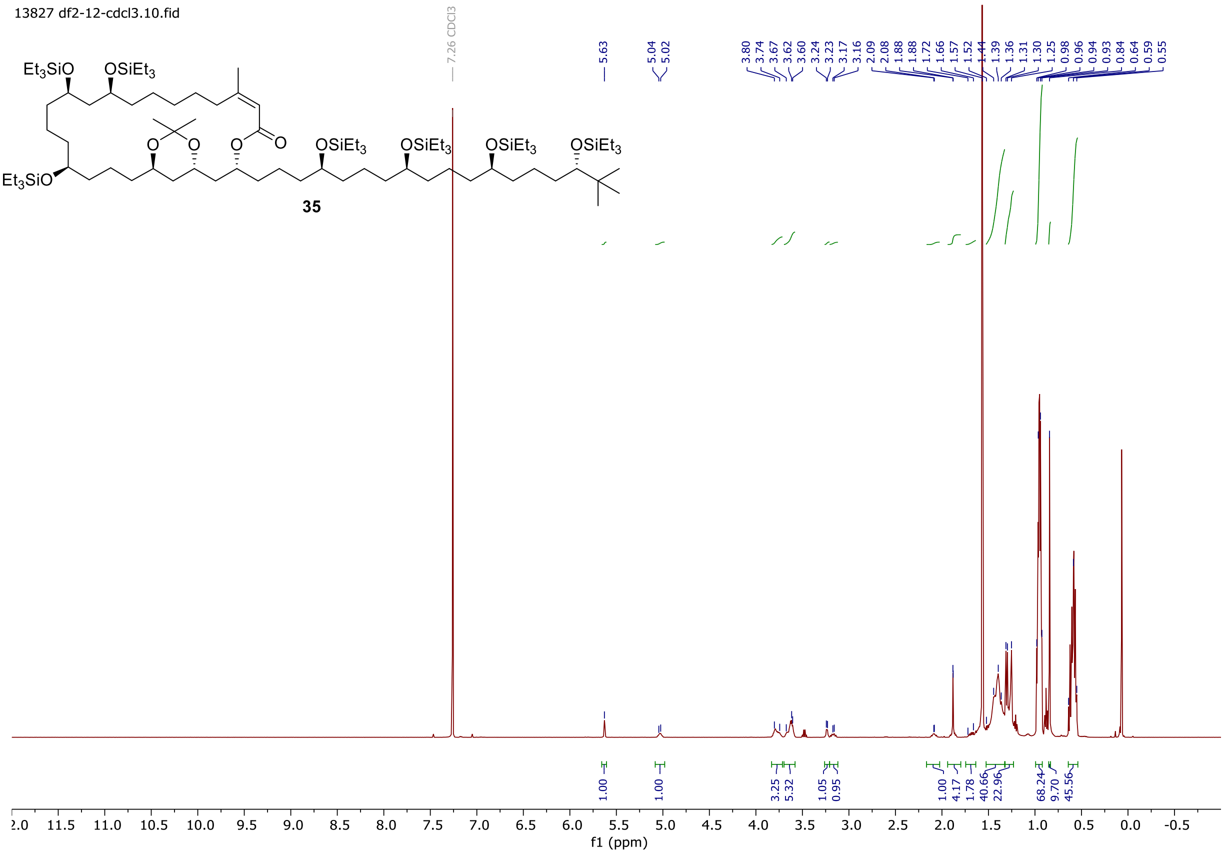

^{1}H NMR (500 MHz, CDCl3) of compound 35 (*partial spectrum*).

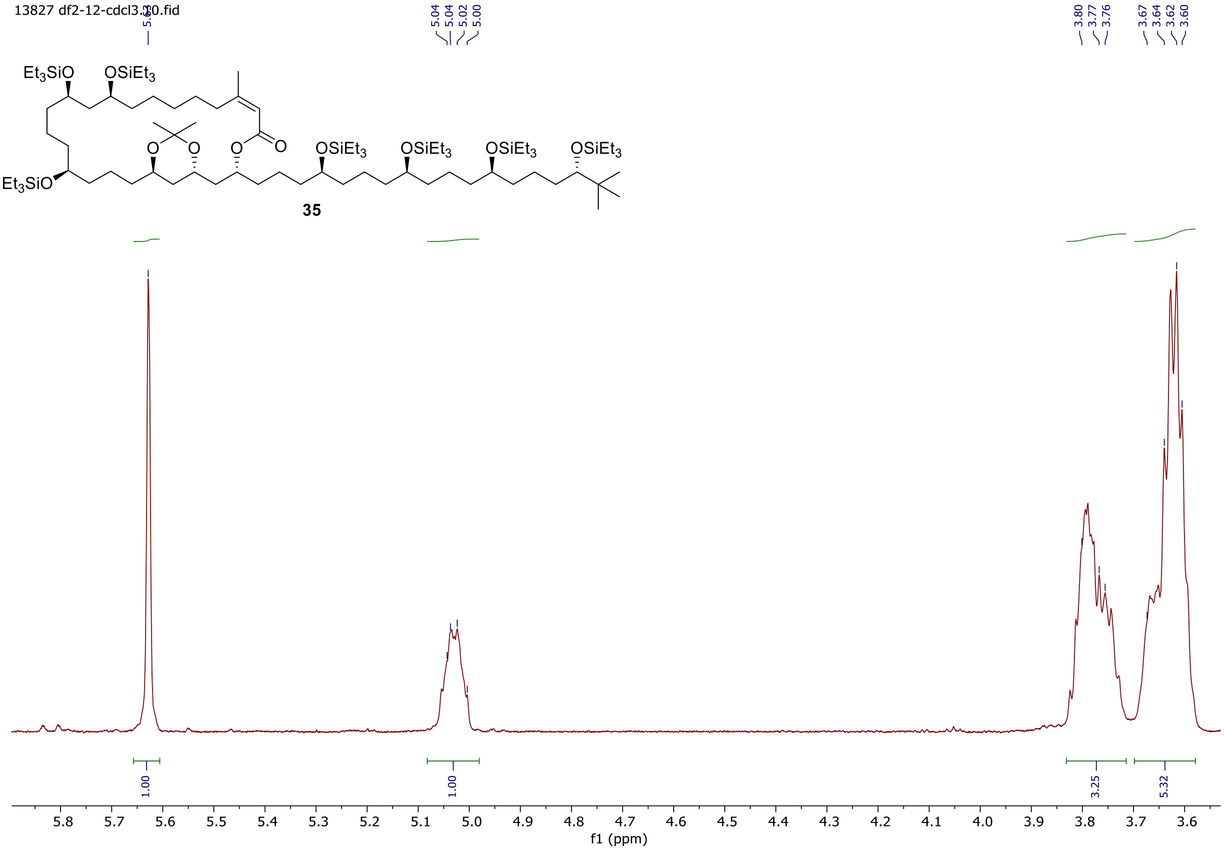

^{1}H NMR (500 MHz, CDCl$_3$) of compound 35 (*partial spectrum*).

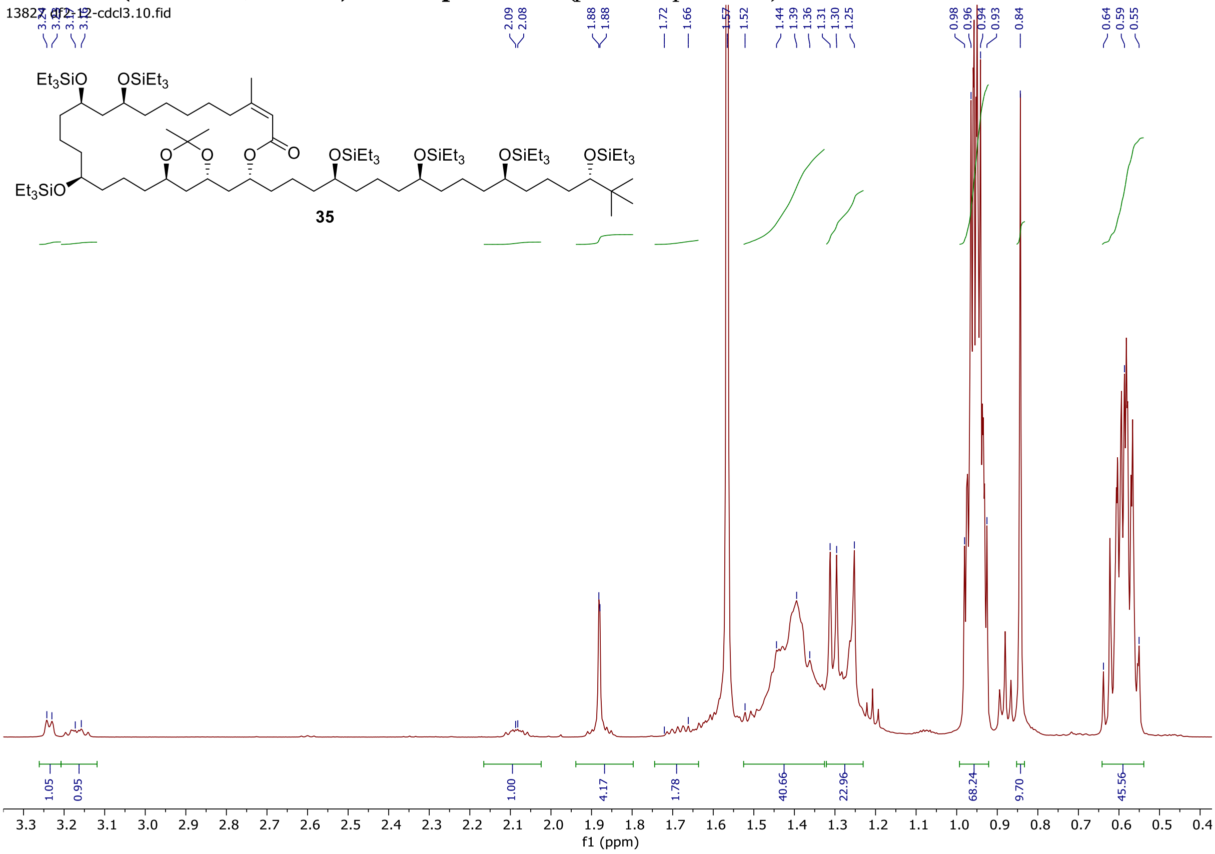

^{13}C NMR (126 MHz, CDCl$_3$) of compound 35 (*full spectrum*). See procedure.

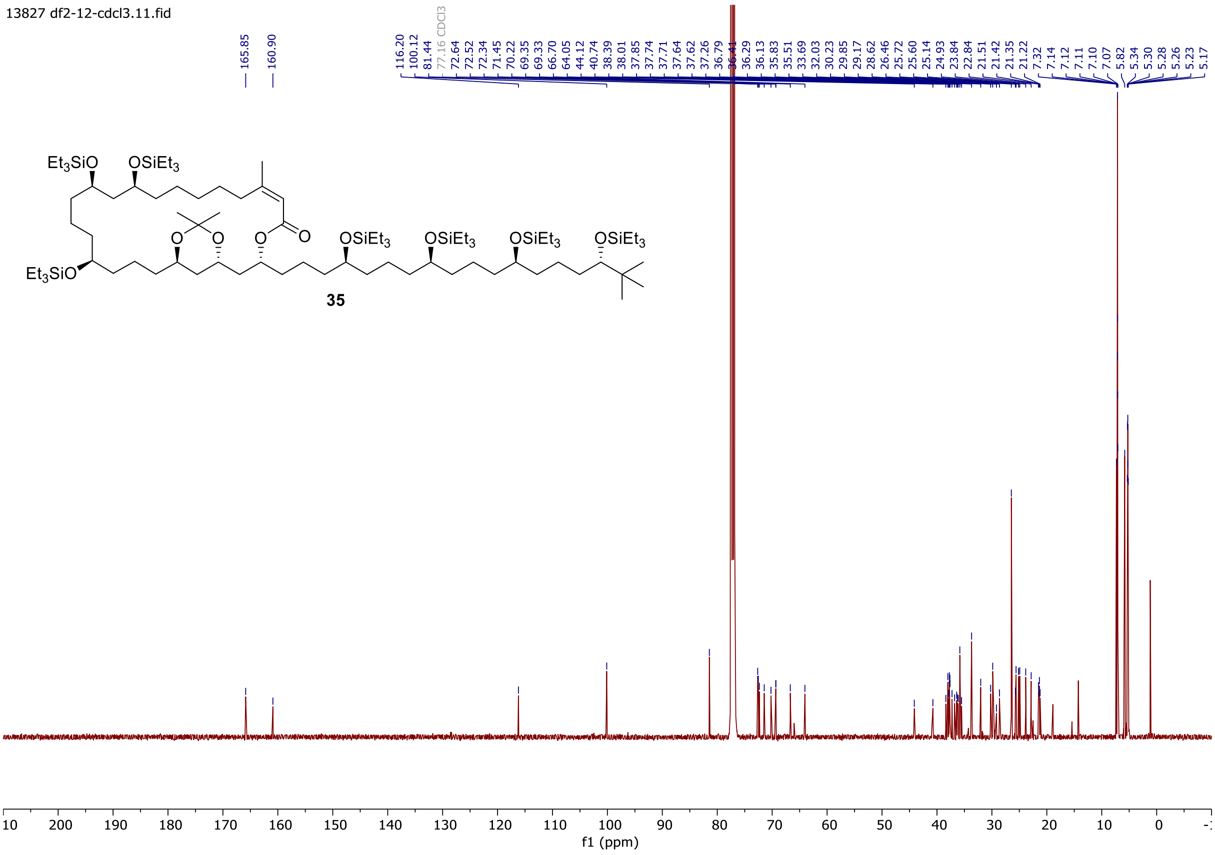

^{13}C NMR (126 MHz, $CDCl_3$) of compound 35 (*partial spectrum*).

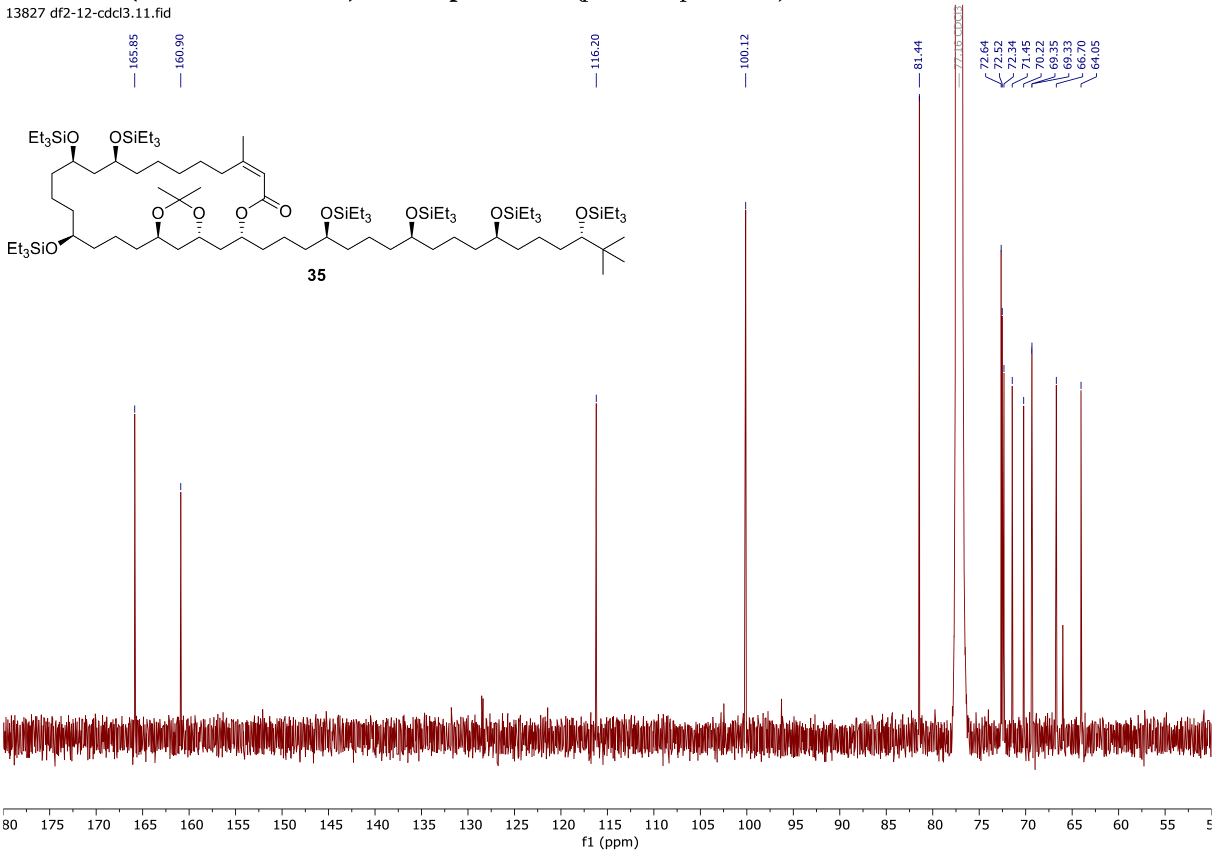

^{13}C NMR (126 MHz, $CDCl_3$) of compound 35 (*partial spectrum*).

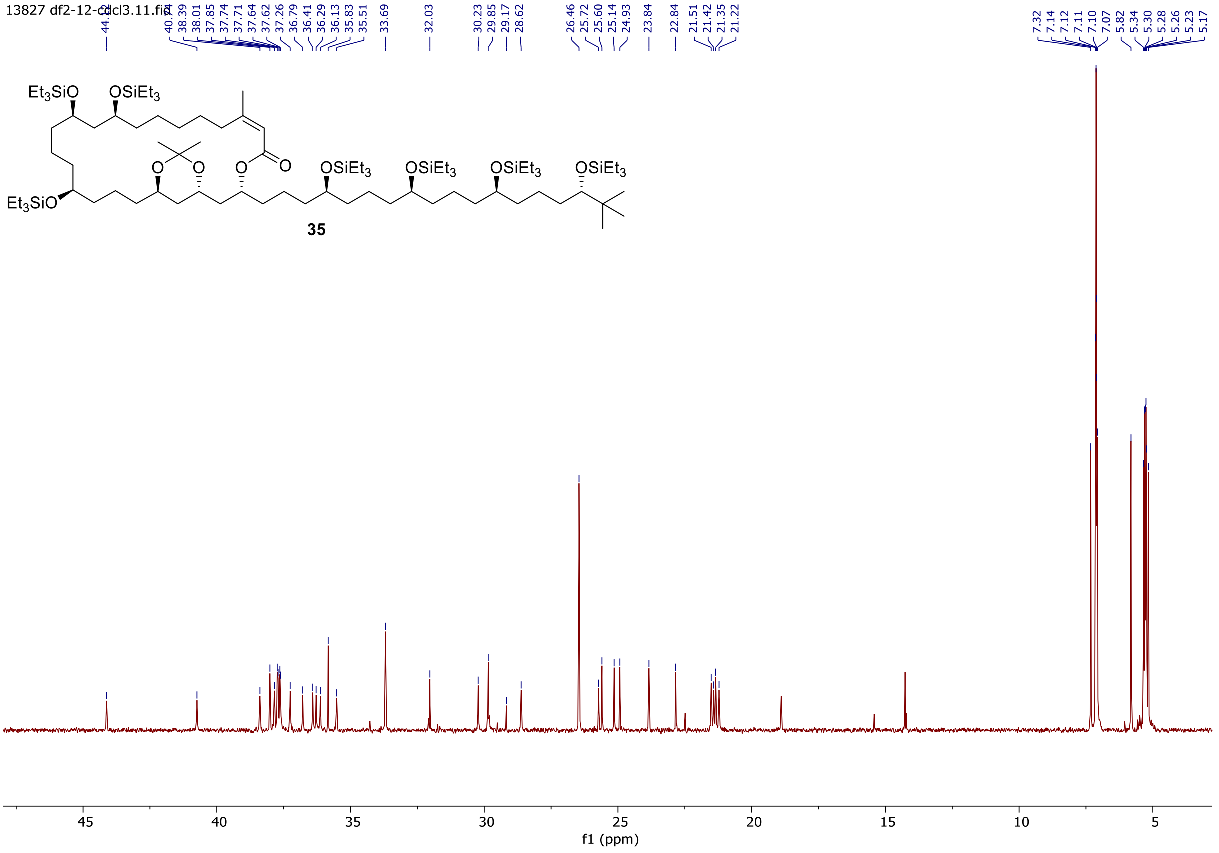

7. NMR spectra of Bastimolide B (1)

^{1}H NMR (500 MHz, CD_3OD) of synthetic bastimolide B (1, *full spectrum*). See procedure.

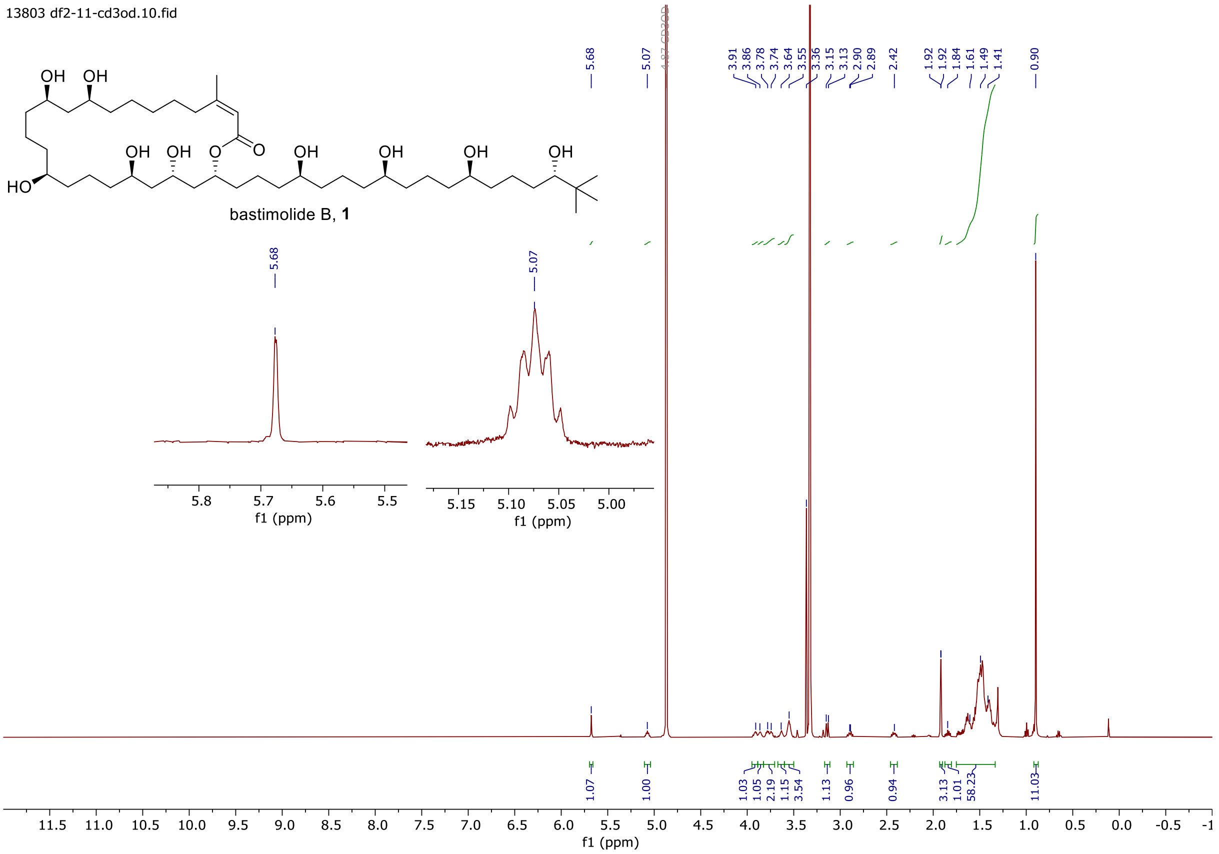

^{1}H NMR (500 MHz, CD_3OD) of synthetic bastimolide B (1, *partial spectrum*).

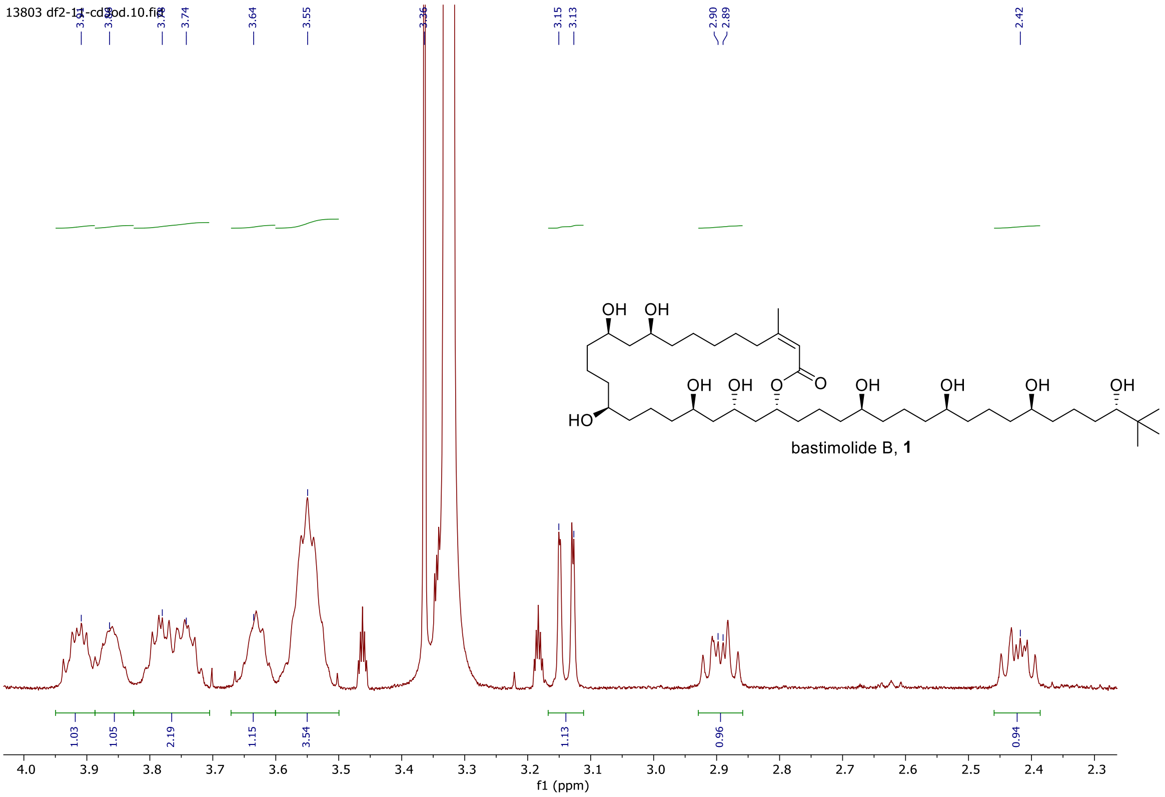

^{1}H NMR (500 MHz, CD_3COD) of synthetic bastimolide B (1, *partial spectrum*).

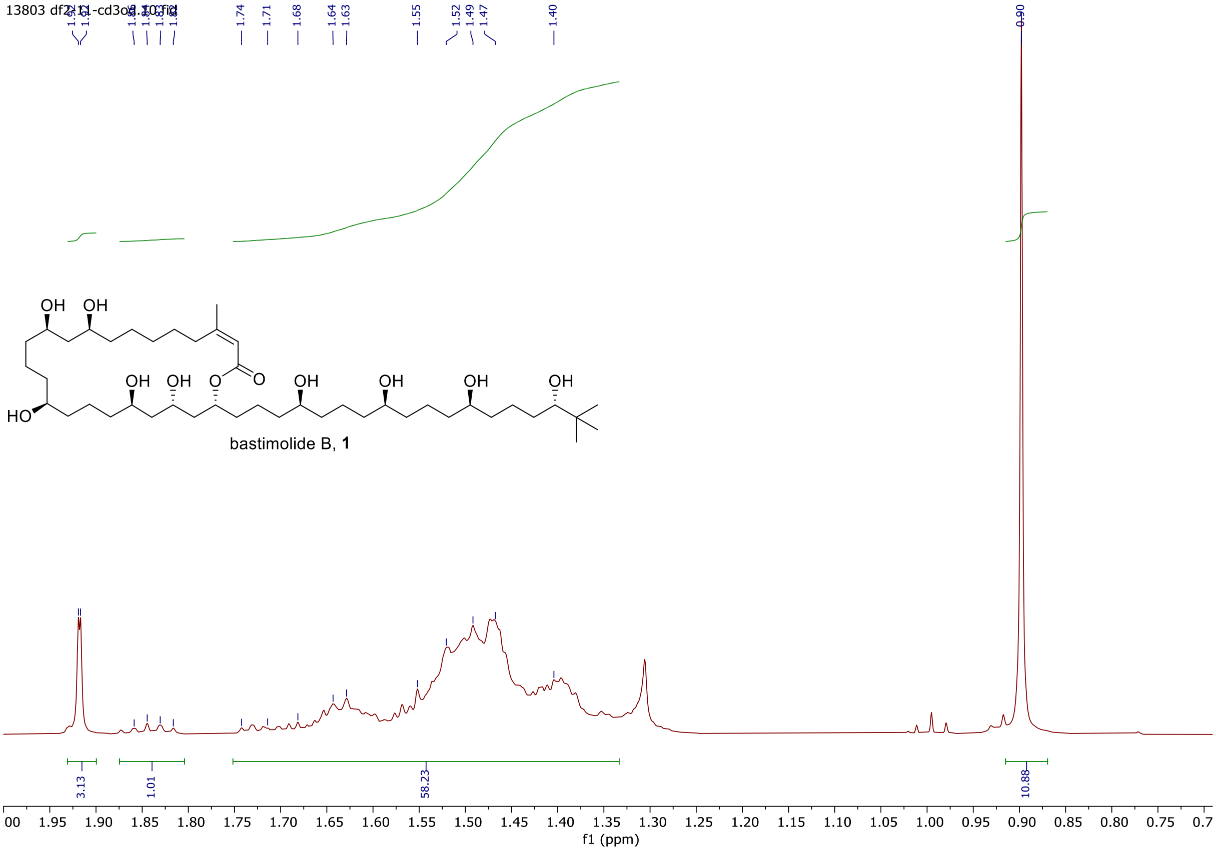

¹H NMR (500 MHz, py-*d*5) of synthetic bastimolide B (1, ***full spectrum***). See procedure.

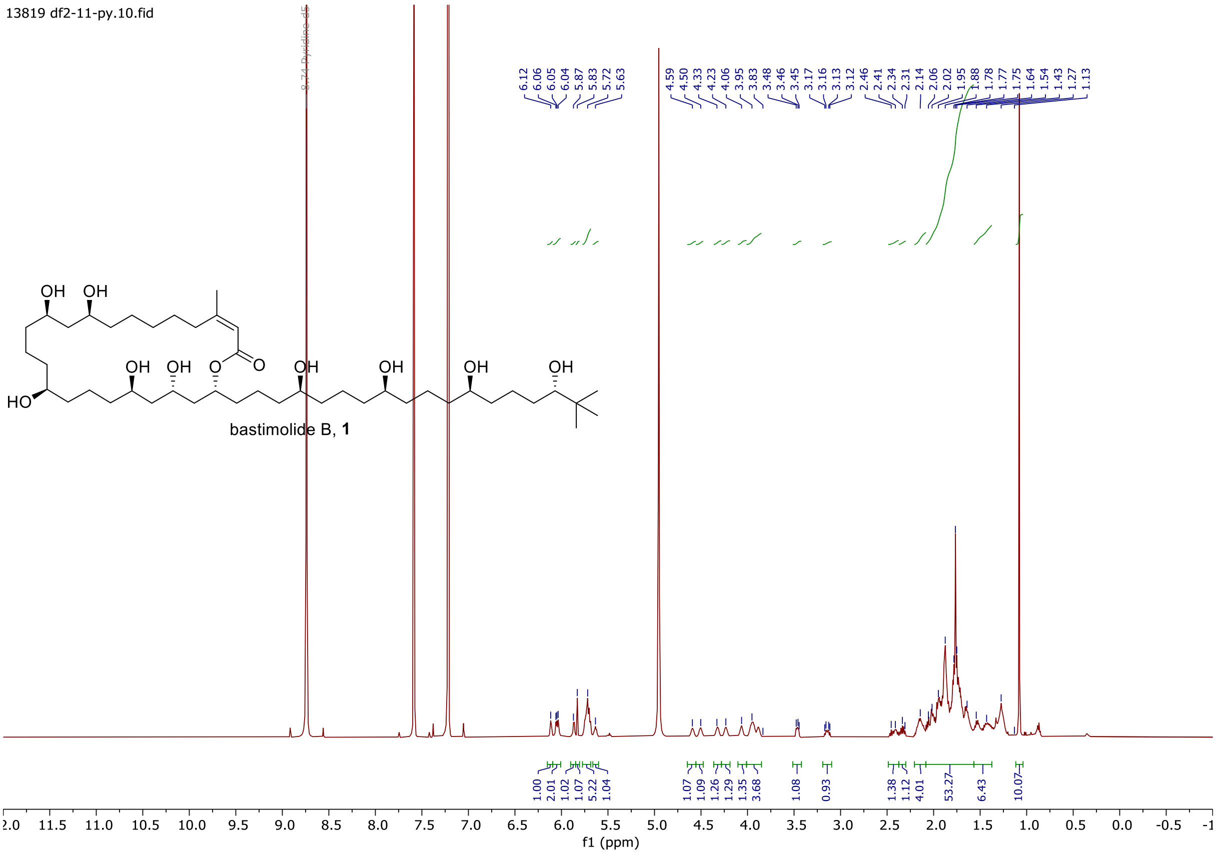

¹H NMR (500 MHz, py-*d*5) of synthetic bastimolide B (**1**, *partial spectrum*).

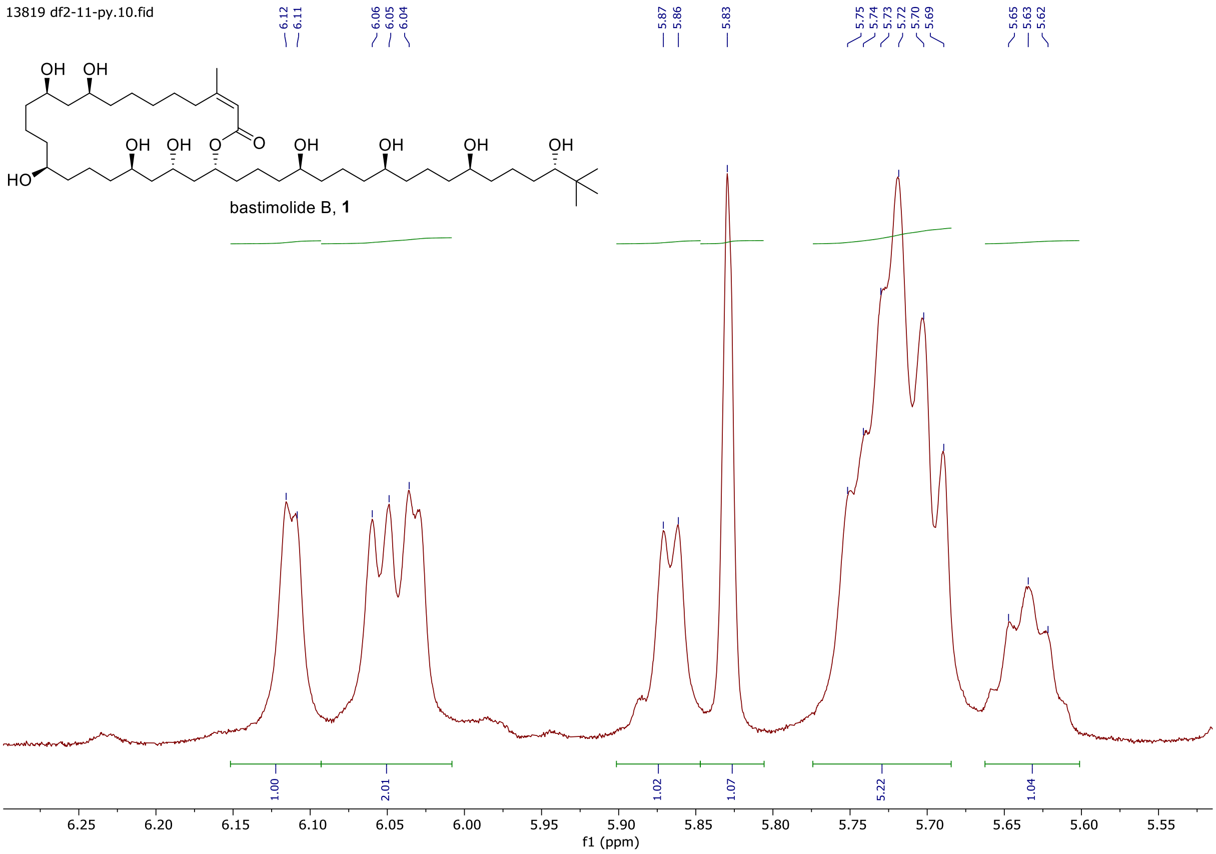

^{1}H NMR (500 MHz, py-*d*5) of synthetic bastimolide B (**1**, *partial spectrum*).

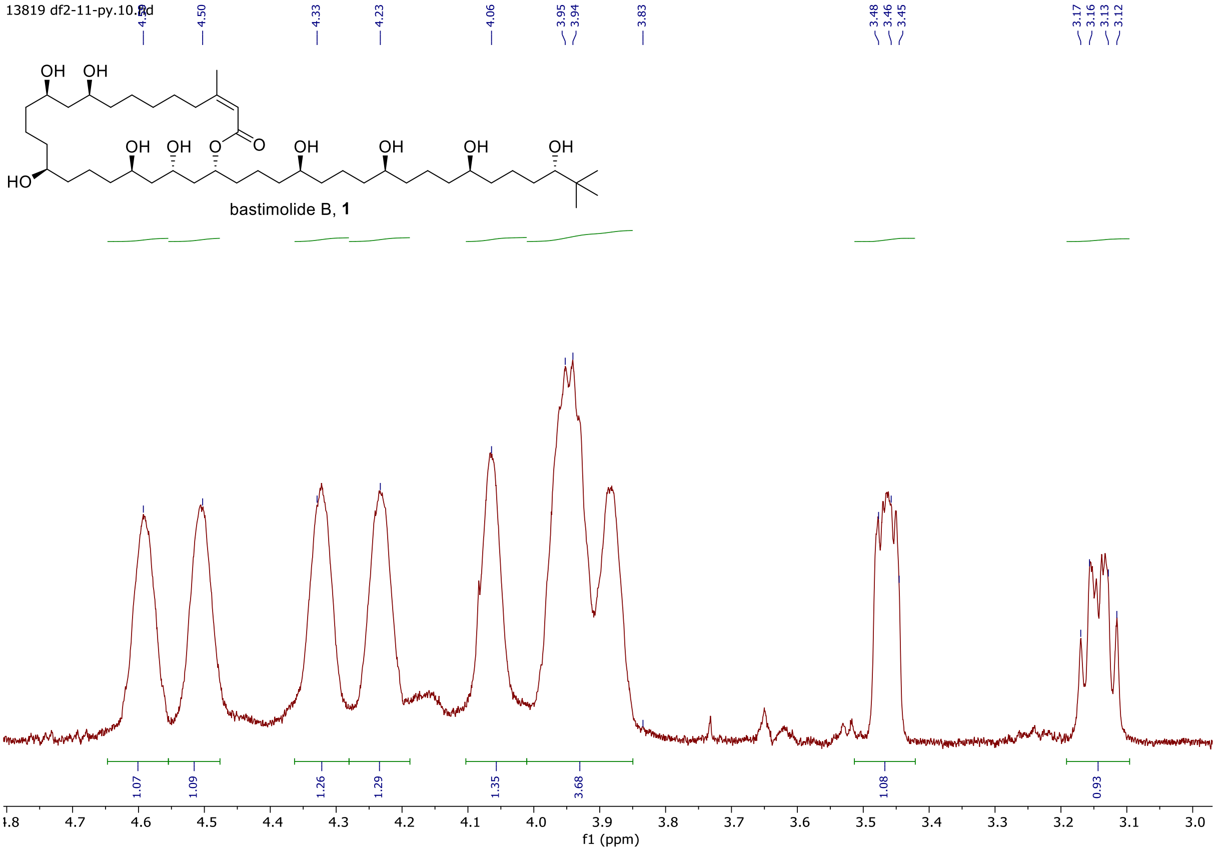

^{1}H NMR (500 MHz, py-*d*5) of synthetic bastimolide B (**1**, *partial spectrum*).

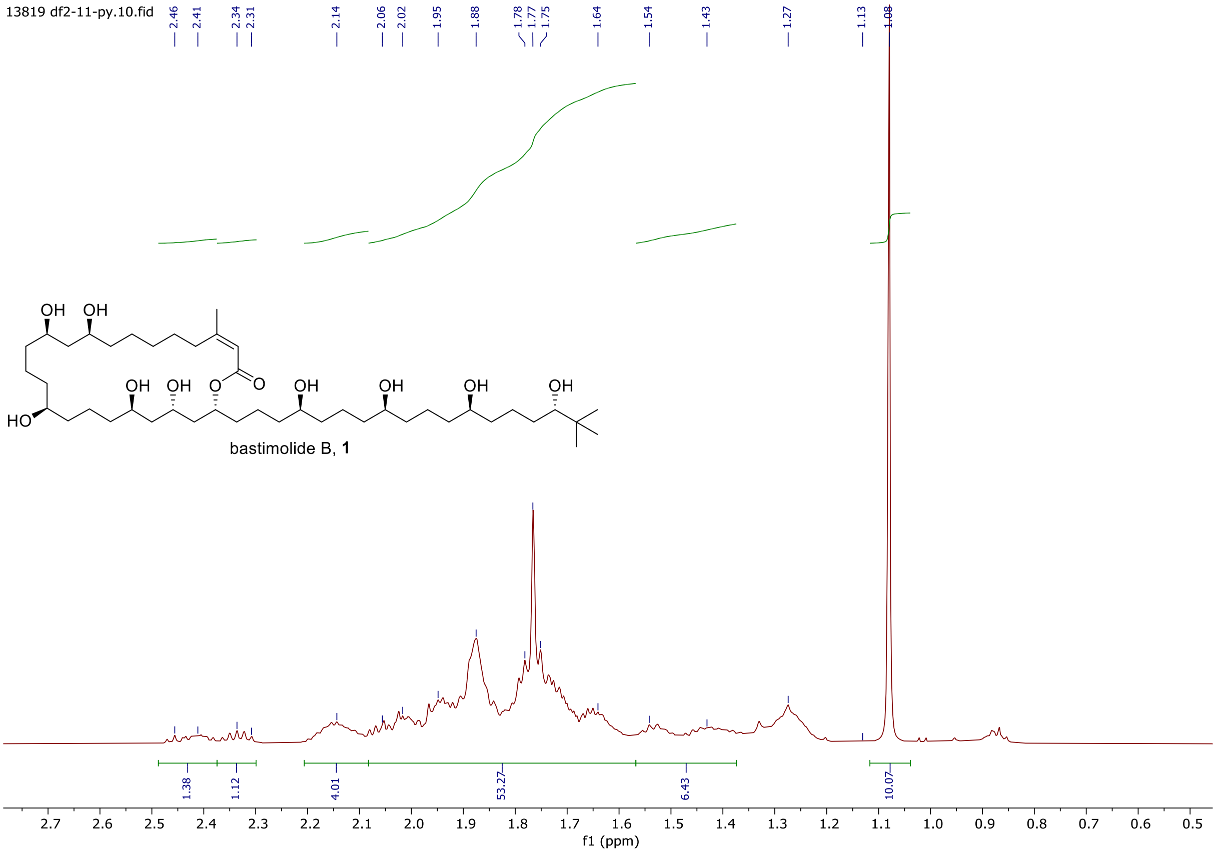

^{13}C NMR (126 MHz, CD_3OD) of synthetic bastimolide B (1, full spectrum). *See procedure*.

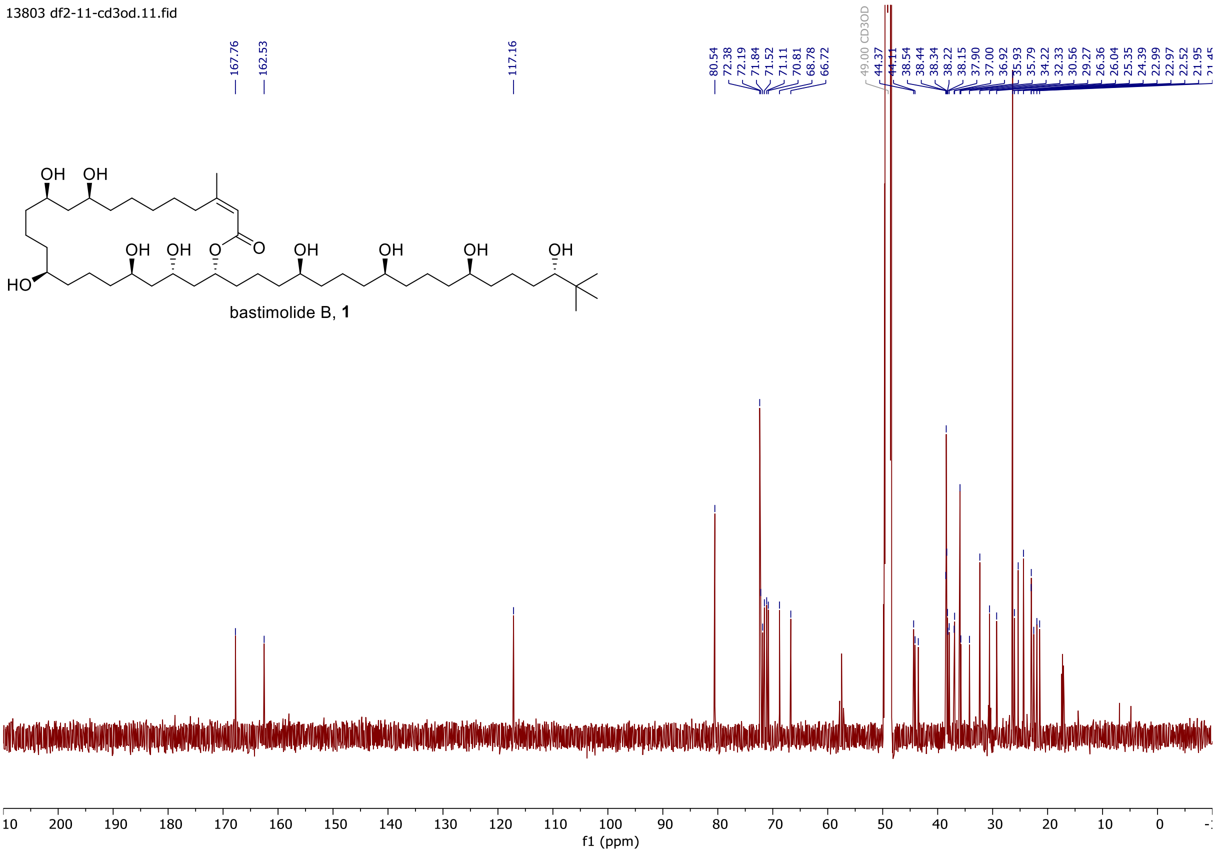

^{13}C NMR (126 MHz, CD_3OD) of synthetic bastimolide B (**1**, *partial spectrum*).

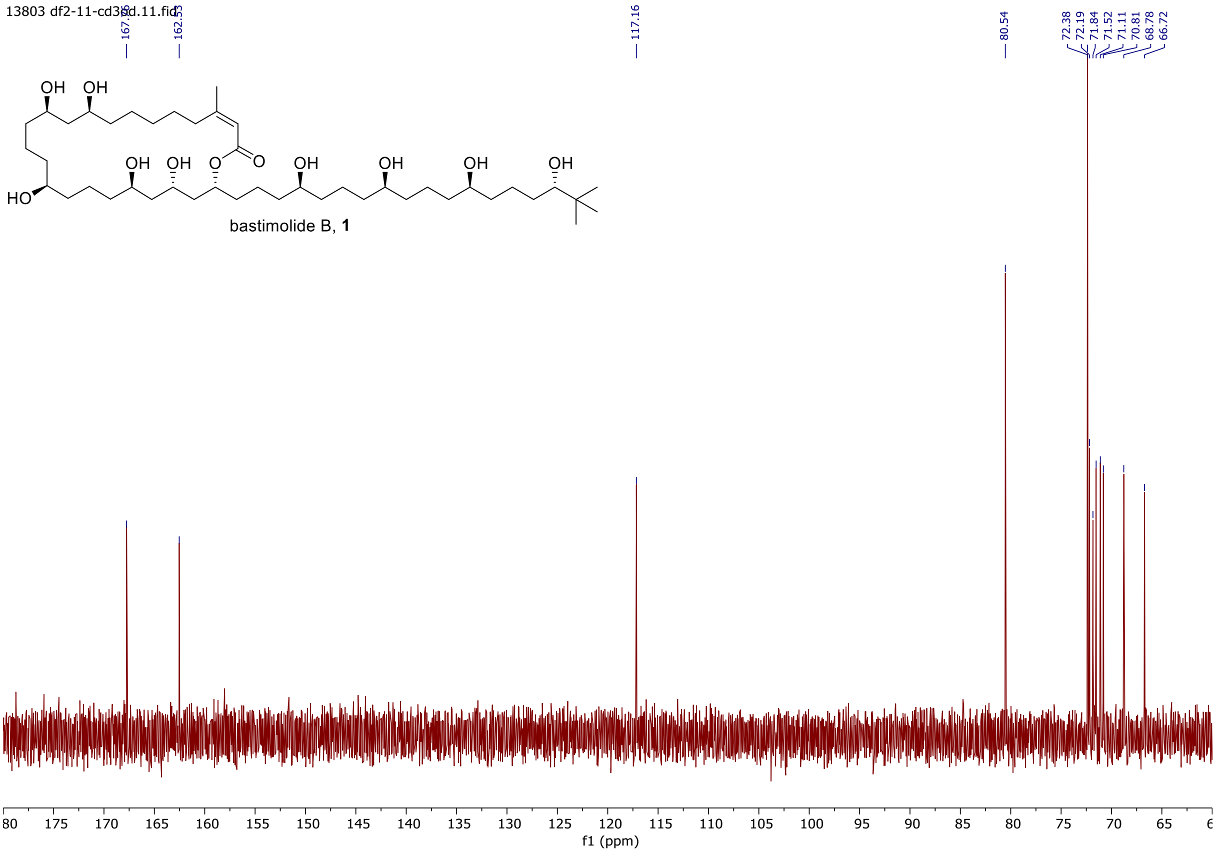

^{13}C NMR (126 MHz, CD_3OD) of synthetic bastimolide B (1, *partial spectrum*).

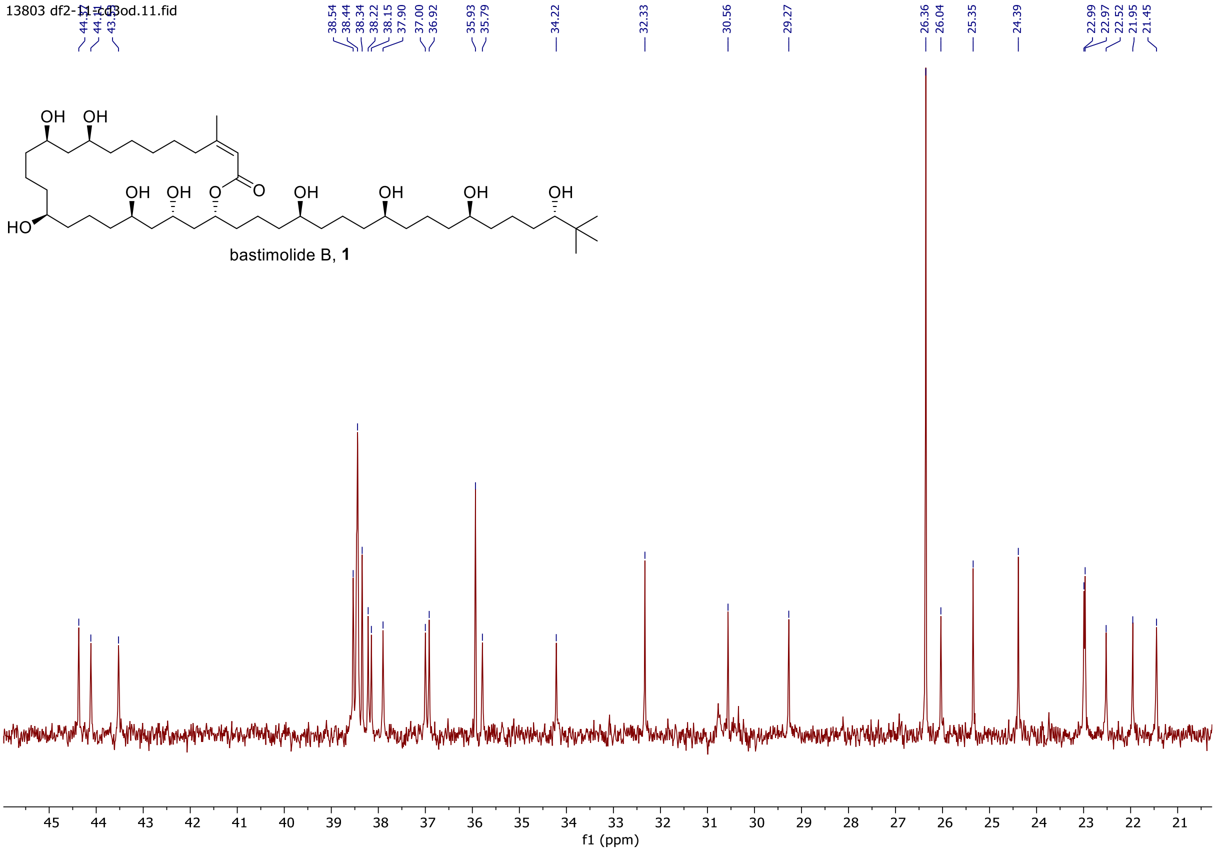

^{13}C NMR (126 MHz, py-*d*5) of synthetic bastimolide B (1, *full spectrum*). See procedure.

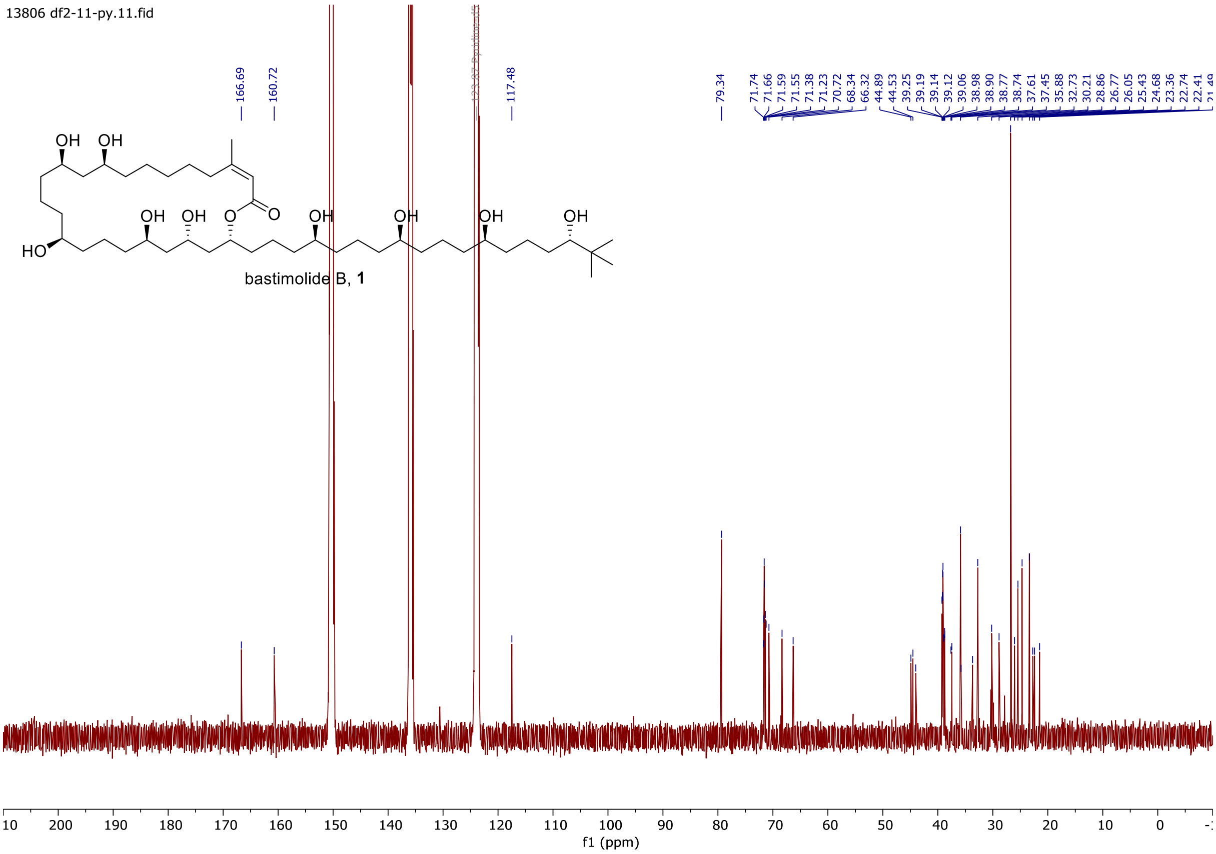

^{13}C NMR (126 MHz, py-*d*5) of synthetic bastimolide B (**1**, *partial spectrum*).

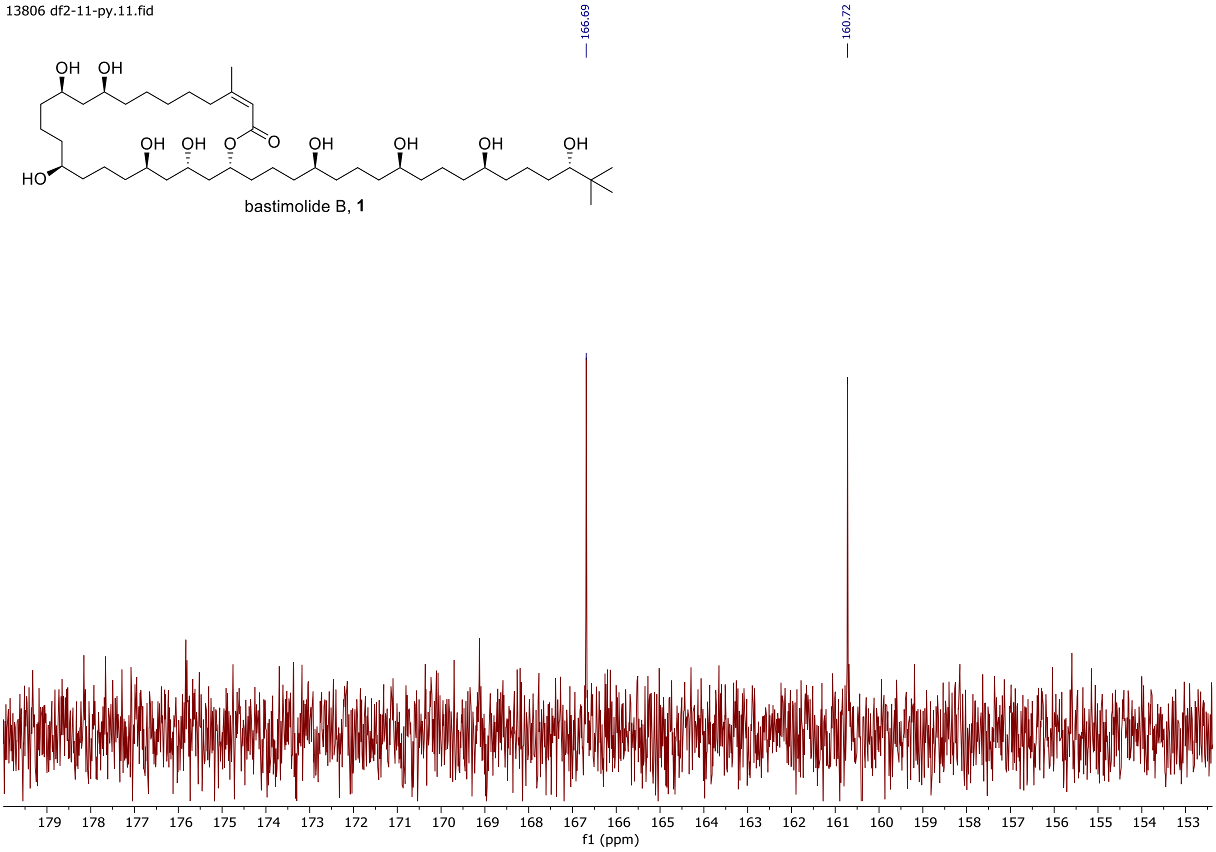

^{13}C NMR (126 MHz, py-*d*5) of synthetic bastimolide B (1, *partial spectrum*).

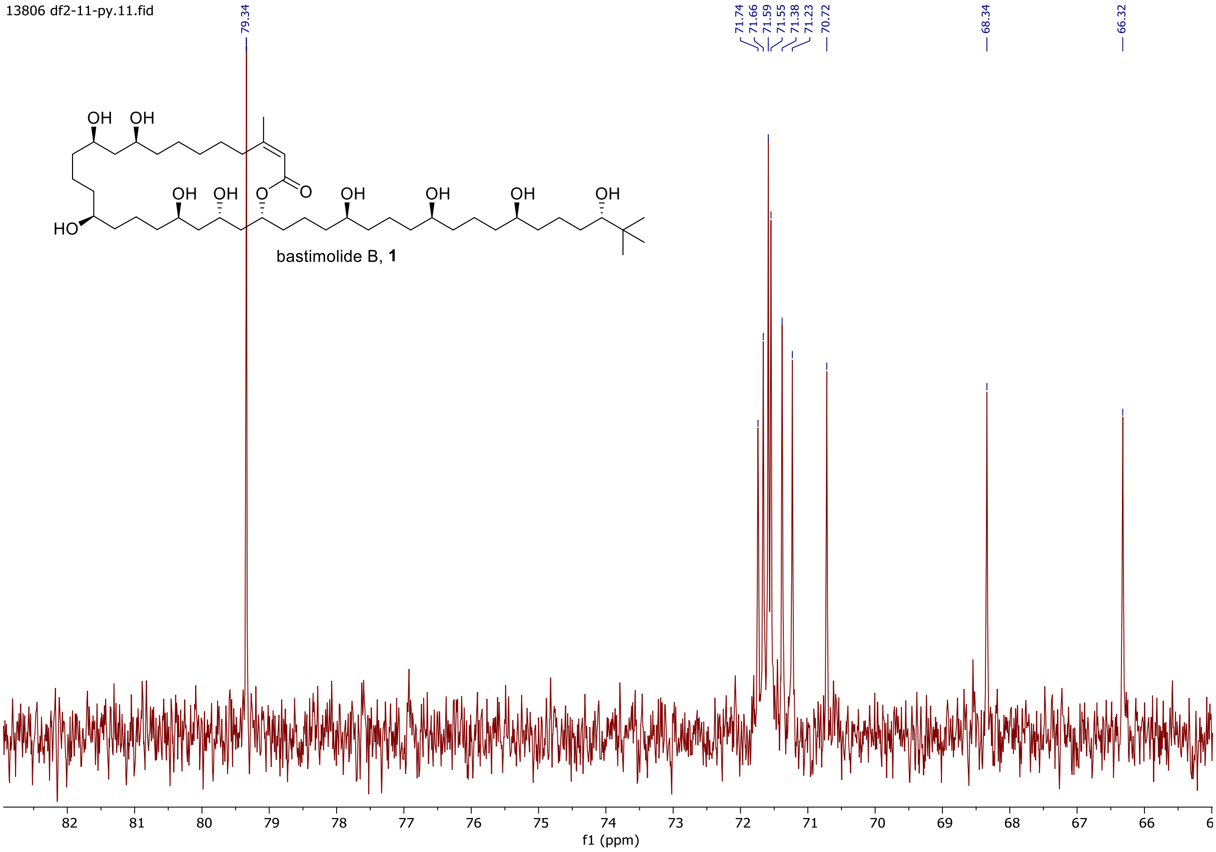

^{13}C NMR (126 MHz, py-*d*5) of synthetic bastimolide B (1, *partial spectrum*).

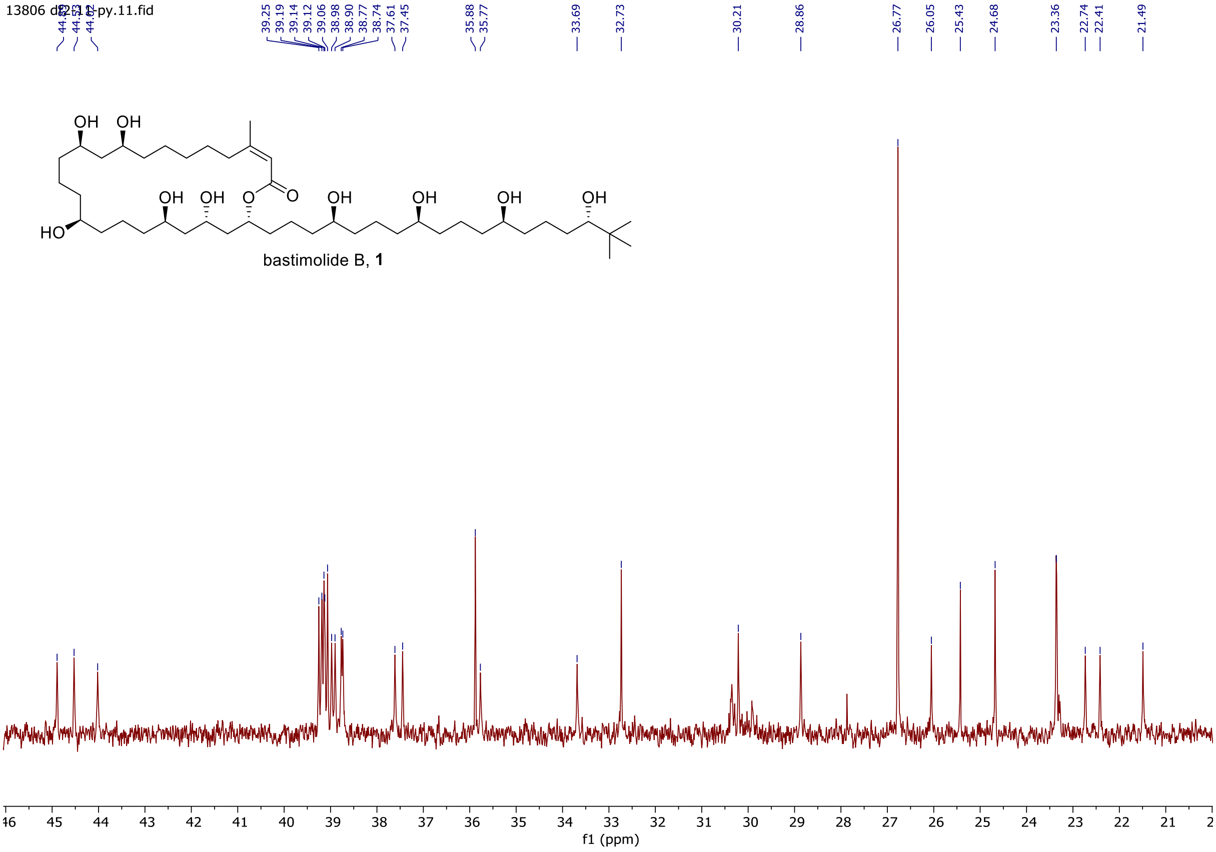

^{1}H NMR comparison of natural bastimolide B (1, top in red, 800 MHz, py-*d*5) and synthetic bastimolide B (1, bottom in green, 500 MHz, py-*d*5) (*full spectrum*). See comparison table.

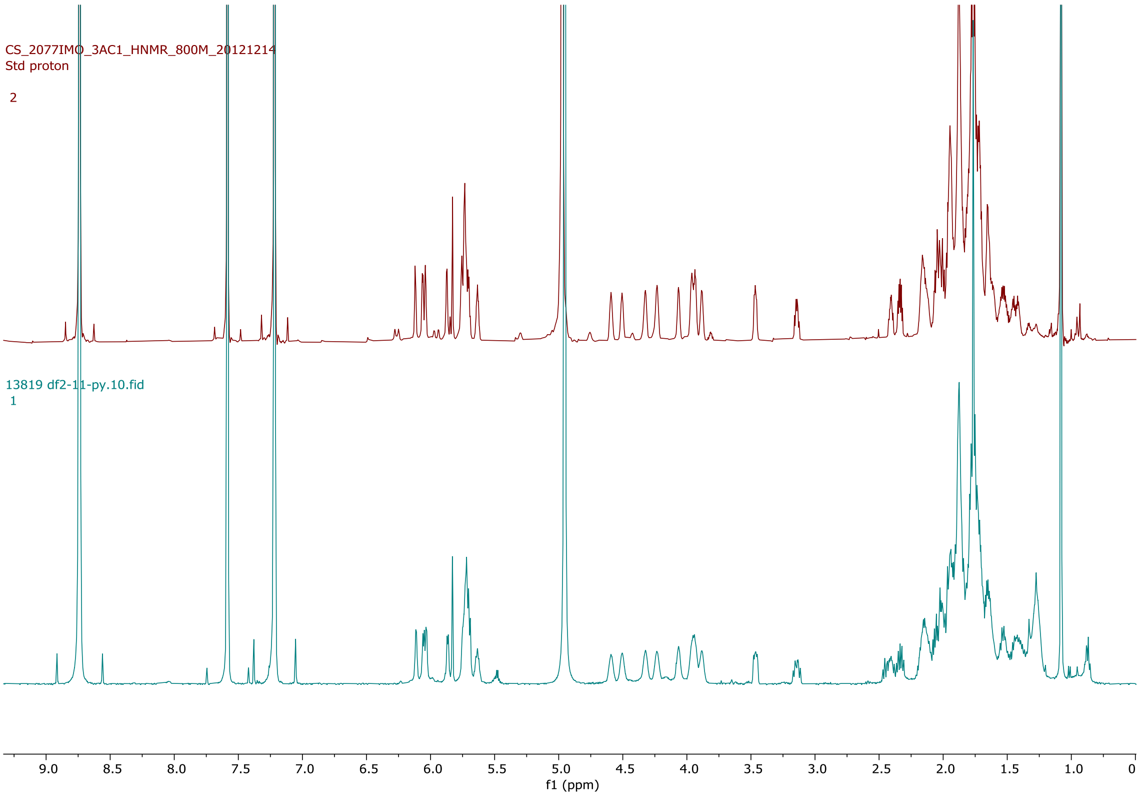

^{1}H NMR comparison of natural bastimolide B (1, top in red, 800 MHz, py-*d*5) and synthetic bastimolide B (1, bottom in green, 500 MHz, py-*d*5) (*partial spectrum*).

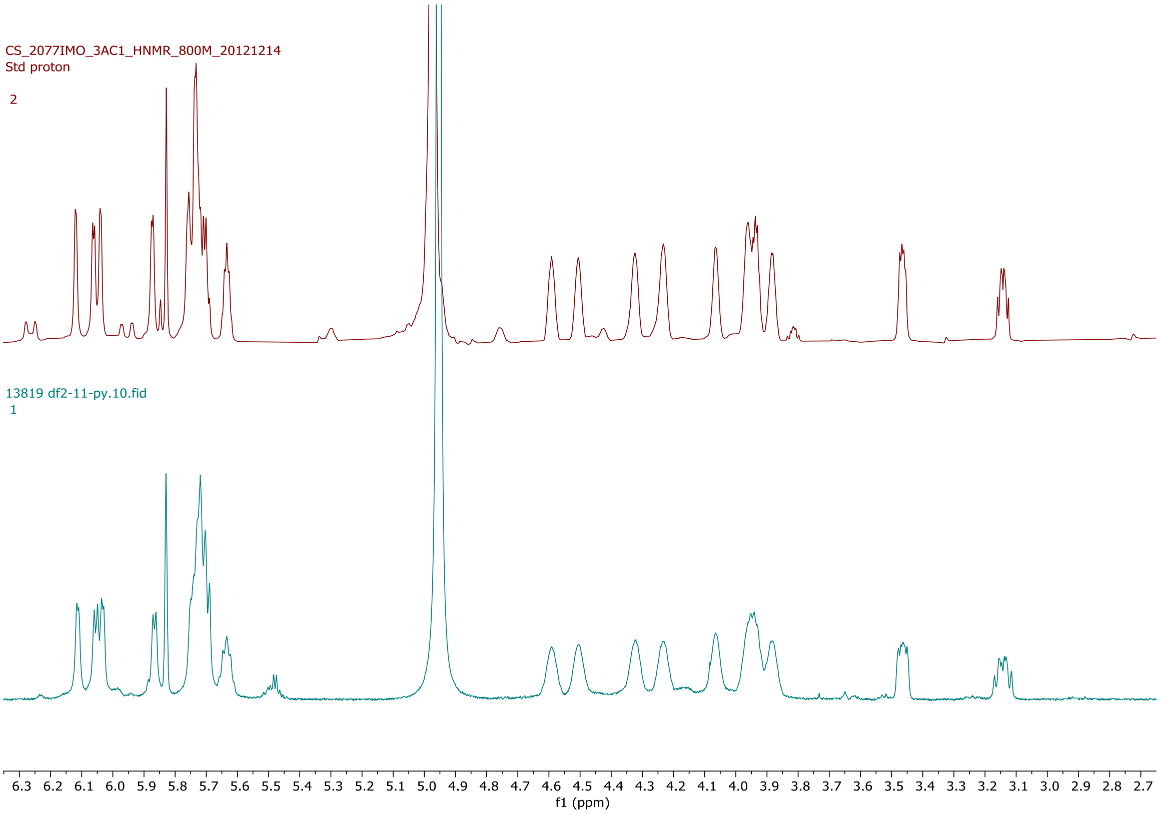

^{13}C NMR comparison of natural bastimolide B (1, top in red, 200 MHz, py-*d*5) and synthetic bastimolide B (1, bottom in green, 126 MHz, py-*d*5) (full spectrum). *See comparison table*.

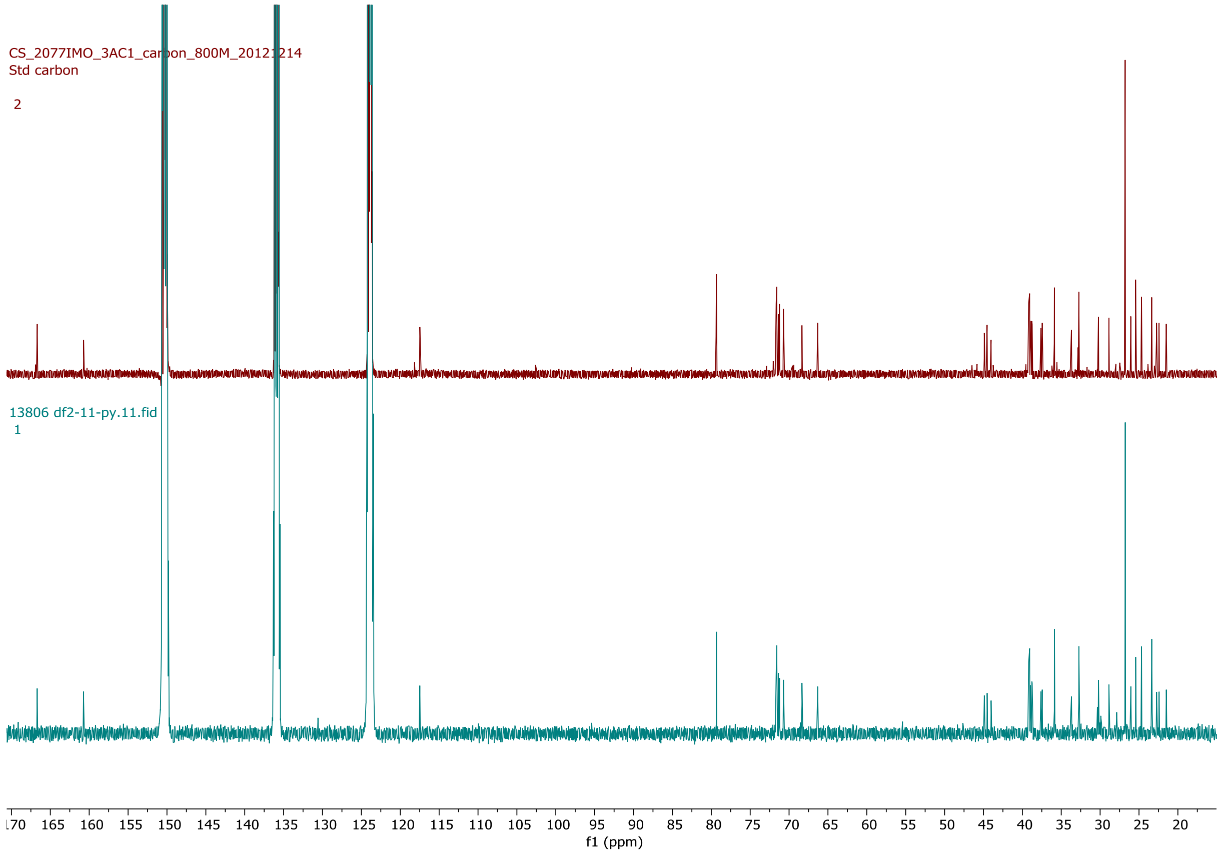

^{13}C NMR comparison of natural bastimolide B (1, top in red, 200 MHz, py-*d*5) and synthetic bastimolide B (1, bottom in green, 126 MHz, py-*d*5) (*partial spectrum*).

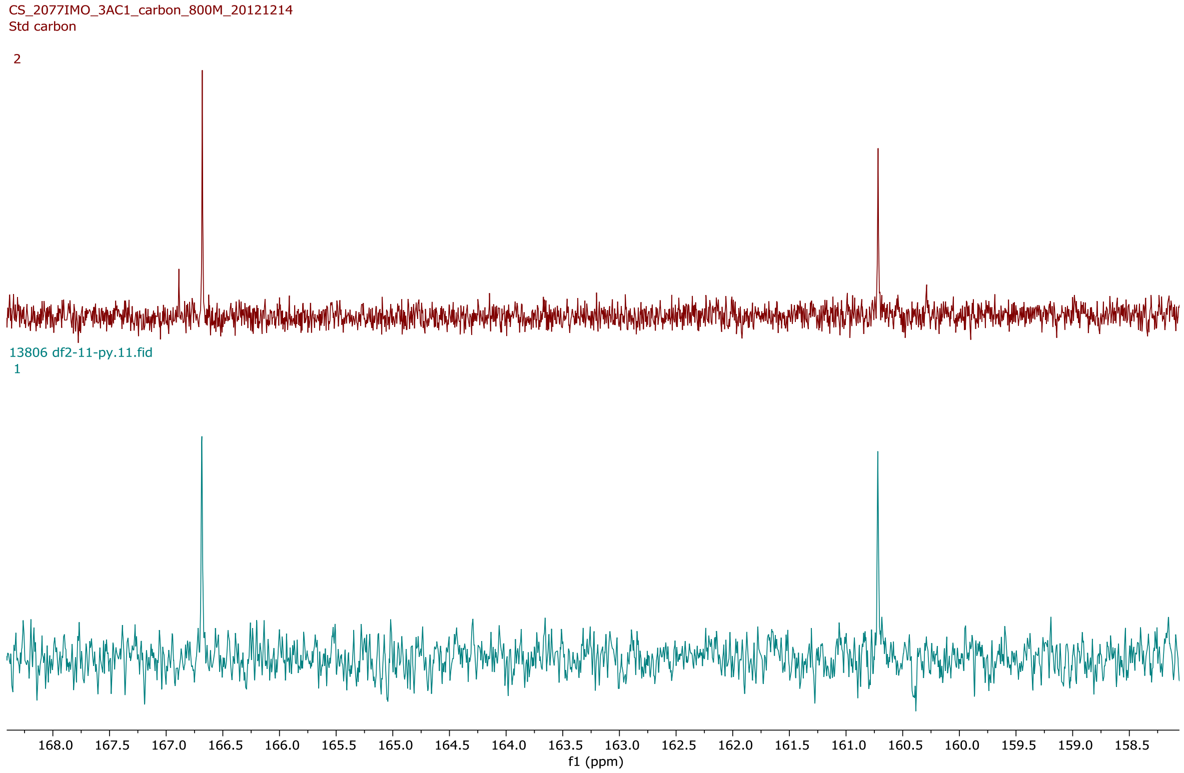

^{13}C NMR comparison of natural bastimolide B (1, top in red, 200 MHz, py-*d*5) and synthetic bastimolide B (1, bottom in green, 126 MHz, py-*d*5) (*partial spectrum*).

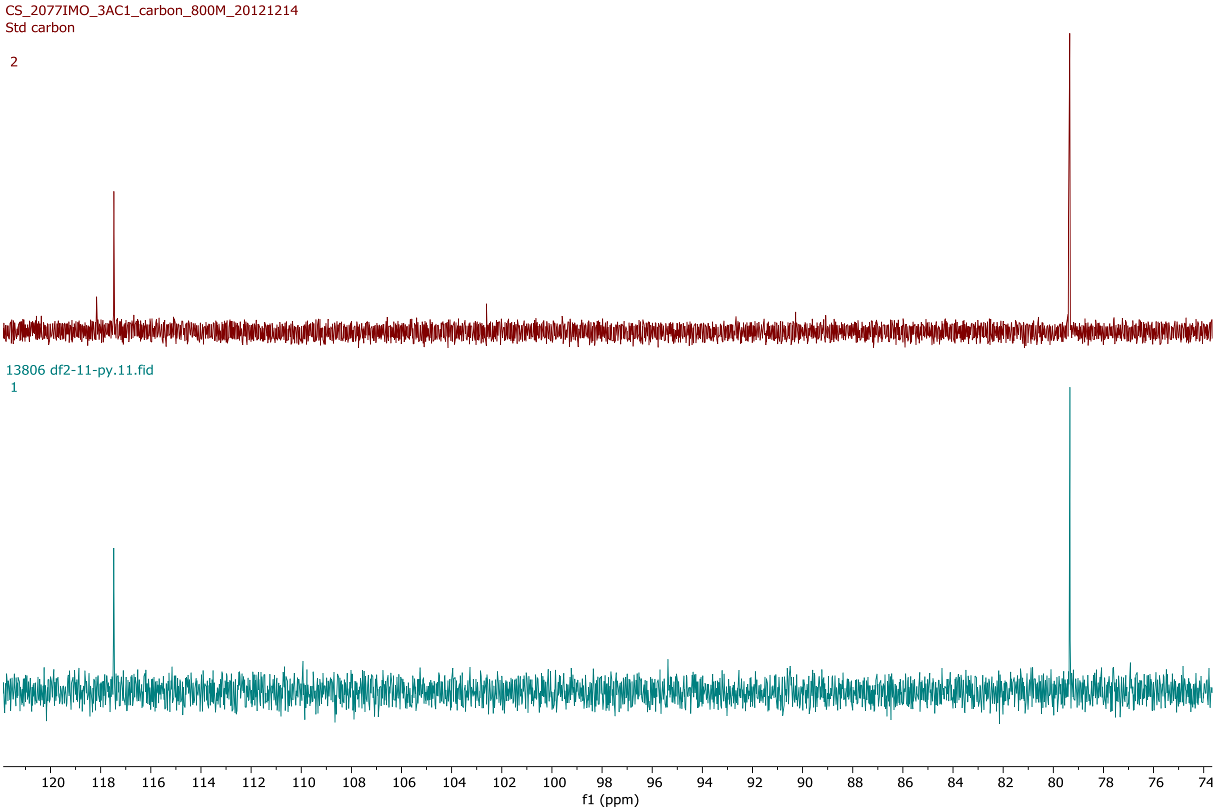

^{13}C NMR comparison of natural bastimolide B (1, top in red, 200 MHz, py-*d*5) and synthetic bastimolide B (1, bottom in green, 126 MHz, py-*d*5) (*partial spectrum*).

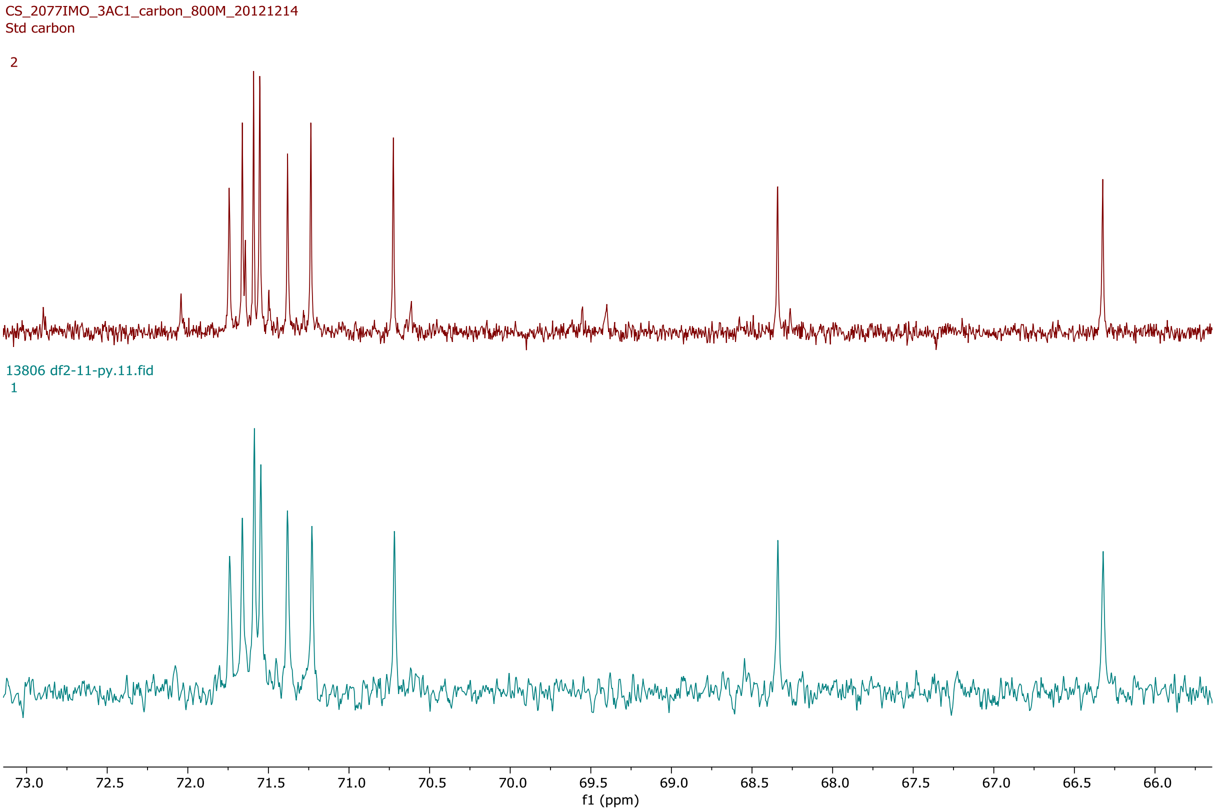

^{13}C NMR comparison of natural bastimolide B (1, top in red, 200 MHz, py-d5) and synthetic bastimolide B (1, bottom in green, 126 MHz, py-d5) (*partial spectrum*).

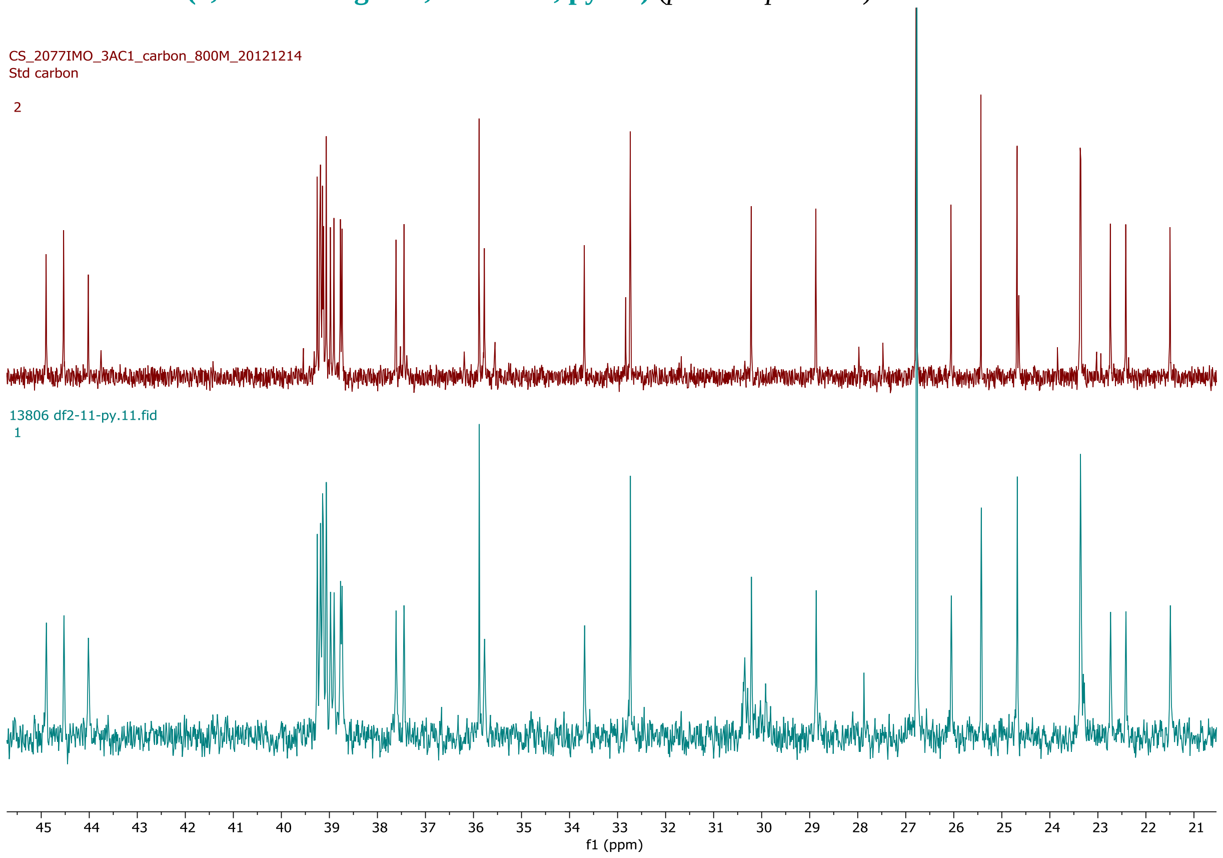

NMR Spectra for the Structural Assignment of Bastimolide B (1). *See tables*.

¹H NMR (700 MHz, py-*d*5) of synthetic Bastimolide B (1)

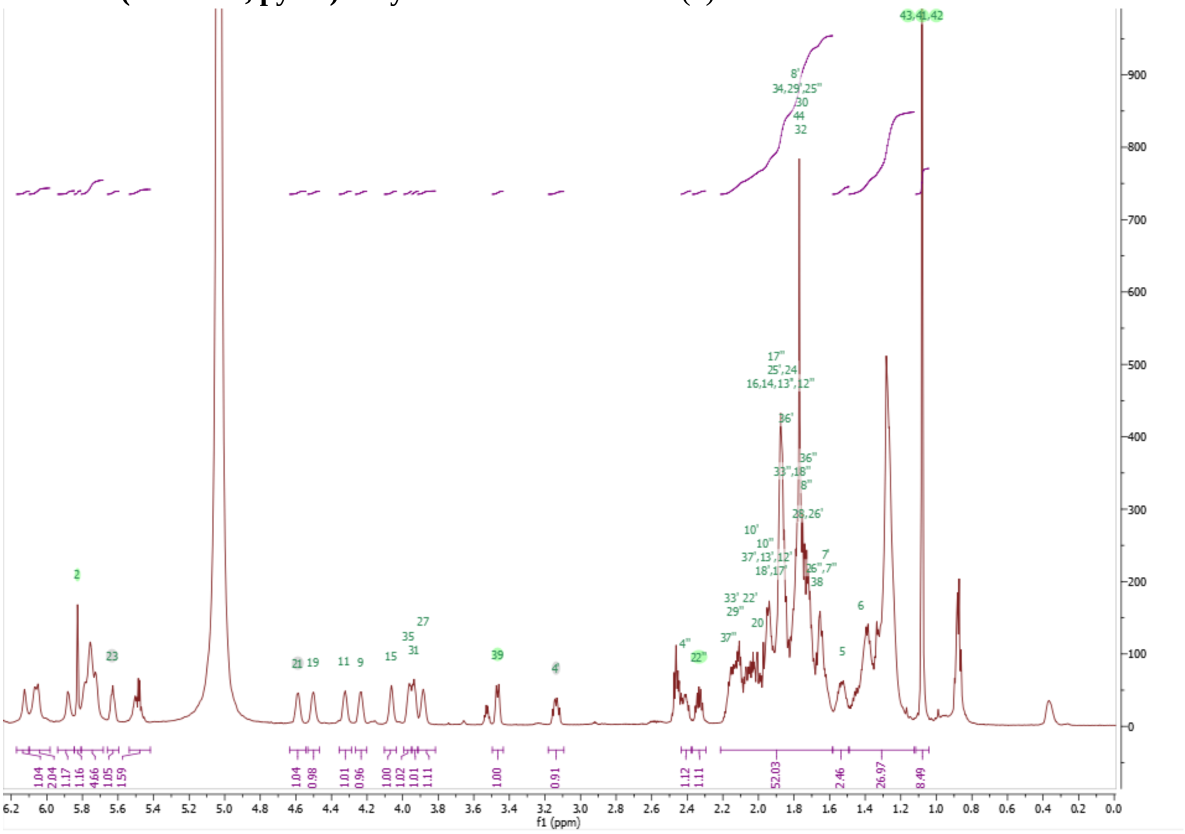

HMBC (700 MHz (¹H), 176 MHz (¹³C), py-*d*5) of synthetic Bastimolide B (1)

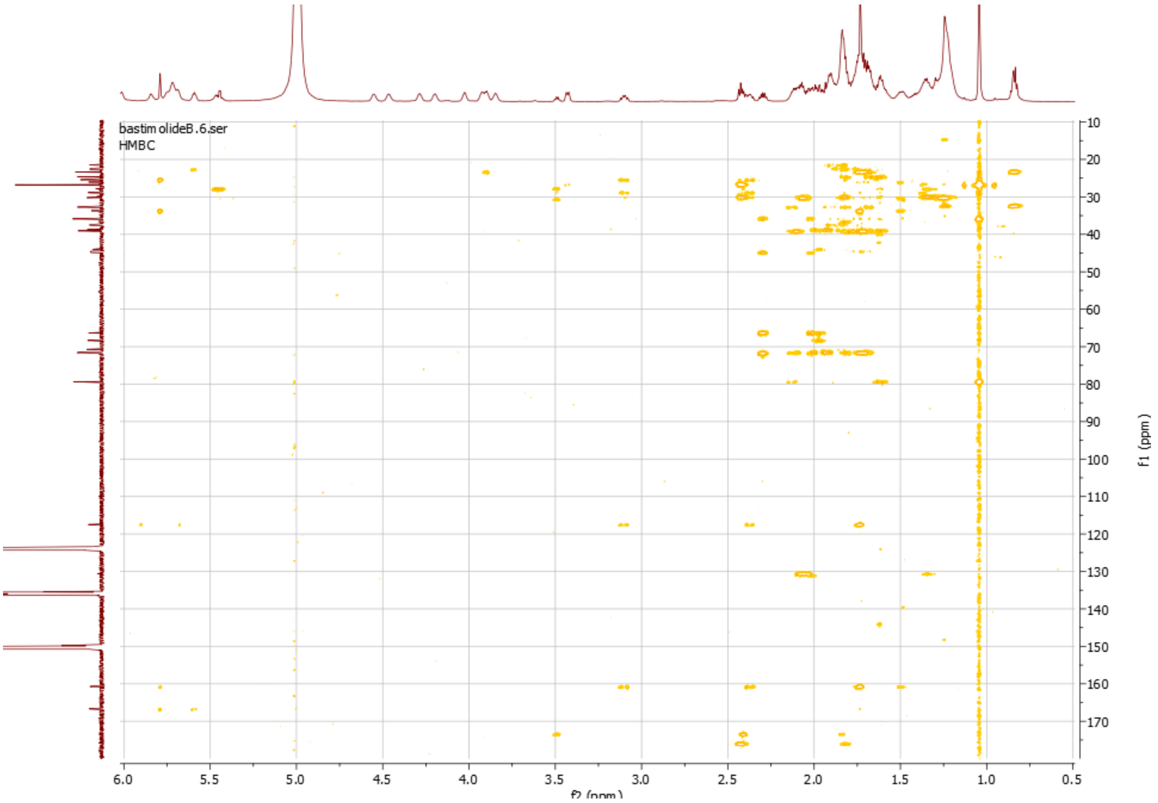

HSQC (700 MHz (^{1}H), 176 MHz (^{13}C), py-*d*5) of synthetic Bastimolide B (1, *aliased to 30 ppm centred at 36 ppm)*

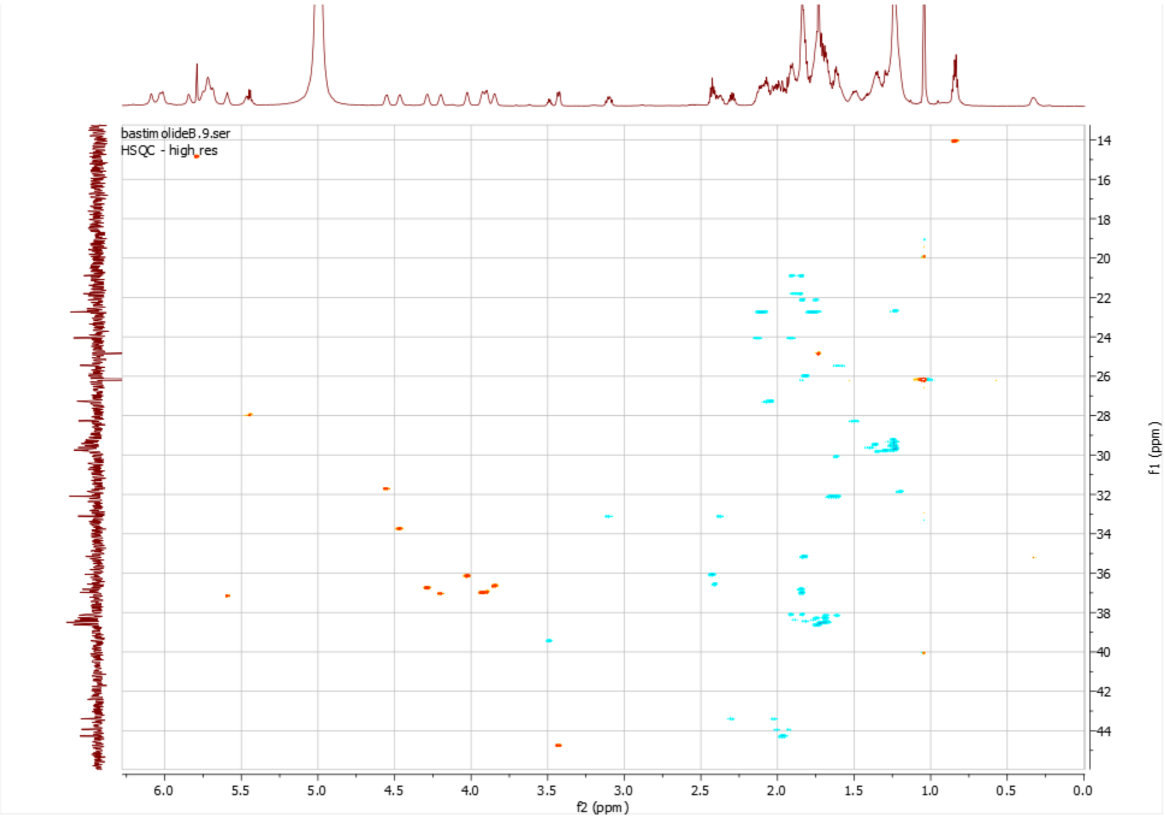

HSQC (700 MHz (^{1}H), 176 MHz (^{13}C), py-*d*5) of synthetic Bastimolide B (1)

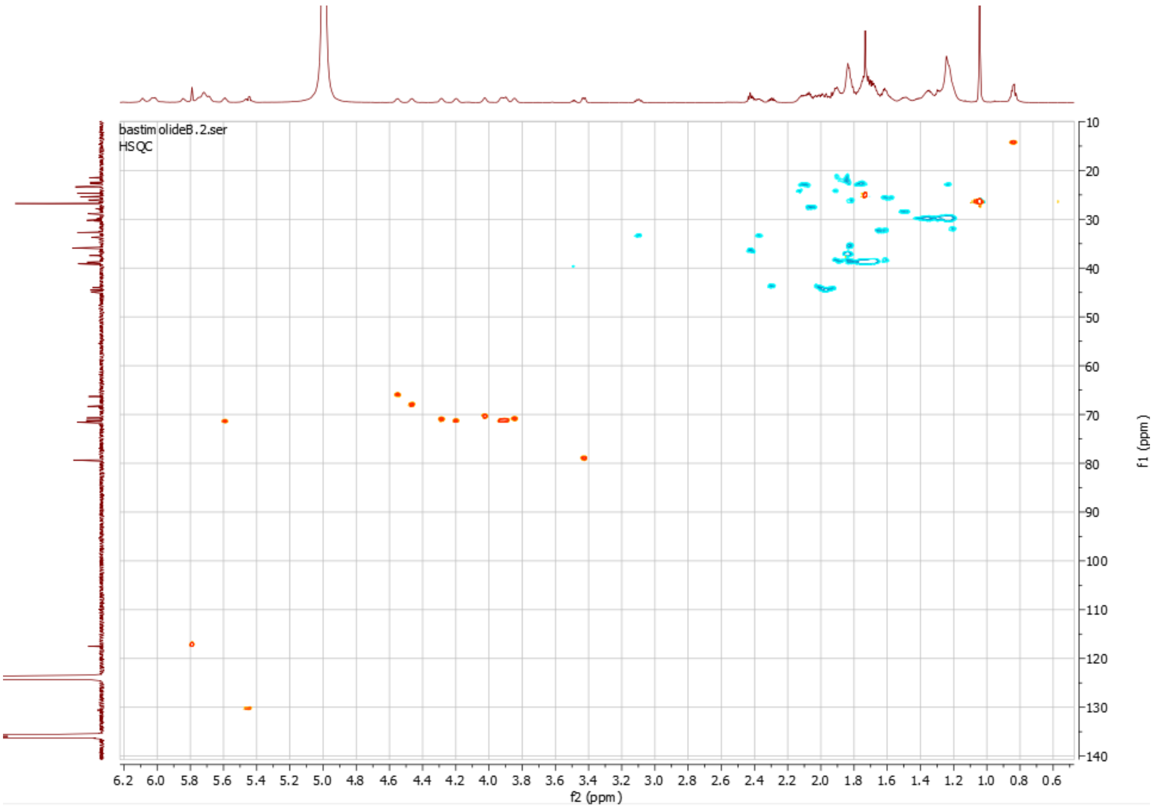

HSQC-TOCSY (60 ms mixing time) (700 MHz (^{1}H), 176 MHz (^{13}C), py-*d*5) of synthetic Bastimolide B (**1**, *aliased to 30 ppm centred at 36 ppm)*

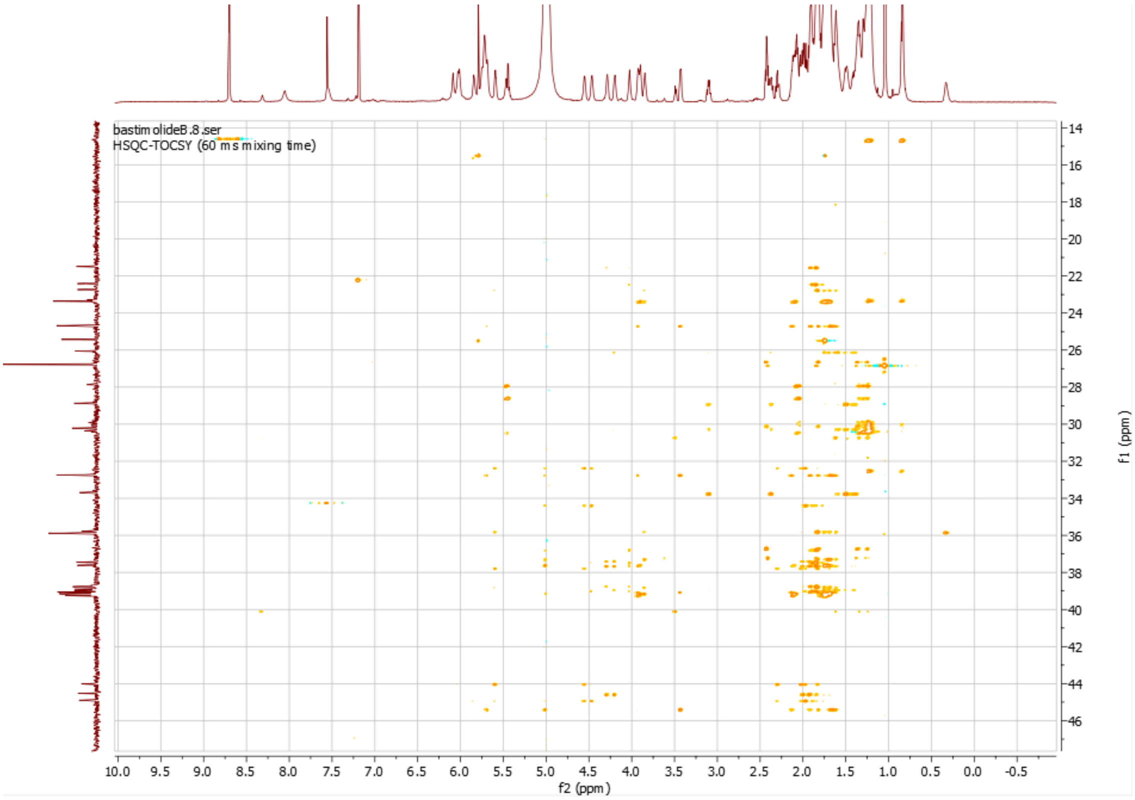

HSQC-TOCSY (30 ms mixing time) (700 MHz (^{1}H), 176 MHz (^{13}C), py-*d*5) of synthetic Bastimolide B (**1**, *aliased to 30 ppm centred at 36 ppm)*

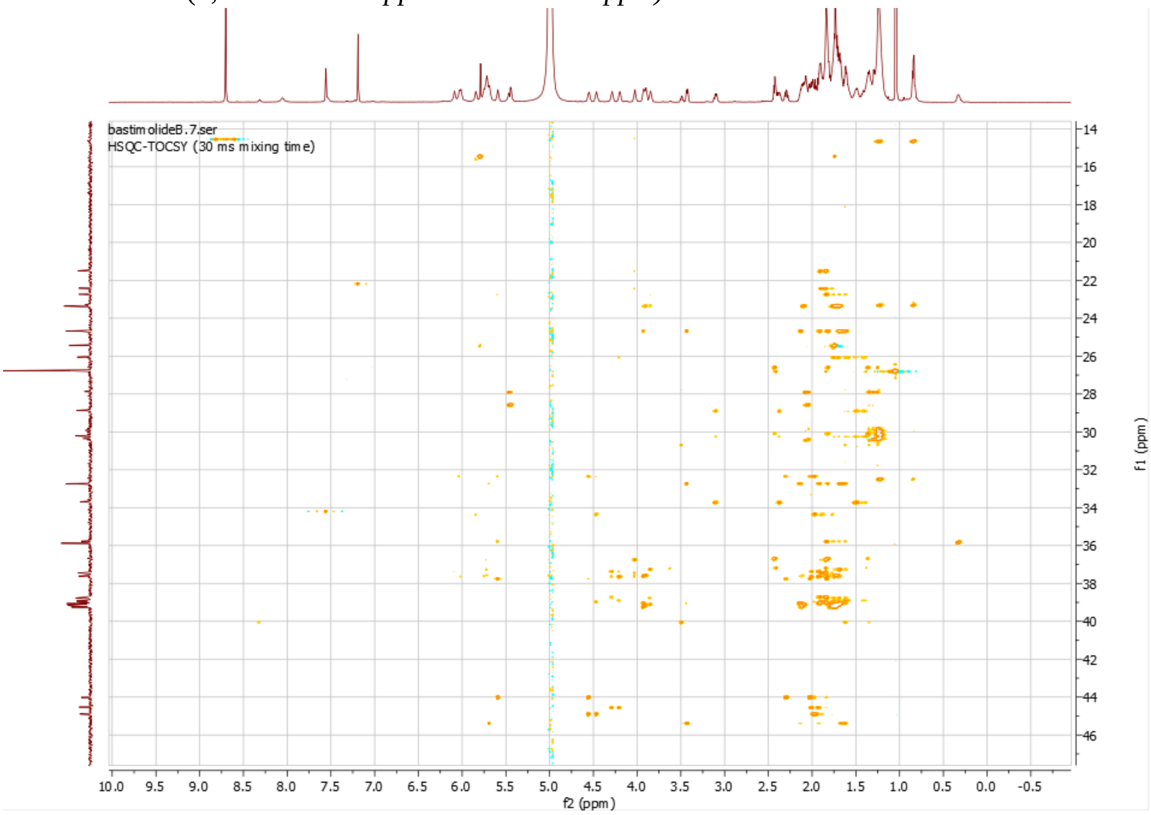

HSQC-TOCSY (30 ms mixing time) (700 MHz (^{1}H), 176 MHz (^{13}C), py-*d*5) of synthetic Bastimolide B (**1**, *aliased to 30 ppm centred at 36 ppm)*

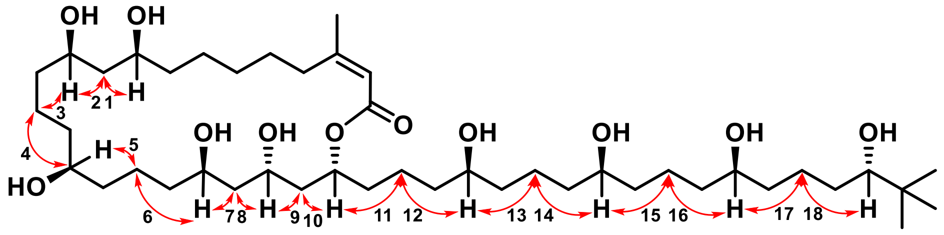

The numbering refers to the HSQC-TOCSY correlation, and it does not refer to the molecule atom numbering.

HSQC-TOCSY (30 ms mixing time) (700 MHz (^{1}H), 176 MHz (^{13}C), py-d5) of synthetic Bastimolide B (**1**, *aliased to 30 ppm centred at 36 ppm).* Spectrum cut to show relevant correlations used to assign the chemical shift of Bastimolide B stereocenters.

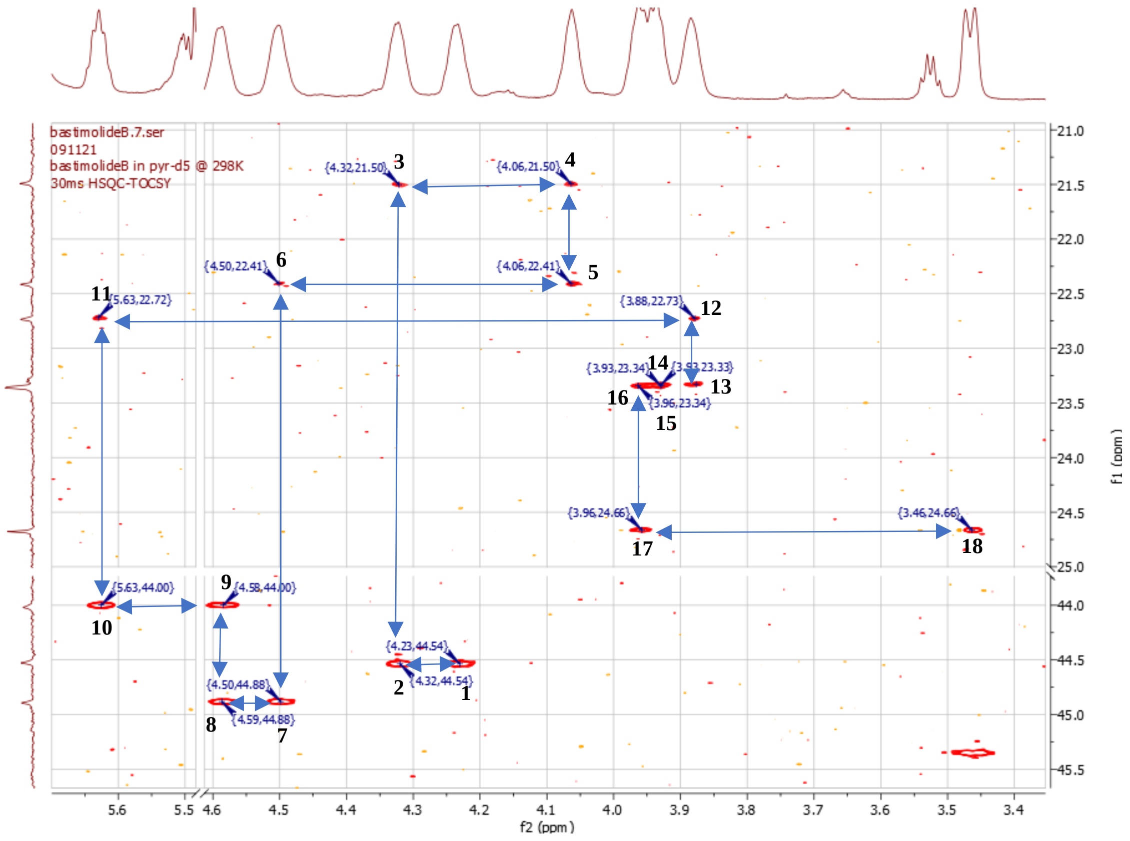

8. References

1) Burchat, A. F.; Chong, J. M.; Nielsen, N. Titration of alkyllithiums with a simple reagent to a blue endpoint. *J. Organomet. Chem.* **1997**, *542*, 281.

2) Krasovskiy, A.; Knochel, P. Convenient Titration Method for Organometallic Zinc, Magnesium, and Lanthanide-Reagents. *Synthesis* **2006**, *2006*, 0890.

3) Beak, P.; Nikolic, N. A. (*R*)-(+)-2-(diphenylhydroxymethyl)pyrrolidine. *Org. Synth.* **1997**, *74,* 23.

4) Larouche-Gauthier, R.; Fletcher, C. J.; Couto, I.; Aggarwal, V. K. Use of Alkyl 2,4,6-Triisopropylbenzoates in the Asymmetric Homologation of Challenging Boronic Esters. *Chem. Commun.* **2011**, *47*, 12592.

5) Shao, C.-L.; Mou, X.-F.; Cao, F.; Spadafora, C.; Glukhov, E.; Gerwick, L.; Wang, C.-Y.; Gerwick, W. H. Bastimolide B, an Antimalarial 24-Membered Marine Macrolide Possessing a tert-Butyl Group. *J. Nat. Prod.* **2018**, *81*, 211.

6) Casoni, G.; Kucukdisli, M.; Fordham, J. M.; Burns, M.; Myers, E. L.; Aggarwal, V. K. α-Sulfinyl Benzoates as Precursors to Li and Mg Carbenoids for the Stereoselective Iterative Homologation of Boronic Esters. *J. Am. Chem. Soc.* **2017**, *139*, 11877.

7) (a) Kliman, L. T.; Mlynarski, S. N.; Ferris, G. E.; Morken, J. P. Catalytic Enantioselective 1,2-Diboration of 1,3-Dienes: Versatile Reagents for Stereoselective Allylation *Angew. Chem., Int. Ed.* **2012**, *51*, 521. (b) Schnell, S. D.; Linden, A.; Gademann, K. Synthesis of Two Key Fragments of the Complex Polyhalogenated Marine Meroterpenoid Azamerone. *Org. Lett.* **2019**, *21*, 1144.

8) Schrof, R.; Altmann, K.-H. Studies Toward the Total Synthesis of the Marine Macrolide Salarin C. *Org. Lett.* **2018**, *20*, 7679

9) Mykura, R. C.; Veth, S.; Varela, A.; Dewis, L.; Farndon, J. J.; Myers, E. L.; Aggarwal, V. K. Investigation of the Deprotonative Generation and Borylation of Diamine-Ligated α-Lithiated Carbamates and Benzoates by in Situ IR spectroscopy. *J. Am. Chem. Soc.* **2018**, *140*, 14677.

10) Kinnaird, J. W. A.; Ng, P. Y.; Kubota, K.; Wang, X.; Leighton, J. L. Strained Silacycles in Organic Synthesis: A New Reagent for the Enantioselective Allylation of Aldehydes. *J. Am. Chem. Soc.* **2002**, *124*, 7920.

11) Hoye, T.; Jeffrey, C; Shao, F. Mosher ester analysis for the determination of absolute configuration of stereogenic (chiral) carbinol carbons. *Nat. Protoc.* **2007**, 2, 2451.

12) Coombs, J. R.; Haefner, F.; Kliman, L. T.; Morken, J. P. Scope and Mechanism of the Pt-Catalyzed Enantioselective Diboration of Monosubstituted Alkenes. *J. Am. Chem. Soc.* **2013**, *135*, 11222.

13) Bonet, A.; Pubill-Ulldemolins, C.; Bo, C.; Gulyás, H.; Fernández, E. Transition-Metal-Free Diboration Reaction by Activation of Diboron Compounds with Simple Lewis Bases. *Angew. Chem., Int. Ed.* **2011**, *50*, 7158.

14) Glaus, F.; Dedic, D.; Tare, P.; Nagaraja, V.; Rodrigues, L.; Aínsa, J. A.; Kunze, J.; Schneider, G.; Hartkoorn, R. C.; Cole, S. T.; Altmann, K.-H. Total Synthesis of Ripostatin B and Structure-Activity Relationship Studies on Ripostatin Analogs *J. Org. Chem.* **2018**, *83*, 7150.
